# Bridging local and global tortuosity of retinal vessels: Objective testing of index performance

**DOI:** 10.1371/journal.pone.0329379

**Published:** 2025-08-07

**Authors:** Natalia Ramírez, Miquel Ralló, Maria S. Millan

**Affiliations:** Group of Applied Optics and Image Processing, Universitat Politècnica de Catalunya–BarcelonaTech, Terrassa, Barcelona, Spain; Coventry University, UNITED KINGDOM OF GREAT BRITAIN AND NORTHERN IRELAND

## Abstract

Retinal vessel tortuosity is a clinically significant parameter that aids in the diagnosis and risk stratification of various ocular and systemic diseases. While local tortuosity indices aim to quantify the winding nature of vessels in the intuitive way perceived by ophthalmologists, their integration into global measures for entire networks remains challenging. This study introduces a novel framework for objectively evaluating global tortuosity indices based on the concept of vessel compositionality. Within this framework, we compare the tortuosity of an unbranched vessel segment to the combined tortuosities of its constituent sub-segments. By analyzing the relationship between local and global tortuosity measures across a representative set of vessel segments, we can objectively assess (using the Spearman rank correlation coefficient) their performance and identify optimal combinations of local indices and global weighting schemes. This approach eliminates the need for subjective assessments of the global tortuosity by specialists, providing a purely mathematical and objective evaluation. Our findings demonstrate the influence of different factors on global tortuosity, including vessel partitioning, local index selection, and weighting schemes. This framework provides a valuable tool for understanding the behavior of tortuosity measures and optimizing their application in clinical settings.

## Introduction

Assessing the global tortuosity of the entire retinal vessel network usually requires methods to integrate local tortuosity measurements. This concept, termed network compositionality by Hart et al. [1], involves partitioning the network into unbranched segments and then combining their individual tortuosities using mathematical formulas. While most research focuses on combining local tortuosity measures based on segment geometric features [1–5], recent work has explored incorporating additional features like vessel type, caliber, and proximity to the fovea and optic disc [6,7]. Defining a global tortuosity measure typically involves three elements: network partition into unbranched segments, a local tortuosity index, and a combination formula. Common segmentation approaches utilize vessel endpoints, bifurcations, and crossings to define segment boundaries [4,8–11]. However, some methods exclude crossings [6,7]. Recent advancements in artificial intelligence have enabled automated assessment of retinal vessel tortuosity [7].

Several studies [1–3,12] have proposed definitions for local tortuosity indices, which are commonly used to quantify the winding nature of retinal vessels, aiming to objectively capture the tortuosity perceived by ophthalmologists during visual examination. Local indices are derived from geometric properties of the vessel centerline, such as length, chord, and curvature. It is important to note that different local tortuosity indices may exhibit varying sensitivities to image features and changes, and may not always align perfectly with visual assessments. Our previous work, for instance, has demonstrated the impact of image resolution [13] and central framing [14] on the behavior of these indices.

Before arriving at a global tortuosity measure, some researchers have identified an intermediate step known as “vessel compositionality,” first introduced by Hart et al. [1] and further explored in subsequent studies [2,3,8]. This concept focuses on a single unbranched vessel segment and involves comparing its overall tortuosity to the combined tortuosities of a set of connected, non-overlapping sub-segments that collectively span the entire segment. This approach allows for a more nuanced evaluation of local tortuosity indices by assessing their performance against specific conceptual criteria within the context of a single vessel.

Hart et al. [2] and Grisan et al. [3] proposed mutually exclusive relationships between the tortuosity of a vessel segment and the tortuosities of its constituent sections, aiming to approximate the intuitive understanding of ophthalmologists. Consequently, no single tortuosity metric consistently satisfies both of these postulates. Researchers are therefore faced with the challenge of selecting a tortuosity index that aligns with their specific conceptual framework. In the section entitled Response of local tortuosity indices to vessel compositionality, we provide some clues through the performance analysis of local tortuosity indices introduced by Hart et al. [1] using simulated vessel segments.

Evaluating the performance of local and global tortuosity measures typically involves comparing them to subjective assessments made by ophthalmologists. These comparisons often utilize statistical methods, such as Spearman rank correlation for ranking order grading [3,4,10] or logistic regression, receiver operative curve (ROC) analysis, and other classifiers, for categorical grading [1,2,5,6,9]. While these methods provide valuable insights, the lack of standardized datasets and diverse expert opinions has hindered the reproducibility and comparability of results. Notably, Grisan et al. [3] addressed this limitation and made a remarkable contribution by publicly releasing a retinal vessel tortuosity dataset at BioImLab (http://bioimlab.dei.unipd.it). This dataset has provided a valuable resource for researchers, offering cropped retinal images of arteries and veins with ranked tortuosity assessments from a single ophthalmologist.

The framework of vessel compositionality allows us to introduce a novel approach for evaluating the performance of global tortuosity indices. By focusing on individual unbranched vessel segments, we can directly assess their tortuosity using a local index. Subsequently, these segments can be divided into smaller sections using bifurcation points, crossings, and endpoints. A global tortuosity value for the segment can then be calculated by combining the local tortuosities of these constituent sections using a predefined formula. This approach enables an objective performance assessment by statistically analyzing the relationship between pairs of local and global tortuosity measures across a representative set of vessel segments. Furthermore, this study provides a mathematical validation of global indices, allowing us to identify, for a given local index, the global scheme that yields the highest Spearman correlation, effectively determining the closest ranking order. This approach offers a significant advantage by eliminating the need for subjective assessments from specialists, providing a purely mathematical and objective evaluation.

We additionally investigate the impact of different segment partitions on the resulting global tortuosity measure by comparing the outcomes obtained from two distinct partitioning schemes of a simulated vessel segment. We utilize the vessel compositionality framework to evaluate the performance of global tortuosity indices based on the criteria established by Hart et al. [2] and Grisan et al [3]. Our analysis employs both simulated vessels and the retinal artery dataset from BioImLab to illustrate these concepts. While we include conventional comparisons with the dataset ophthalmologist’s ranking for completeness, it is worth emphasizing that our primary focus lies on the objective and mathematically grounded evaluation of local and global tortuosity indices within the framework of vessel compositionality.

## Response of local tortuosity indices to vessel compositionality

### Local indices

This study considers the local tortuosity indices included by Hart et al. [1,2]. Let C(t)=(x(ttextrm,y(t)) with $\text{\textbackslash{} }t_{\text{0}}\leq t\leq t_{\text{1}}$ be a parameterized curve describing the centerline of an unbranched retinal vessel segment. Its curvature κ(t can be expressed in terms of the first $\begin{array}{c} \left(x^{\prime }(t)\text{, }y^{\prime }(t)\right) \end{array}$ and second $\begin{array}{c} \left(x^{\prime \prime }(t)\text{, }y^{\prime \prime }(t)\right) \end{array}$ derivatives, according to


$$\kappa (t)\text{ = }\frac{{\text{y}}^{\prime \prime }(t)\cdot {\text{x}}^{\prime }(t)\text{ - }{\text{y}}^{\prime }(t)\cdot \;x^{\prime \prime }(t)}{{\left({{\text{x}}^{\prime }(t)}^{\text{2}}\text{ + }{{\text{y}}^{\prime }(t)}^{\text{2}}\right)}^{\frac{\text{3}}{\text{2}}}}\;\text{.}$$ (1)

The tortuosity indices can be formulated in terms of several geometric features of this parametric curve: distance between endpoints or chord (D), curve length (L), total curvature (TK), and total squared curvature (TSK), as presented in Table 1. Table 2 contains the definitions of the eight local tortuosity indices studied in this work. Application of these formulas to digital images involves the estimation of the first and second derivatives for discrete x(t) and y(t). Our estimations resulted from the second order Taylor’s expansion of these functions. The procedure is fully described elsewhere [14].

**Table 1:** Geometric features involved in the definitions of local tortuosity indices [1,2].

Chord	$D(Ctextrm=\sqrt{{\left(x\left(t_{\text{1}}\right)\text{ - }x(t_{\text{0}})\right)}^{\text{2}}\text{ + }{\left(y\left(t_{\text{1}}\right)\text{ - }y(t_{\text{0}})\right)}^{\text{2}}}$
Curve length	$L(Ctextrm=\int_{C}^{\text{\textbackslash{} }}\text{1}ds\text{ = }\int_{t_{\text{0}}}^{t_{\text{1}}}\text{1}\cdot \sqrt{{{\text{x}}^{\prime }(t)}^{\text{2}}\text{ + }{{\text{y}}^{\prime }(t)}^{\text{2}}}\text{\textbackslash{} }dt$
Total curvature	$TK(Ctextrm=\int_{C}^{\text{\textbackslash{} }}|\kappa |ds\text{ = }\int_{t_{\text{0}}}^{t_{\text{1}}}\left|\kappa (t)\right|\cdot \sqrt{{{\text{x}}^{\prime }(t)}^{\text{2}}\text{ + }{{\text{y}}^{\prime }(t)}^{\text{2}}}\text{\textbackslash{} }dt$
Total squared curvature	$TSK(Ctextrm=\int_{C}^{\text{\textbackslash{} }}\kappa^{\text{2}}ds\text{ = }\int_{t_{\text{0}}}^{t_{\text{1}}}\kappa^{\text{2}}(t)\cdot \sqrt{{{\text{x}}^{\prime }(t)}^{\text{2}}\text{ + }{{\text{y}}^{\prime }(t)}^{\text{2}}}\text{\textbackslash{} }dt$

**Table 2:** Definitions of eight local tortuosity indices in terms of the geometric features listed in Table 1 [1,2].

Local tortuosity index	DF	$T_{\text{1}}$	$T_{\text{2}}$	$T_{\text{3}}$	$T_{\text{4}}$	$T_{\text{5}}$	$T_{\text{6}}$	$T_{\text{7}}$
Expression	$\frac{L}{D}$	$\frac{L}{D}\text{ - 1}$	TK	TSK	$\frac{TK}{L}$	$\frac{TSK}{L}$	$\begin{array}{c} \frac{TK}{D} \end{array}$	$\begin{array}{c} \frac{TSK}{D} \end{array}$

### Two opposite criteria of vessel compositionality

Vessel compositionality establishes a vessel feature from those of its constituent parts. Let C be an unbranched vessel segment composed of two vessel sections $C_{\text{1}}$ and $C_{\text{2}}$ smoothly connected (i.e., with continuous first derivative), $C\text{ = }C_{\text{1}}⊗C_{\text{2}}.\;$ And let $\tau (C),\;\tau \left(C_{\text{1}}\right)$ and $\tau \left(C_{\text{2}}\right)$ be their corresponding tortuosity measures, according to some local tortuosity index τ. What should be the tortuosity τ(C) of the whole vessel segment in terms of $\tau \left(C_{\text{1}}\right)$ and $\tau \left(C_{\text{2}}\right)$? According to Hart et al. [2], τ(C) should lie between the values $\tau \left(C_{\text{1}}\right)$ and $\tau \left(C_{\text{2}}\right)$, that is,


$$Hart\;inequality\;min\left(\tau \left(C_{\text{1}}\right)\text{,}\tau \left(C_{\text{2}}\right)\right)\leq \text{\textbackslash{} }\tau (C)\leq \text{max}\left(\tau \left(C_{\text{1}}\right)\text{,}\tau \left(C_{\text{2}}\right)\right)\text{,}$$ (2)

the equality holding only when . The authors asserted that any tortuosity measure consistent with the intuition of ophthalmologists should satisfy this inequality. They proposed to compute the tortuosity of the whole segment C\ from the tortuosity values $\tau \left(C_{\text{1}}\right)$ and $\tau \left(C_{\text{2}}\right)$ of its constituent sections as a weighted average (named “weighted additivity” in [2]), the weights being determined by the fraction of arc length that each section contributes to the vessel segment:


$$\tau_{WA}(C)\text{ = }\frac{L\left(C_{\text{1}}\right)\cdot \tau \left(C_{\text{1}}\right)\text{ + }L\left(C_{\text{2}}\right)\cdot \tau \left(C_{\text{2}}\right)}{L\left(C_{\text{1}}\right)\text{ + }L\left(C_{\text{2}}\right)},\;$$ (3)

where $L\left(C_{i}\right)$ is the arc length of the curve $C_{i}$, i = 1,2. Equation (2), together with the assumption that the tortuosity of a vessel should be independent of how it is split into parts led the authors to an inconsistency, which they expressed for a vessel segment C composed of four sections $C_{\text{1}}..C_{\text{4}}$: “Let , and consider the curves $C_{\text{1}}⊗C_{\text{2}}⊗C_{\text{3}}$ and $C_{\text{2}}⊗C_{\text{3}}⊗C_{\text{4}}$. If we segment these curves as $C_{\text{1}}⊗\left(C_{\text{2}}⊗C_{\text{3}}\right)$ and $\left(C_{\text{2}}⊗C_{\text{3}}\right)⊗C_{\text{4}}$, then the tortuosity measures for these two curves are different. However, if these curves are segmented as $\left(C_{\text{1}}⊗C_{\text{2}}\right)⊗C_{\text{3}}$ and $C_{\text{2}}⊗\left(C_{\text{3}}⊗C_{\text{4}}\right)$, the tortuosities of the constituent segments of the two vessels are the same, viz. $\tau \left(C_{\text{1}}\right)$ and $\tau \left(C_{\text{3}}\right)$.” They concluded that the tortuosity of a vessel segment cannot depend exclusively on the tortuosity values of its constituent sections. They also introduced the property of chord-colinear compositionality for vessel extensions that follow a tortuosity pattern (e.g., retinal vessels are often roughly periodic along a centerline). Based on this property, if a vessel C is segmented such that each part has the same tortuosity and the chords of the parts are colinear, then the tortuosity of the vessel is the same as the constituent parts.

Grisan et al. [3] did not accepted the statement of Eq. 2 and illustrated their dissension with an example: a simulated vessel segment C with three turns composed by three smoothly connected semicircle sections of same tortuosity . Assuming the principle of invariance with respect to rotation and scale, the authors observed that while the individual sections were perceived as non-tortuous, when connected ${C=C}_{\text{1}}⊗C_{\text{2}}⊗C_{\text{3}}$, the resulting vessel was far more tortuous than any of its sections. However, taking into account Hart inequality (Eq. 2), .

Therefore, Grisan et al. stated that the tortuosity of a vessel segment should not be less than any of its composing parts:


$$Grisan\;inequality\text{\textbackslash{} \textbackslash{} \textbackslash{} \textbackslash{} \textbackslash{} }\text{max}\left(\tau \left(C_{\text{1}}\right)\text{,}\tau \left(C_{\text{2}}\right)\text{,}\tau \left(C_{\text{3}}\right)\right)\leq \text{\textbackslash{} }\tau (C).$$ (4)

The key concept behind Grisan’s idea of tortuosity is that of turn curves, sections of a vessel segment with constant curvature sign. $C=C_{\text{1}}⊗C_{\text{2}}⊗C_{\text{3}}$ has three turn curves, whereas $C_{\text{1}}$ has just one. The higher the number of turn curves, the higher the tortuosity should be.

Note that Hart and Grisan inequalities (Eqs. 2 and 4) cannot be fulfilled simultaneously when $\tau \left(C_{\text{i}}\right)$ are not the same value. The two mutually exclusive mathematical approaches respond to different concepts of tortuosity. In terms of (absolute) curvature, Grisan’s approach seems to be closer to total curvature, while Hart’s seems closer to mean curvature.

### Performance of local indices

**Fig 1:**
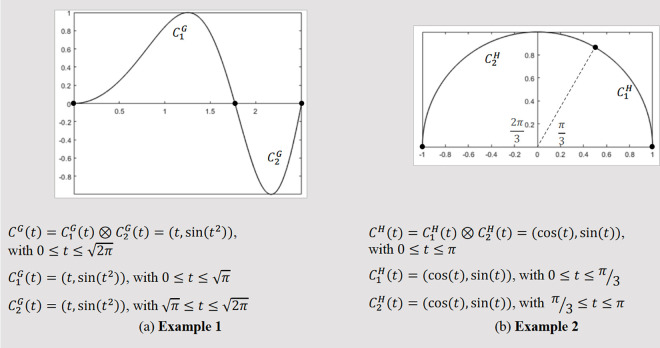
Examples used to test the response of local indices $DF,\;T_{1},\;T_{6},and\;T_{7}\;$ to Grisan and Hart inequalities.

We investigated which inequality, Hart (Eq. 2) or Grisan (Eq. 4), is satisfied by the local tortuosity indices $DF,\;T_{\text{1}}\ldots \text{\textbackslash{} }T_{\text{7}}$ (Table 2). As demonstrated below with an example, some local indices do not consistently fulfill either inequality. To illustrate this, consider two parametric curves, $C^{G}(t\text{and}\;C^{H}(t)$ (Fig 1), and their corresponding sections. Table 3 presents the geometric features and tortuosity values for these curves, calculated using the $DF,\;T_{\text{1}},\;T_{\text{6}}$, and $T_{\text{7}}$ indices.

**Table 3:** Geometric features, local tortuosity $DF,\;T_{1},\;T_{6}$, and $T_{7}$ values, and Hart and Grisan performance criteria for the examples of Fig 1.

	Example 1					Example 2				
Geometric features	$C_{1}^{G}$	$C_{2}^{G}$	$C^{G}$			$C_{1}^{H}$	$C_{2}^{H}$	$C^{H}$		
D	$\sqrt{\pi }$	$\sqrt{2\pi }-\sqrt{\pi }$	$\sqrt{2\pi }$			1	$\sqrt{3}$	2		
L	2.830	2.181	5.011			$\frac{\pi }{3}$	$\frac{2\pi }{3}$	π		
TK	2.540	1.945	4.485			$\frac{\pi }{3}$	$\frac{2\pi }{3}$	π		
TSK	8.185	21.990	30.174			$\frac{\pi }{3}$	$\frac{2\pi }{3}$	π		
**Local index**				**Hart** **inequality**	**Grisan inequality**				**Hart inequality**	**Grisan inequality**
DF	1,597	2,971	1,999	yes	no	1,047	1,209	1,571	no	yes
$T_{1}$	0,597	1,971	0,999	yes	no	0,047	0,209	0,571	no	yes
$T_{6}$	1,433	2,649	1,789	yes	no	1,047	1,209	1,571	no	yes
$T_{7}$	4,618	29,952	12,038	yes	no	1,047	1,209	1,571	no	yes

Table 3 shows that local indices $DF,\;T_{\text{1}},\;T_{\text{6}}$, and $T_{\text{7}}$ satisfy Hart inequality (Eq. 2) for the curve and sections in Example 1 (Fig 1) but not for those in Example 2. Conversely, these indices do not meet Grisan inequality (Eq. 4) in Example 1 but do so in Example 2. These two curves, $C^{G}(t\text{and}\;C^{H}(t)$, show that the compliance of local tortuosity indices $DF,\;T_{\text{1}},\;T_{\text{6}}$, and $T_{\text{7}}$ with Hart or Grisan inequalities is not consistent, but depends on the specific vessel path under consideration.

Let us remind some geometrical properties of compositionality prior to test the response of local indices $T_{\text{2}},\;T_{\text{3}},\;T_{\text{4}}$, and $T_{\text{5}}$ to Hart and Grisan inequalities. From the definitions of length, total curvature and total squared curvature as definite integrals (Table 1), it follows directly that their values for a vessel segment equal the summation of the values of its constituent sections (i.e., $L(C)=\sum L\left(C_{\text{i}}\right)$, $TK(C)=\sum TK\left(C_{\text{i}}\right)$, $TSK(C)=\sum TSK\left(C_{\text{i}}\right)$). For the chord, however, the triangular inequality leads to state $D(C)\leq \sum D\left(C_{\text{i}}\right)$.

From the definitions of local indices $T_{\text{2}}(Ctextrm=TK(C)$ and $T_{\text{3}}(Ctextrm=TSK(C)$ as definite integrals of a positive function (Table 1), they satisfy Grisan inequality (Eq. 4), in general, for any vessel segment composed by individual sections. However, from the definition $T_{\text{4}}=\frac{TK}{L}$, it follows the identity $T_{4}(C)=\sum T_{\text{4}}\left(C_{\text{i}}\right)\frac{L\left(C_{\text{i}}\right)}{L(C)}$, which is a weighted average of the tortuosity value of its constituent sections $T_{\text{4}}\left(C_{\text{i}}\right)$, with weights equal to their length fractions $\frac{L\left(C_{\text{i}}\right)}{L(C)}$. Therefore, ${\text{min}\left(T_{\text{4}}\left(C_{\text{i}}\right)\right)\leq T}_{4}(C)\leq \text{max}\left(T_{\text{4}}\left(C_{\text{i}}\right)\right)$, which is the Hart inequality. The same reasoning holds for the local index $T_{\text{5}}=\frac{TSK}{L}$. All these results are summarized in Table 4. While the concepts of tortuosity underlying $T_{\text{2}}$ and $T_{\text{3}}$ are aligned with Grisan’s proposal, those underlying $T_{\text{4}}$ and $T_{\text{5}}$ are aligned with Hart’s. Since the definitions of $T_{\text{6}}$ and $T_{\text{7}}$ are close to the definitions of $T_{\text{4}}$ and $T_{\text{5}}$ respectively, their performance should be also relatively close to Hart’s concept of tortuosity. DF\ and $T_{\text{1}}$ are not aligned with any of these two concepts. We will provide further insight after applying the local tortuosity indices to dataset vessel segments in next sections.

**Table 4:** Criterion compliance of local indices when applied to a generic segment composed by two or more sections.

Performance criterion	DF	$T_{\text{1}}$	$T_{\text{2}}$	$T_{\text{3}}$	$T_{\text{4}}$	$T_{\text{5}}$	$T_{\text{6}}$	$T_{\text{7}}$
Hart inequality (Eq. 2)	No	No	No	No	Yes	Yes	No	No
Grisan inequality (Eq. 4)	No	No	Yes	Yes	No	No	No	No

### Global tortuosity

Handayani et al. [4] aimed to find an optimal weighting scheme of local tortuosities to build a global tortuosity measure. They firstly stated the weaknesses of two existing weighting schemes: the mean of the tortuosity values (MT) calculated on individual sections [10,11] and the weighted additivity (Eq. 3) proposed by Hart et al. While the mathematical mean does not distinguish contributions from sections of different lengths, the weighted additivity only considers the crude arc length of the vessel sections, failing to appreciate the complexity of the vascular network, in particular, the amount of possible branching points. These facts led them to propose three new weighting schemes (WS): the mean tortuosity with weighted additivity (MTWA), the weighted additivity on chord (WAC) and tortuosity density global (TDG). These three schemes are formulated in Table 5 along with MT and weighted additivity on length (WAL). Note that ‘on length’ has been added for clarity. This clarifies that WAL (Weighted Additivity on Length) is equivalent to the concept of weighted additivity (WA) introduced by Hart et al. in Eq. 3. [2]. All mathematical expressions in Table 5 are formulated for a vessel segment C, which is partitioned into a set of N unbranched sections $C_{i}$, where i=1, … , N. The WS are expressed in terms of local tortuosity values $\tau \left(C_{i}\right)$, with τ representing a particular index within the eight studied $\tau \text{ = }\left\{DF,\;T_{\text{1}}\text{,}\ldots \text{,}T_{\text{7}}\right\}$.

**Table 5:** Weighting schemes (WS) to build a global measure from local tortuosity values [4]. $C_{i}$, with i=1, … , N represents a partition of the vessel segment C. Length L and chord D are defined in Table 1. Local tortuosity indices are represented by $\tau =\left\{DF,\;T_{1},\ldots ,T_{7}\right\}$ (Table 2).

Mean Tortuosity (MT)	$\tau_{MT}(Ctextrm=\frac{\sum_{i\text{ = 1}}^{N}\tau \left(C_{i}\right)}{N}$	(5)
Weighted Additivity on Length (WAL)	$\tau_{WAL}(Ctextrm=\frac{\sum_{i\text{ = 1}}^{N}L\left(C_{i}\right)\cdot \tau \left(C_{i}\right)}{\sum_{i\text{ = 1}}^{N}L\left(C_{i}\right)}$	(6)
Mean Tortuosity with Weighted Additivity (MTWA)	$\tau_{MTWA}(Ctextrm=\frac{\sum_{i\text{ = 1}}^{N}L\left(C_{i}\right)\cdot \tau \left(C_{i}\right)}{N\cdot \sum_{i\text{ = 1}}^{N}L\left(C_{i}\right)}$	(7)
Weighted Additivity on Chord (WAC)	$\tau_{WAC}(Ctextrm=\frac{\sum_{i\text{ = 1}}^{N}D\left(C_{i}\right)\cdot \tau \left(C_{i}\right)}{\sum_{i\text{ = 1}}^{N}D\left(C_{i}\right)}$	(8)
Tortuosity Density Global index (TDG)	$\tau_{TDG}(Ctextrm=\frac{\sum_{i\text{ = 1}}^{N}\tau \left(C_{i}\right)}{N\cdot \sum_{i\text{ = 1}}^{N}L\left(C_{i}\right)}$	(9)

The set of all combinations of local tortuosity indices and formulas results in some redundancies. From the definitions, it is straight to find that ${\left[T_{\text{2}}\right]}_{TDG}(Ctextrm={\left[T_{\text{4}}\right]}_{MTWA}(C)$ and ${\left[T_{\text{3}}\right]}_{TDG}(Ctextrm={\left[T_{\text{5}}\right]}_{MTWA}(C)$.

Two issues arise from these definitions. One is the interconnection between the global tortuosity measure yielded by a given WS and the local tortuosity index. The other is the dependence of the global tortuosity WS on the partition.

Regarding the first issue, we compare the global tortuosity values $\tau_{MT}(C)$, $\tau_{WAL}(C)$, $\tau_{MTWA}(C)$, $\tau_{WAC}(C)$, and $\tau_{TDG}(C)$, computed from the local tortuosity values $\tau \left(C_{i}\right)$ of the composing unbranched segments, i=1, … , N, and study whether they meet the Hart (Eq. 2) or Grisan (Eq. 4) inequalities for every local tortuosity index of the set $\tau \text{ = }\left\{DF,\;T_{\text{1}},\ldots ,T_{\text{7}}\right\}$. Note that $\tau_{MT}(C)$, $\tau_{WAL}(C)$, and $\tau_{WAC}(C)$ are all weighted means, i. e., linear combinations of $\tau \left(C_{i}\right)$, i=1, … , N, with respective coefficients (weights): $w_{MT\text{,}i}\;=\frac{\text{1}}{N}$, $w_{WAL\text{,}i}\;=\frac{L\left(C_{i}\right)}{L(C)}$ and $w_{WAC\text{,}i}\;=\frac{D\left(C_{i}\right)}{\sum_{i=1}^{N}D\left(C_{i}\right)}$. Therefore, all three satisfy a Hart inequality (Eq. 2), that is, $\text{min}\left(\tau \left(C_{i}\right)\right)\leq \left\{\tau_{MT}(C),\;\tau_{WAL}(C),\;\tau_{WAC}(C)\right\}\;\leq max(\tau \left(C_{i}\right))$, for all the studied local indices. Taking into account that $\tau_{MTWA}(Ctextrm=\frac{\tau_{WAL}(C)}{N}$ and $\tau_{TDG}(Ctextrm=\frac{\tau_{MT}(C)}{L(C)}$, we obtain two inequations: $\frac{\text{min}\left(\tau \left(C_{i}\right)\right)}{N}\leq \;\tau_{MTWA}(C\leq \frac{max(\tau \left(C_{i}\right))}{N}$ and $\frac{\text{min}\left(\tau \left(C_{i}\right)\right)}{L(C)}\leq \tau_{TDG}(C)\leq \frac{max(\tau \left(C_{i}\right))}{L(C)}$, which can be identified as scaled versions of Hart inequality. The first one anticipates an undesired effect caused by the partition of the vessel segment into a number N of sections: the higher the N, the smaller the global measure $\tau_{MTWA}(C)$. Analogously for the second inequality, the longer the length L(C) for any vessel geometry, the smaller the global measure $\tau_{TDG}(C)$.

We illustrate the second issue, i.e., the dependence of the global tortuosity on the partition, with an example: let us consider a simulated vessel segment C(t)=(x(t),y(t)), consisting of three connected semicircles of decreasing radii, as defined by the parametrization of expression


$$x(t)\text{ = }\left\{ \begin{array}{cc} 4-4\cdot cos(t) & 0\leq t\leq \pi \\ 10-2\cdot cos(t) & \pi \leq t\leq 2\pi \\ 13-cos(t) & \text{2}\pi \leq t\leq 3\pi \end{array}\right.\;y(t)\text{ = }\left\{ \begin{array}{cc} 4\cdot sin(t) & 0\leq t\leq \pi \\ 2\cdot sin(t) & \pi \leq t\leq 2\pi \\ sin(t) & \text{2}\pi \leq t\leq 3\pi \end{array}\right.\;$$ (10)

**Fig 2:**
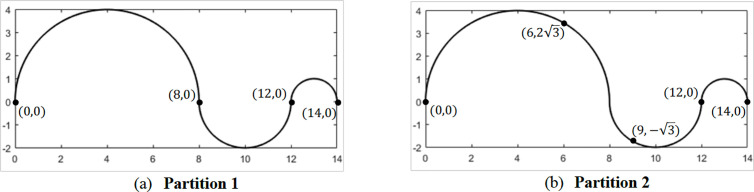
Vessel segment centerline parametrized by Eq. 10, with different partitions: (a) Partition 1, with three sections; (b) Partition 2, with four sections.

Two different partitions for this vessel segment are depicted in Fig 2: Partition 1 has three sections, limited by points {(0,0),(8,0),(12,0),(14,0)} (Fig 2a), while Partition 2 has four sections, limited by points $\left\{(0,0),\left(6,2\sqrt{3}\right),\left(9,-\sqrt{3}\right),(12,0),\;(14,0)\right\}$ (Fig 2b). For these two partitions, Table 6 contains the section’s geometric features and the local tortuosity values, and Table 7 the global tortuosity values computed for all WS (Eqs. 5–9) and local indices. Table 7, first raw, contains the local tortuosity calculated for the entire vessel segment considered as one section.

**Table 6:** Geometric features and local tortuosity of the sections depicted in partitions 1 and 2 of the segment of Fig 2.

	Sections					
	(0,0) to (8,0)	(8,0) to (12,0)	(12,0) to (14,0)	$(0,0to\left(6,2\sqrt{3}\right)$	$\left(6,2\sqrt{3}\right)to\left(9,-\sqrt{3}\right)$	$\left(9,-\sqrt{3}\right)to\;(12,0)$
Geometric features						
D	8	4	2	$4\sqrt{3}$	6	$2\sqrt{3}$
L	4π	2π	π	8π/3	2π	4π/3
TK	π	π	π	2π/3	2π/3	2π/3
TSK	π/4	π/2	π	π/6	π/4	π/3
Local tortuosity						
DF	π/2	π/2	π/2	$2\pi /3\sqrt{3}$	π/3	$2\pi /3\sqrt{3}$
$T_{\text{1}}$	(π/2)−1	(π/2)−1	(π/2)−1	$\left(2\pi /3\sqrt{3}\right)-1$	(π/3)−1	$\left(2\pi /3\sqrt{3}\right)-1$
$T_{2}$	π	π	π	2π/3	2π/3	2π/3
$T_{3}$	π/4	π/2	π	π/6	π/4	π/3
$T_{4}$	1/4	1/2	1	1/4	1/3	1/2
$T_{5}$	1/16	1/4	1	1/16	1/8	1/4
$T_{6}$	π/8	π/4	π/2	$\pi /6\sqrt{3}$	π/9	$\pi /3\sqrt{3}$
$T_{7}$	π/32	π/8	π/2	$\pi /24\sqrt{3}$	π/24	$\pi /6\sqrt{3}$

**Table 7:** Global tortuosity of the vessel with partitions 1, 2 (Fig 2). The WS are calculated on the basis of the local tortuosity indices. First raw contains the tortuosity values for a one section vessel segment.

Global tortuosity									
		Local index (τ)							
Vessel Partition		DF	$T_{\text{1}}$	$T_{\text{2}}$	$T_{\text{3}}$	$T_{\text{4}}$	$T_{\text{5}}$	$T_{\text{6}}$	$T_{\text{7}}$
One section (unpartitioned)									
τ(C)		1,5708	0,5708	9,4248	5,4978	0,4286	0,2500	0,6732	0,3927
Partition 1 3 sections (Fig. 2a)									
Weighting scheme	$\tau_{MT}(C)$	1,5708	0,5708	3,1416	1,8326	0,5833	0,4375	0,9163	0,6872
	$\tau_{WAL}(C)$	1,5708	0,5708	3,1416	1,3464	0,4286	0,2500	0,6732	0,3927
	$\tau_{MTWA}(C)$	0,5236	0,1903	1,0472	0,4488	0,1429	0,0833	0,2244	0,1309
	$\tau_{WAC}(C)$	1,5708	0,5708	3,1416	1,3464	0,4286	0,2500	0,6732	0,3927
	$\tau_{TDG}(C)$	0,0714	0,0260	0,1429	0,0833	0,0265	0,0199	0,0417	0,0313
Partition 2 4 sections (Fig. 2b)									
Weighting scheme	$\tau_{MT}(C)$	1,2591	0,2591	2,3562	1,3744	0,5208	0,3594	0,7067	0,5199
	$\tau_{WAL}(C)$	1,2146	0,2146	2,2440	1,0721	0,4286	0,2500	0,5545	0,3482
	$\tau_{MTWA}(C)$	0,3037	0,0537	0,5610	0,2680	0,1071	0,0625	0,1386	0,0870
	$\tau_{WAC}(C)$	1,1957	0,1957	2,2083	0,9923	0,4058	0,2201	0,5124	0,2989
	$\tau_{TDG}(C)$	0,0573	0,0118	0,1071	0,0625	0,0237	0,0163	0,0321	0,0236

Most global tortuosity measures exhibit differences between partitions 1 and 2. However, the measure $\tau_{WAL}(C)$ WS (6), utilizing indices $T_{\text{4}}$ and $T_{\text{5}}$ (i.e., ${\left[T_{\text{4}}\right]}_{WAL}(C)$ and ${\left[T_{\text{5}}\right]}_{WAL}(C)$), yields identical global values for both partitions, as expected based on their definitions and properties. These values are highlighted in Table 7. They also match those values obtained for a single, unpartitioned vessel segment. In this case, the local values $T_{\text{4}}$ and $T_{\text{5}}$ (also highlighted in Table 7) effectively become the global measurements.

An increase in the number of sections generally leads to lower global tortuosity values for most combinations of WS and indices. This observation highlights the significant influence of the chosen partition on the computed global tortuosity of a vessel segment, and consequently, of a vessel network. The $\tau_{MTWA}(C)$ WS (7) exhibited the highest relative differences between the two partitions. Among the local indices, WS based on $T_{\text{1}}\;$ accounted for the highest relative differences.

The highest relative differences between the unpartitioned vessel tortuosity τ(C) (Table 7, first row) and the global tortuosities calculated using various WS were observed for $\tau_{TDG}(C)$, followed by $\tau_{MTWA}(C)$ and $\tau_{MT}(C)$, regardless of the number of sections in partitions 1 and 2. Among the local indices, WS based on $T_{\text{2}}$ and $T_{\text{3}}$ accounted for the highest relative differences with the unpartitioned tortuosity values.

It is important to note that the total curvature of the entire simulated segment $T_{\text{2}}(C)$ is greater than any WS based on total curvature calculated for partitions 1 and 2, regardless of whether the segment is divided into three or four sections. Consequently, ${\left[T_{\text{2}}\right]}_{MT}(C)$, i.e., the mean of the section values $T_{\text{2}}(C_{i})$, is smaller than the total curvature of the entire vessel segment $T_{\text{2}}(C)$. Similar arguments apply to other WS formulas and the squared total curvature index $T_{\text{3}}$.

## Method

We provide a method for an objective evaluation of the local tortuosity indices and the global tortuosity WS. We use a dataset for illustrative reasons.

### Dataset

The need for free public image datasets was claimed by Abdalla et al. [15] to be one of the main problems for the evaluation of retinal blood vessel tortuosity. They reviewed public and private datasets used in the field up to 2015. While the RET-TORT dataset from BioIm Lab (http://bioimlab.dei.unipd.it) (Padova, Italy) appears to be the only publicly available option in recent years, it has faced discontinuity. Public and private datasets often differ significantly in vessel characteristics (type, length, caliber), segmentation techniques, sample size, and patient pathologies. Additionally, many studies rely on subjective assessments by individual ophthalmologists or specialist teams, making comparisons between studies challenging. This inherent subjectivity emphasizes the importance of objective methodologies like the one employed in this study.

RET-TORT database consists of 30 artery segments and 30 vein segments from normal retina or affected by hypertensive retinopathy of different severity [3], manually ordered in the two sets on the basis of increasing tortuosity by Dr. S. Piermarocchi (dataset ophthalmologist), a retinal specialist of the Department of Ophthalmology of the University of Padova (Italy). The images were captured with a 50° fundus camera (TRC 50, Topcon, Japan) and digitized to a resolution of 1100 × 1300 pixels using a scanner, in TIF format. Vessel segments of similar length and caliber were extracted from the major retinal arteries or veins that exhibit minimal overlap or entangling with other surrounding blood vessels. The dataset provides two more files: “ClinicalOrdering.xls,” which contains the ranked images in increasing tortuosity as assigned by the dataset ophthalmologist, and “ManualData.mat,” with coordinate samples of the manually traced vessel centerlines, interpolated through cubic smoothing splines.

### Vessel parametrization and partition

This section describes the method followed to analyze the 30 RET-TORT dataset arteries and compute their local and global tortuosity with all combination pairs of local tortuosity indices and global tortuosity WS.

**Fig 3:**
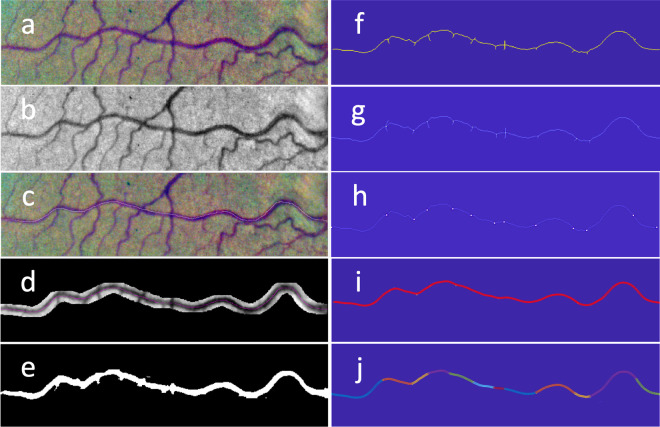
Steps for vessel segment parametrization and partition into sections. (a) original BioImLab image artery No.48; (b) green channel; (c) manual trace; (d) ROI; (e) binary segmentation; (f) skeletonization; (g) identification of endpoints, crossing points and bifurcations points (bright points); (h) vessel centerline after removing secondary branches; (i) smoothed segment to be parametrized; (j) partition into (colored) sections defined by division points.

Vessel segmentation and parametrization was performed following the procedure described in detail in [14] and summarized here for the sake of clarity. Fig 3 illustrates the main steps of this procedure for the color image of the dataset artery No. 48 (146×552 pixel size) (Fig 3a). Segmentation was done using the green component of the eye fundus image because it typically shows enhanced contrast (Fig 3b). We used a customized tool coded with MATLAB (2021a version; Natick, Massachusetts: The MathWorks Inc.) that allows to roughly trace a line along the vessel path (Fig 3c). A region of interest (ROI) was cleared out from the image by setting a surrounding area of 7 pixels in both the X and Y directions from the traced line (Fig 3d). We used the segmentation method described in [16] to segment the vessel contained in the ROI. This unsupervised binarization method is based on an iterative algorithm. Starting from a seed, the algorithm adds a new vessel segment connected to the previously segmented part at each iteration. The result preserves connectivity, which is a distinct feature of the retinal vessel tree. A morphological closing operation is applied to smooth small irregularities (Fig 3e). For the morphological closing, we used a circular structural element whose radius approximated the average width of the dataset vessels (about 7 pixels). This step was followed by skeletonization (Fig 3f). We used a thinning algorithm implemented in the MATLAB Image Processing Toolbox (more specifically, the bwmorph function with ‘thin’ and Inf’ options) to reduce the vessel traces to single-pixel-wide skeletons.

Endpoints, bifurcation points, and crossing points were identified in the skeletonized element (Fig 3g) according to the following criteria: pixels with one neighbor pixel only were labeled as endpoints, whereas pixels with three or more neighbor pixels were labeled as tree-branching pixels (either bifurcation or crossing). If multiple connected tree-branching pixels resulted, their centroid was selected to represent the group. Endpoints of the main vessel segment, located at both the left and right extremes of the image, were identified amongst all endpoints. Spurious branches were removed by an iterative procedure: secondary endpoints, that is, endpoints other than the left and right image extremes, were removed from the skeleton; in the next iteration, new secondary endpoints were found and further removed. The procedure was repeated until no endpoints other than the left and right image extremes remained (Fig 3h). The resulting 1-pixel wide centerline was parametrized as a string of its pixel coordinates and then smoothed with a three-term moving average (Fig 3i). Division points, corresponding to the original bifurcation and crossing points, were identified as the points on the smoothed line (Fig 3i) closest to their respective counterparts on the skeletonized segment (bright points in Fig 3h). We added the endpoints of the smoothed line to the list of division points. Fig. 3j shows the resulting partition of the vessel segment into a number of composing sections. We parametrized these sections according to the smoothed line. The application of this procedure to the 30 arteries in the RET-TORT dataset yielded intermediate results analogous to those shown in Figs 3d, 3f, 3h, 3i, and 3j, which are available in a supporting information file (S1 Appendix).

Although the dataset provided smoothed, spline-interpolated vessels in the ManualData.mat file, we adhered to the vessel processing procedure outlined in our previous work [13,14,16] and summarized in Fig 3. This procedure differs from the smoothing approach employed by Grisan et al. [3], potentially preserving minor oscillations that could slightly increase tortuosity values, particularly those based on curvature or squared curvature. However, we consider this a minor factor in our analysis, as our primary focus is on the correlation between rankings rather than the absolute values of tortuosity.

### Tortuosity calculation

As detailed for a simulated vessel in Fig 2, the discrete parametrization of each vessel segment was followed by the calculation of geometric features {D, L, TK, TSK} (Table 1) and local tortuosity with the local indices $\tau \text{ = }\left\{DF,\;T_{\text{1}}\text{,}\ldots \text{,}T_{\text{7}}\right\}$ (Table 2) for both the entire (one-section or unpartitioned) segment and for the sections created by the division points. Following this, global tortuosity was calculated using the weighting schemes $WS=\left\{\tau_{MT},\;\tau_{WAL},\tau_{MTWA},\tau_{WAC},\tau_{TDG}\right\}$ (Table 5) for the partitioned segments. The process was repeated for the 30 arteries of the BioImLab RET-TORT dataset. The results provided tortuosity rankings for objective comparison and evaluation using statistics, such as the Spearman rank correlation coefficient. Former studies used this coefficient to find out whether clinical rankings were in agreement with measures yielded by tortuosity indices [3,4,10]. For completeness, we include further comparison with the ranking done by the dataset ophthalmologist.

## Results

**Fig 4:**
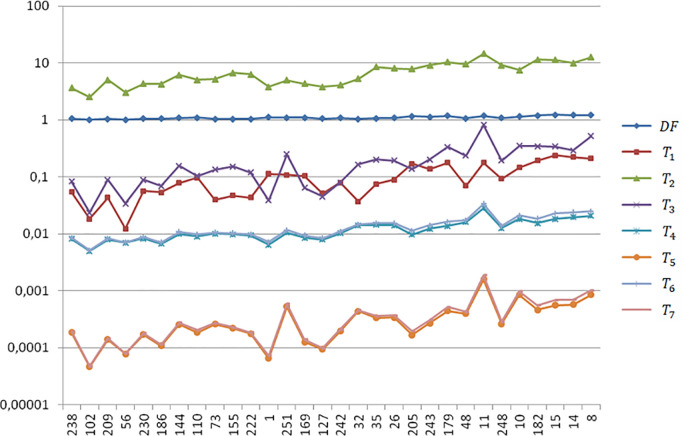
Local tortuosity $\tau \text{ = }\left\{DF,\;T_{\text{1}}\text{,}\ldots \text{,}T_{\text{7}}\right\}\;$ values of the entire (unpartitioned) vessel segments on a logarithmic scale. The abscissa shows the vessel numbers ordered with increasing tortuosity as ranked by the dataset ophthalmologist.

Fig 4 plots the local tortuosity of the entire (unpartitioned) vessel segments on a logarithmic scale, as measured by the local indices $\tau \text{ = }\left\{DF,\;T_{\text{1}}\text{,}\ldots \text{,}T_{\text{7}}\right\}$. The dataset number is presented on the abscissa axis of Fig 4, where the samples are arranged from left to right in ascending order of tortuosity, based on the dataset ophthalmologist’s ranking. The data displayed on Fig 4 are available in the supporting information (S1 Table).

Each local index $\tau_{i}$ ranks the vessel segments based on their tortuosity. The Spearman rank correlation coefficient $\rho \left(\tau_{i},\tau_{\text{j}}\right)$ between two indices, $\tau_{\text{i}}$ and $\tau_{\text{j}}$, equals 1 when they produce identical rankings. Lower correlation values indicate greater discrepancies in their ranking of vessel tortuosity. Therefore, the Spearman correlation coefficient serves as a measure of consistency between tortuosity indicators. Table 8 presents the Spearman correlation coefficients for all pairs of local tortuosity indices. For comparison, we have also included the correlation of each index with the dataset ophthalmologist’s ranking and the results reported by Grisan et al. [3].

**Table 8:** Spearman rank correlation coefficients $\rho \left(\tau_{i},\tau_{\text{j}}\right)$ between pairs of local indices (Fig 4) including ranking provided by the dataset ophthalmologist (Ophthalm) and the correlation reported by Grisan et al. [3].

	DF	$T_{\text{1}}$	$T_{\text{2}}$	$T_{\text{3}}$	$T_{\text{4}}$	$T_{\text{5}}$	$T_{\text{6}}$	$T_{\text{7}}$
$T_{\text{1}}$	1,000							
$T_{\text{2}}$	0,669	0,669						
$T_{\text{3}}$	0,682	0,682	0,908					
$T_{\text{4}}$	0,625	0,625	0,883	0,936				
$T_{\text{5}}$	0,617	0,617	0,815	0,962	0,957			
$T_{\text{6}}$	0,706	0,706	0,901	0,947	0,989	0,949		
$T_{\text{7}}$	0,644	0,644	0,834	0,969	0,963	0,996	0,961	
Ophthalm	0,770	0,770	0,842	0,804	0,863	0,770	0,895	0,790
Grisan [3]	0,792	0,792	0,922	0,925	0,919	0,917	0,939	0,928

All p-values < 0,0005

A trivial result from Table 8 is , that is, DF and $T_{\text{1}}$ rank the vessels in precisely the same order, as expected from the definitions of these indices (Table 2). With close definitions, $T_{\text{5}}$ and $T_{\text{7}}$ perform very similarly, $\rho \left(T_{\text{5}}\text{,}T_{\text{7}}\right)=\text{0,996}$. Note that the graphs corresponding to $T_{\text{5}}$ and $T_{\text{7}}$ in Fig 4 almost coincide. The same comment holds for $T_{\text{4}}$ and $T_{\text{6}}$, with $\rho \left(T_{\text{4}}\text{,}T_{\text{6}}\right)=\text{0,989}$ and close graphs in Fig 4. Despite the magnitude order, note the similarity between the plot profile of $T_{\text{3}}$ and those of $T_{\text{5}}$ and $T_{\text{7}}\;$ in Fig 4. This fact leads to a similar tortuosity ranking and high Spearman correlation coefficients: $\rho \left(T_{\text{3}}\text{,}T_{\text{5}}\right)=\text{0,962}$ and $\rho \left(T_{\text{3}}\text{,}T_{\text{7}}\right)=\text{0,969}$. All the correlations where significant (p < 0,0005) under the null hypothesis ($H_{0}:\;\rho =0$).

**Fig 5:**
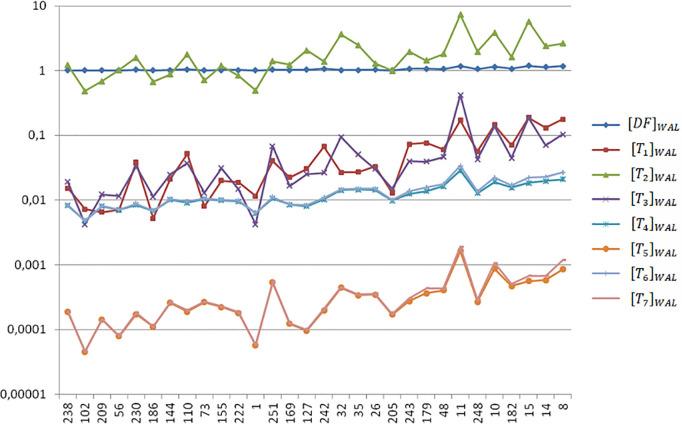
Global tortuosity computed with the WS of Weighted Additivity on Length (*WAL*) for the partitioned vessel segments on a logarithmic scale. The abscissa shows the vessel numbers ordered with increasing tortuosity as ranked by the dataset ophthalmologist.

With similar distribution to Fig 4, Fig 5 plots the global tortuosity of the same 30 artery vessels, calculated using the $\tau_{WAL}(C)$ WS (Table 5) on partitioned segments. The similar patterns observed in the graph pairs of ${\left[T_{\text{5}}\right]}_{WAL}$ and ${\left[T_{\text{7}}\right]}_{WAL}$, and ${\left[T_{\text{4}}\right]}_{WAL}$ and ${\left[T_{\text{6}}\right]}_{WAL}$, indicate a close agreement in their rankings of the 30 arteries. This is confirmed by the high Spearman rank correlation coefficients in Table 9: $\rho \left({\left[T_{\text{5}}\right]}_{WAL}\text{,}{\left[T_{\text{7}}\right]}_{WAL}\right)=\text{0,999}$ and $\rho \left({\left[T_{\text{4}}\right]}_{WAL}\text{,}{\left[T_{\text{6}}\right]}_{WAL}\right)=\text{0,996}$. The global tortuosity data displayed in Fig 5 are available in the supporting information (S1 Table).

**Table 9:** Spearman rank correlation coefficient between pairs of global tortuosity measures (Fig 5) for the *WAL* weighting scheme, including the dataset ophthalmologist’s ranking (Ophthalm).

	$[DF{]}_{WAL}$	${\left[T_{\text{1}}\right]}_{WAL}$	${\left[T_{\text{2}}\right]}_{WAL}$	${\left[T_{\text{3}}\right]}_{WAL}$	${\left[T_{\text{4}}\right]}_{WAL}$	${\left[T_{\text{5}}\right]}_{WAL}$	${\left[T_{\text{6}}\right]}_{WAL}$	${\left[T_{\text{7}}\right]}_{WAL}$
${\left[T_{\text{1}}\right]}_{WAL}$	1,000							
${\left[T_{\text{2}}\right]}_{WAL}$	0,843	0,843						
${\left[T_{\text{3}}\right]}_{WAL}$	0,876	0,876	0,929					
${\left[T_{\text{4}}\right]}_{WAL}$	0,823	0,823	0,782	0,891				
${\left[T_{\text{5}}\right]}_{WAL}$	0,809	0,809	0,755	0,914	0,961			
${\left[T_{\text{6}}\right]}_{WAL}$	0,852	0,852	0,786	0,893	0,996	0,959		
${\left[T_{\text{7}}\right]}_{WAL}$	0,813	0,813	0,755	0,913	0,959	0,999	0,958	
Ophthalm.	0,825	0,825	0,741	0,766	0,864	0,768	0,873	0,769

All p-values < 0,0005.

Table 10 compares the tortuosity rankings of two versions of the 30 artery dataset segments: one-section (unpartitioned) and partitioned, using the Spearman rank correlation coefficient $\rho \left({\left[\tau_{i}\right]}_{{WS}_{j}}\text{,}\tau_{\text{i}}\right)$. The ranking of one-section segments is based on their local tortuosity values $\tau_{i}$. The ranking of partitioned segments uses global measures calculated from a weighting scheme ${\text{WS}}_{j}$ applied to the local tortuosities (same $\tau_{i}$) of their constituent sections. Thus, for example, Table 10 shows a high Spearman rank correlation, $\rho \left({\left[T_{\text{4}}\right]}_{WAL}\text{,}T_{\text{4}}\right)=\text{0,999}$, between the ${\left[T_{\text{4}}\right]}_{WAL}$ global tortuosity measures and the one-section $T_{\text{4}}$ local tortuosity values for the 30 segments. This indicates a strong agreement in the rankings produced by these two approaches. The highest correlations (≥0.990) were observed with the WAL and WAC weighting schemes when used with the local tortuosity indices $T_{\text{4}}$, $T_{\text{5}}$, $T_{\text{6}}$, and $T_{\text{7}}$. In contrast, weighting schemes MTWA and TDG exhibited very low correlations, particularly when combined with the local indices DF and $T_{\text{2}}$.

**Table 10:** Spearman rank correlation coefficient $\rho \left({\left[\tau_{i}\right]}_{{WS}_{j}}\text{,}\tau_{\text{i}}\right)$ and p-value between global tortuosity of partitioned vessels and one-section (unpartitioned) tortuosity for the studied global WS and local indices.

	Local index (τ)							
Global weighting scheme (WS)	DF	$T_{\text{1}}$	$T_{\text{2}}$	$T_{\text{3}}$	$T_{\text{4}}$	$T_{\text{5}}$	$T_{\text{6}}$	$T_{\text{7}}$
$\tau_{MT}(C)$	0.798 (p < 0.0005)	0.798 (p < 0.0005)	0.633 (p < 0.0005)	0.892 (p < 0.0005)	0.976 (p < 0.0005)	0.948 (p < 0.0005)	0.981 (p < 0.0005)	0.961 (p < 0.0005)
$\tau_{WAL}(C)$	0.761 (p < 0.0005)	0.761 (p < 0.0005)	0.622 (p < 0.0005)	0.871 (p < 0.0005)	0.999 (p < 0.0005)	0.999 (p < 0.0005)	0.991 (p < 0.0005)	0.996 (p < 0.0005)
$\tau_{MTWA}(C)$	0.216 (p = 0.251)	0.616 (p < 0.0005)	0.305 (p = 0.102)	0.635 (p < 0.0005)	0.702 (p < 0.0005)	0.867 (p < 0.0005)	0.713 (p < 0.0005)	0.875 (p < 0.0005)
$\tau_{WAC}(C)$	0.758 (p < 0.0005)	0.758 (p < 0.0005)	0.606 (p < 0.0005)	0.859 (p < 0.0005)	0.998 (p < 0.0005)	0.993 (p < 0.0005)	0.990 (p < 0.0005)	0.995 (p < 0.0005)
$\tau_{TDG}(C)$	-0.164 (p = 0.387)	0.738 (p < 0.0005)	0.396 (p = 0.030)	0.752 (p < 0.0005)	0.865 (p < 0.0005)	0.919 (p < 0.0005)	0.839 (p < 0.0005)	0.911 (p < 0.0005)

Table 11 compares the global tortuosity-based rank order of the partitioned 30 artery dataset segments with the ranking provided by the dataset ophthalmologist. The highest correlations (≥0.860) were observed when using the WAL and WAC weighting schemes with the local tortuosity indices $T_{\text{4}}$ and $T_{\text{6}}$. The MT weighting scheme also exhibited good correlations with these same indices $T_{\text{4}}$ (0.847) and $T_{\text{6}}$ (0.853). In contrast, the MTWA and TDG weighting schemes again demonstrated very low or non-significant correlations, particularly when combined with the local indices DF and $T_{\text{2}}$.

**Table 11:** Spearman rank correlation coefficient $\rho \left({\left[\tau_{i}\right]}_{{WS}_{j}}\text{,ophthalm}\right)$ and p-value between global tortuosity of partitioned vessels and dataset ophthalmologist. Global tortuosities are calculated for studied WS and local indices.

	Local index (τ)							
Global weighting scheme (WS)	DF	$T_{\text{1}}$	$T_{\text{2}}$	$T_{\text{3}}$	$T_{\text{4}}$	$T_{\text{5}}$	$T_{\text{6}}$	$T_{\text{7}}$
$\tau_{MT}(C)$	0.833 (p < 0.0005)	0.833 (p < 0.0005)	0.752 (p < 0.0005)	0.750 (p < 0.0005)	0.847 (p < 0.0005)	0.692 (p < 0.0005)	0.853 (p < 0.0005)	0.722 (p < 0.0005)
$\tau_{WAL}(C)$	0.825 (p < 0.0005)	0.825 (p < 0.0005)	0.741 (p < 0.0005)	0.766 (p < 0.0005)	0.864 (p < 0.0005)	0.768 (p < 0.0005)	0.873 (p < 0.0005)	0.769 (p < 0.0005)
$\tau_{MTWA}(C)$	0.222 (p = 0.238)	0.674 (p < 0.0005)	0.504 (p = 0.005)	0.586 (p = 0.001)	0.570 (p = 0.001)	0.638 (p < 0.0005)	0.600 (p < 0.0005)	0.662 (p < 0.0005)
$\tau_{WAC}(C)$	0.824 (p < 0.0005)	0.824 (p < 0.0005)	0.734 (p < 0.0005)	0.747 (p < 0.0005)	0.863 (p < 0.0005)	0.761 (p < 0.0005)	0.872 (p < 0.0005)	0.768 (p < 0.0005)
$\tau_{TDG}(C)$	-0.093 (p = 0.626)	0.782 (p < 0.0005)	0.570 (p = 0.001)	0.683 (p < 0.0005)	0.660 (p < 0.0005)	0.615 (p < 0.0005)	0.671 (p < 0.0005)	0.624 (p < 0.0005)

In Table 10, we used the same local index to quantify both the tortuosity of the one-section (unpartitioned) vessel and the local tortuosity of its composing sections when deriving the segment’s global tortuosity. However, it is important to note that using the same local index for both measures is not necessary. In fact, cross-combinations of local indices can also provide valuable insights. Table 12 presents the Spearman correlation coefficient $\rho ({\left[T_{4}\right]}_{WAL}\text{,}\tau_{\text{i}}\backslash )$, which compares the global tortuosity-based ranking of partitioned segments (using WAL weighting scheme with local index $T_{4}$) with the DF, $T_{\text{1}}$, …, $T_{\text{7}}$ local tortuosity-based rankings of one-section (unpartitioned) segments. The results in Table 12 indicate that the ${\left[T_{\text{4}}\right]}_{WAL}$ global measure yields similar rankings independently of whether the one-section vessel’s tortuosity is assessed using $T_{\text{4}}$ (0.999) or $T_{\text{6}}$(0.988).

**Table 12:** Spearman rank correlation coefficient $\rho ({\left[T_{4}\right]}_{WAL}\text{,}\tau_{\text{i}}\backslash )$ and p-value for global tortuosity based on ${\left[T_{4}\right]}_{WAL}$ and $\tau_{\text{i}}$ local indices applied to the one-section (unpartitioned) segments.

	Local index (τ)							
	DF	$T_{\text{1}}$	$T_{\text{2}}$	$T_{\text{3}}$	$T_{\text{4}}$	$T_{\text{5}}$	$T_{\text{6}}$	$T_{\text{7}}$
${\left[T_{\text{4}}\right]}_{WAL}$	0,618	0,618	0,879	0,937	0,999	0,959	0,988	0,963

All p-values < 0,0005

## Discussion and conclusions

A significant challenge in utilizing tortuosity measures for retinal vessel assessment in clinical studies lies in the absence of standardized criteria for their definition, evaluation, and comparison [17]. While various studies, beginning with Lotmar et al. [18] and followed by Hart et al. [2], Grisan et al. [3], Kalitzeos et al. [12], and others, have explored the medical intuitions of tortuosity and formulated abstract properties through a range of local tortuosity indices, these properties can lead to conflicting results [3], complicating the index selection and comparative analysis. This is even more challenging when assessing the tortuosity of a vascular tree, which is a complex network of connected vessels with branches and possible entanglements that requires a transition from local to global tortuosity measurements through appropriate WS [2–4,6]. This need for a global tortuosity scheme arises even at the level of individual segments composed of smoothly connected vessel sections. Typically, the evaluation of local and global tortuosity indices has relied on comparing their numerical outputs to subjective assessments provided by one or more ophthalmologists for a given set of retinal vessel images. However, the variability inherent in both the retinal vessel datasets (many of which are not publicly accessible) and the ophthalmologist assessments (ground-truth) significantly hinders meaningful comparisons across different studies.

This study contributes to a deeper understanding of the performance of eight widely used local tortuosity indices, $\tau =\left\{DF,\;T_{1},..,T_{7}\right\}$, and five global tortuosity weighting schemes $WS=\left\{\tau_{MT},\;\tau_{WAL},\tau_{MTWA},\tau_{WAC},\tau_{TDG}\right\}$. Our analysis leverages the framework of vessel compositionality, introduced by Hart et al. [2], which focuses on a single unbranched vessel segment and compares its local tortuosity to the combined tortuosities of its constituent, non-overlapping sections. We began by examining two opposite conceptualizations of vessel tortuosity: Hart’s and Grisan’s. From a curvature perspective, Hart’s composition property aligns more closely with the concept of mean curvature, while Grisan’s property aligns more closely with the idea of total or accumulated curvature. We applied these criteria, mathematically formulated as inequalities, to the eight local indices. Our analysis revealed that $T_{\text{2}}$ and $T_{\text{3}}$ satisfy Grisan’s inequality, while $T_{\text{4}}$ and $T_{\text{5}}$ fulfill Hart’s inequality for any vessel segment. However, we demonstrated through an example that the remaining indices ($DF,\;T_{\text{1}},\;T_{\text{6}}$, and $T_{\text{7}}$) do not consistently satisfy either inequality, with their behavior depending on the specific vessel trace.

Global tortuosity measures are influenced by three key factors: vessel partition into sections, local index used to quantify the tortuosity of individual sections, and weighting scheme. To evaluate the influence of these factors, we compared the tortuosity rankings of the 30 artery vessel segments from the BioImLab RET-TORT dataset across different conditions. Spearman rank correlation and its associated p-value were used to objectively assess the performance of the studied local indices and global tortuosity weighting schemes.

Our analysis revealed that most global tortuosity measures are sensitive to vessel partitioning. Notably, only the ${\left[T_{4}\right]}_{WAL}$ and ${\left[T_{5}\right]}_{WAL}$ combinations remained unaffected by changes in the partition, consistent with their definitions. Generally, for most other ${\left[\tau_{i}\right]}_{{WS}_{j}}$ combinations, increasing the number of sections (resulting in shorter section lengths) typically leads to a decrease in the calculated global tortuosity value. Therefore, the need for a standardized partitioning protocol is highlighted, particularly when using global tortuosity measures sensitive to this factor.

For one-section (unpartitioned) vessel segments, the local indices were applied to the entire segment to determine their tortuosity. Spearman rank correlation $\rho \left(\tau_{i},\tau_{\text{j}}\right)$ analysis revealed a strong agreement between indices $T_{\text{5}}$ and $T_{\text{7}}$ ($\rho \left(T_{5},T_{\text{7}}\right)=0,996$), indicating that they produced highly similar vessel rankings. These indices also showed strong agreement with $T_{\text{3}}$ ($\rho \left(T_{3},T_{\text{5}}\right)=0,962\;and\rho \left(T_{3},T_{\text{7}}\right)=0,969$). Similarly, indices $T_{\text{4}}$ and $T_{\text{6}}$ exhibited a very high correlation ($\rho \left(T_{4},T_{\text{6}}\right)=0,989$). Furthermore, $T_{\text{4}}$ and $T_{\text{6}}$ showed the closest agreement with the dataset ophthalmologist ($\rho \left(T_{4},\;\text{ophthalm}\right)=0,863$ and $\rho \left(T_{6},\;\text{ophthalm}\right)=0,895$). This finding aligns well with the results by Grisan et al. [3], who also reported the highest correlation for $T_{\text{6}}$ with the ophthalmologist’s assessments ($\rho_{\text{Grisan}}\left(T_{6},\;\text{ophthalm}\right)=0,939$).

Vessel segments were partitioned into sections based on bifurcation points, crossing points, and endpoints, a common approach in related research (e.g., [4]). Global tortuosity measures were then derived by combining a local tortuosity index ($\tau =\left\{DF,\;T_{1},..,T_{7}\right\}$) with a weighting scheme ($WS=\left\{\tau_{MT},\;\tau_{WAL},\tau_{MTWA},\tau_{WAC},\tau_{TDG}\right\}$) [4]. This study investigated the consistency of vessel segment rankings obtained under different conditions.

One condition involved evaluating the WAL weighting scheme with different local tortuosity indices. Spearman rank correlation $\rho \left({\left[\tau_{i}\right]}_{WAL},{\left[\tau_{j}\right]}_{WAL}\right),\;\text{with}\;i\neq j$, revealed high correlations between the following pairs of local indices: $T_{5\;}and\;T_{7\;}$ ($\rho \left({\left[T_{5}\right]}_{WAL},{\left[T_{7}\right]}_{WAL}\right)=0,999$), and $T_{4\;}\text{and}\;T_{6\;}$ ($\rho \left({\left[T_{4}\right]}_{WAL},{\left[T_{6}\right]}_{WAL}\right)=0,996$) (Table 9).

In a second condition, we compared the tortuosity rankings obtained from local indices applied to one-section (unpartitioned) vessel segments with those rankings derived from global weighting schemes (using the same local index) applied to the partitioned segments. Spearman rank correlation coefficients $\rho \left({\left[\tau_{i}\right]}_{{WS}_{j}}\text{,}\tau_{\text{i}}\right)$ were calculated to assess these comparisons. The WAL weighting scheme demonstrated the highest correlations with local indices $T_{\text{4}}$ and $T_{\text{5}}$ ($\rho \left({\left[T_{4}\right]}_{WAL}\text{,}T_{\text{4}}\right)=\rho \left({\left[T_{5}\right]}_{WAL}\text{,}T_{\text{5}}\right)=0.999$) (Table 10). WAL and WAC schemes also exhibited very high correlations (≥0.990) with local indices $T_{\text{4}}$, $T_{\text{5}}$, $T_{\text{6}}$ and $T_{\text{7}}$. In contrast, the MTWA and TDG weighting schemes showed significantly lower correlations, particularly when combined with local indices DF and $T_{\text{2}}$ (Table 10). To further analyze these results, we compared the rankings obtained from the global weighting schemes applied to partitioned segments with the rankings provided by the dataset ophthalmologist. The highest Spearman rank correlations ($\rho \left({\left[\tau_{i}\right]}_{{WS}_{j}}\text{,ophthalm}\right)\geq 0.860$) (Table 11) were again observed with the WAL and WAC schemes and local indices $T_{\text{4}}$ and $T_{\text{6}}$ ($\rho \left({\left[T_{6}\right]}_{WAL}\text{,ophthalm}\right)=0.873$; $\rho \left({\left[T_{6}\right]}_{WAC}\text{,ophthalm}\right)=0.872$; $\rho \left({\left[T_{4}\right]}_{WAL}\text{,ophthalm}\right)=0.864,\;\rho \left({\left[T_{4}\right]}_{WAC},\;ophthalm\right)=0.863$). The MT weighting scheme also showed good correlations with local indices $T_{\text{4}}$ ($\rho \left({\left[T_{4}\right]}_{MT}\text{,ophthalm}\right)=0.847$) and $T_{\text{6}}$ ($\rho \left({\left[T_{6}\right]}_{MT}\text{,ophthalm}\right)=0.853$). Conversely, the MTWA and TDG weighting schemes consistently indicated low correlations, particularly when combined with local indices DF and $T_{\text{2}}$.

In a third condition, we explored cross-combinations of local indices and weighting schemes. Specifically, we analyzed the performance of the global ${\left[T_{4}\right]}_{WAL}$ measures (derived from partitioned segments) in comparison with different local indices $\tau =\left\{DF,\;T_{1},..,T_{7}\right\}$ applied to one-section (unpartitioned) segments. Spearman rank correlation values ($\rho \left({\left[T_{4}\right]}_{WAL}\text{,}\tau_{i}\right),\;i\neq 4$) revealed that the ${\left[T_{\text{4}}\right]}_{WAL}$ measure exhibited a strong similarity to the local index $T_{\text{6}}\;$ among the other local indices applied to one-section segments ($\begin{array}{c} \rho \left({\left[T_{4}\right]}_{WAL}\text{,}T_{\text{6}}\right)=0.988 \end{array}$) (Table 12).

Our findings suggest that the global weighting schemes WAL and WAC, particularly when used in conjunction with local indices $T_{\text{4}}$ and $T_{\text{6}}$, consistently produce reliable tortuosity rankings. This objective conclusion is further supported by comparisons with the ranking provided by the dataset ophthalmologist. These results align well with previous research. Hart et al. [2] found that indices $T_{\text{3}}$ and $T_{\text{4}}$ most closely aligned with ophthalmologists’ perceptions of tortuosity, although $T_{\text{3}}$ exhibited slightly better performance in classification tasks. Grisan et al. [3] highlighted $T_{\text{6}}$ as the most resilient to sparse and noisy vessel descriptions. Handayani et al. [4] identified $T_{\text{4}}$ as the most suitable local index for use with the WAL weighting scheme, achieving a mean Spearman rank correlation of 0.96 with the assessments of five ophthalmologists. We recall that these three studies [2–4] neither shared the dataset images nor the ophthalmologists’ assessments of ground-truths. In our study, we have used the set of artery segments of RET-TORT dataset [3] on which we applied a segmentation procedure designed in our prior works [13,14,16].

In addition to these three conditions, we analyzed two more factors in prior studies on local tortuosity indices: scaling [13] and image frame center [14]. While local indices $T_{\text{4}}$ and $T_{\text{6}}$ are sensitive to scaling [2,13], their normalized ratios (relative to arc length (L) or chord length (D)) contribute to the robustness of the derived global tortuosity weighting schemes, as noted by Handayani et al. [4]. Finally, in [14], we found that local index $T_{\text{4}}$ exhibited the highest robustness to changes in frame center (macula to optic disc), followed closely by $T_{\text{6}}$ (Spearman rank correlation of 0.995).

Tortuosity measures can be affected by a variety of image acquisition and analysis factors, potentially acting as confounders. These include ocular scattering, nonuniform illumination, retinal camera characteristics, image compression algorithms, and processing tools such as segmentation, skeletonization, and smoothing, all of which could introduce bias in metric calculations. To enhance methodological robustness, adjust for confounders, and achieve stronger statistical validation for reliable clinical implementation, further investigation using publicly accessible and large datasets is necessary.

In this line of progress, our study contributes a novel, objective method for evaluating local and global tortuosity indices, offering an alternative to the subjective assessments commonly employed in previous research. This operational framework can contribute to the standardization of criteria for comparing local and global tortuosity indices. While this work provides valuable insights on the performance of eight local indices and five global weighting schemes, it has certain limitations. Our analysis focused on vessel segments rather than the entire retinal vascular tree. Moreover, the proposed objective evaluation method cannot directly compare tortuosity values calculated with local and global indices across the vessels of vascular trees. Despite these limitations, our analysis of tortuosity concepts and index performance can be highly valuable for emerging techniques based on deep learning and artificial intelligence. While these techniques often rely on extensive datasets with annotations from expert teams, they demonstrate significant potential for integrating information from various sources: tortuosity indices, anatomical factors [6,7], and other relevant data not featured in standard fundus images. This integration can ultimately contribute to improved diagnostic accuracy and risk stratification in various diseases.

## Supporting information

S1 Appendix Intermediate results obtained from the application of the procedure (vessel segment parametrization and partition into sections) to the 30 arteries in the RET-TORT dataset. Figures analogous to those shown in Figs. 3d, 3f, 3h, 3i, and 3j, are included.
(ZIP)

S1 Table Numerical data of plots included in Fig 4 and Fig 5. (XLSX)
