# Supplementary figures and images for: Bridging local and global tortuosity of retinal vessels: Objective testing of index performance (part 2 of 2)
Source: PLoS One. 2025 Aug 7;20(8):e0329379. doi: 10.1371/journal.pone.0329379 (PMC12331121; doi:10.1371/journal.pone.0329379)

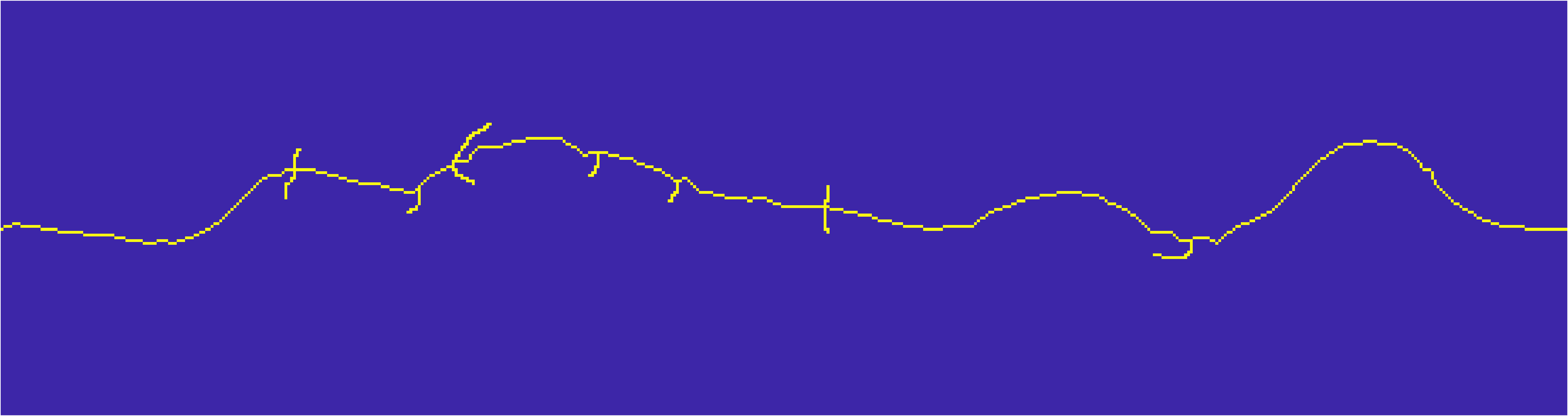

Supplement: S1 Appendix — Figures analogous to those shown in Figs. 3d, 3f, 3h, 3i, and 3j, are included. (ZIP) [file pone.0329379.s001.zip › S1 Appendix/048_Artery/f_Skeleton_048.tif]

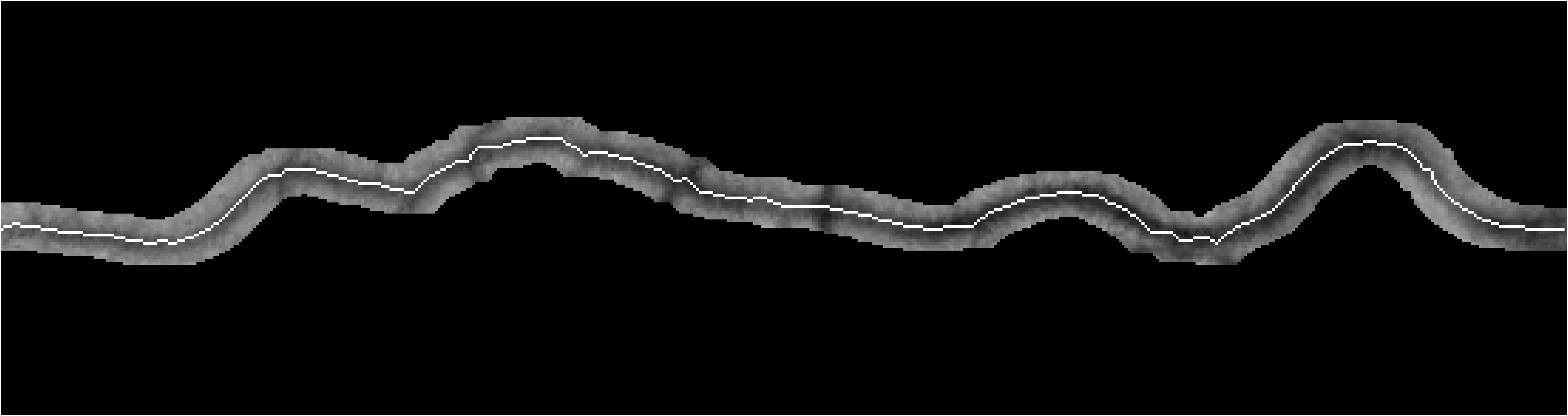

Supplement: S1 Appendix — Figures analogous to those shown in Figs. 3d, 3f, 3h, 3i, and 3j, are included. (ZIP) [file pone.0329379.s001.zip › S1 Appendix/048_Artery/d_ROI with manual trace_048.tif]

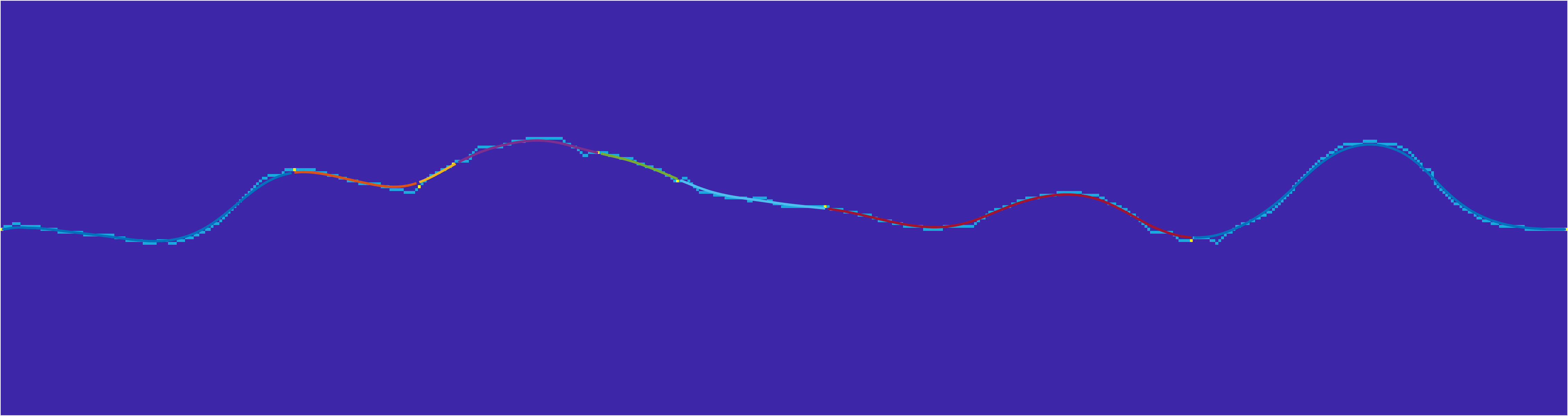

Supplement: S1 Appendix — Figures analogous to those shown in Figs. 3d, 3f, 3h, 3i, and 3j, are included. (ZIP) [file pone.0329379.s001.zip › S1 Appendix/048_Artery/j_partition_048.tif]

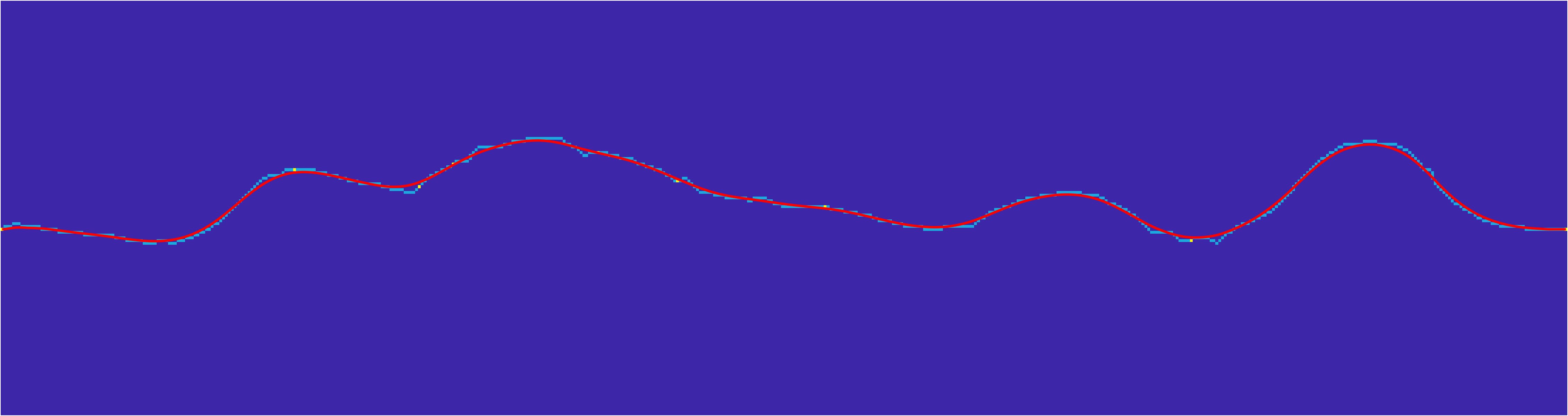

Supplement: S1 Appendix — Figures analogous to those shown in Figs. 3d, 3f, 3h, 3i, and 3j, are included. (ZIP) [file pone.0329379.s001.zip › S1 Appendix/048_Artery/i_smoothed segment_048.tif]

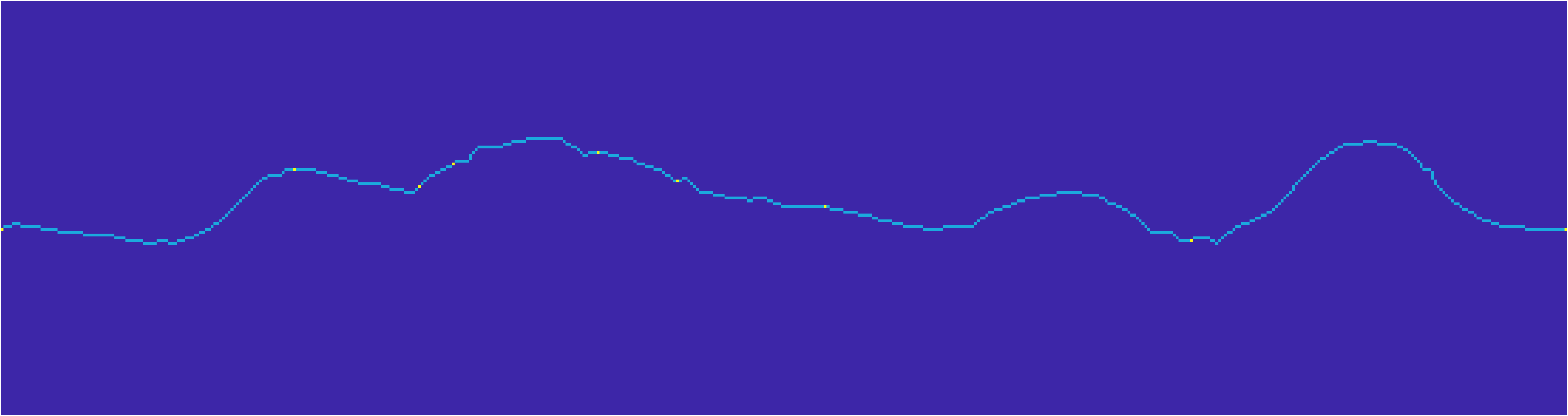

Supplement: S1 Appendix — Figures analogous to those shown in Figs. 3d, 3f, 3h, 3i, and 3j, are included. (ZIP) [file pone.0329379.s001.zip › S1 Appendix/048_Artery/h_centerline and division points_048.tif]

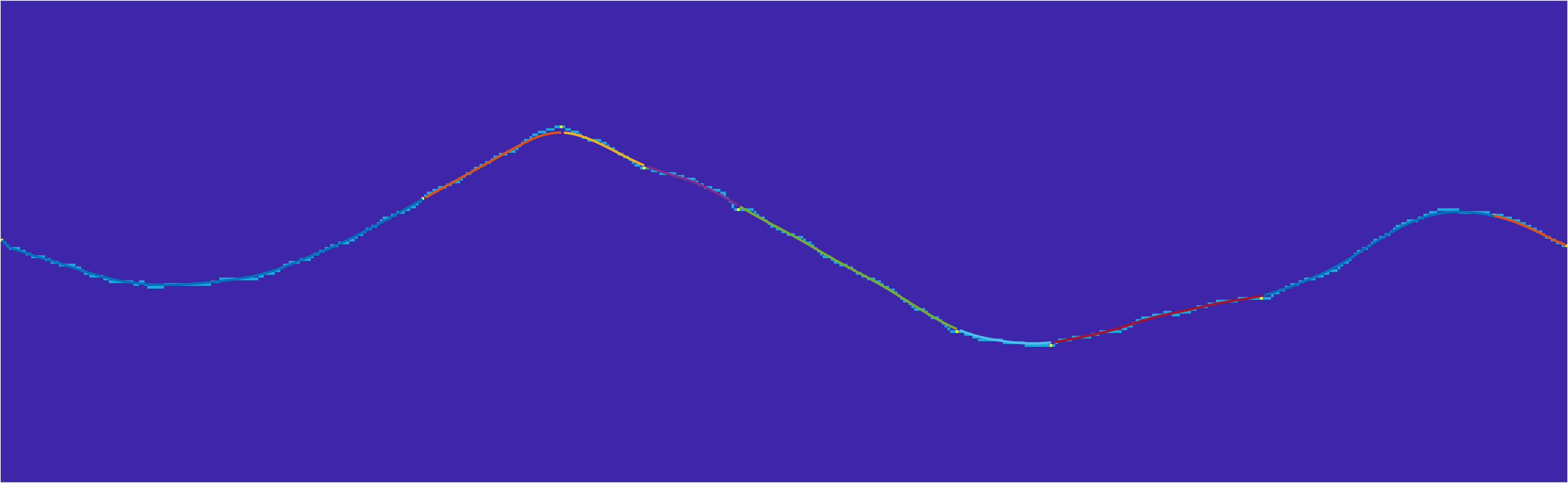

Supplement: S1 Appendix — Figures analogous to those shown in Figs. 3d, 3f, 3h, 3i, and 3j, are included. (ZIP) [file pone.0329379.s001.zip › S1 Appendix/144_Artery/j_partition_144.tif]

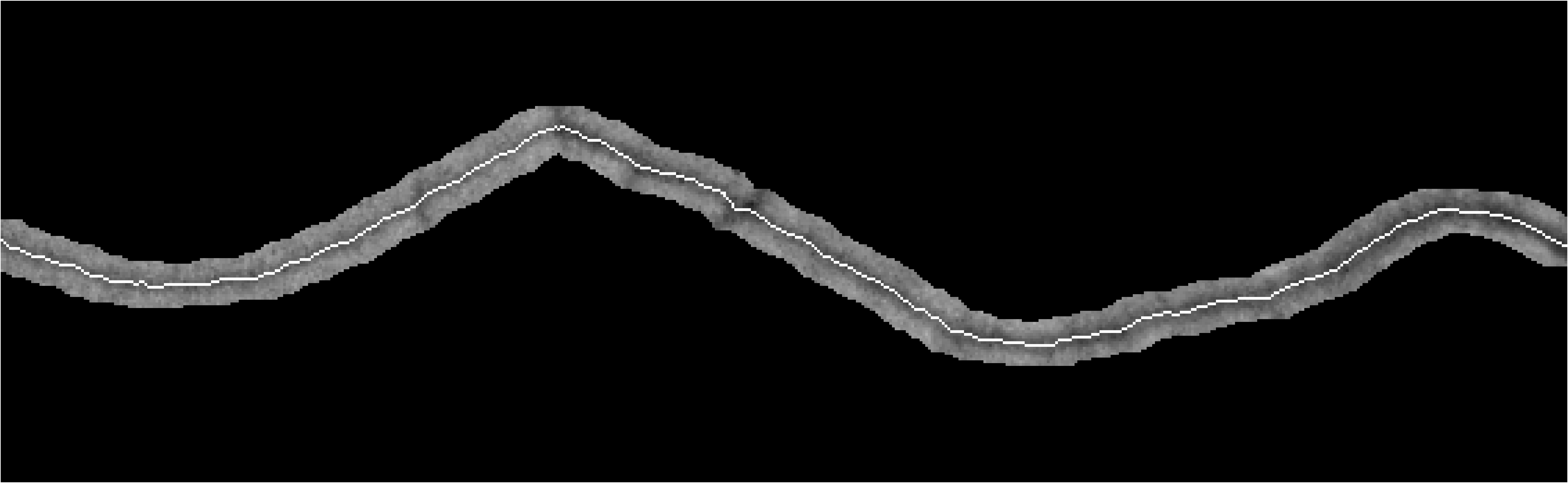

Supplement: S1 Appendix — Figures analogous to those shown in Figs. 3d, 3f, 3h, 3i, and 3j, are included. (ZIP) [file pone.0329379.s001.zip › S1 Appendix/144_Artery/d_ROI with manual trace_144.tif]

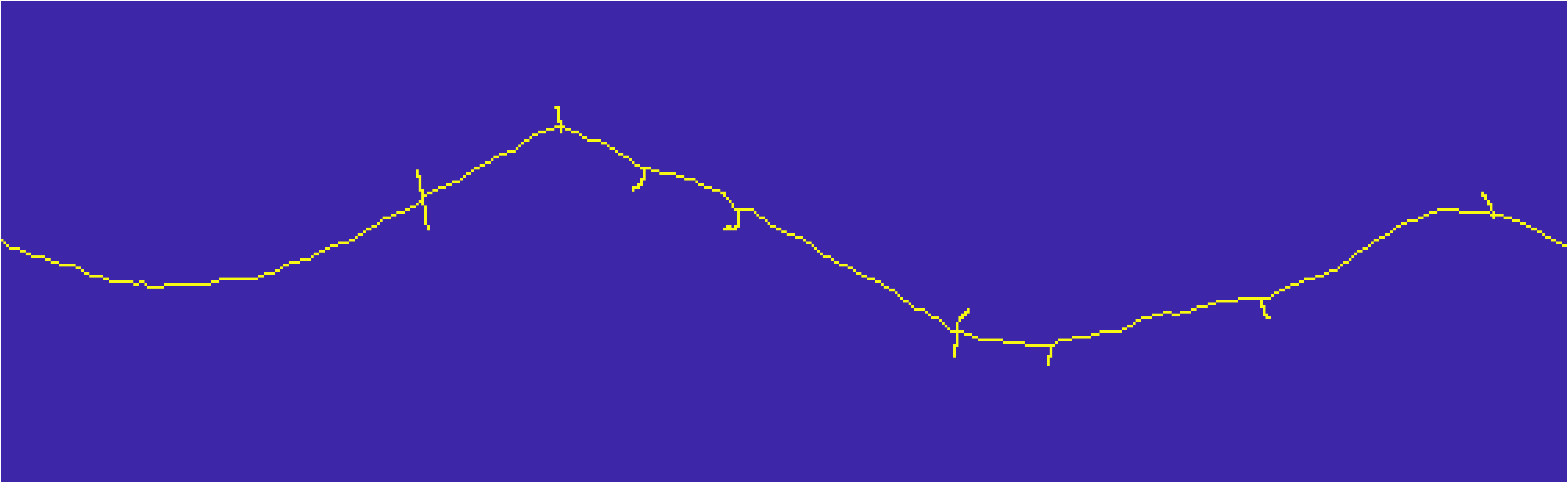

Supplement: S1 Appendix — Figures analogous to those shown in Figs. 3d, 3f, 3h, 3i, and 3j, are included. (ZIP) [file pone.0329379.s001.zip › S1 Appendix/144_Artery/f_Skeleton_144.tif]

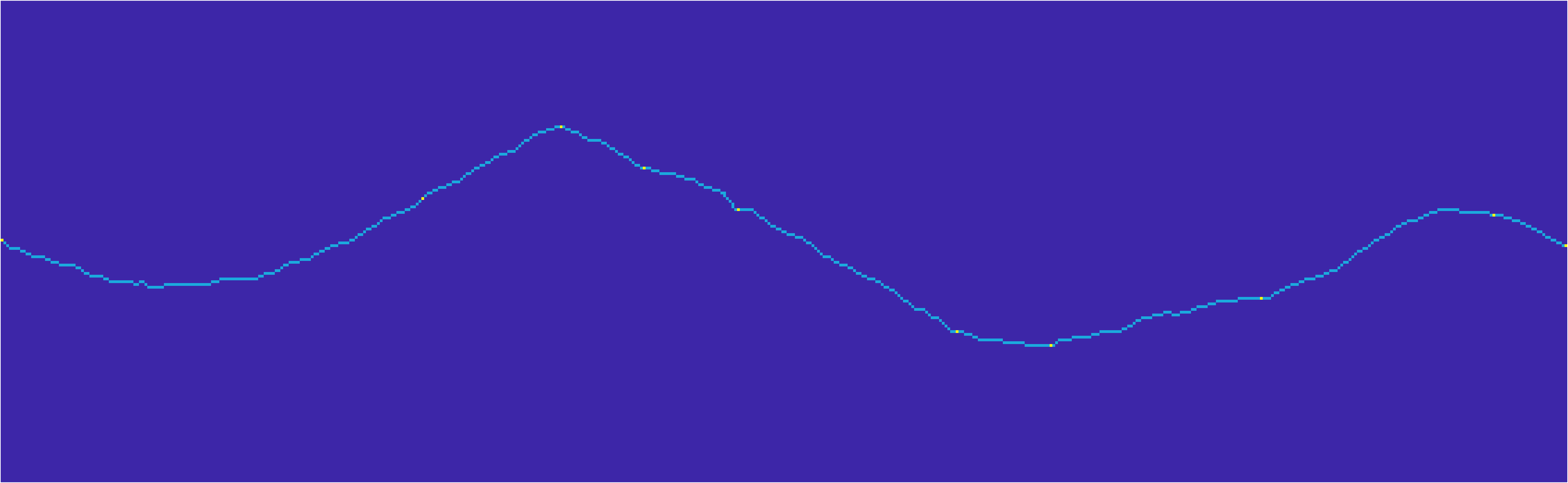

Supplement: S1 Appendix — Figures analogous to those shown in Figs. 3d, 3f, 3h, 3i, and 3j, are included. (ZIP) [file pone.0329379.s001.zip › S1 Appendix/144_Artery/h_centerline and division points_144.tif]

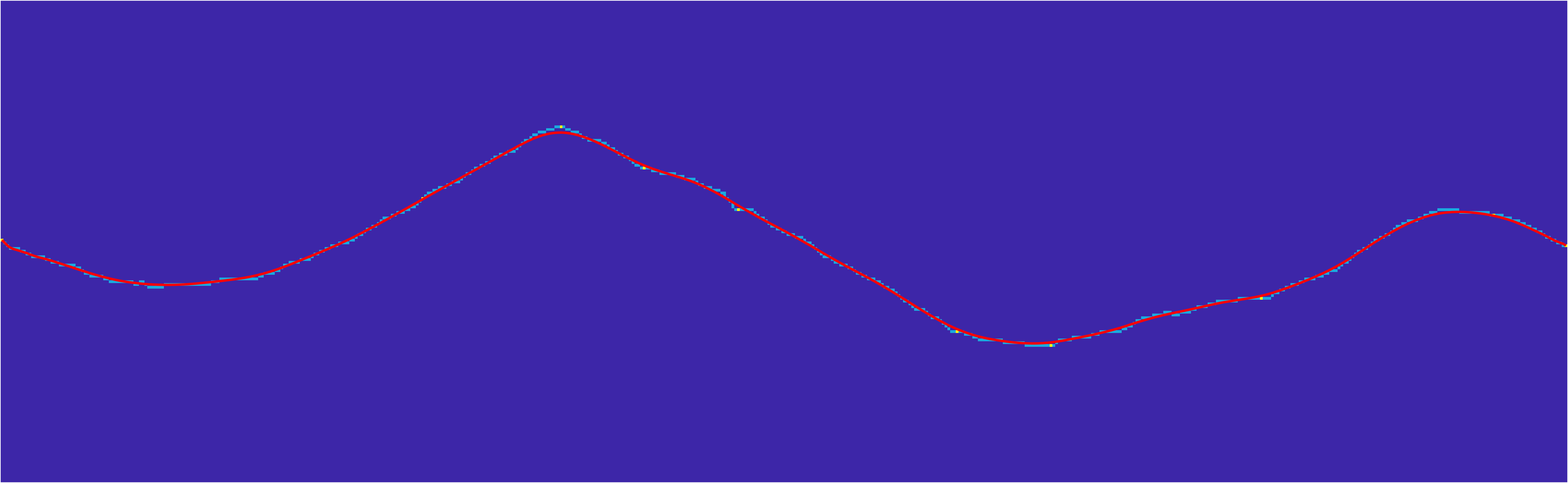

Supplement: S1 Appendix — Figures analogous to those shown in Figs. 3d, 3f, 3h, 3i, and 3j, are included. (ZIP) [file pone.0329379.s001.zip › S1 Appendix/144_Artery/i_smoothed segment_144.tif]

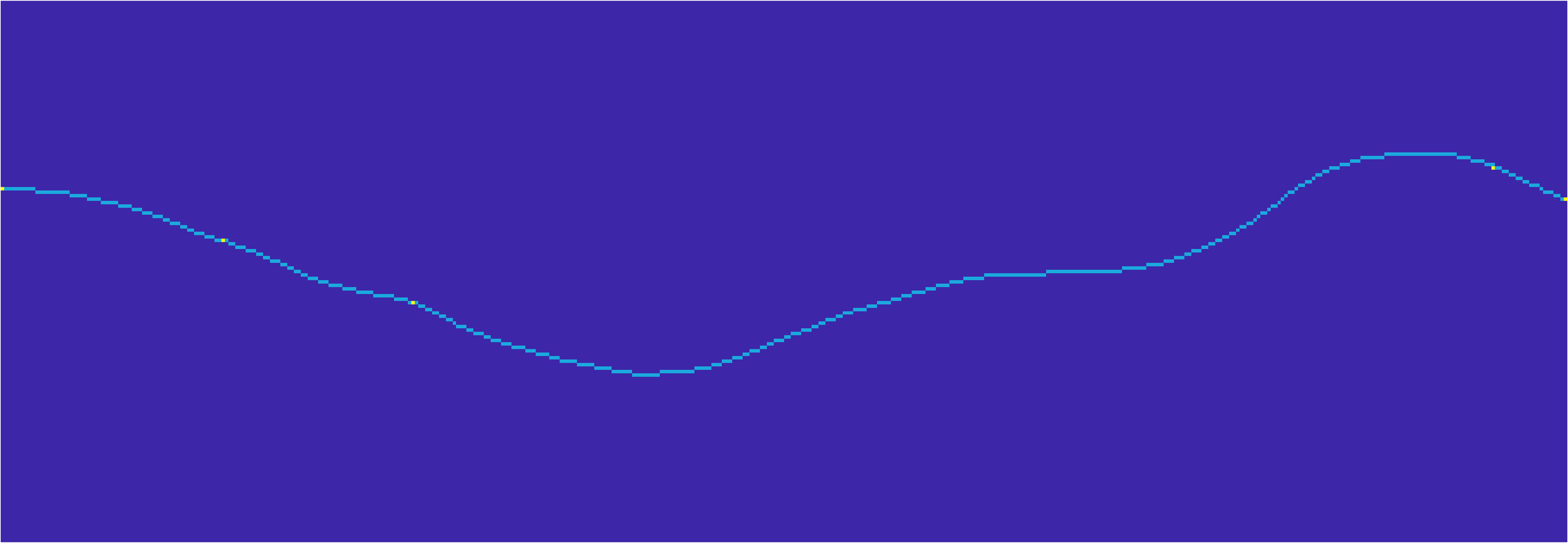

Supplement: S1 Appendix — Figures analogous to those shown in Figs. 3d, 3f, 3h, 3i, and 3j, are included. (ZIP) [file pone.0329379.s001.zip › S1 Appendix/127_Artery/h_centerline and division points_127.tif]

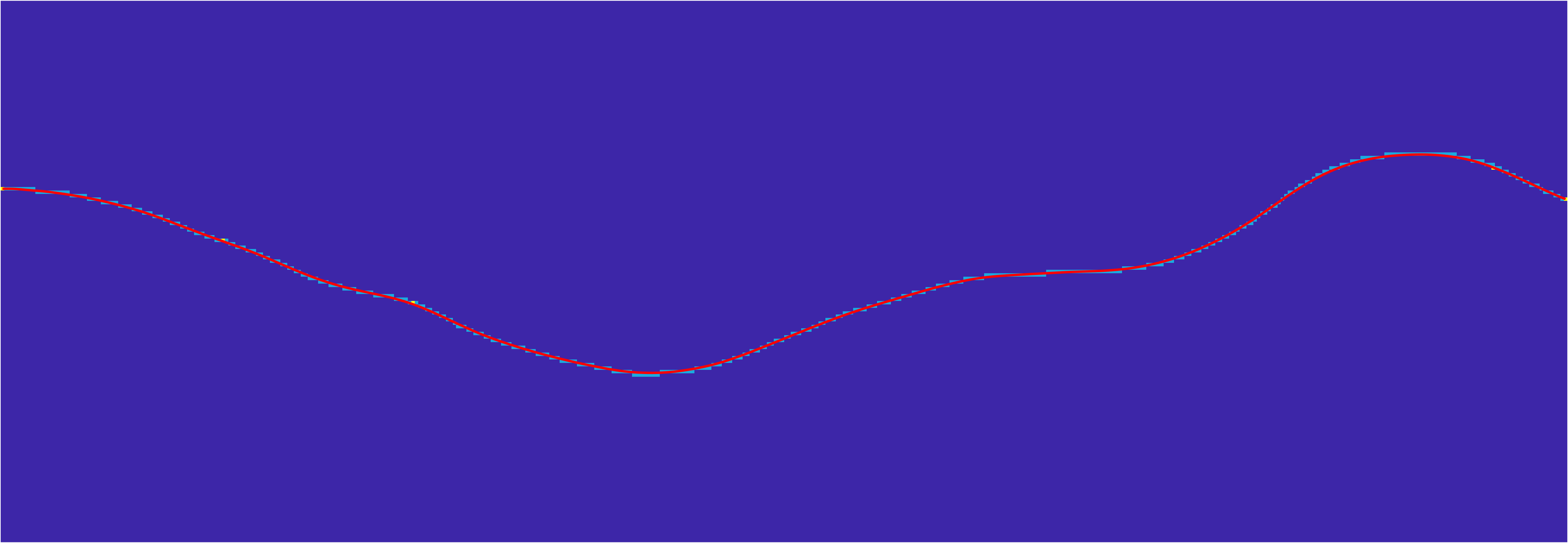

Supplement: S1 Appendix — Figures analogous to those shown in Figs. 3d, 3f, 3h, 3i, and 3j, are included. (ZIP) [file pone.0329379.s001.zip › S1 Appendix/127_Artery/i_smoothed segment_127.tif]

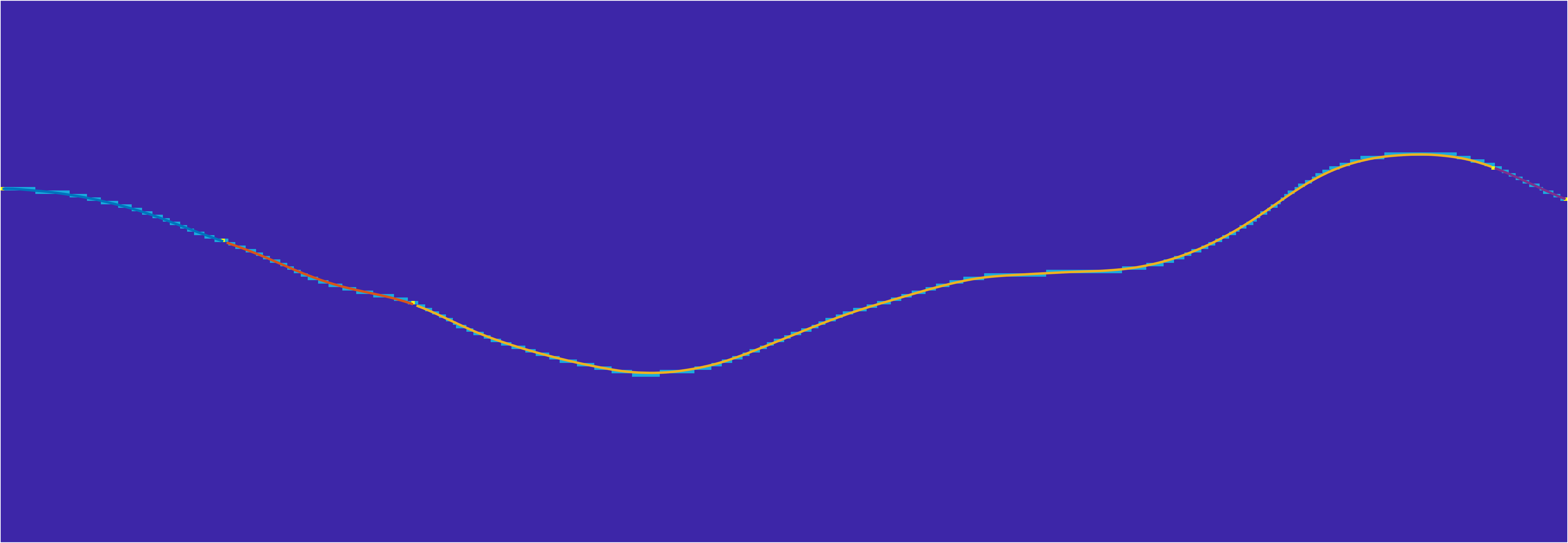

Supplement: S1 Appendix — Figures analogous to those shown in Figs. 3d, 3f, 3h, 3i, and 3j, are included. (ZIP) [file pone.0329379.s001.zip › S1 Appendix/127_Artery/j_partition_127.tif]

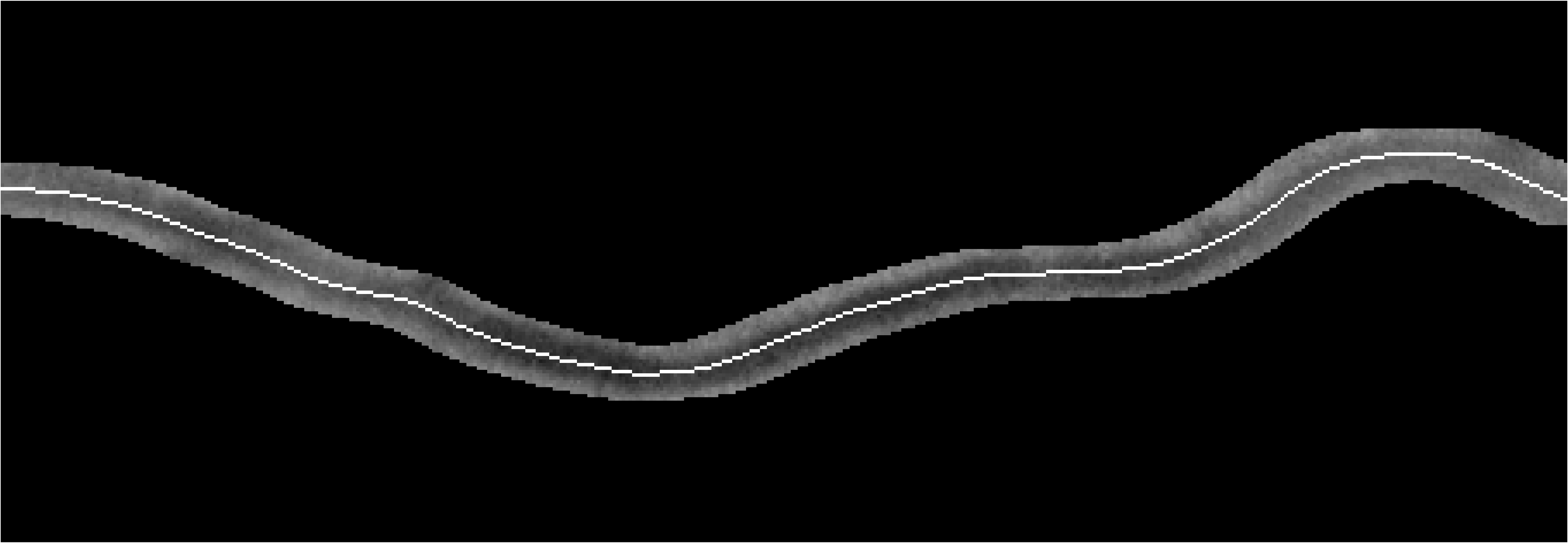

Supplement: S1 Appendix — Figures analogous to those shown in Figs. 3d, 3f, 3h, 3i, and 3j, are included. (ZIP) [file pone.0329379.s001.zip › S1 Appendix/127_Artery/d_ROI with manual trace_127.tif]

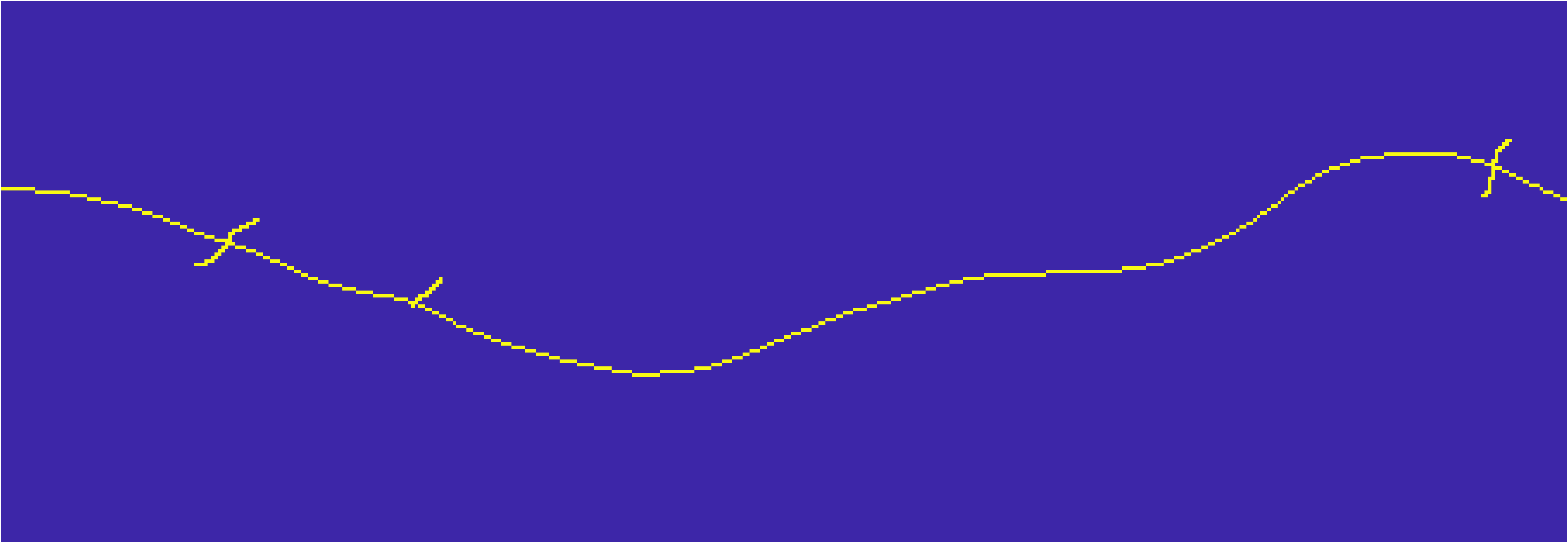

Supplement: S1 Appendix — Figures analogous to those shown in Figs. 3d, 3f, 3h, 3i, and 3j, are included. (ZIP) [file pone.0329379.s001.zip › S1 Appendix/127_Artery/f_Skeleton_127.tif]

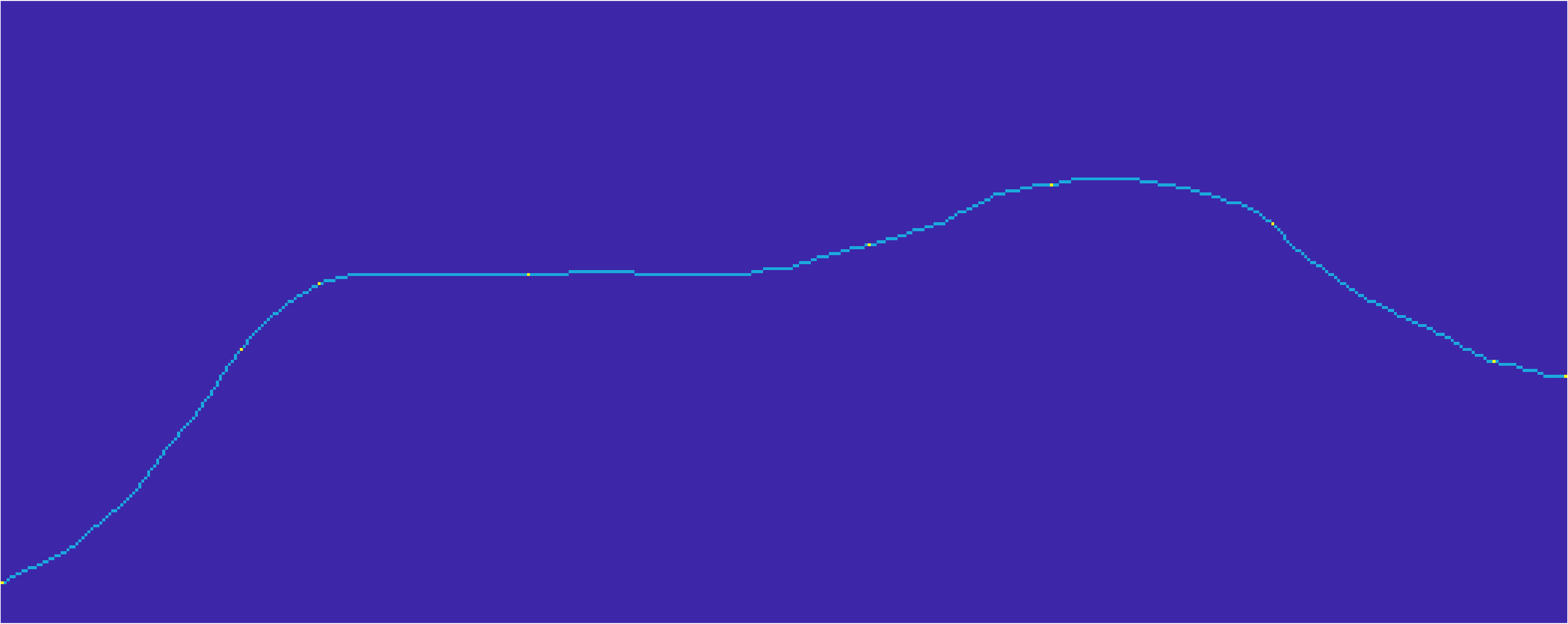

Supplement: S1 Appendix — Figures analogous to those shown in Figs. 3d, 3f, 3h, 3i, and 3j, are included. (ZIP) [file pone.0329379.s001.zip › S1 Appendix/001_Artery/h_centerline and division points_001.tif]

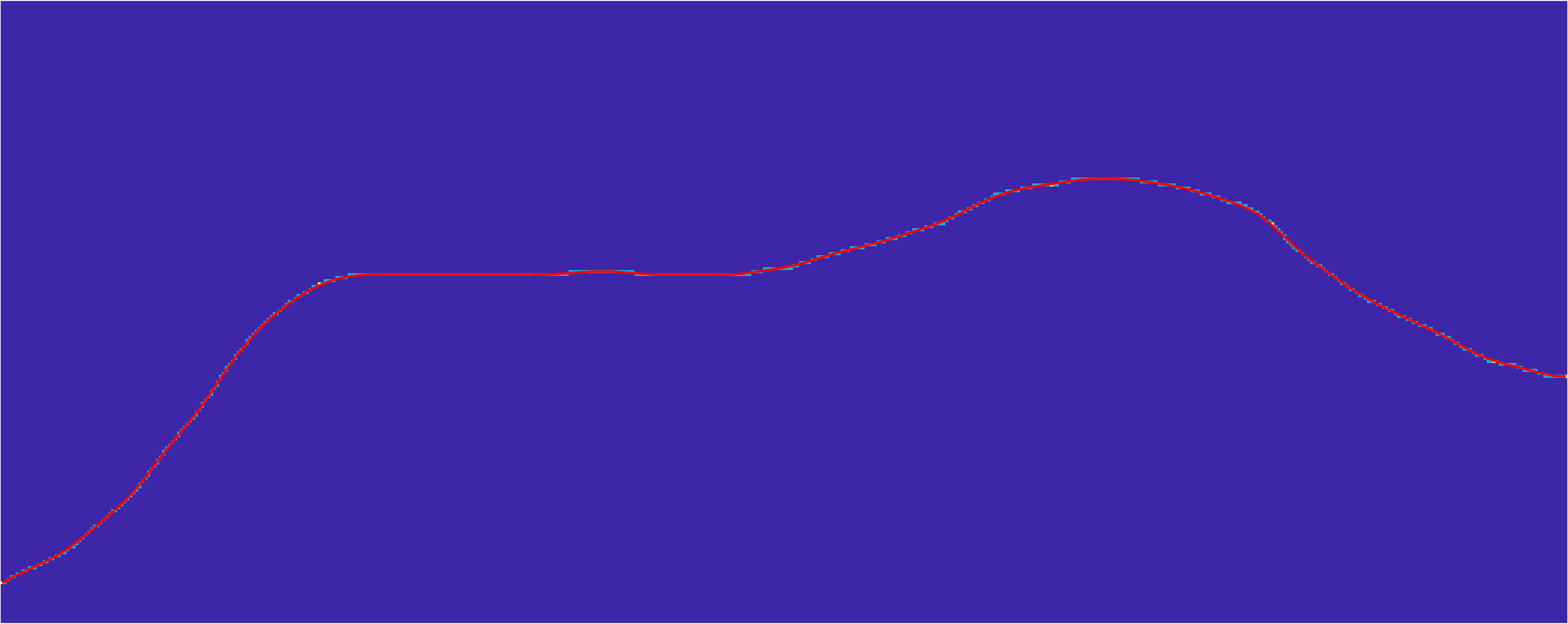

Supplement: S1 Appendix — Figures analogous to those shown in Figs. 3d, 3f, 3h, 3i, and 3j, are included. (ZIP) [file pone.0329379.s001.zip › S1 Appendix/001_Artery/i_smoothed segment_001.tif]

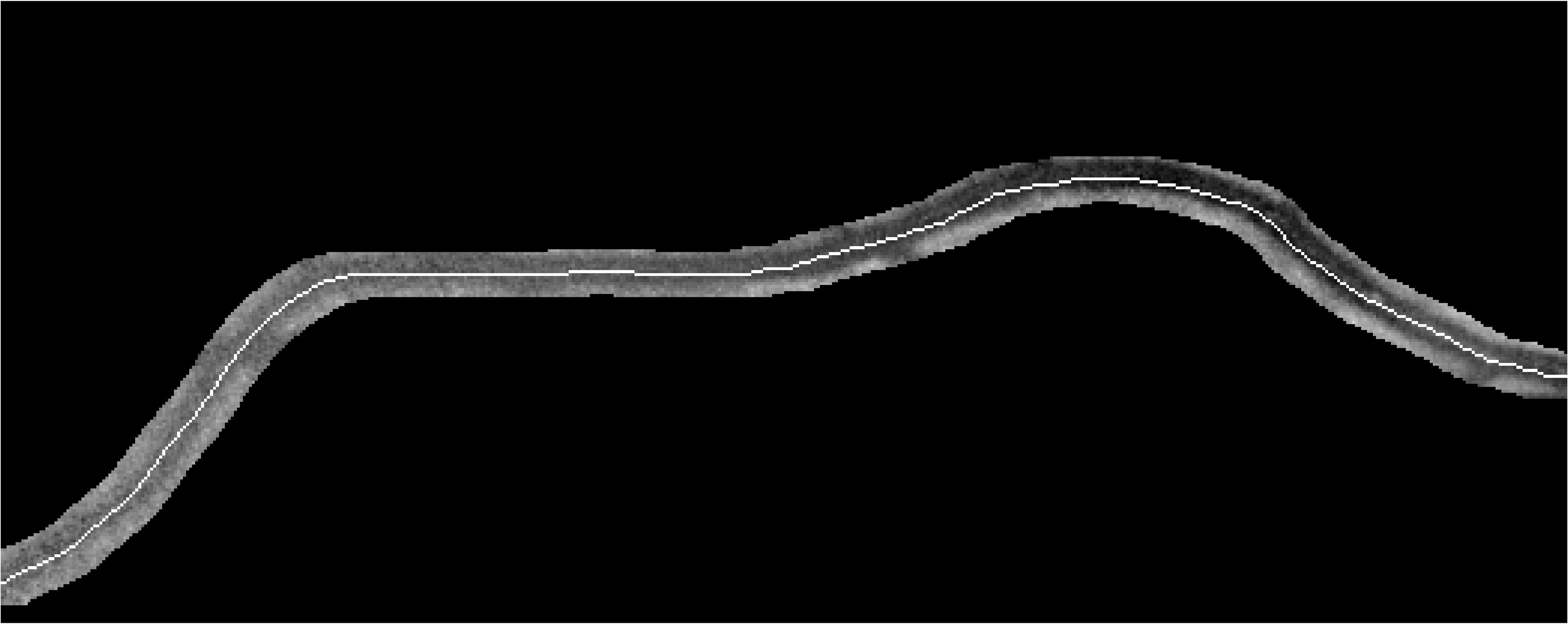

Supplement: S1 Appendix — Figures analogous to those shown in Figs. 3d, 3f, 3h, 3i, and 3j, are included. (ZIP) [file pone.0329379.s001.zip › S1 Appendix/001_Artery/d_ROI with manual trace_001.tif]

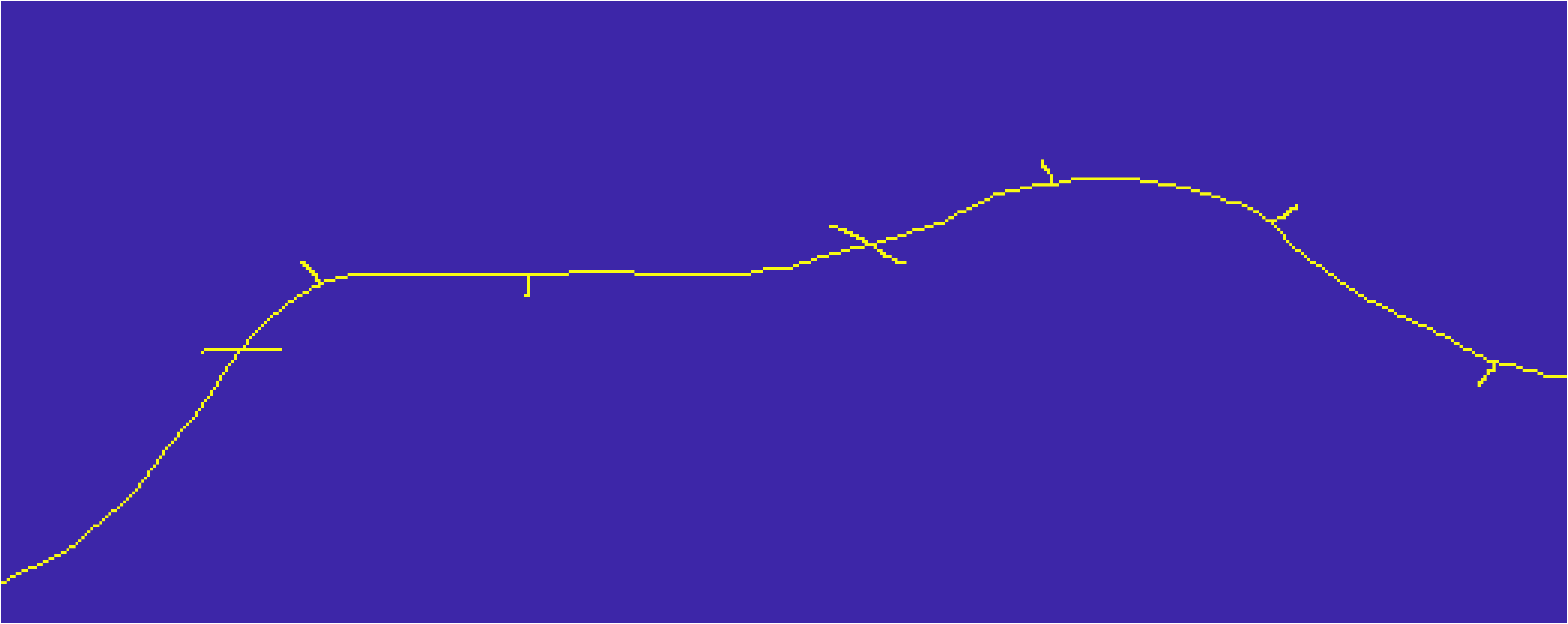

Supplement: S1 Appendix — Figures analogous to those shown in Figs. 3d, 3f, 3h, 3i, and 3j, are included. (ZIP) [file pone.0329379.s001.zip › S1 Appendix/001_Artery/f_Skeleton_001.tif]

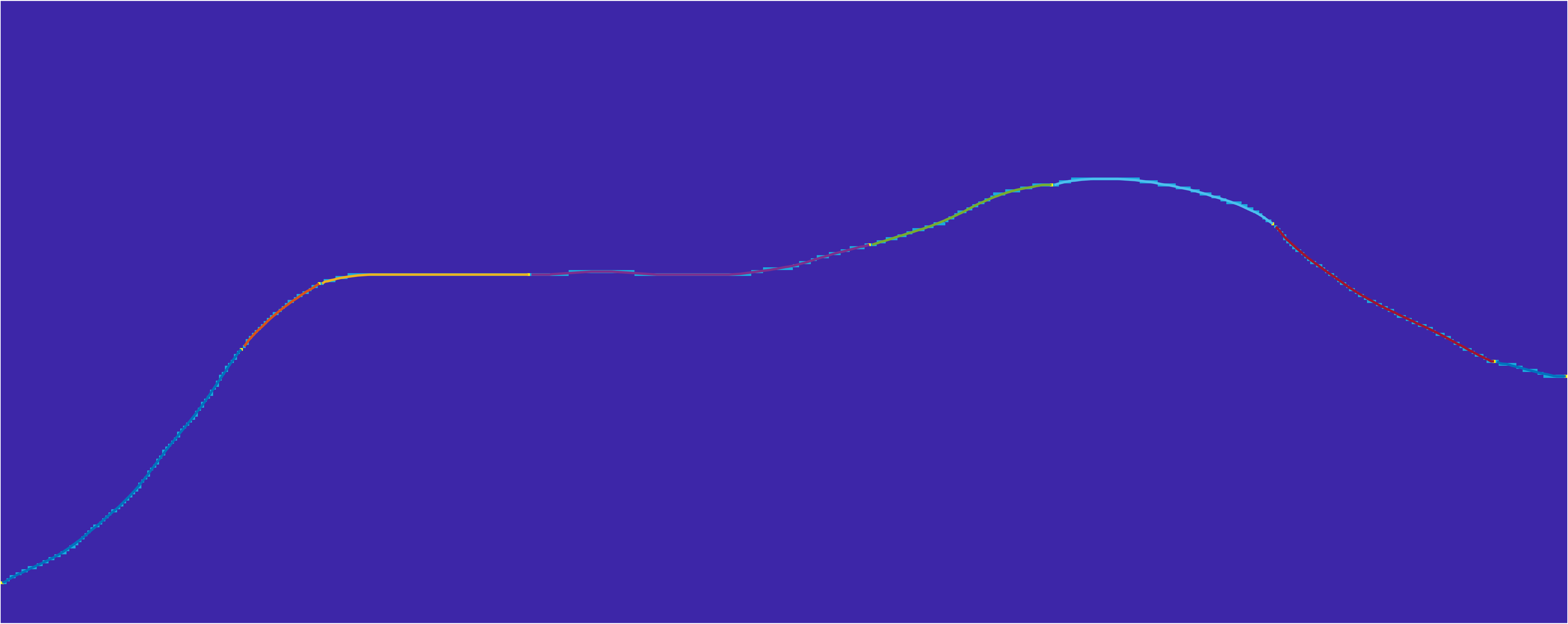

Supplement: S1 Appendix — Figures analogous to those shown in Figs. 3d, 3f, 3h, 3i, and 3j, are included. (ZIP) [file pone.0329379.s001.zip › S1 Appendix/001_Artery/j_partition_001.tif]

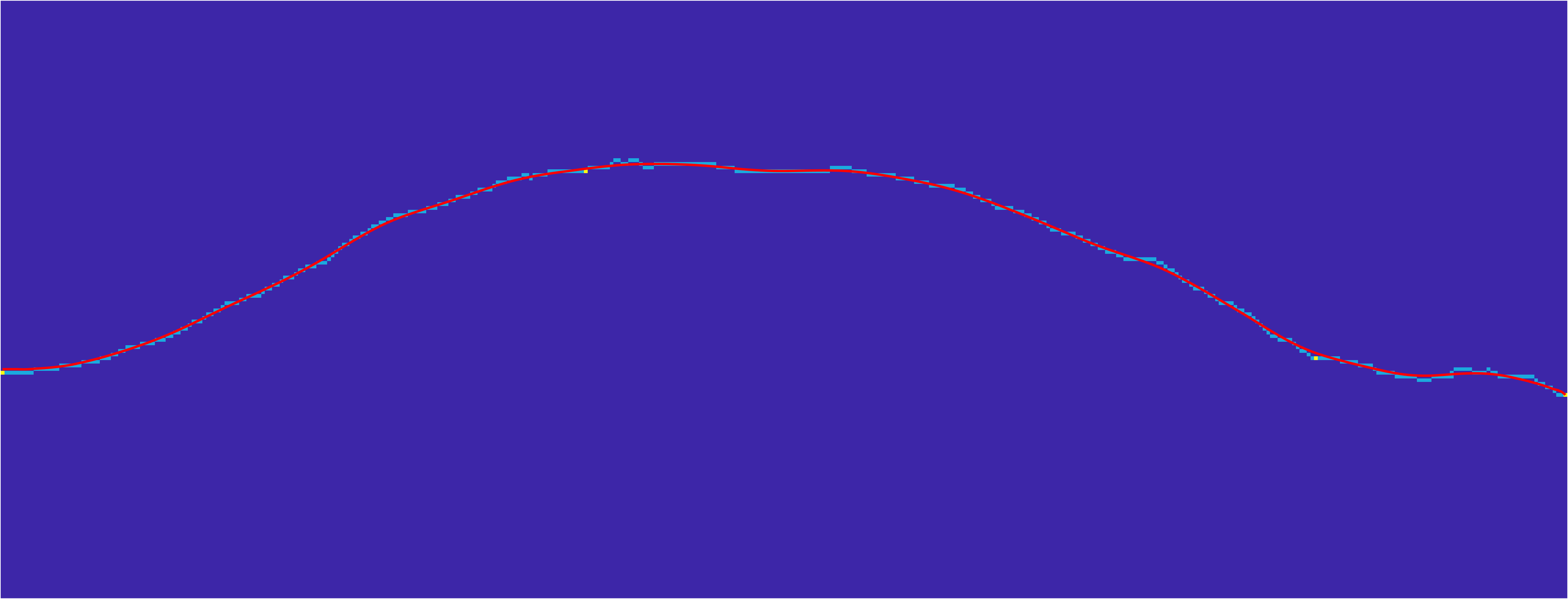

Supplement: S1 Appendix — Figures analogous to those shown in Figs. 3d, 3f, 3h, 3i, and 3j, are included. (ZIP) [file pone.0329379.s001.zip › S1 Appendix/238_Artery/i_smoothed segment_238.tif]

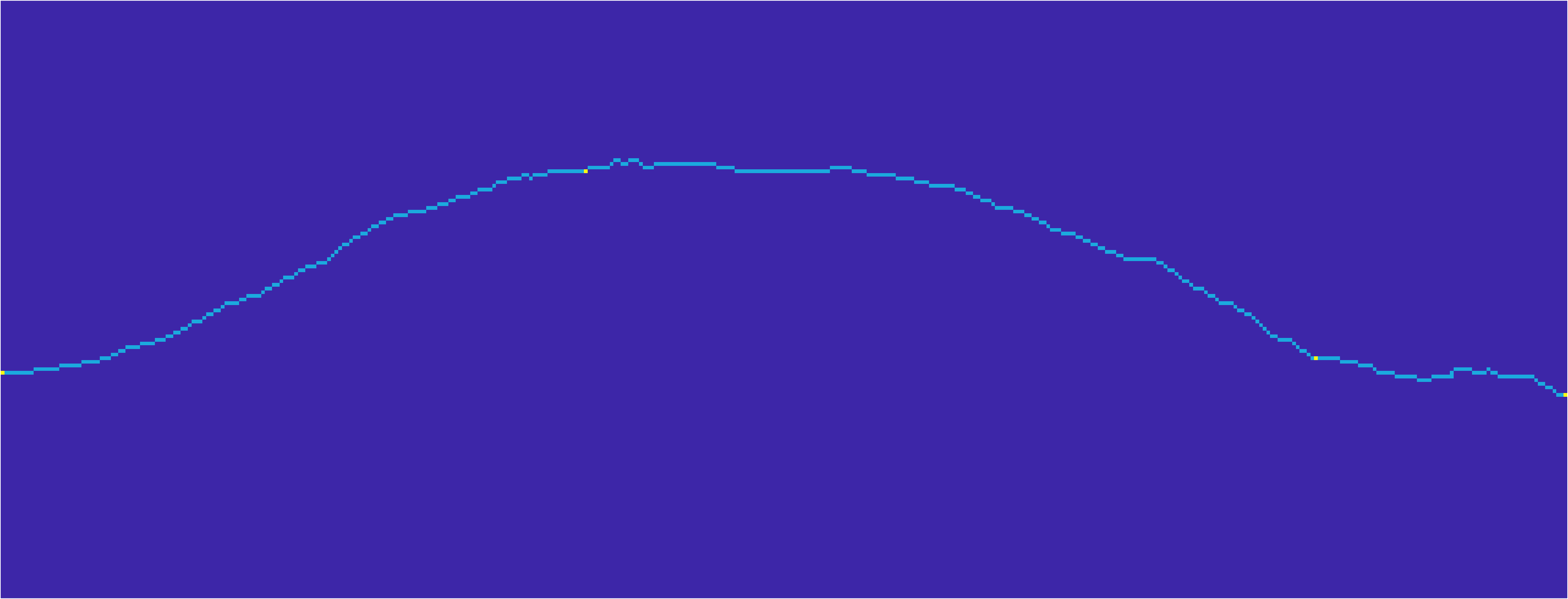

Supplement: S1 Appendix — Figures analogous to those shown in Figs. 3d, 3f, 3h, 3i, and 3j, are included. (ZIP) [file pone.0329379.s001.zip › S1 Appendix/238_Artery/h_centerline and division points_238.tif]

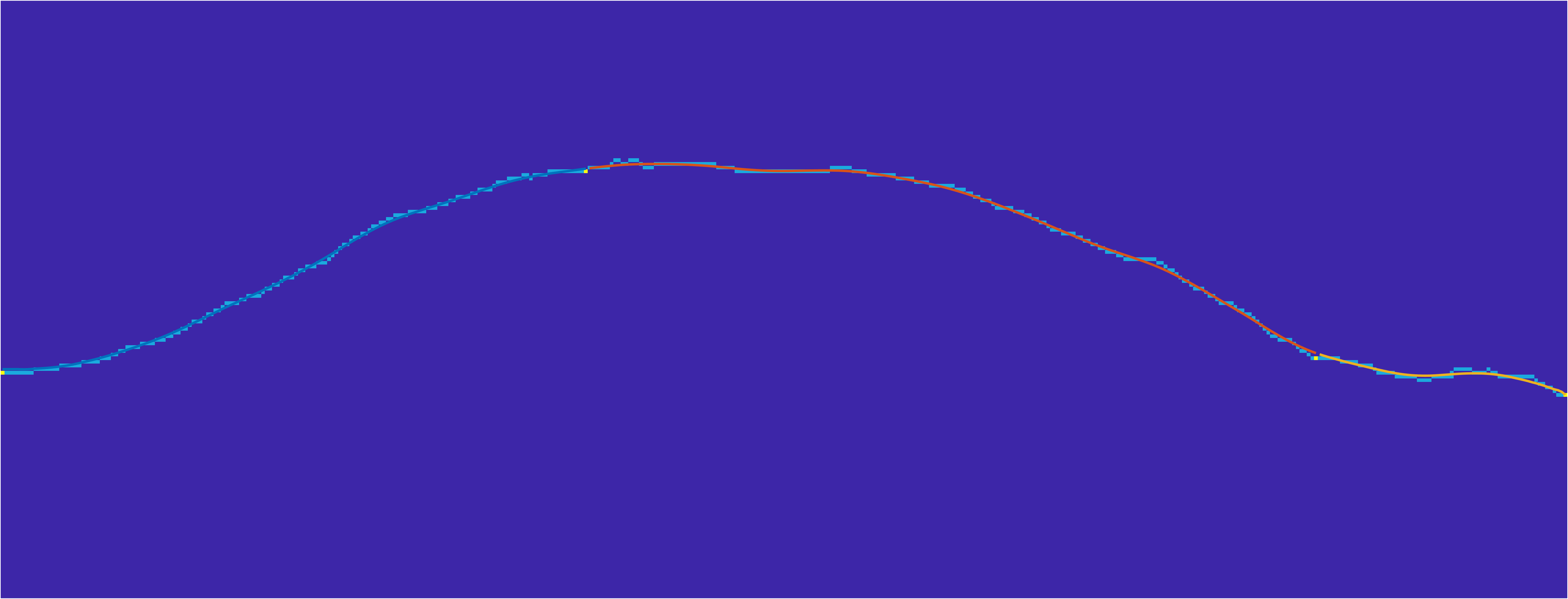

Supplement: S1 Appendix — Figures analogous to those shown in Figs. 3d, 3f, 3h, 3i, and 3j, are included. (ZIP) [file pone.0329379.s001.zip › S1 Appendix/238_Artery/j_partition_238.tif]

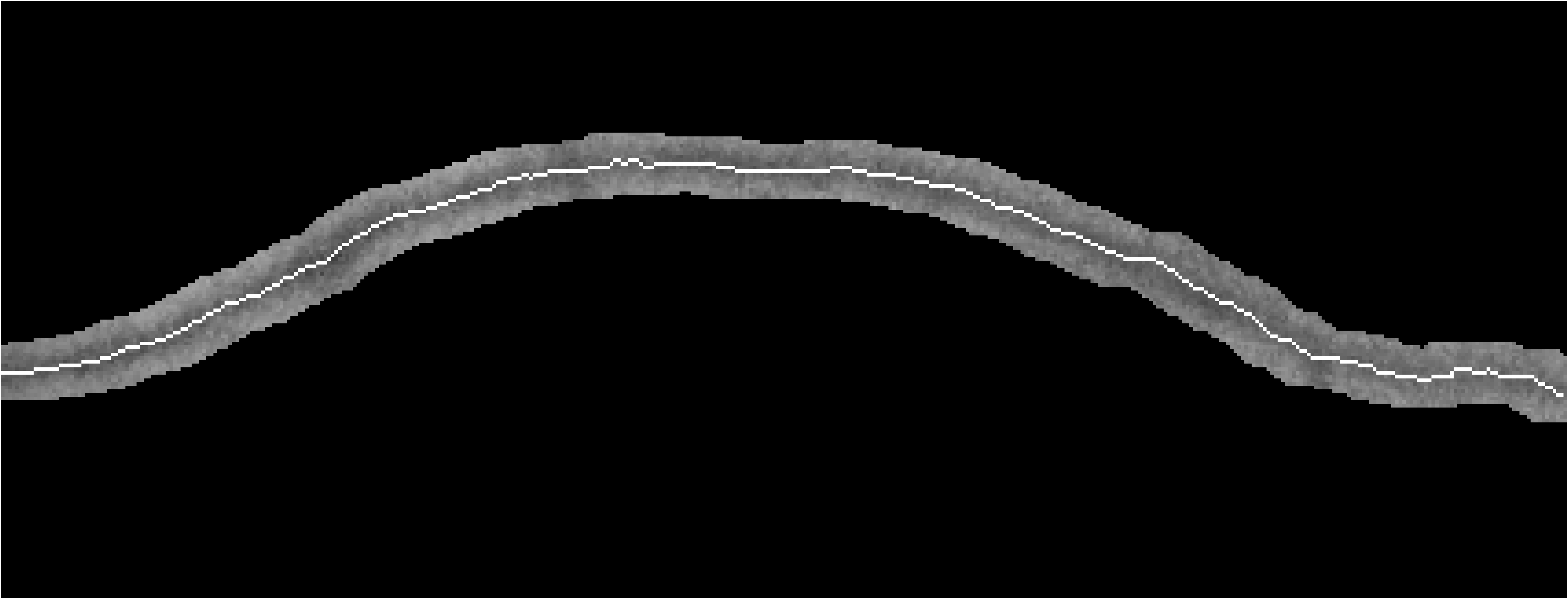

Supplement: S1 Appendix — Figures analogous to those shown in Figs. 3d, 3f, 3h, 3i, and 3j, are included. (ZIP) [file pone.0329379.s001.zip › S1 Appendix/238_Artery/d_ROI with manual trace_238.tif]

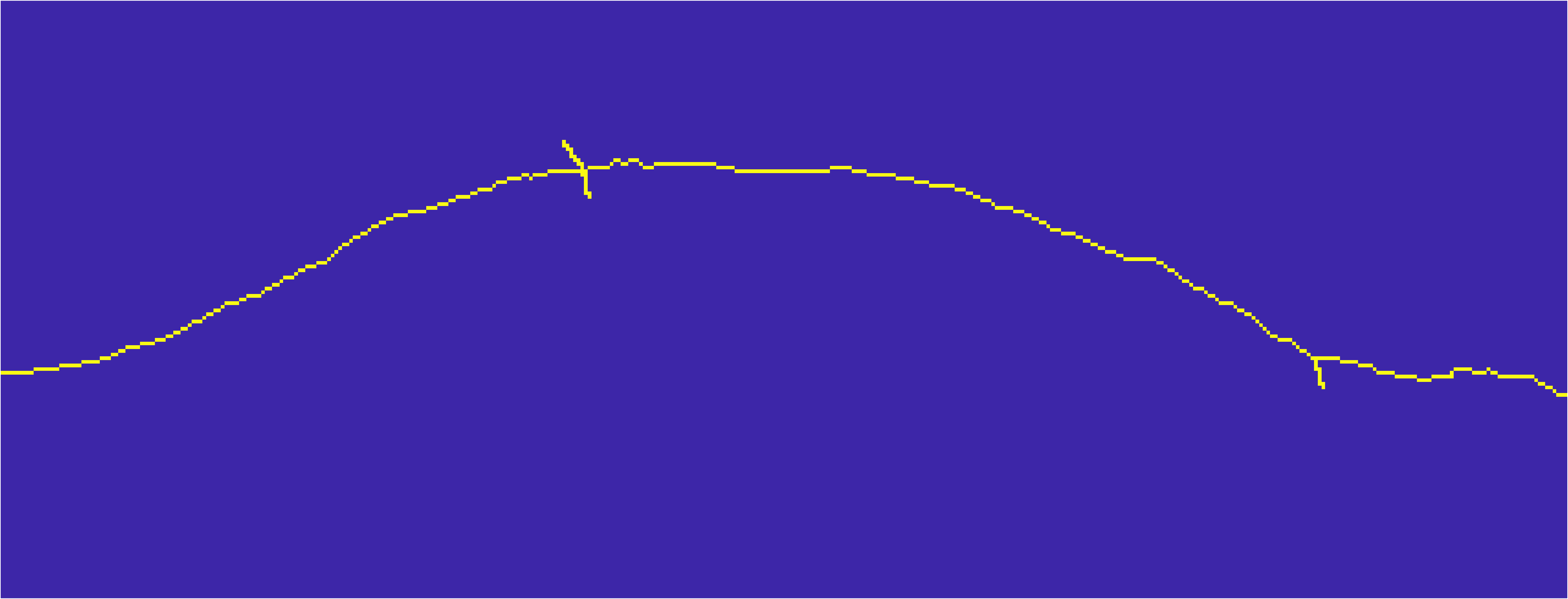

Supplement: S1 Appendix — Figures analogous to those shown in Figs. 3d, 3f, 3h, 3i, and 3j, are included. (ZIP) [file pone.0329379.s001.zip › S1 Appendix/238_Artery/f_Skeleton_238.tif]

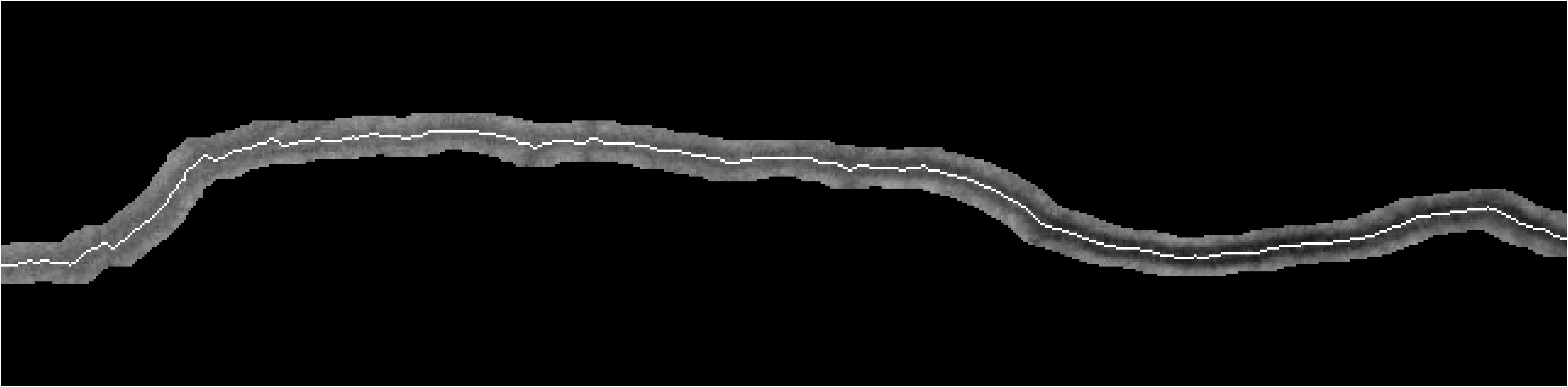

Supplement: S1 Appendix — Figures analogous to those shown in Figs. 3d, 3f, 3h, 3i, and 3j, are included. (ZIP) [file pone.0329379.s001.zip › S1 Appendix/222_Artery/d_ROI with manual trace_222.tif]
