# Supplementary figures and images for: Bridging local and global tortuosity of retinal vessels: Objective testing of index performance (part 1 of 2)
Source: PLoS One. 2025 Aug 7;20(8):e0329379. doi: 10.1371/journal.pone.0329379 (PMC12331121; doi:10.1371/journal.pone.0329379)

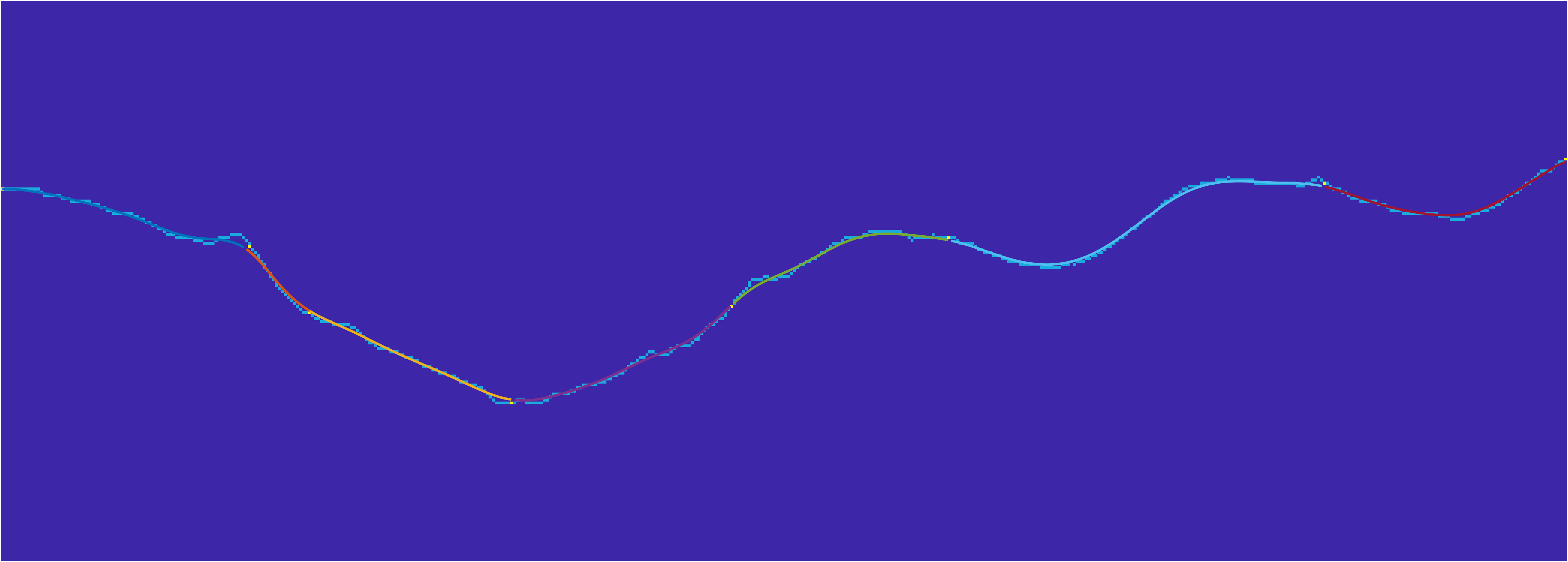

Supplement: S1 Appendix — Figures analogous to those shown in Figs. 3d, 3f, 3h, 3i, and 3j, are included. (ZIP) [file pone.0329379.s001.zip › S1 Appendix/026_Artery/j_partition_026.tif]

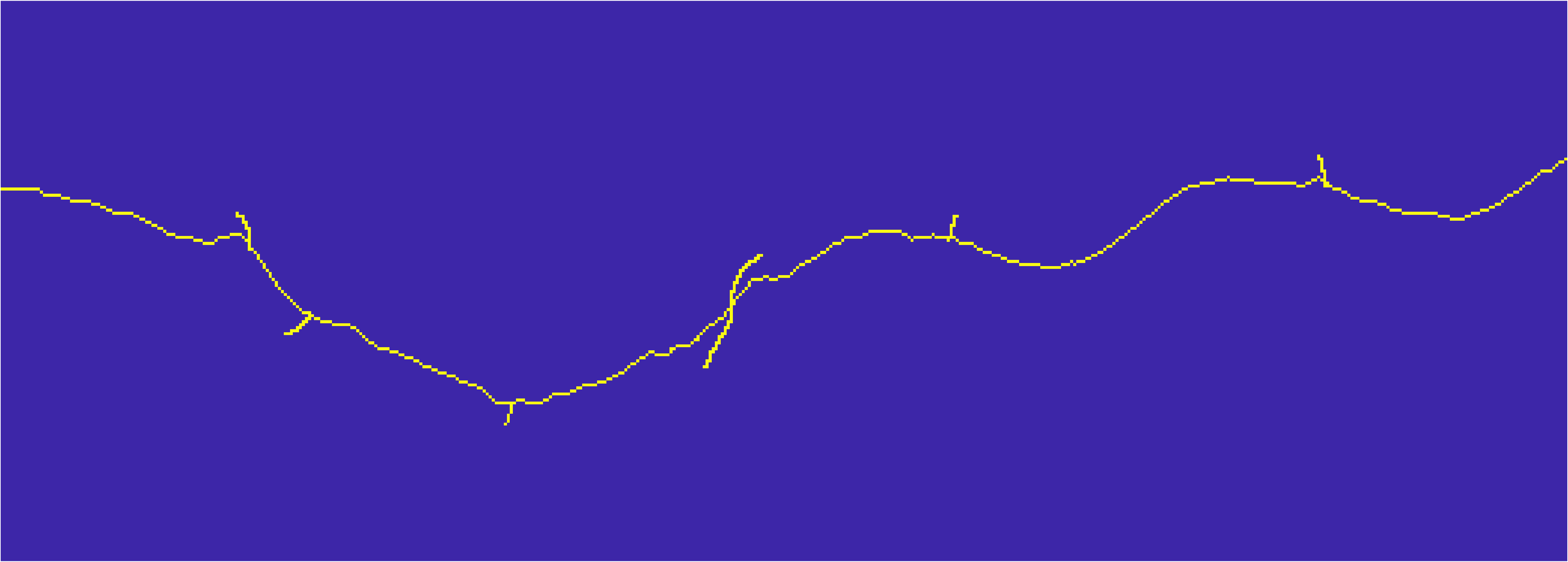

Supplement: S1 Appendix — Figures analogous to those shown in Figs. 3d, 3f, 3h, 3i, and 3j, are included. (ZIP) [file pone.0329379.s001.zip › S1 Appendix/026_Artery/f_Skeleton_026.tif]

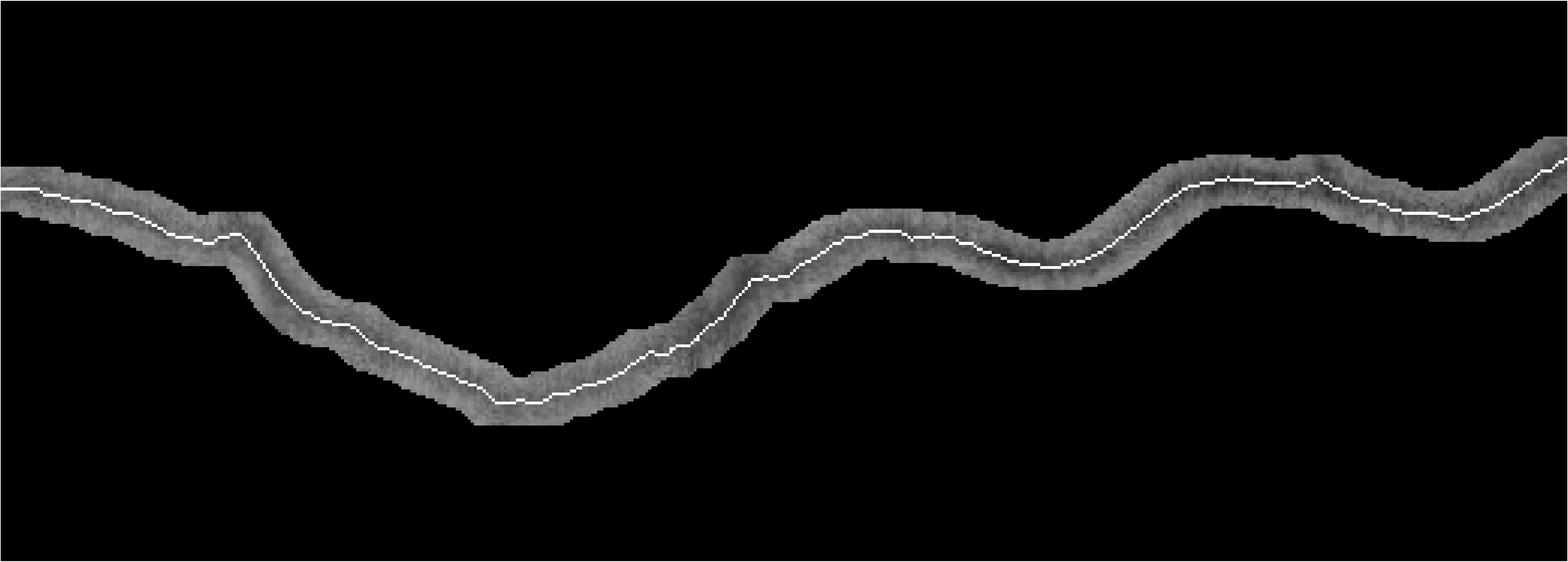

Supplement: S1 Appendix — Figures analogous to those shown in Figs. 3d, 3f, 3h, 3i, and 3j, are included. (ZIP) [file pone.0329379.s001.zip › S1 Appendix/026_Artery/d_ROI with manual trace_026.tif]

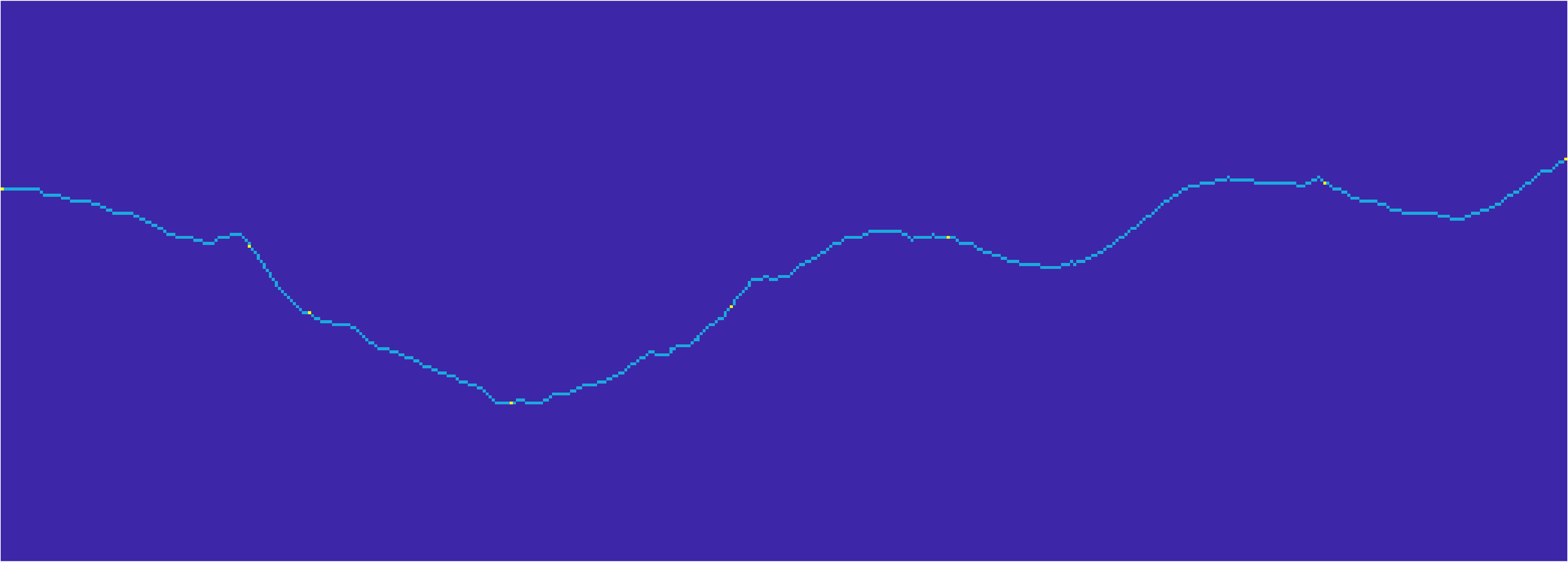

Supplement: S1 Appendix — Figures analogous to those shown in Figs. 3d, 3f, 3h, 3i, and 3j, are included. (ZIP) [file pone.0329379.s001.zip › S1 Appendix/026_Artery/h_centerline and division points_026.tif]

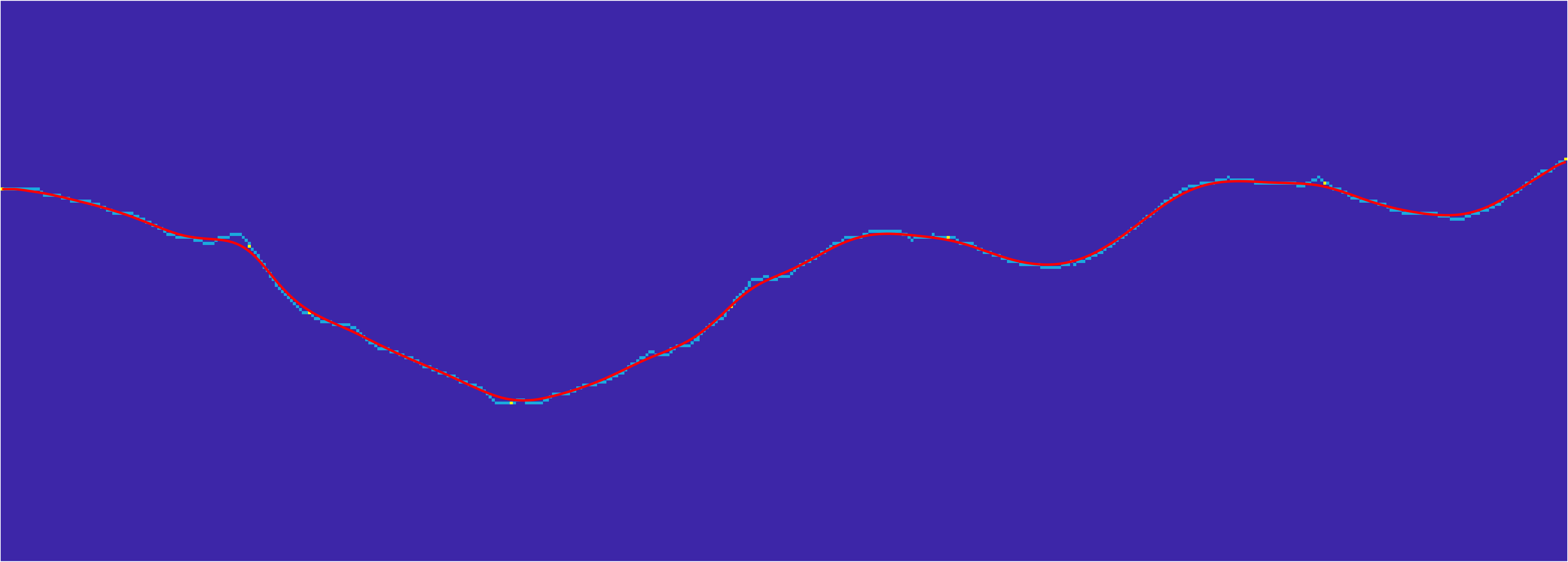

Supplement: S1 Appendix — Figures analogous to those shown in Figs. 3d, 3f, 3h, 3i, and 3j, are included. (ZIP) [file pone.0329379.s001.zip › S1 Appendix/026_Artery/i_smoothed segment_026.tif]

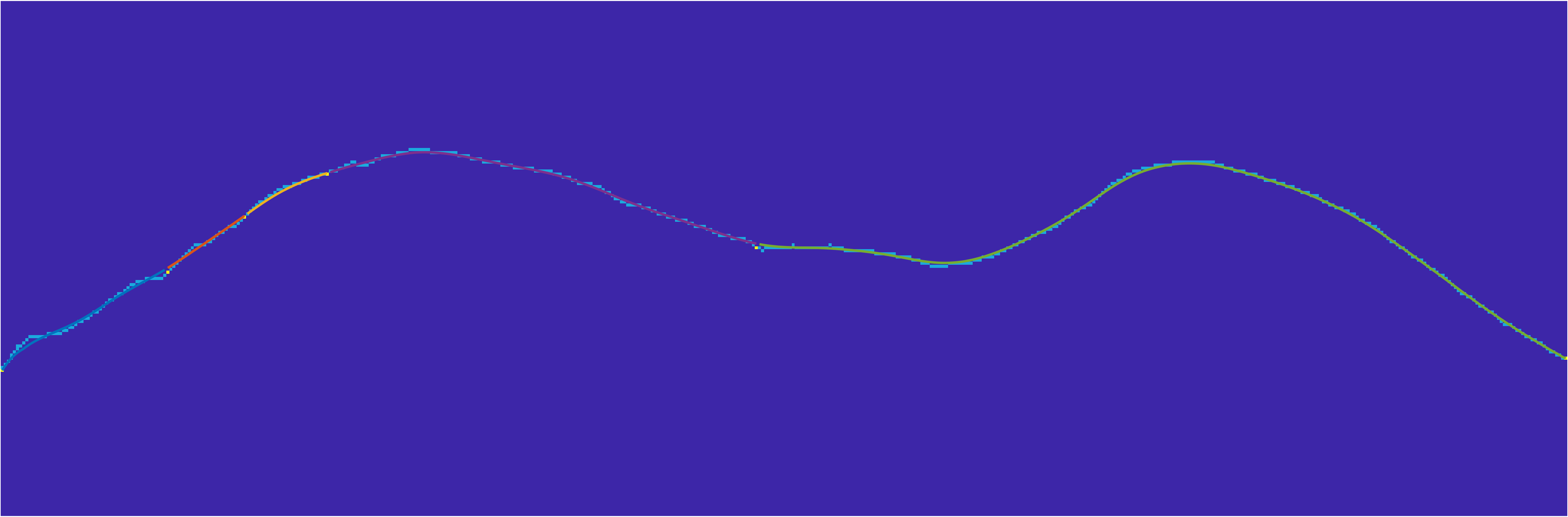

Supplement: S1 Appendix — Figures analogous to those shown in Figs. 3d, 3f, 3h, 3i, and 3j, are included. (ZIP) [file pone.0329379.s001.zip › S1 Appendix/110_Artery/j_partition_110.tif]

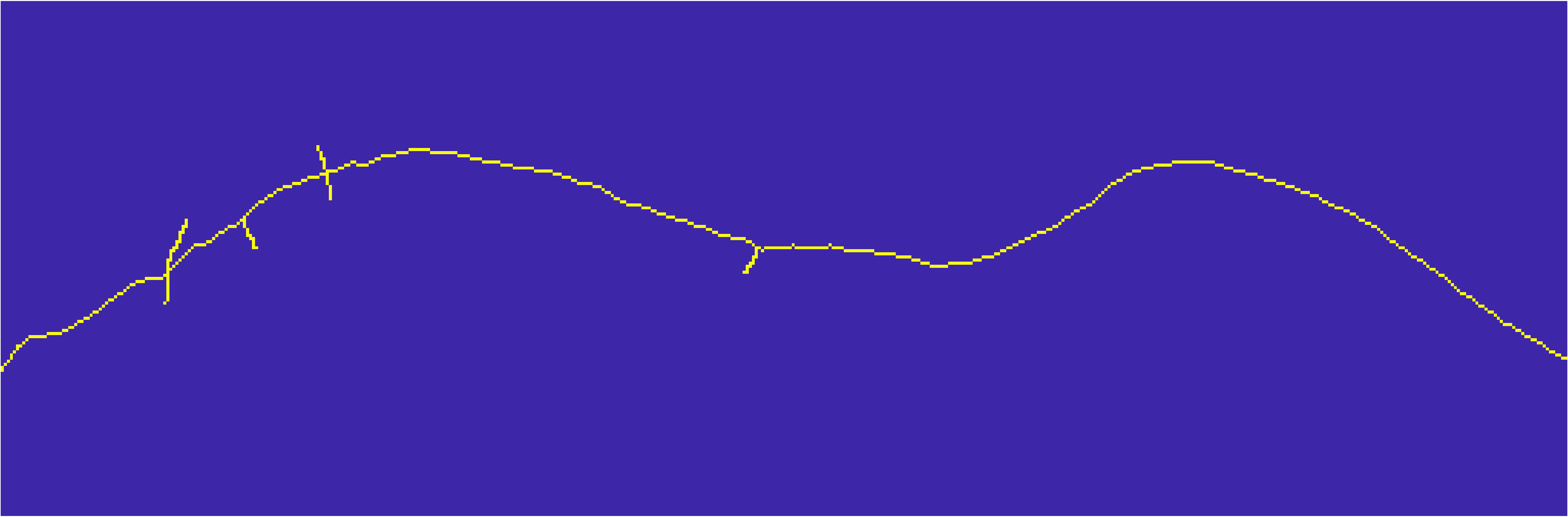

Supplement: S1 Appendix — Figures analogous to those shown in Figs. 3d, 3f, 3h, 3i, and 3j, are included. (ZIP) [file pone.0329379.s001.zip › S1 Appendix/110_Artery/f_Skeleton_110.tif]

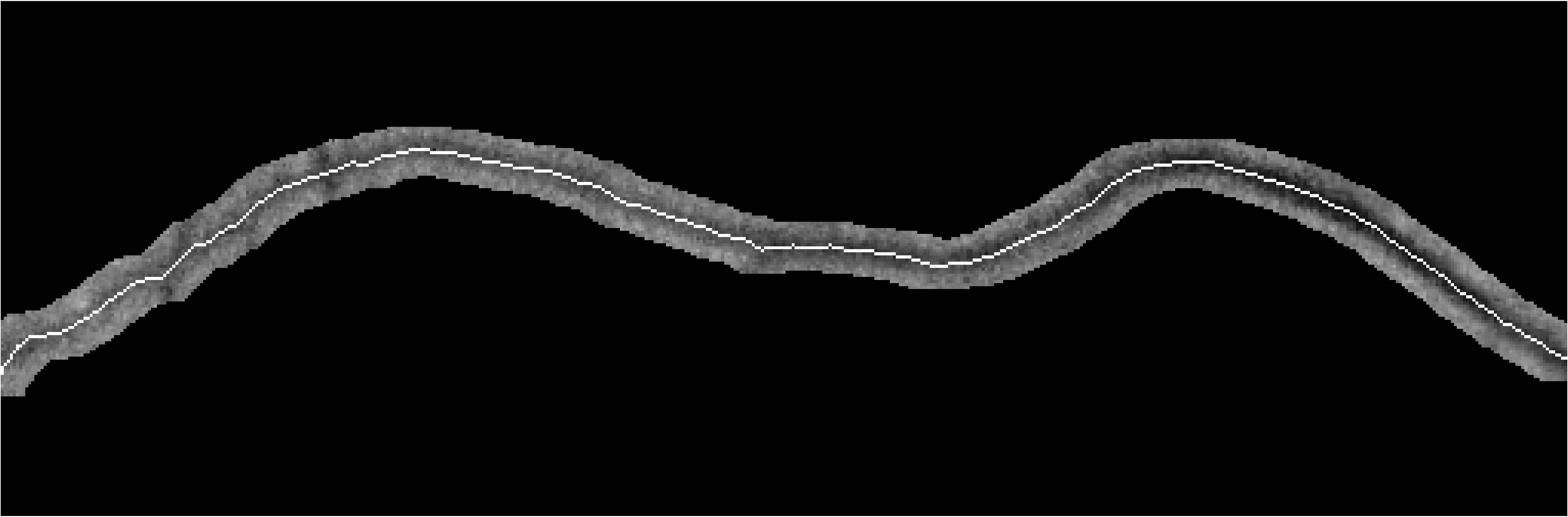

Supplement: S1 Appendix — Figures analogous to those shown in Figs. 3d, 3f, 3h, 3i, and 3j, are included. (ZIP) [file pone.0329379.s001.zip › S1 Appendix/110_Artery/d_ROI with manual trace_110.tif]

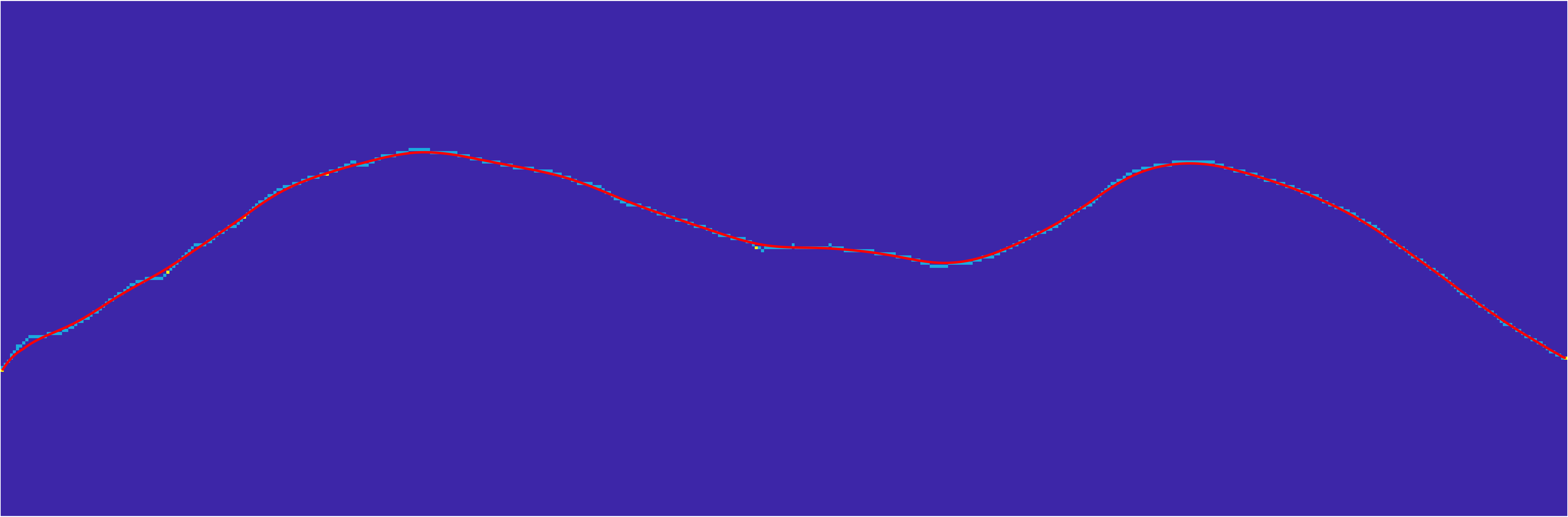

Supplement: S1 Appendix — Figures analogous to those shown in Figs. 3d, 3f, 3h, 3i, and 3j, are included. (ZIP) [file pone.0329379.s001.zip › S1 Appendix/110_Artery/i_smoothed segment_110.tif]

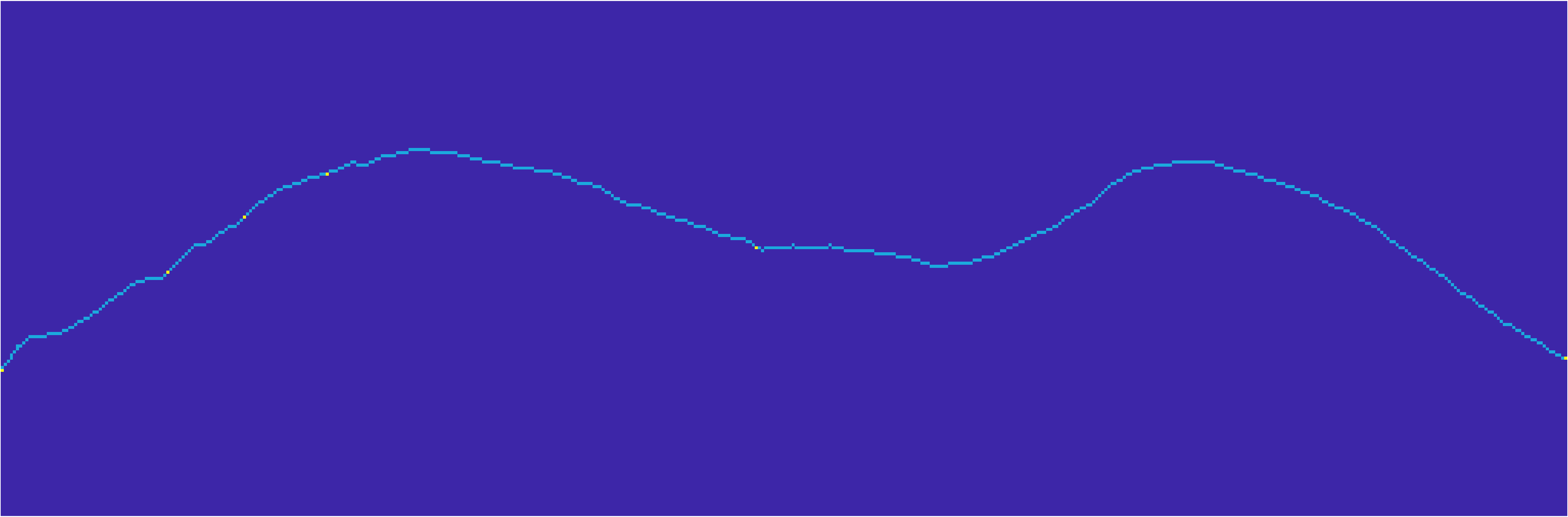

Supplement: S1 Appendix — Figures analogous to those shown in Figs. 3d, 3f, 3h, 3i, and 3j, are included. (ZIP) [file pone.0329379.s001.zip › S1 Appendix/110_Artery/h_centerline and division points_110.tif]

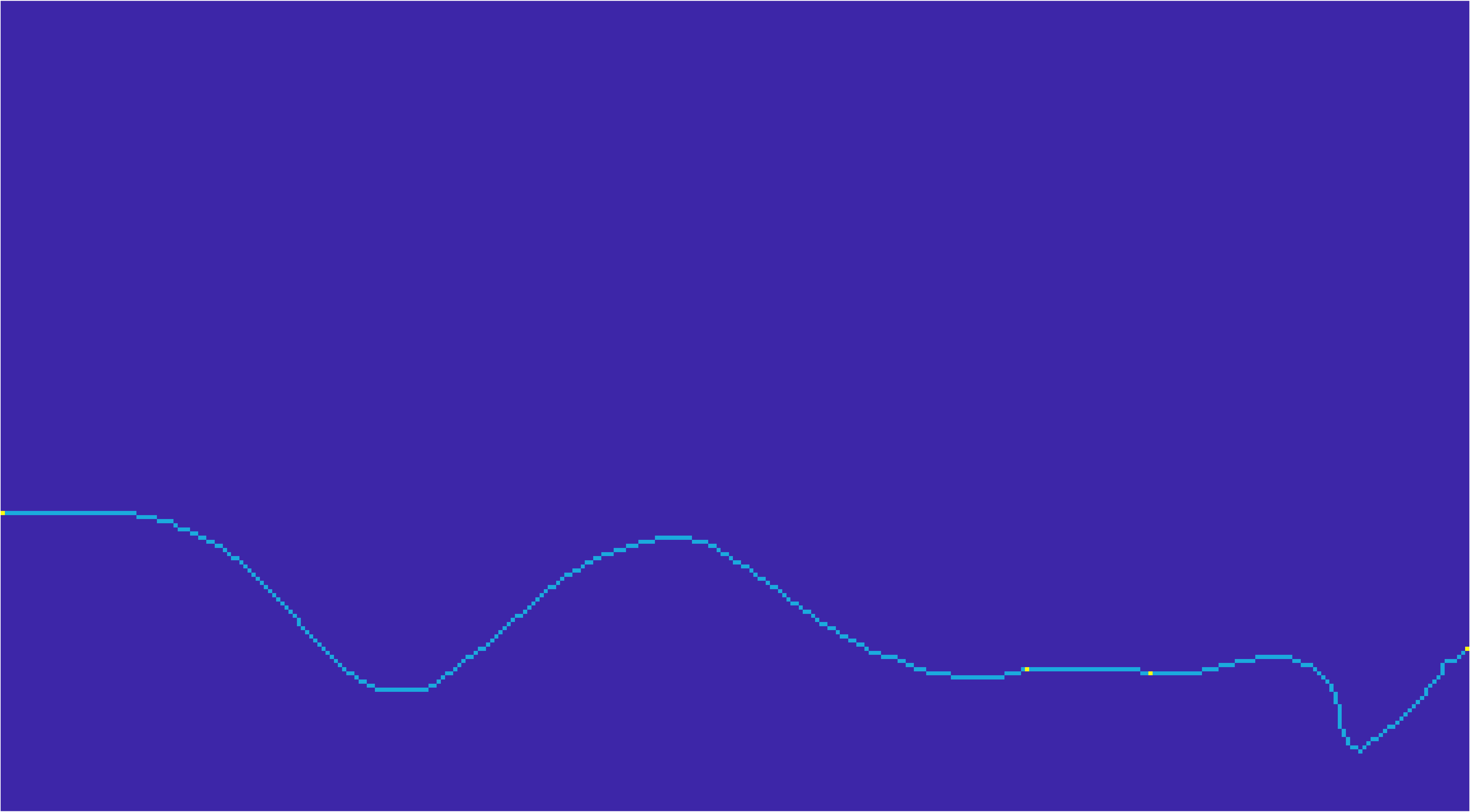

Supplement: S1 Appendix — Figures analogous to those shown in Figs. 3d, 3f, 3h, 3i, and 3j, are included. (ZIP) [file pone.0329379.s001.zip › S1 Appendix/010_Artery/h_centerline and division points_010.tif]

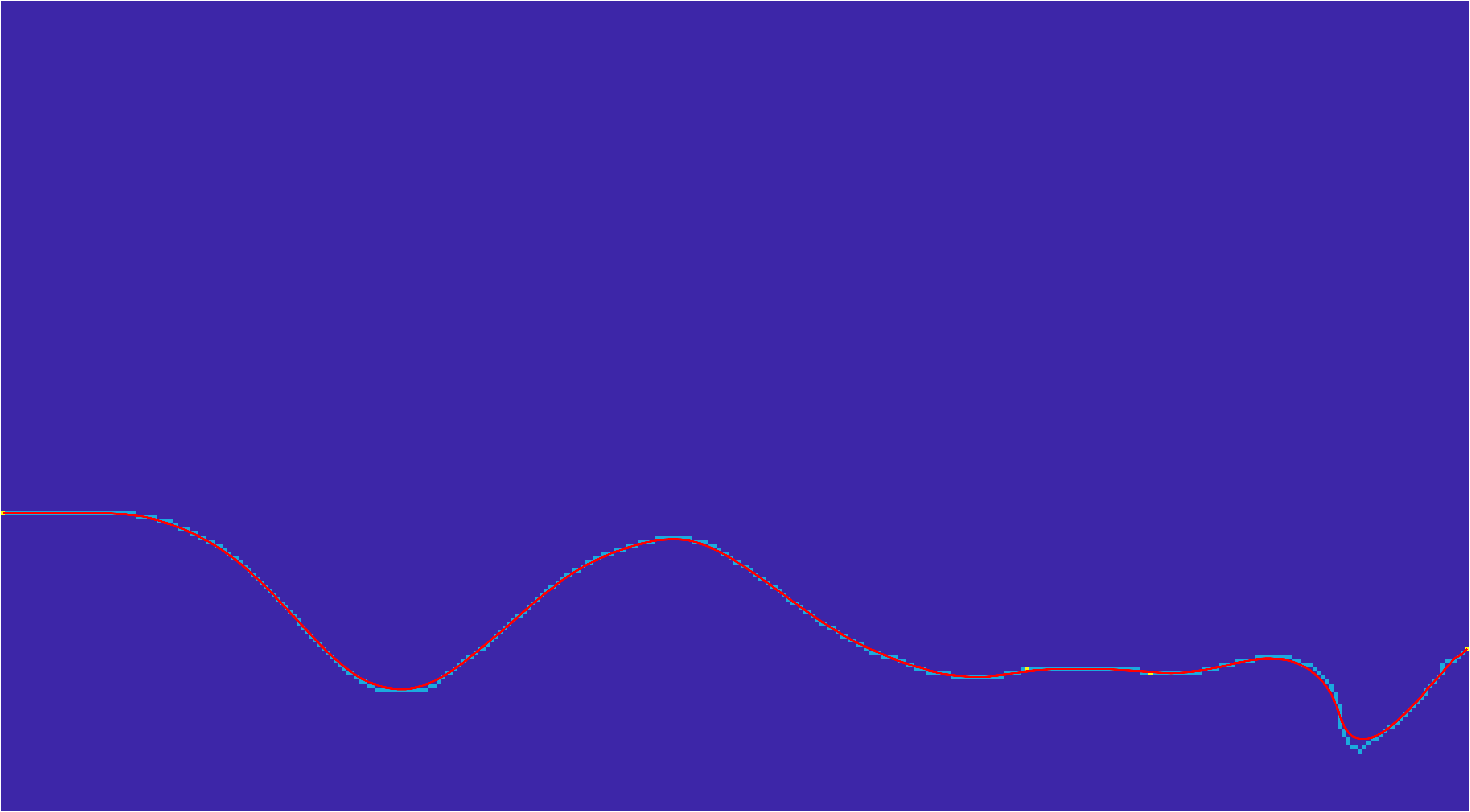

Supplement: S1 Appendix — Figures analogous to those shown in Figs. 3d, 3f, 3h, 3i, and 3j, are included. (ZIP) [file pone.0329379.s001.zip › S1 Appendix/010_Artery/i_smoothed segment_010.tif]

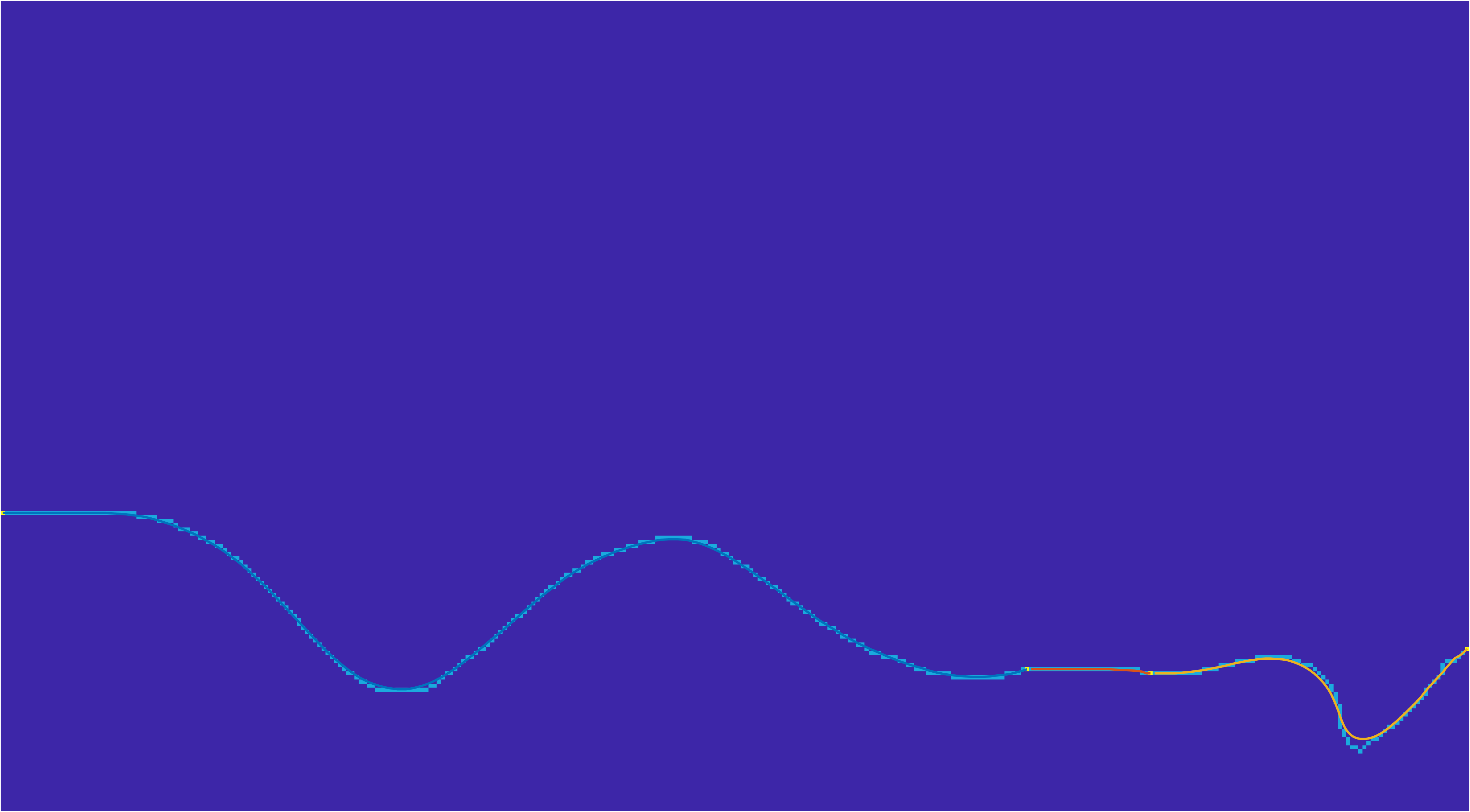

Supplement: S1 Appendix — Figures analogous to those shown in Figs. 3d, 3f, 3h, 3i, and 3j, are included. (ZIP) [file pone.0329379.s001.zip › S1 Appendix/010_Artery/j_partition_010.tif]

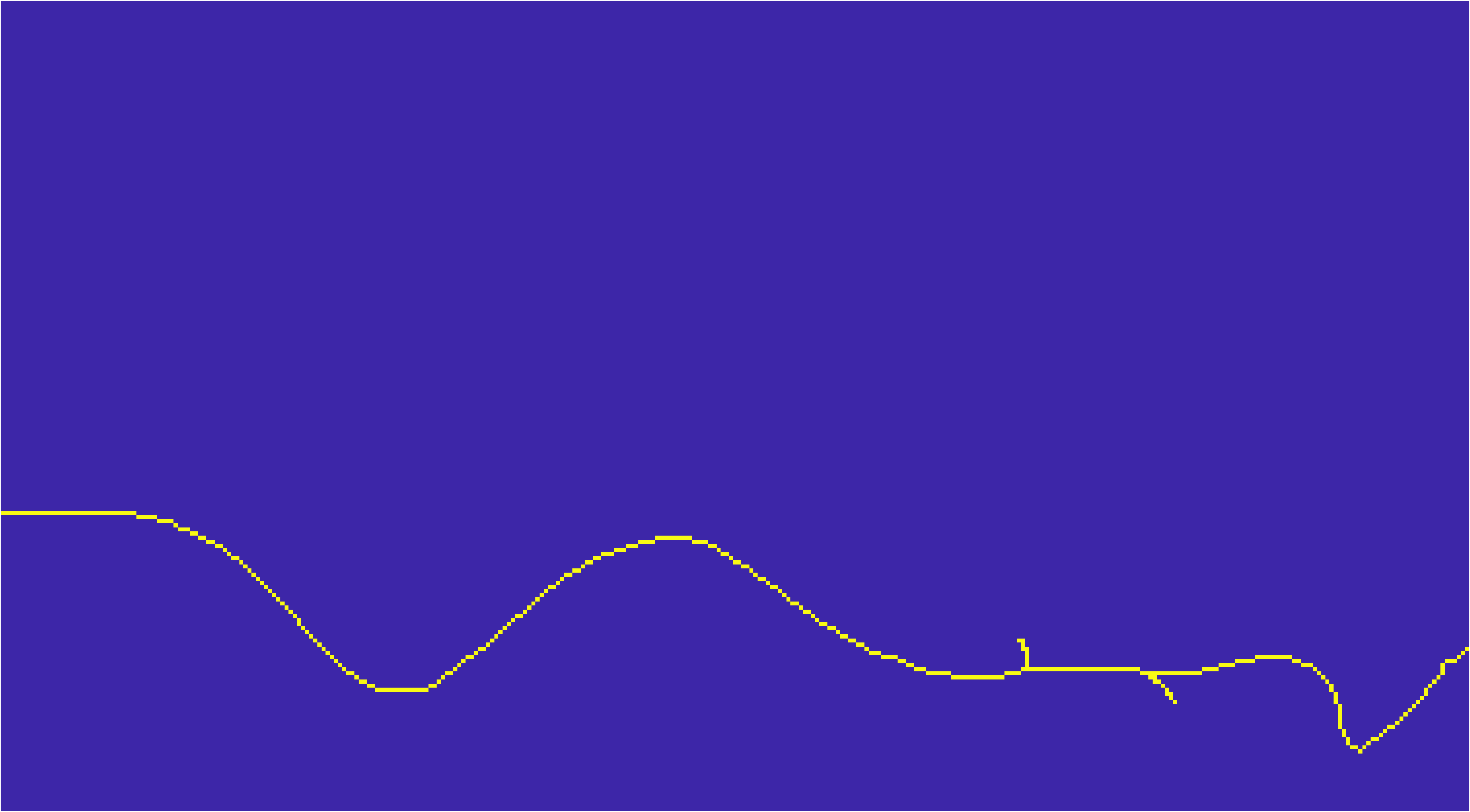

Supplement: S1 Appendix — Figures analogous to those shown in Figs. 3d, 3f, 3h, 3i, and 3j, are included. (ZIP) [file pone.0329379.s001.zip › S1 Appendix/010_Artery/f_Skeleton_010.tif]

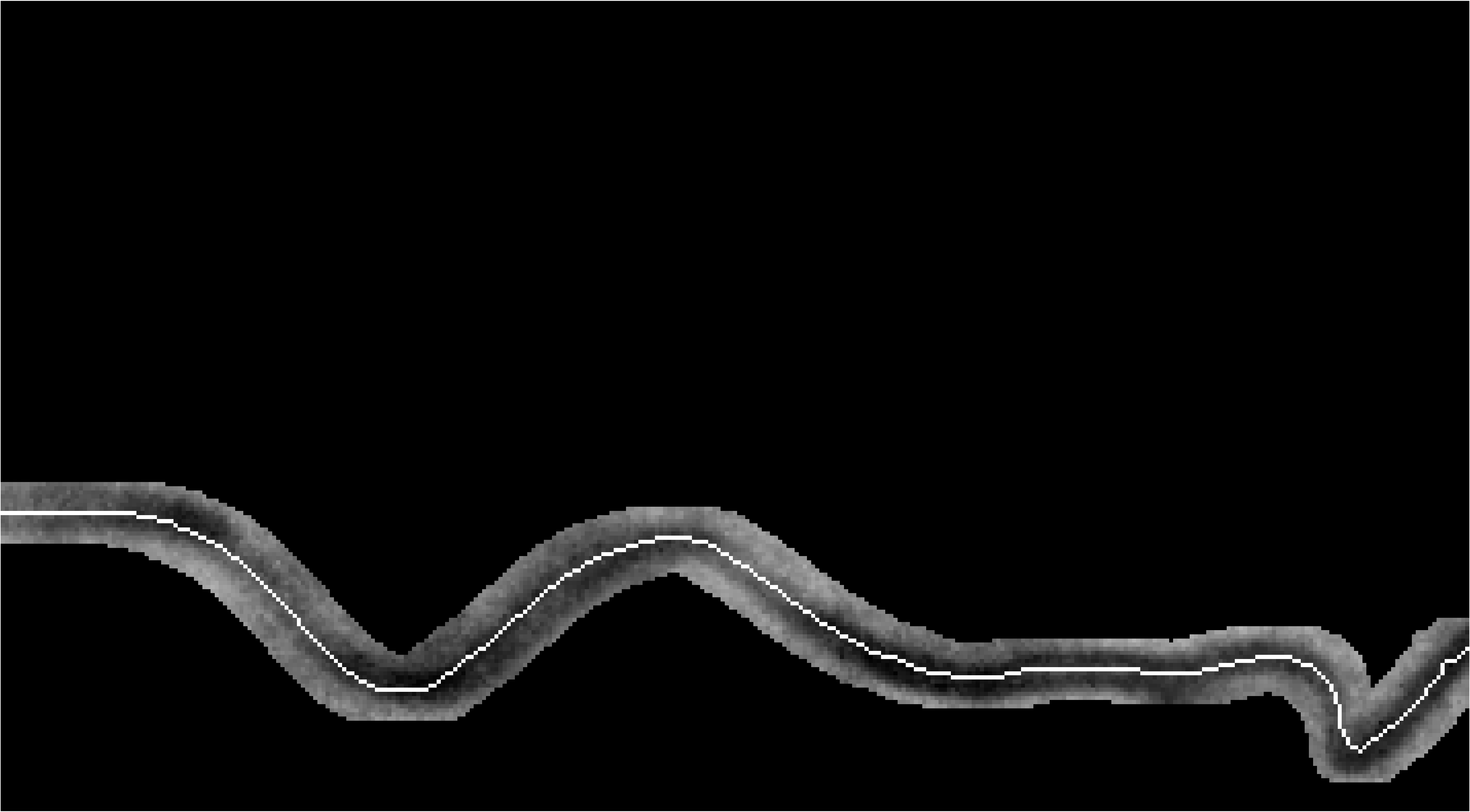

Supplement: S1 Appendix — Figures analogous to those shown in Figs. 3d, 3f, 3h, 3i, and 3j, are included. (ZIP) [file pone.0329379.s001.zip › S1 Appendix/010_Artery/d_ROI with manual trace_010.tif]

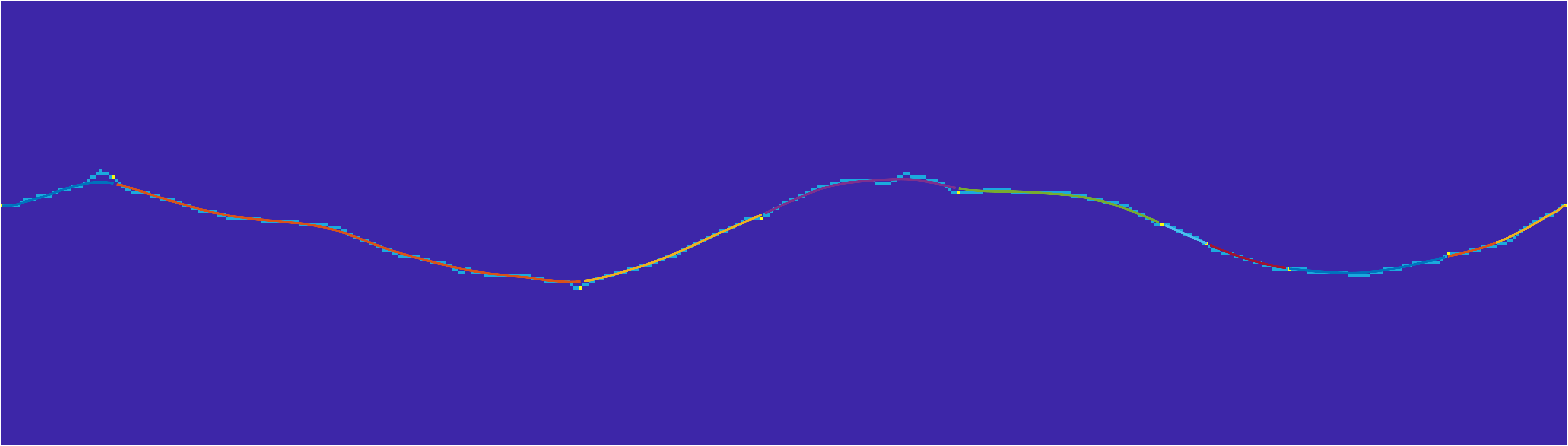

Supplement: S1 Appendix — Figures analogous to those shown in Figs. 3d, 3f, 3h, 3i, and 3j, are included. (ZIP) [file pone.0329379.s001.zip › S1 Appendix/073_Artery/j_partition_073.tif]

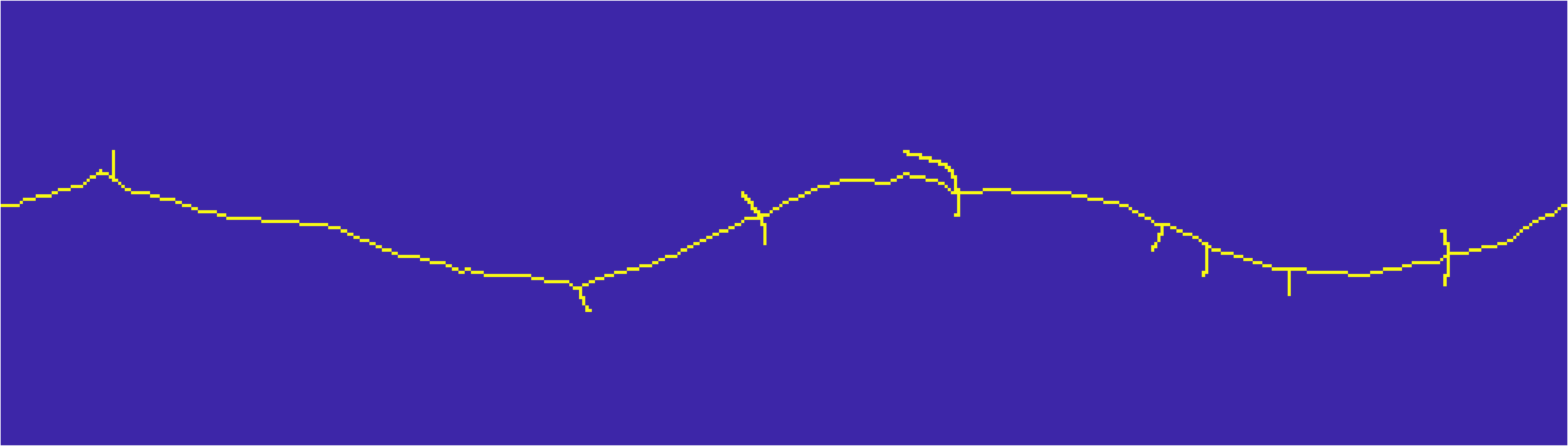

Supplement: S1 Appendix — Figures analogous to those shown in Figs. 3d, 3f, 3h, 3i, and 3j, are included. (ZIP) [file pone.0329379.s001.zip › S1 Appendix/073_Artery/f_Skeleton_073.tif]

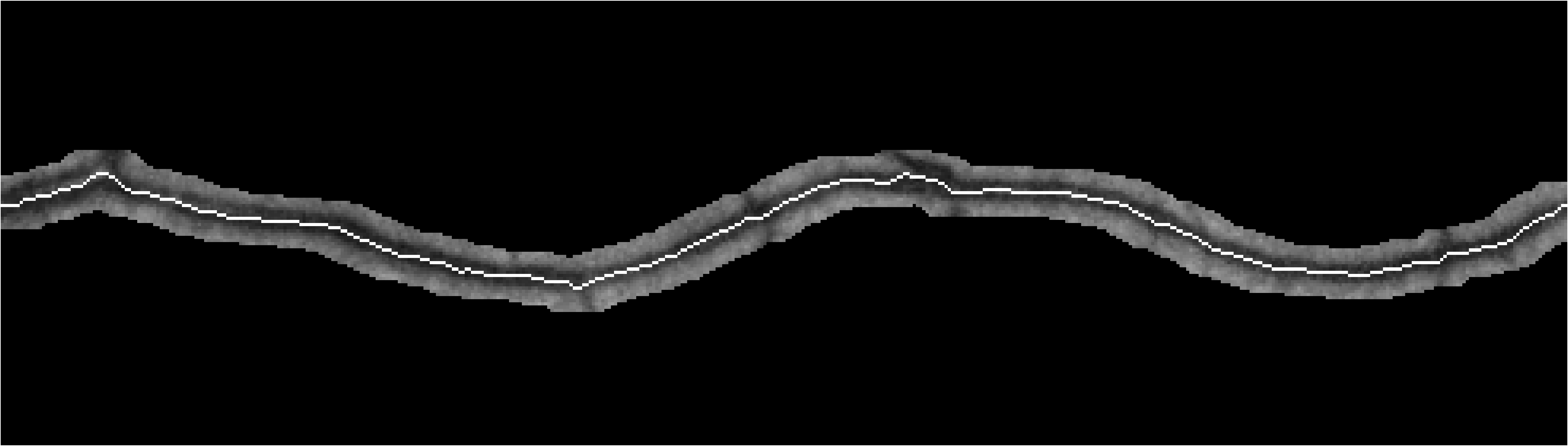

Supplement: S1 Appendix — Figures analogous to those shown in Figs. 3d, 3f, 3h, 3i, and 3j, are included. (ZIP) [file pone.0329379.s001.zip › S1 Appendix/073_Artery/d_ROI with manual trace_073.tif]

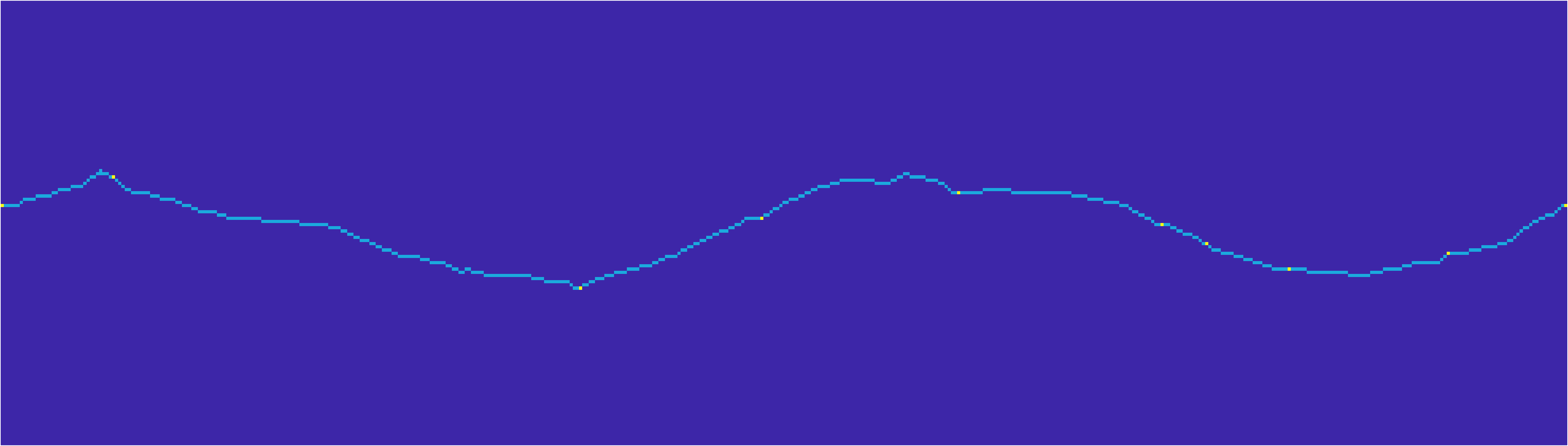

Supplement: S1 Appendix — Figures analogous to those shown in Figs. 3d, 3f, 3h, 3i, and 3j, are included. (ZIP) [file pone.0329379.s001.zip › S1 Appendix/073_Artery/h_centerline and division points_073.tif]

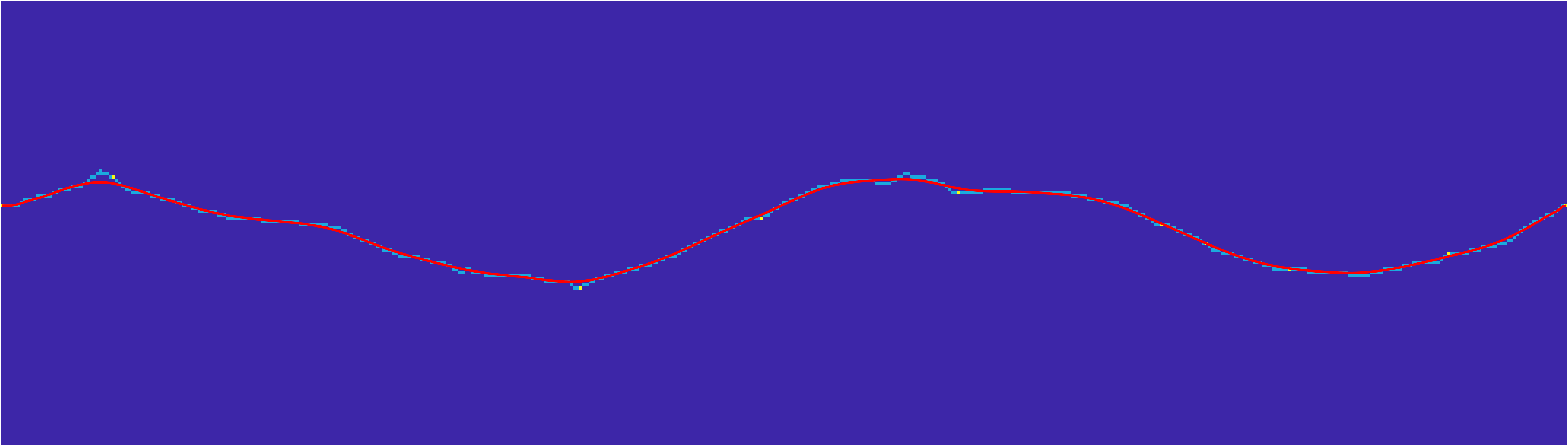

Supplement: S1 Appendix — Figures analogous to those shown in Figs. 3d, 3f, 3h, 3i, and 3j, are included. (ZIP) [file pone.0329379.s001.zip › S1 Appendix/073_Artery/i_smoothed segment_073.tif]

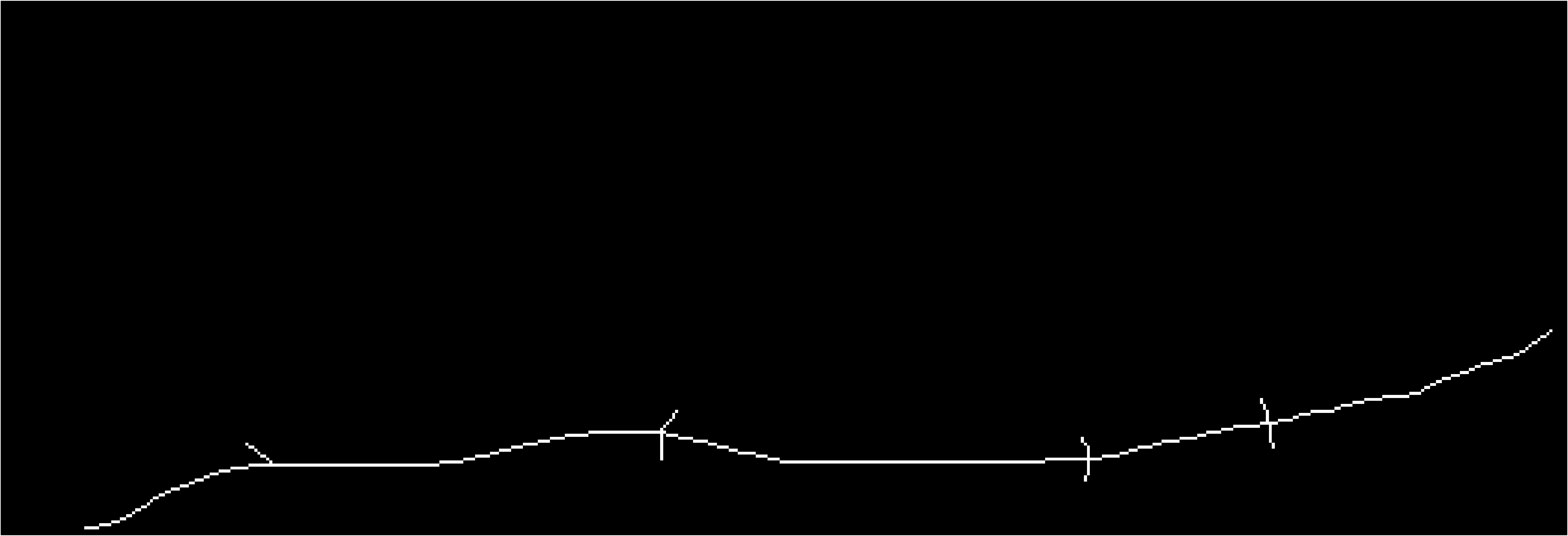

Supplement: S1 Appendix — Figures analogous to those shown in Figs. 3d, 3f, 3h, 3i, and 3j, are included. (ZIP) [file pone.0329379.s001.zip › S1 Appendix/102_Artery/f_Skeleton_102.tif]

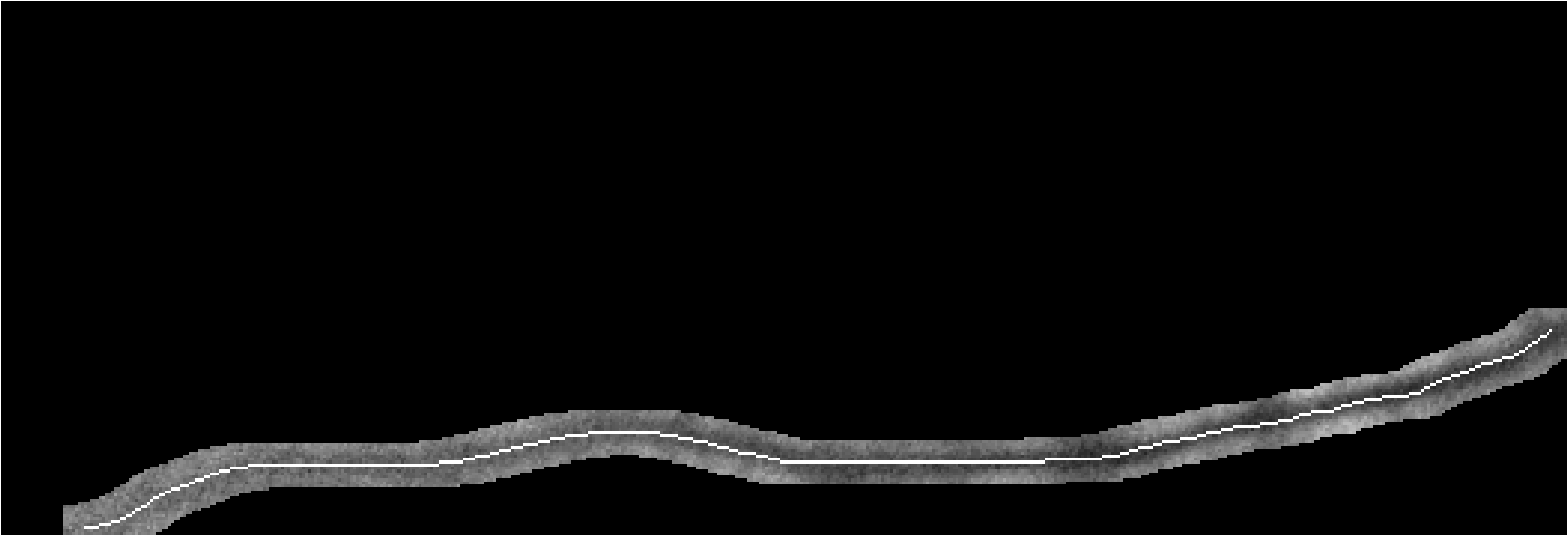

Supplement: S1 Appendix — Figures analogous to those shown in Figs. 3d, 3f, 3h, 3i, and 3j, are included. (ZIP) [file pone.0329379.s001.zip › S1 Appendix/102_Artery/d_ROI with manual trace_102.tif]

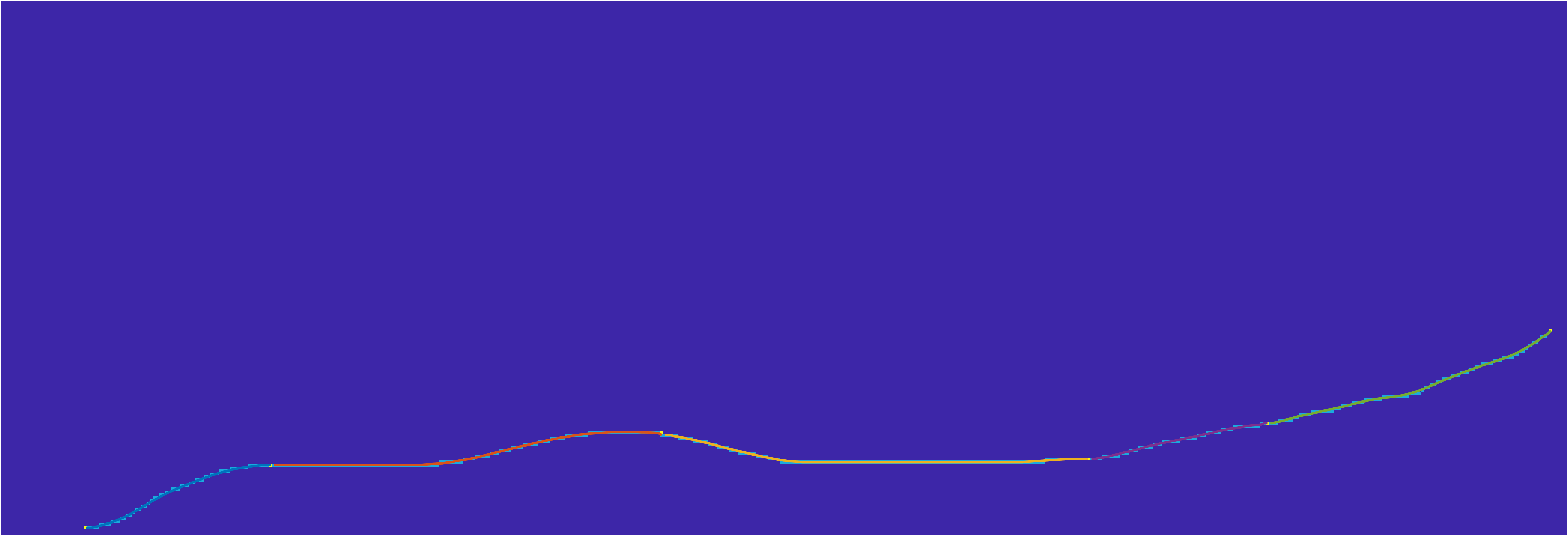

Supplement: S1 Appendix — Figures analogous to those shown in Figs. 3d, 3f, 3h, 3i, and 3j, are included. (ZIP) [file pone.0329379.s001.zip › S1 Appendix/102_Artery/j_partition_102.tif]

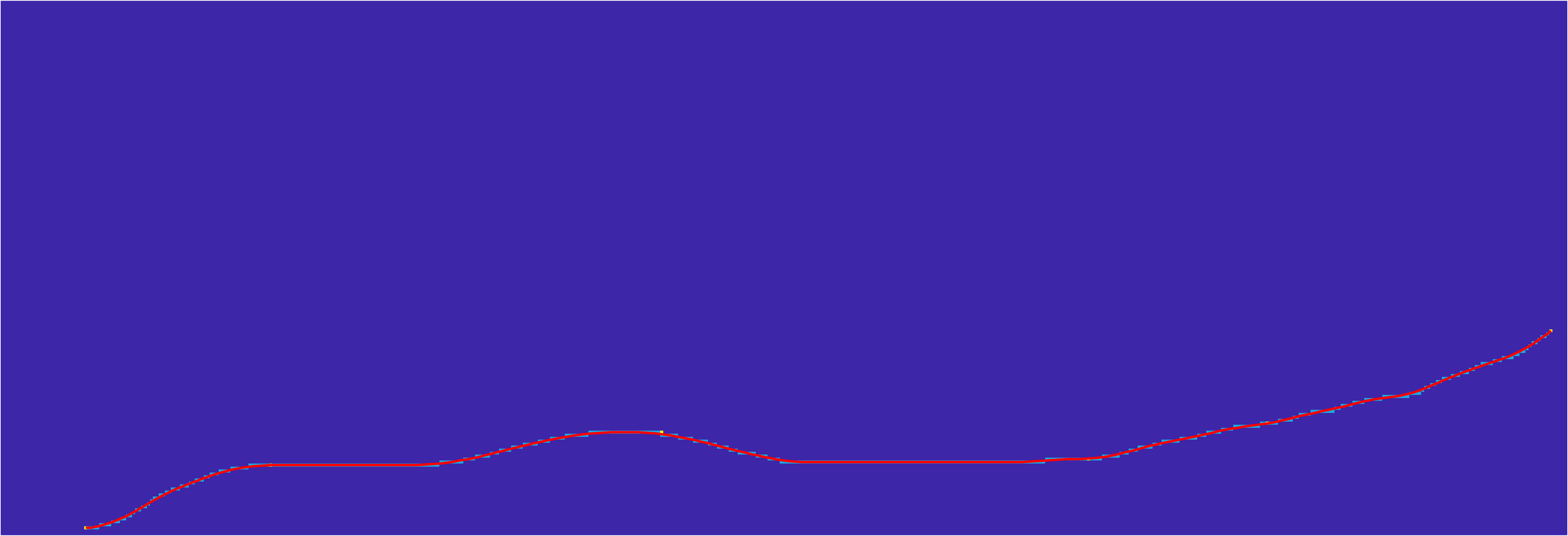

Supplement: S1 Appendix — Figures analogous to those shown in Figs. 3d, 3f, 3h, 3i, and 3j, are included. (ZIP) [file pone.0329379.s001.zip › S1 Appendix/102_Artery/i_smoothed segment_102.tif]

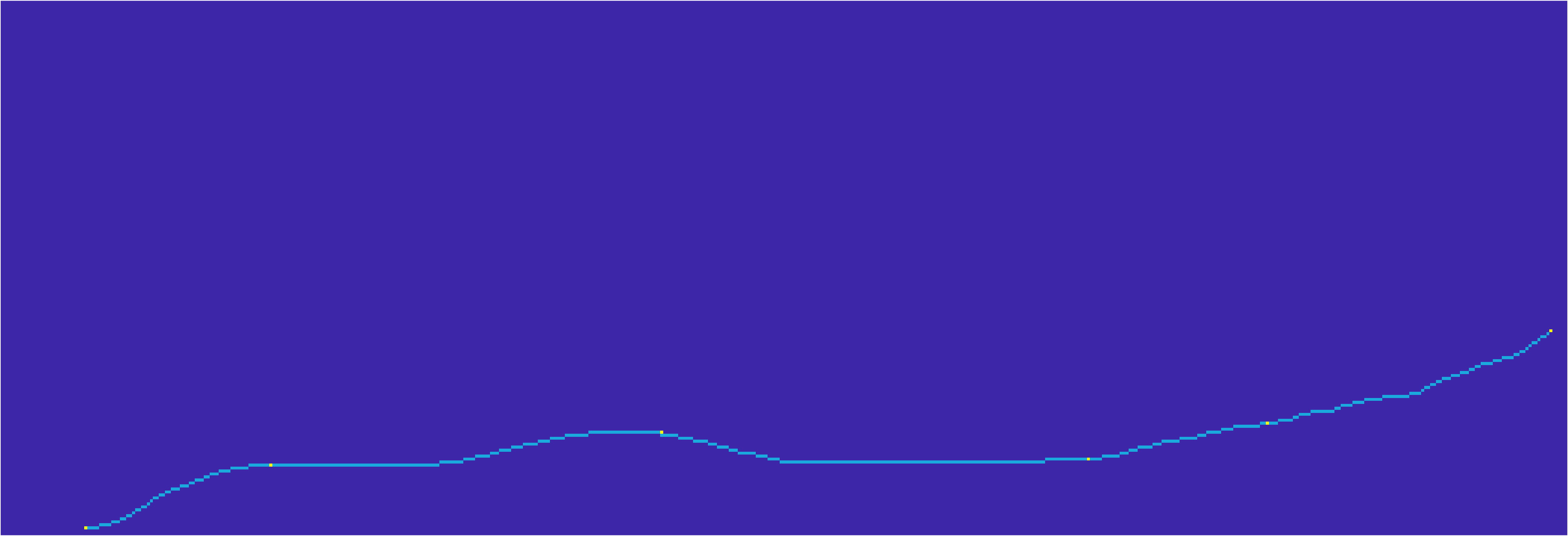

Supplement: S1 Appendix — Figures analogous to those shown in Figs. 3d, 3f, 3h, 3i, and 3j, are included. (ZIP) [file pone.0329379.s001.zip › S1 Appendix/102_Artery/h_centerline and division points_102.tif]

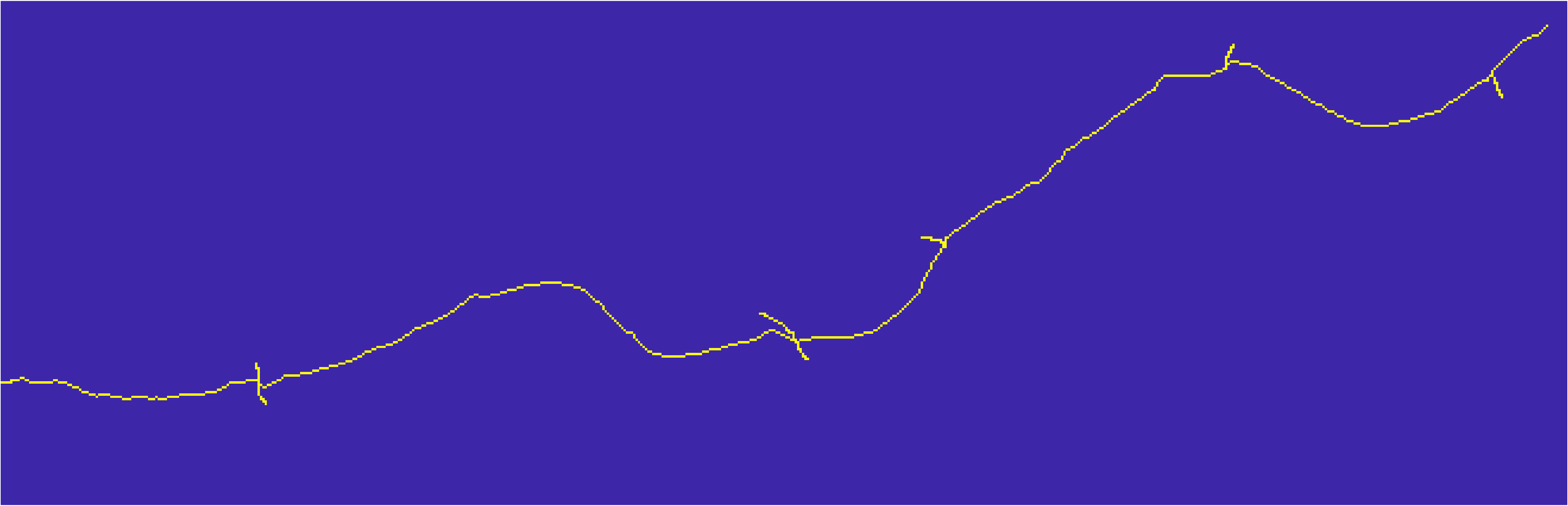

Supplement: S1 Appendix — Figures analogous to those shown in Figs. 3d, 3f, 3h, 3i, and 3j, are included. (ZIP) [file pone.0329379.s001.zip › S1 Appendix/248_Artery/f_Skeleton_248.tif]

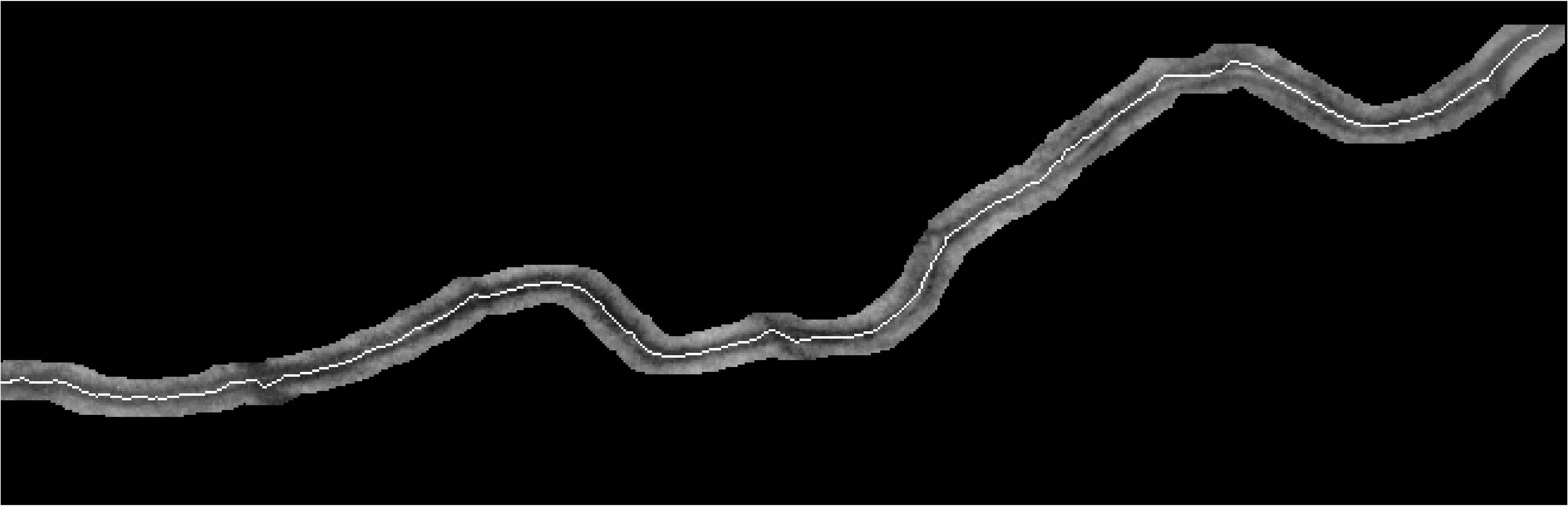

Supplement: S1 Appendix — Figures analogous to those shown in Figs. 3d, 3f, 3h, 3i, and 3j, are included. (ZIP) [file pone.0329379.s001.zip › S1 Appendix/248_Artery/d_ROI with manual trace_248.tif]

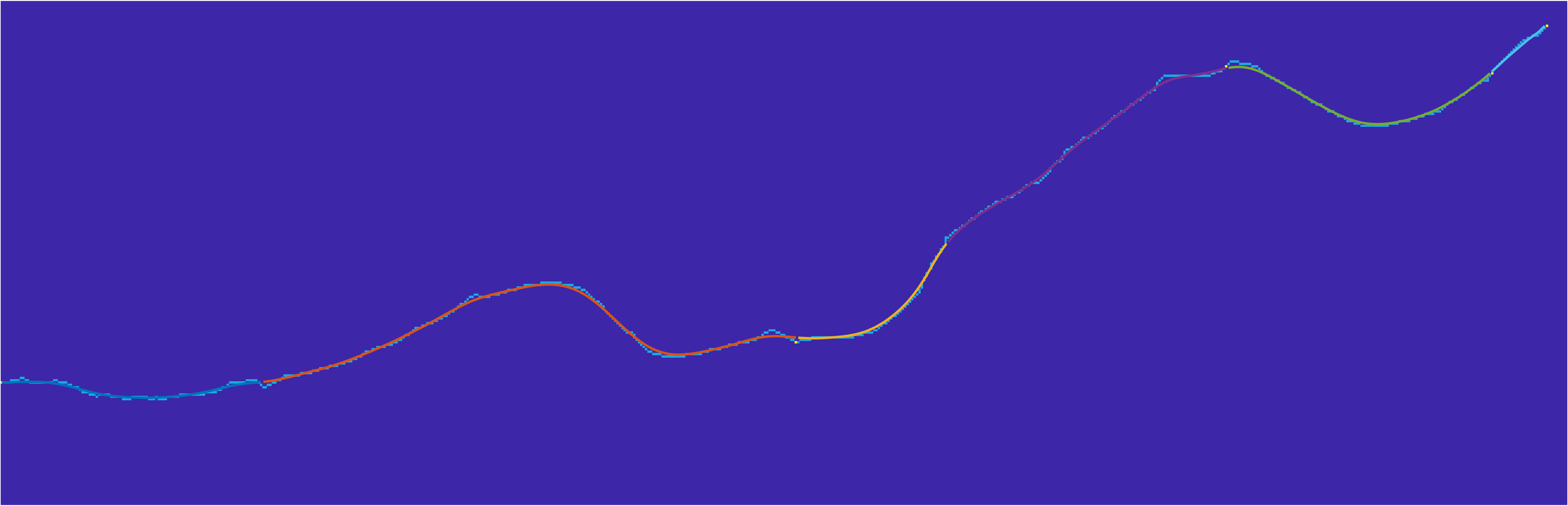

Supplement: S1 Appendix — Figures analogous to those shown in Figs. 3d, 3f, 3h, 3i, and 3j, are included. (ZIP) [file pone.0329379.s001.zip › S1 Appendix/248_Artery/j_partition_248.tif]

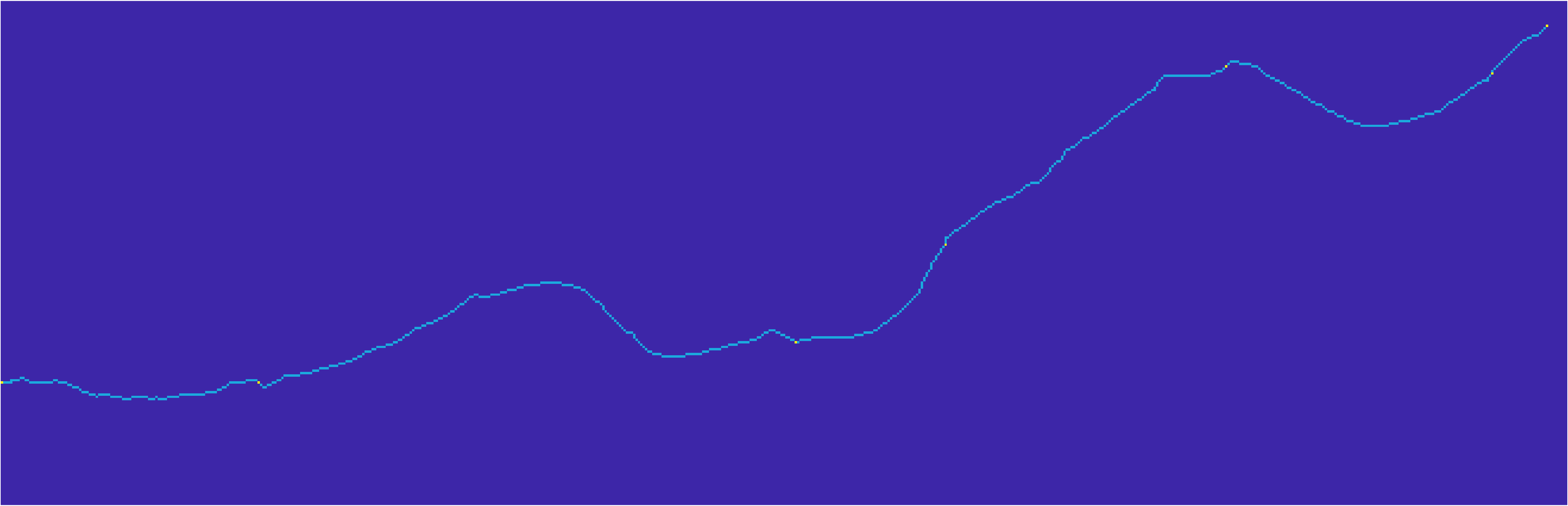

Supplement: S1 Appendix — Figures analogous to those shown in Figs. 3d, 3f, 3h, 3i, and 3j, are included. (ZIP) [file pone.0329379.s001.zip › S1 Appendix/248_Artery/h_centerline and division points_248.tif]

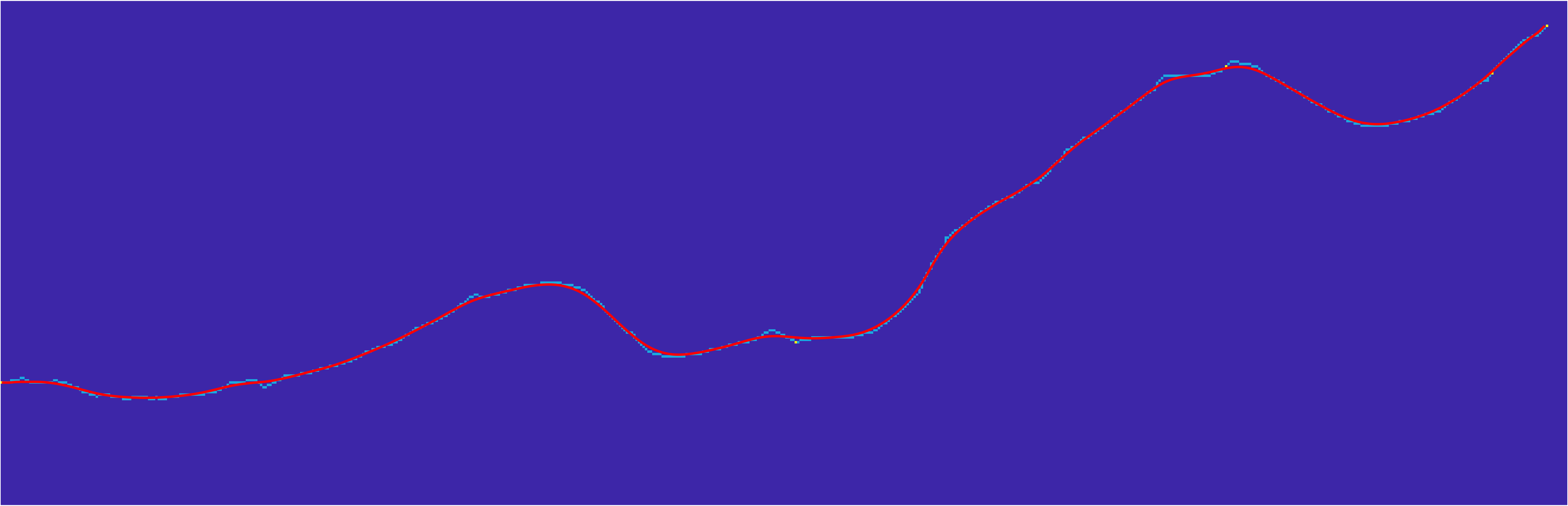

Supplement: S1 Appendix — Figures analogous to those shown in Figs. 3d, 3f, 3h, 3i, and 3j, are included. (ZIP) [file pone.0329379.s001.zip › S1 Appendix/248_Artery/i_smoothed segment_248.tif]

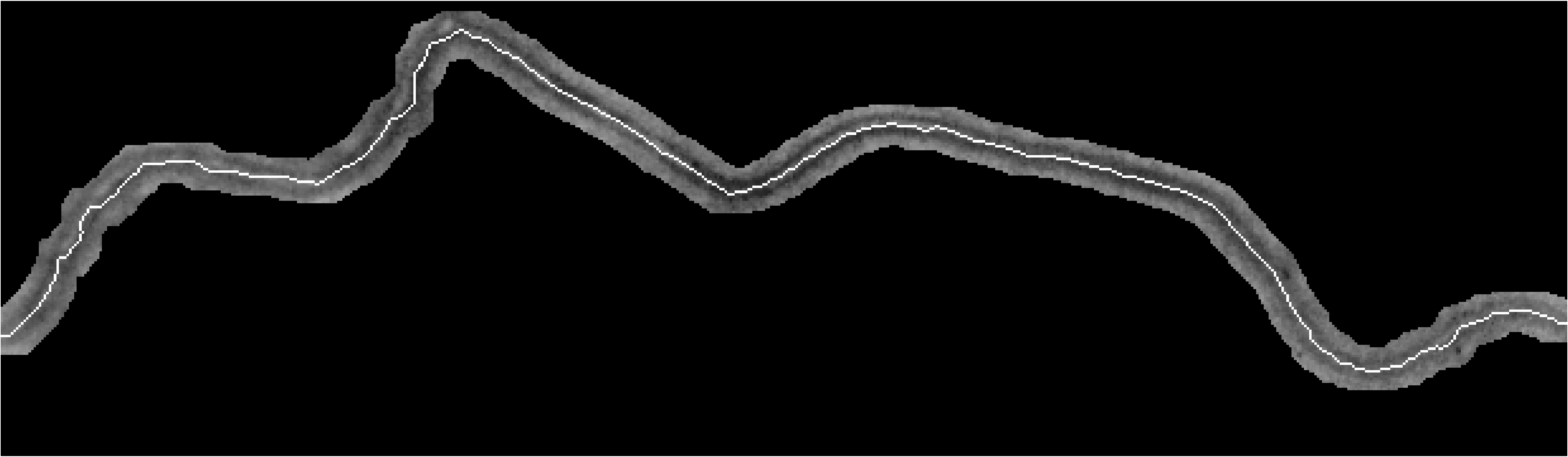

Supplement: S1 Appendix — Figures analogous to those shown in Figs. 3d, 3f, 3h, 3i, and 3j, are included. (ZIP) [file pone.0329379.s001.zip › S1 Appendix/182_Artery/d_ROI with manual trace_182.tif]

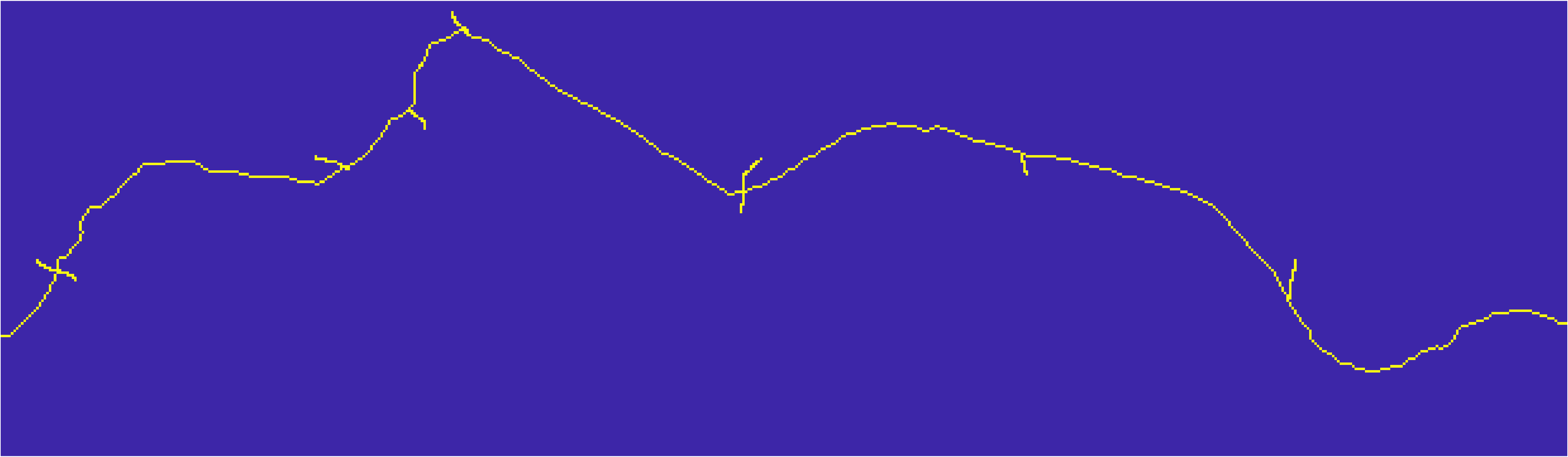

Supplement: S1 Appendix — Figures analogous to those shown in Figs. 3d, 3f, 3h, 3i, and 3j, are included. (ZIP) [file pone.0329379.s001.zip › S1 Appendix/182_Artery/f_Skeleton_182.tif]

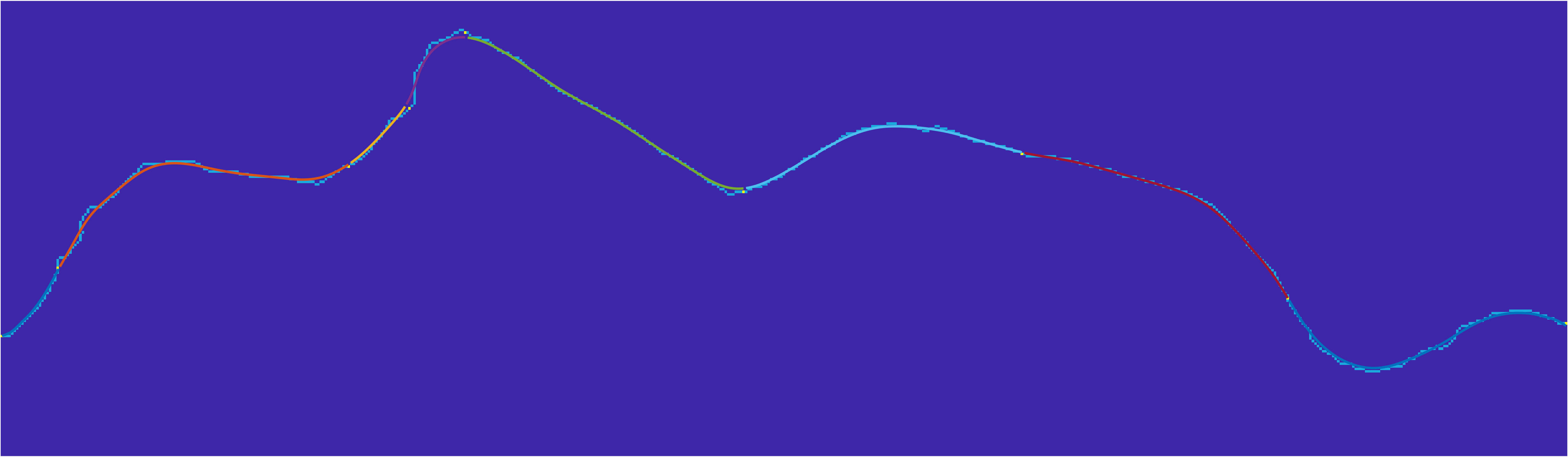

Supplement: S1 Appendix — Figures analogous to those shown in Figs. 3d, 3f, 3h, 3i, and 3j, are included. (ZIP) [file pone.0329379.s001.zip › S1 Appendix/182_Artery/j_partition_182.tif]

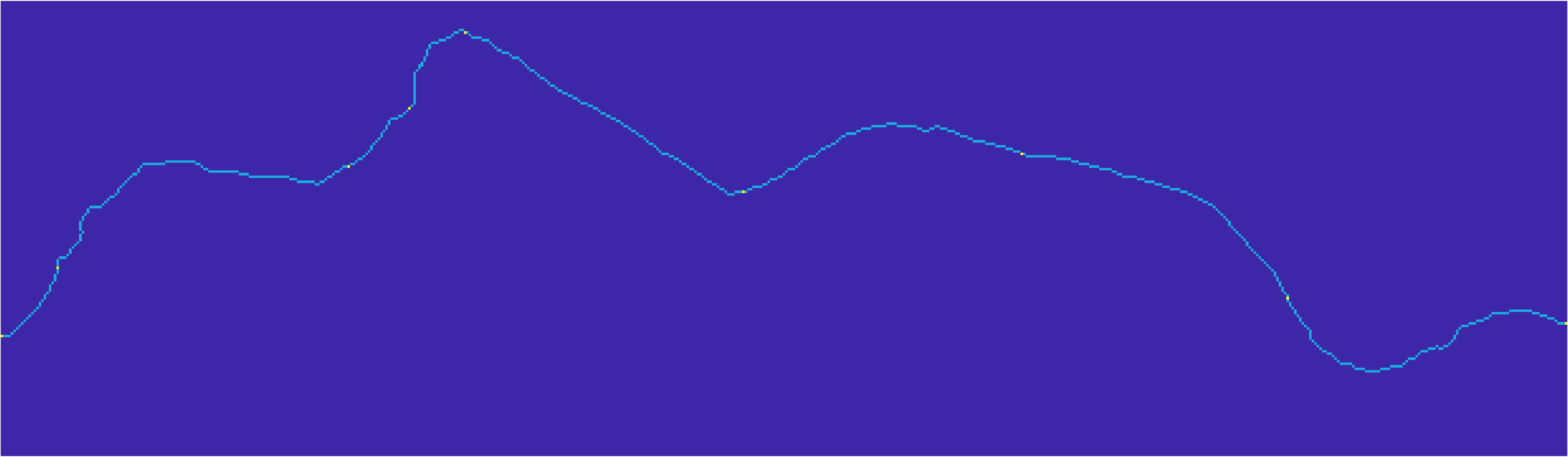

Supplement: S1 Appendix — Figures analogous to those shown in Figs. 3d, 3f, 3h, 3i, and 3j, are included. (ZIP) [file pone.0329379.s001.zip › S1 Appendix/182_Artery/h_centerline and division points_182.tif]

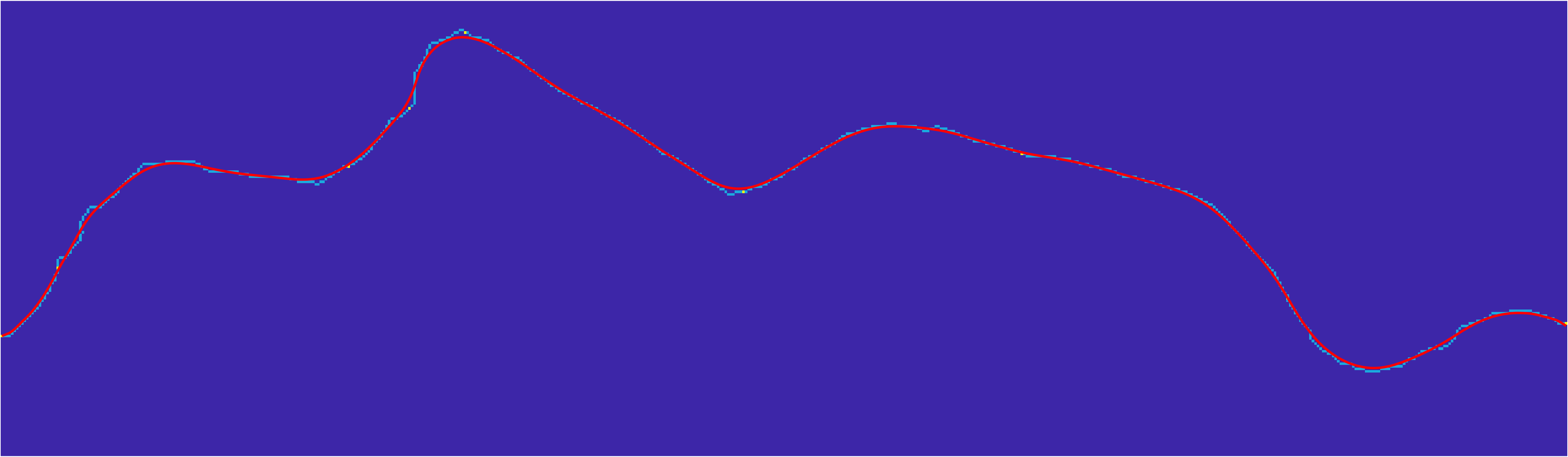

Supplement: S1 Appendix — Figures analogous to those shown in Figs. 3d, 3f, 3h, 3i, and 3j, are included. (ZIP) [file pone.0329379.s001.zip › S1 Appendix/182_Artery/i_smoothed segment_182.tif]

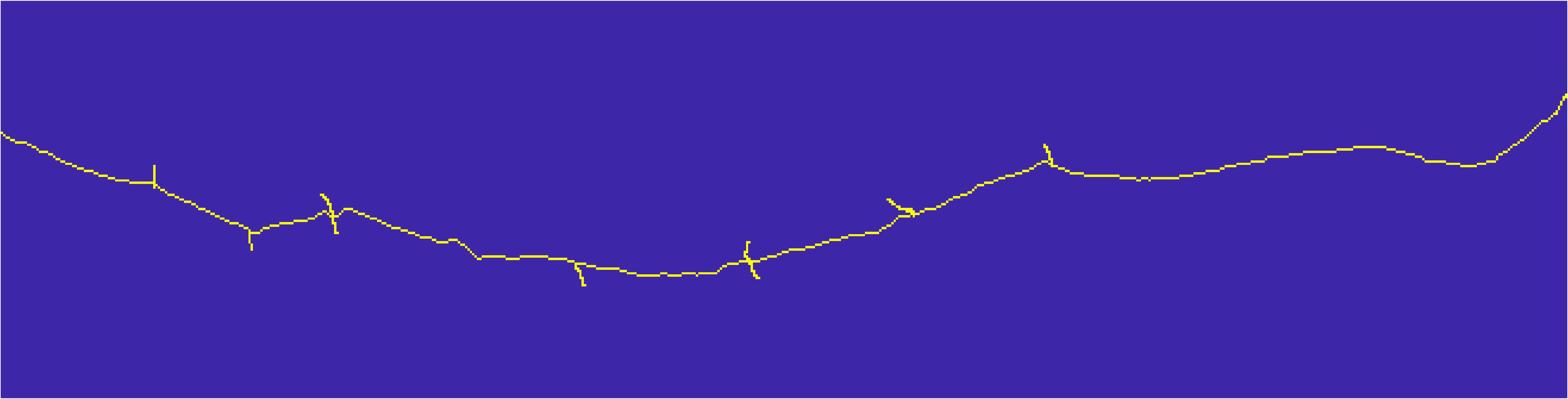

Supplement: S1 Appendix — Figures analogous to those shown in Figs. 3d, 3f, 3h, 3i, and 3j, are included. (ZIP) [file pone.0329379.s001.zip › S1 Appendix/155_Artery/f_Skeleton_155.tif]

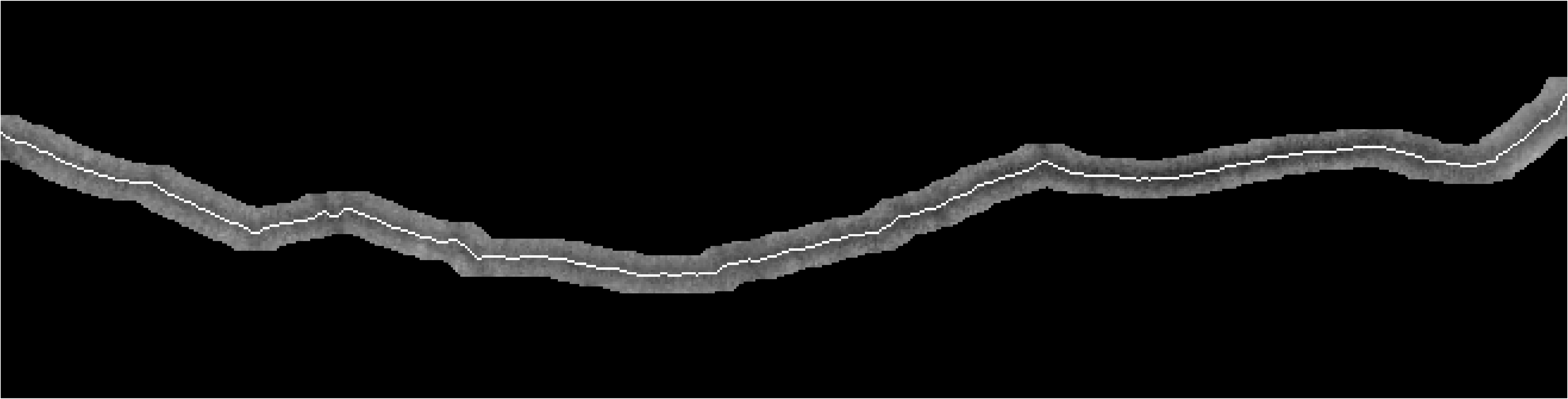

Supplement: S1 Appendix — Figures analogous to those shown in Figs. 3d, 3f, 3h, 3i, and 3j, are included. (ZIP) [file pone.0329379.s001.zip › S1 Appendix/155_Artery/d_ROI with manual trace_155.tif]

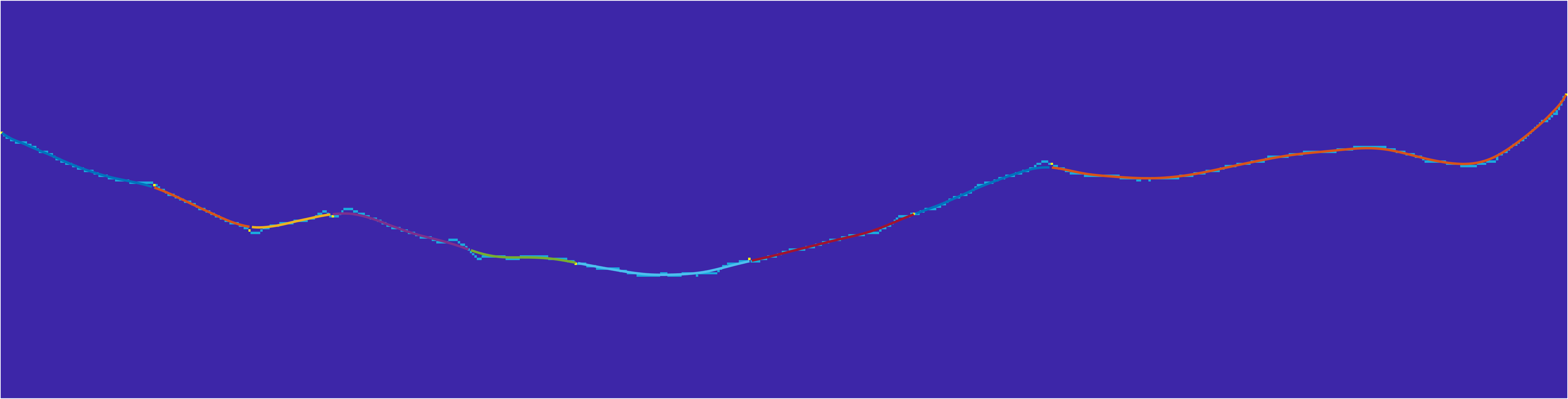

Supplement: S1 Appendix — Figures analogous to those shown in Figs. 3d, 3f, 3h, 3i, and 3j, are included. (ZIP) [file pone.0329379.s001.zip › S1 Appendix/155_Artery/j_partition_155.tif]

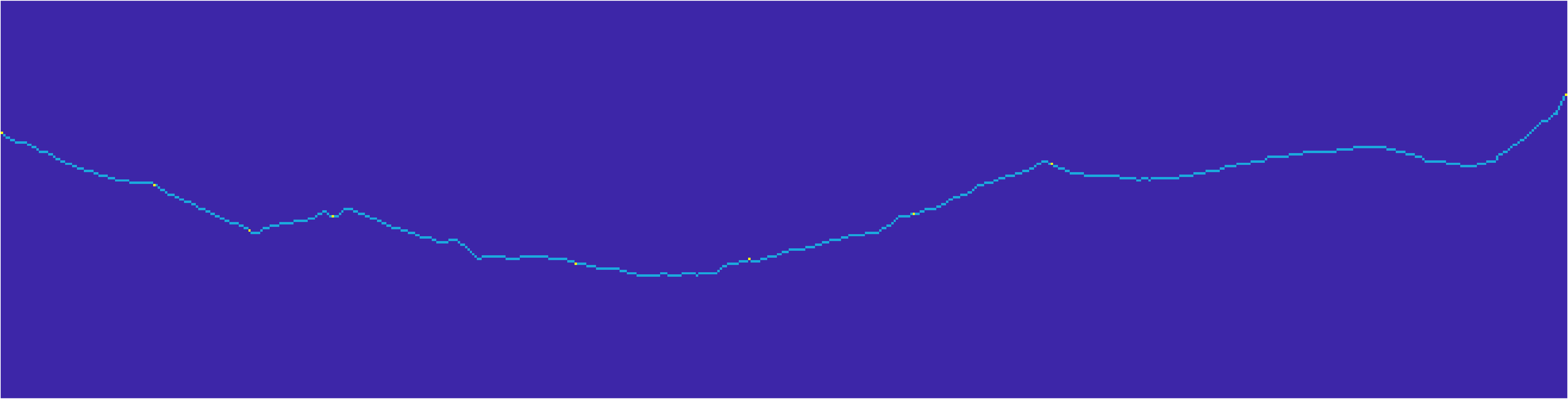

Supplement: S1 Appendix — Figures analogous to those shown in Figs. 3d, 3f, 3h, 3i, and 3j, are included. (ZIP) [file pone.0329379.s001.zip › S1 Appendix/155_Artery/h_centerline and division points_155.tif]

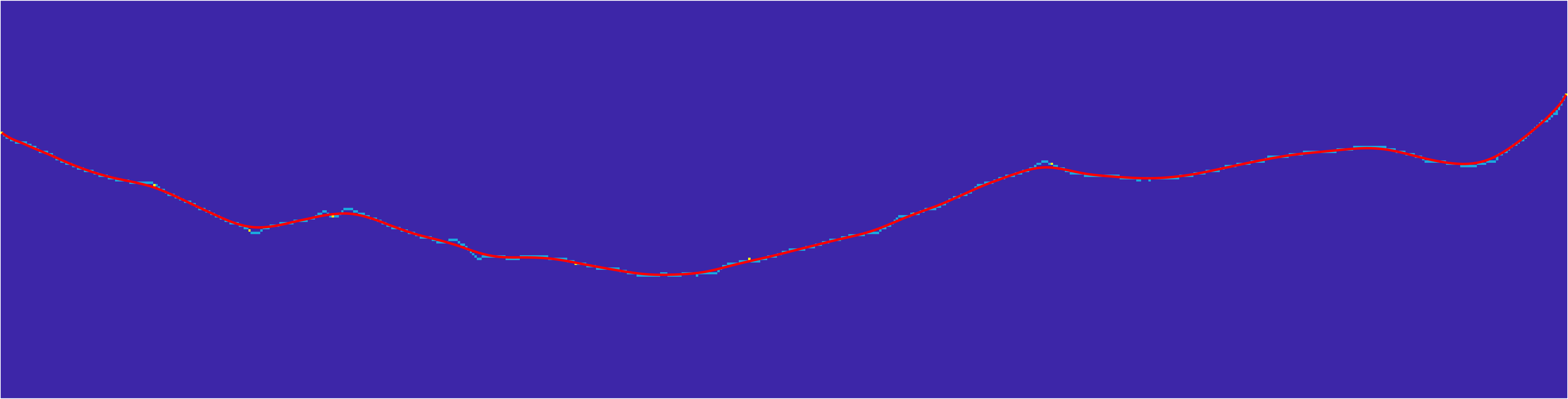

Supplement: S1 Appendix — Figures analogous to those shown in Figs. 3d, 3f, 3h, 3i, and 3j, are included. (ZIP) [file pone.0329379.s001.zip › S1 Appendix/155_Artery/i_smoothed segment_155.tif]

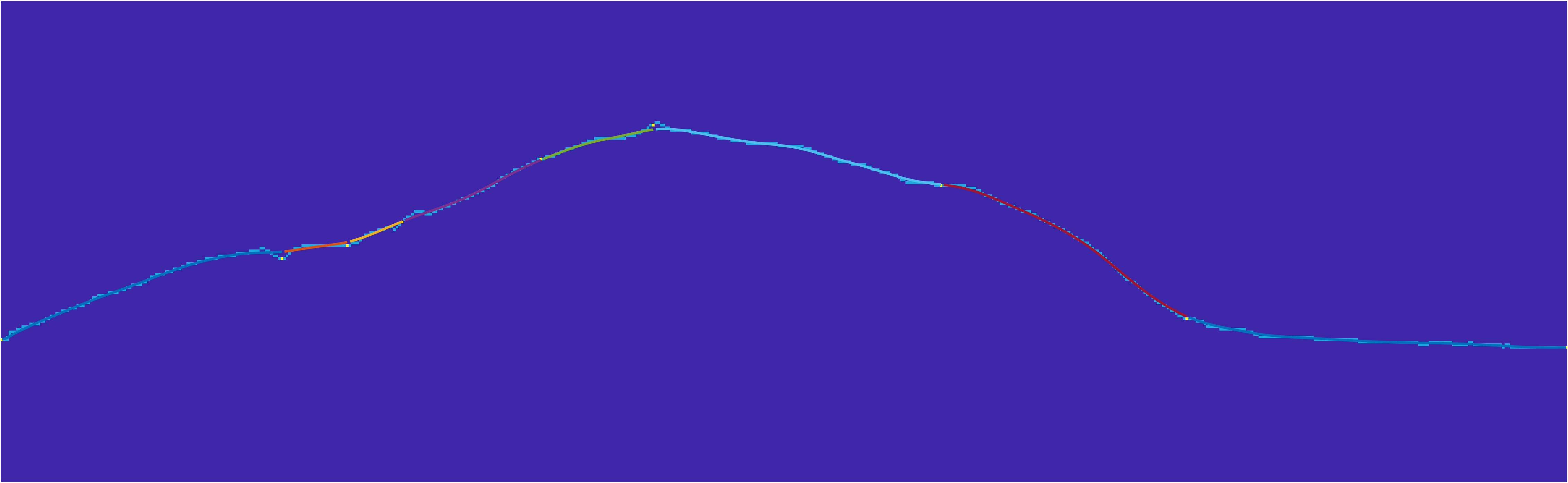

Supplement: S1 Appendix — Figures analogous to those shown in Figs. 3d, 3f, 3h, 3i, and 3j, are included. (ZIP) [file pone.0329379.s001.zip › S1 Appendix/186_Artery/j_partition_186.tif]

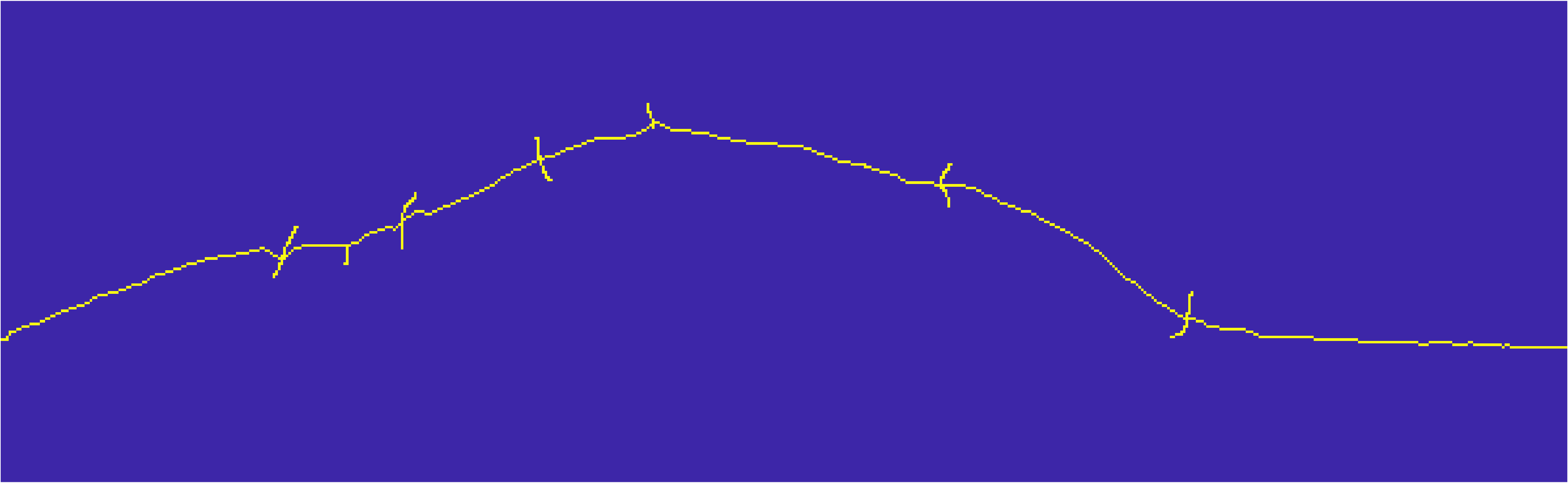

Supplement: S1 Appendix — Figures analogous to those shown in Figs. 3d, 3f, 3h, 3i, and 3j, are included. (ZIP) [file pone.0329379.s001.zip › S1 Appendix/186_Artery/f_Skeleton_186.tif]

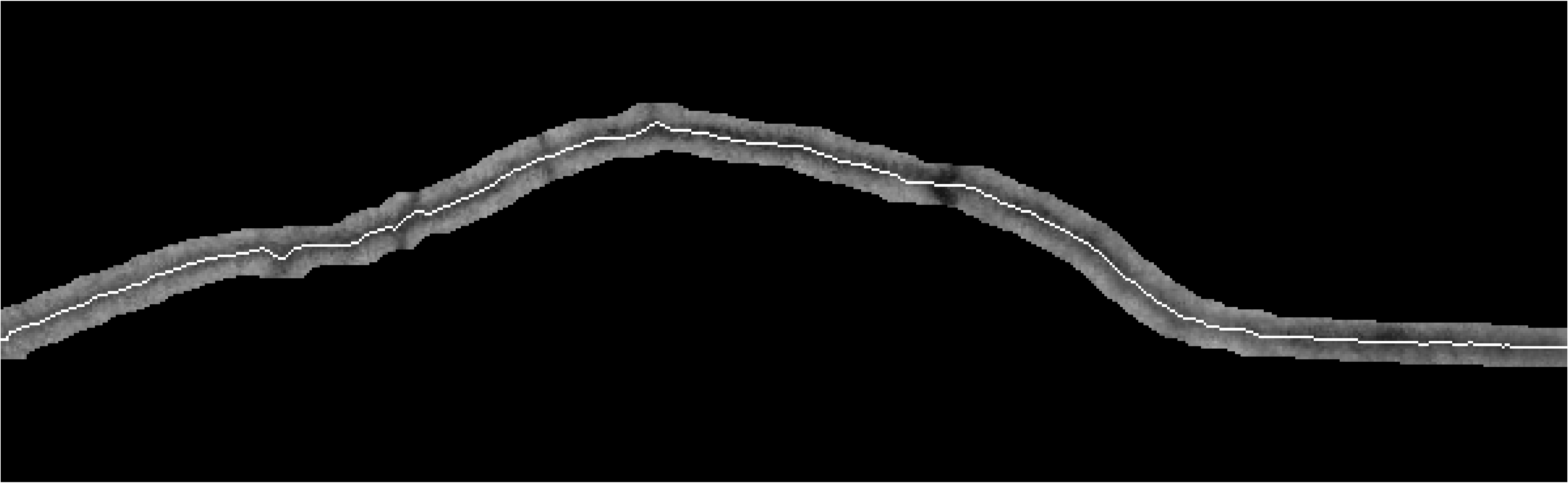

Supplement: S1 Appendix — Figures analogous to those shown in Figs. 3d, 3f, 3h, 3i, and 3j, are included. (ZIP) [file pone.0329379.s001.zip › S1 Appendix/186_Artery/d_ROI with manual trace_186.tif]

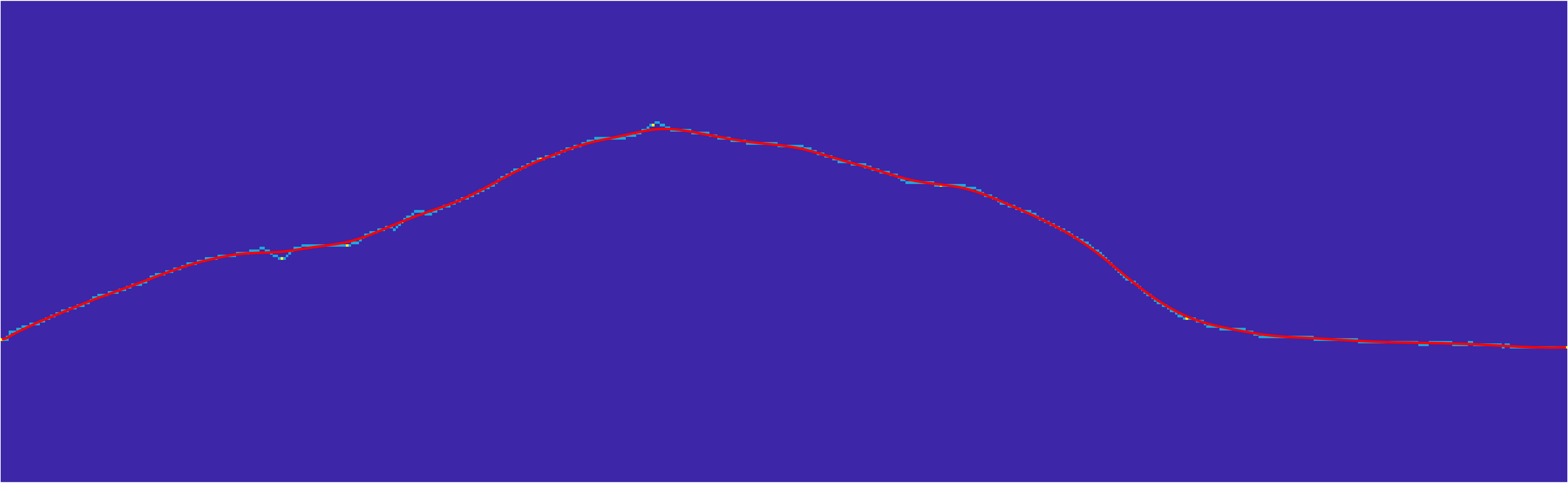

Supplement: S1 Appendix — Figures analogous to those shown in Figs. 3d, 3f, 3h, 3i, and 3j, are included. (ZIP) [file pone.0329379.s001.zip › S1 Appendix/186_Artery/i_smoothed segment_186.tif]

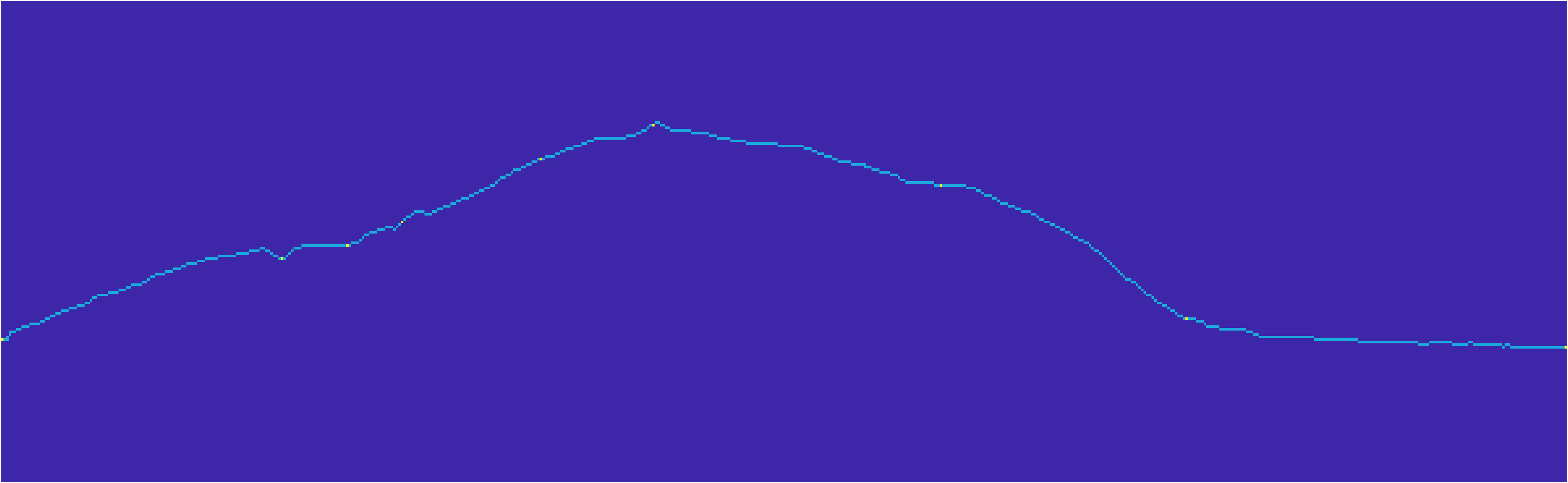

Supplement: S1 Appendix — Figures analogous to those shown in Figs. 3d, 3f, 3h, 3i, and 3j, are included. (ZIP) [file pone.0329379.s001.zip › S1 Appendix/186_Artery/h_centerline and division points_186.tif]

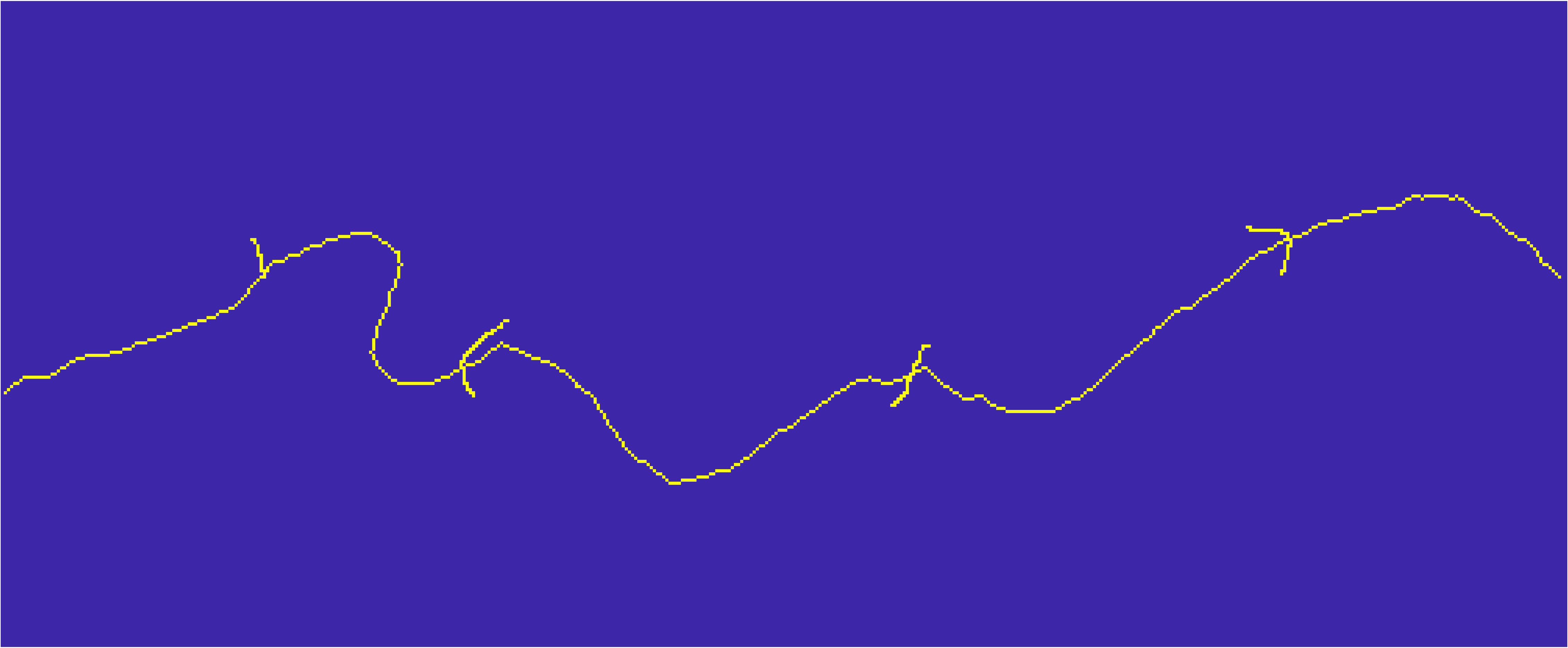

Supplement: S1 Appendix — Figures analogous to those shown in Figs. 3d, 3f, 3h, 3i, and 3j, are included. (ZIP) [file pone.0329379.s001.zip › S1 Appendix/008_Artery/f_Skeleton_008.tif]

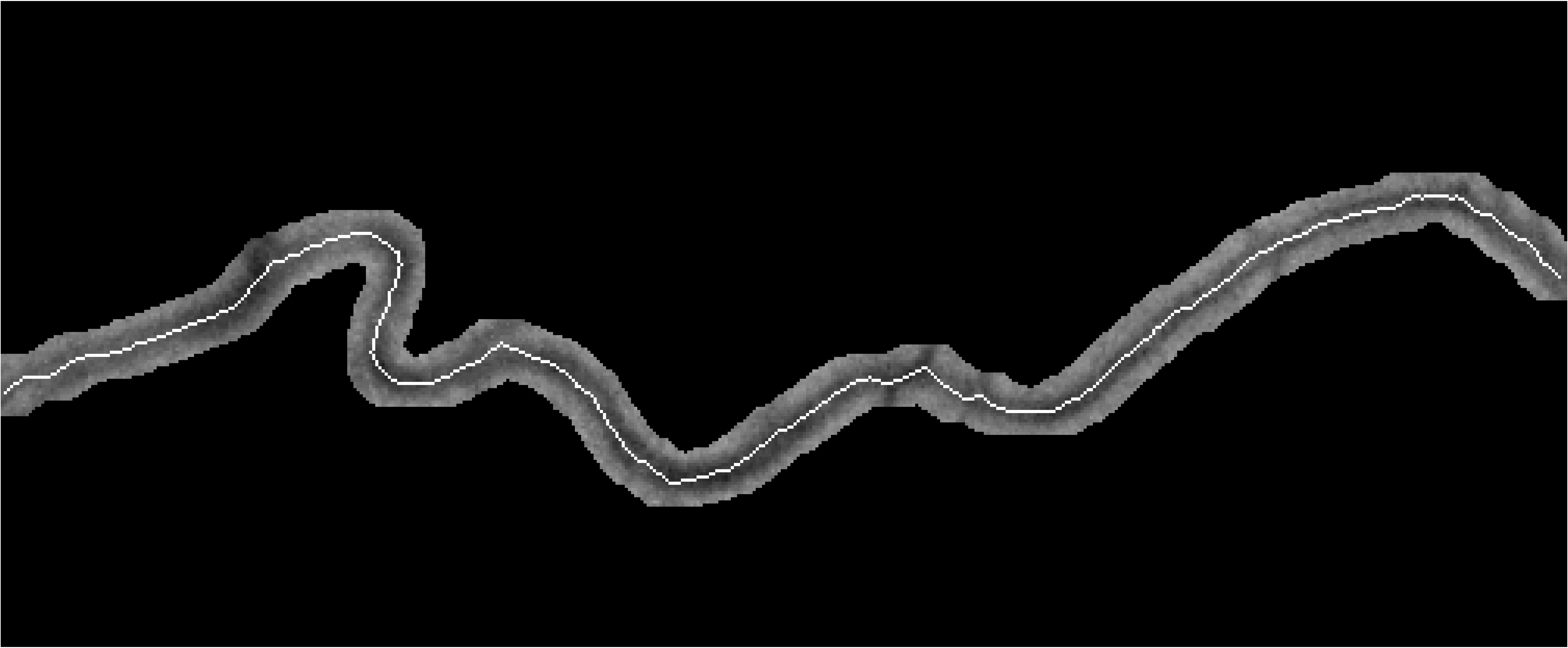

Supplement: S1 Appendix — Figures analogous to those shown in Figs. 3d, 3f, 3h, 3i, and 3j, are included. (ZIP) [file pone.0329379.s001.zip › S1 Appendix/008_Artery/d_ROI with manual trace_008.tif]

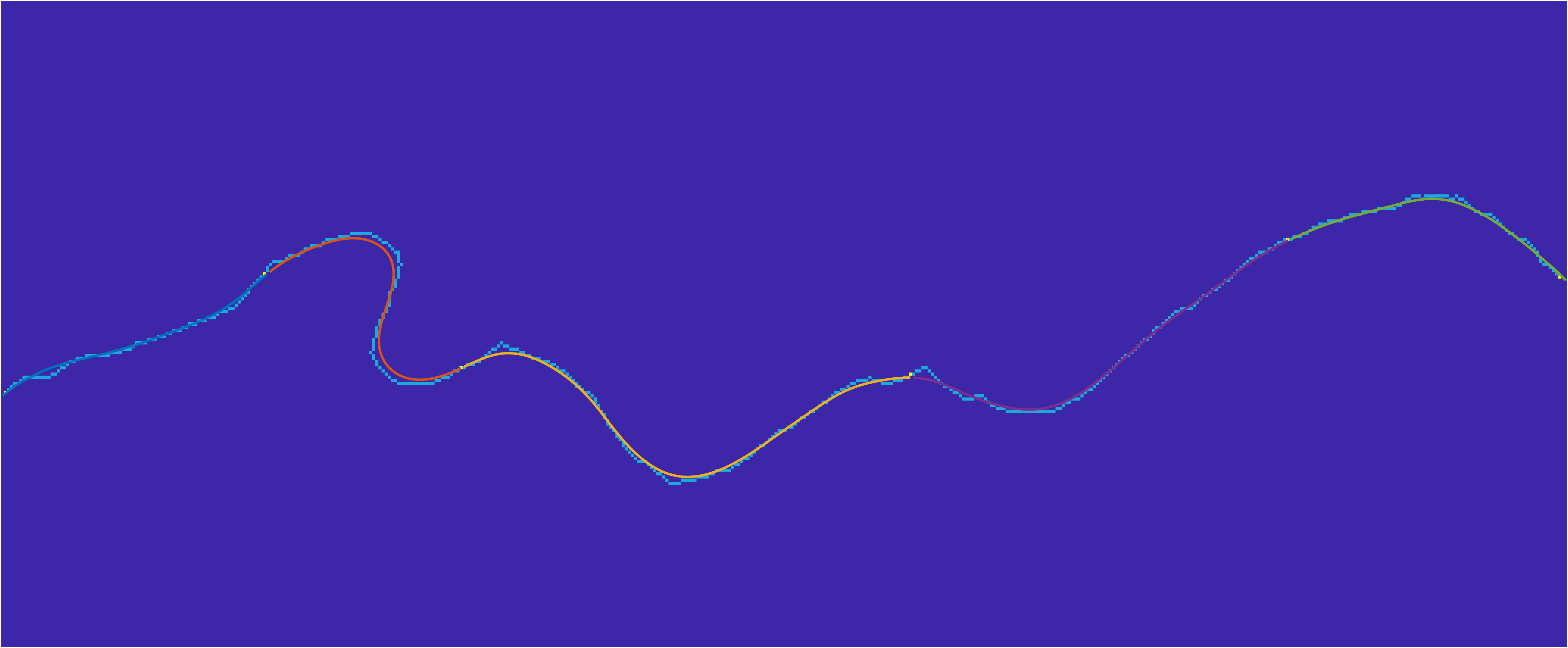

Supplement: S1 Appendix — Figures analogous to those shown in Figs. 3d, 3f, 3h, 3i, and 3j, are included. (ZIP) [file pone.0329379.s001.zip › S1 Appendix/008_Artery/j_partition_008.tif]

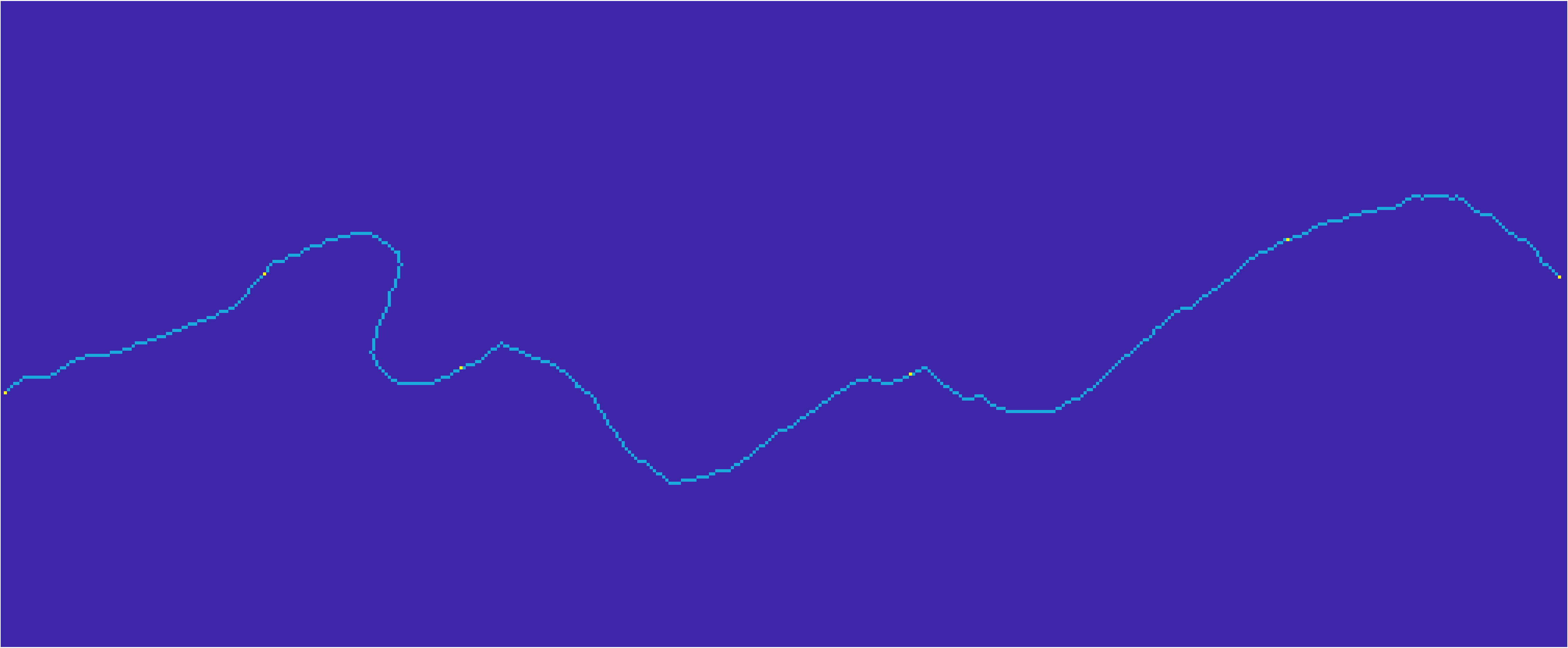

Supplement: S1 Appendix — Figures analogous to those shown in Figs. 3d, 3f, 3h, 3i, and 3j, are included. (ZIP) [file pone.0329379.s001.zip › S1 Appendix/008_Artery/h_centerline and division points_008.tif]

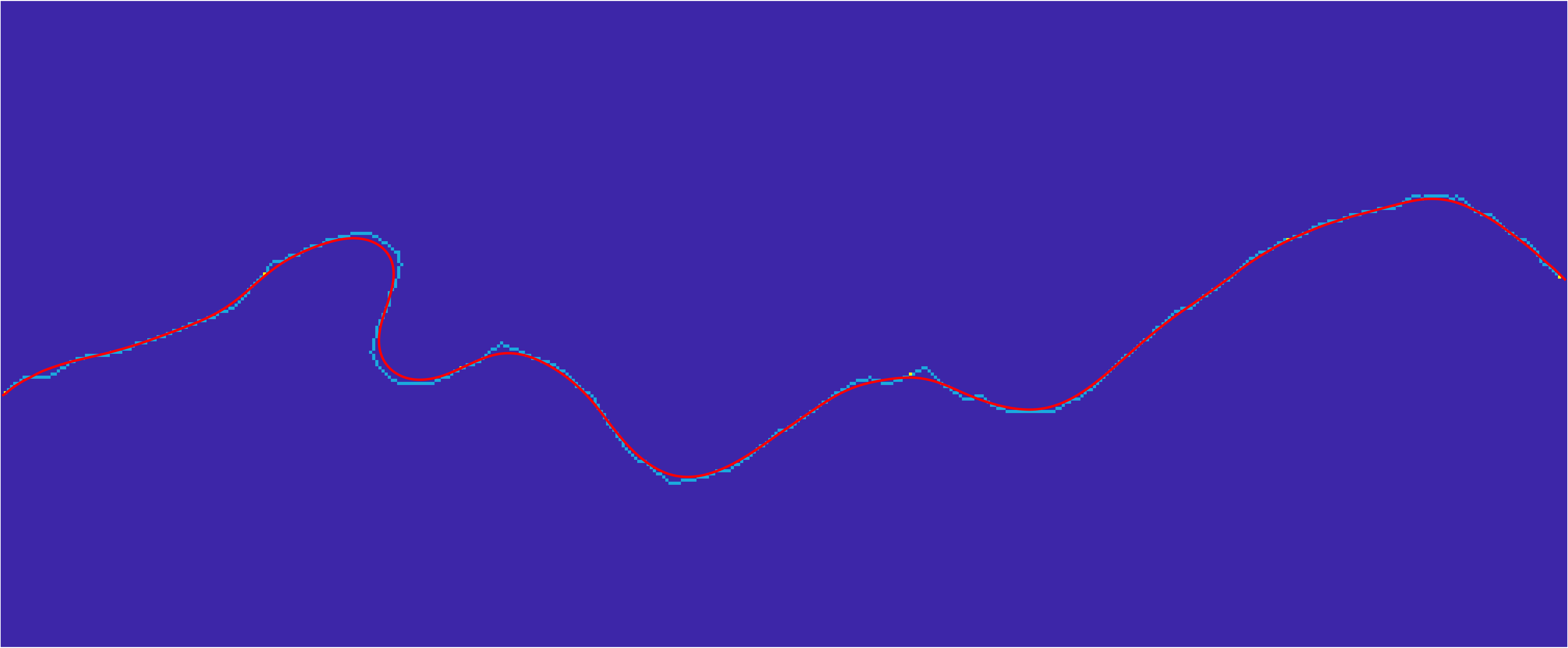

Supplement: S1 Appendix — Figures analogous to those shown in Figs. 3d, 3f, 3h, 3i, and 3j, are included. (ZIP) [file pone.0329379.s001.zip › S1 Appendix/008_Artery/i_smoothed segment_008.tif]

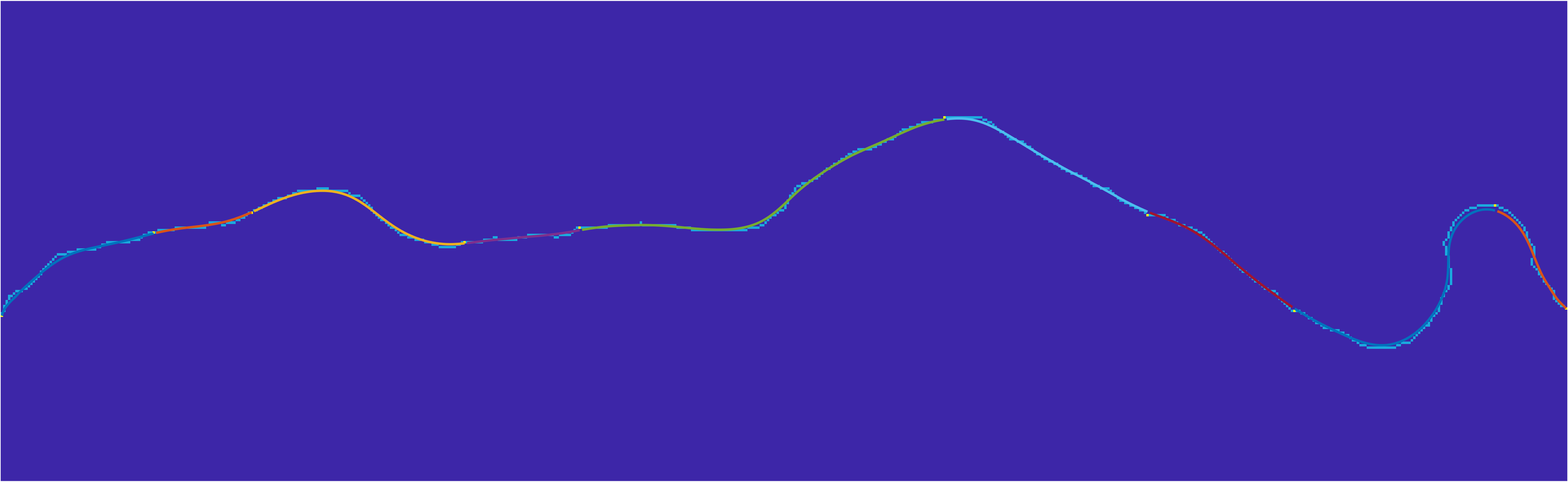

Supplement: S1 Appendix — Figures analogous to those shown in Figs. 3d, 3f, 3h, 3i, and 3j, are included. (ZIP) [file pone.0329379.s001.zip › S1 Appendix/179_Artery/j_partition_179.tif]

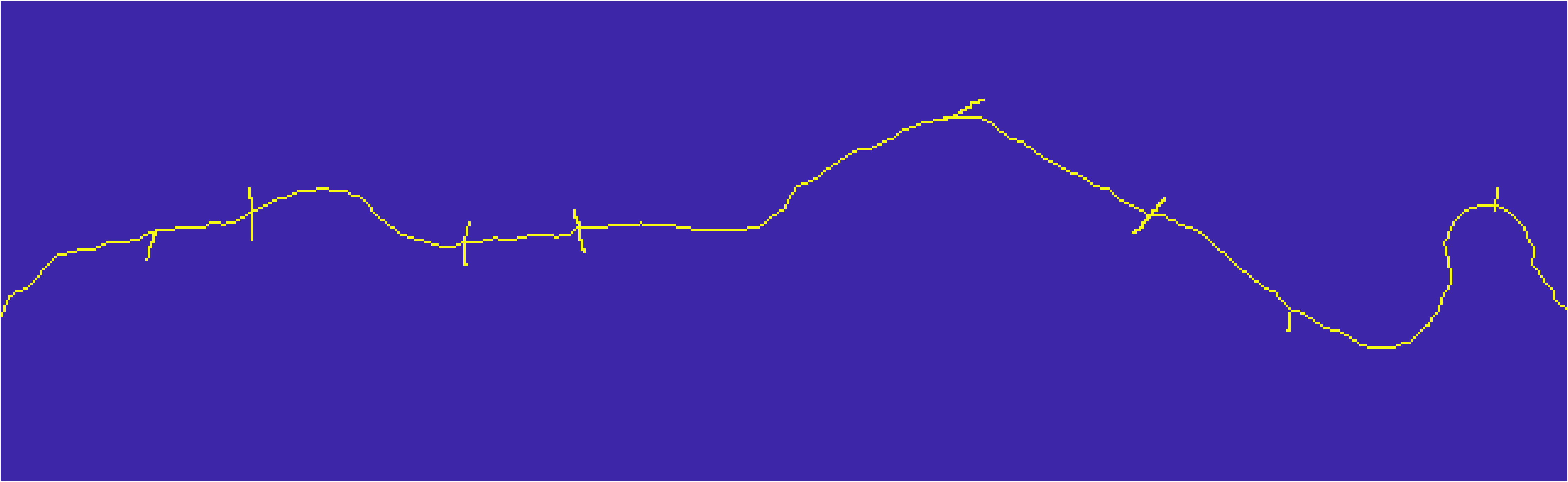

Supplement: S1 Appendix — Figures analogous to those shown in Figs. 3d, 3f, 3h, 3i, and 3j, are included. (ZIP) [file pone.0329379.s001.zip › S1 Appendix/179_Artery/f_Skeleton_179.tif]

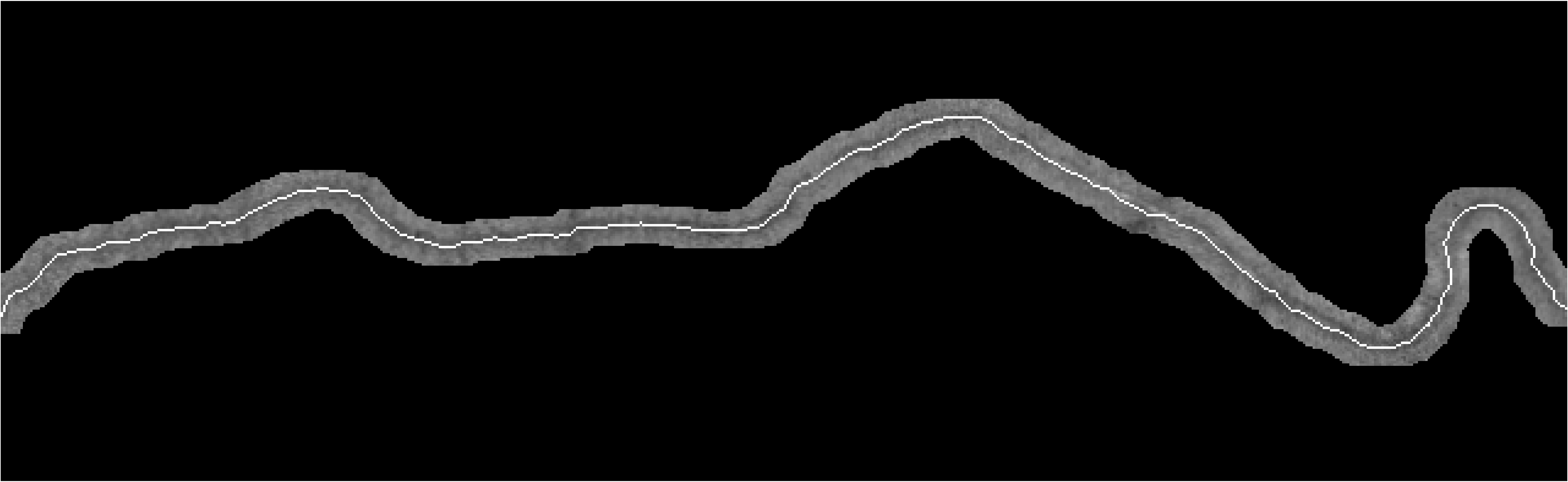

Supplement: S1 Appendix — Figures analogous to those shown in Figs. 3d, 3f, 3h, 3i, and 3j, are included. (ZIP) [file pone.0329379.s001.zip › S1 Appendix/179_Artery/d_ROI with manual trace_179.tif]

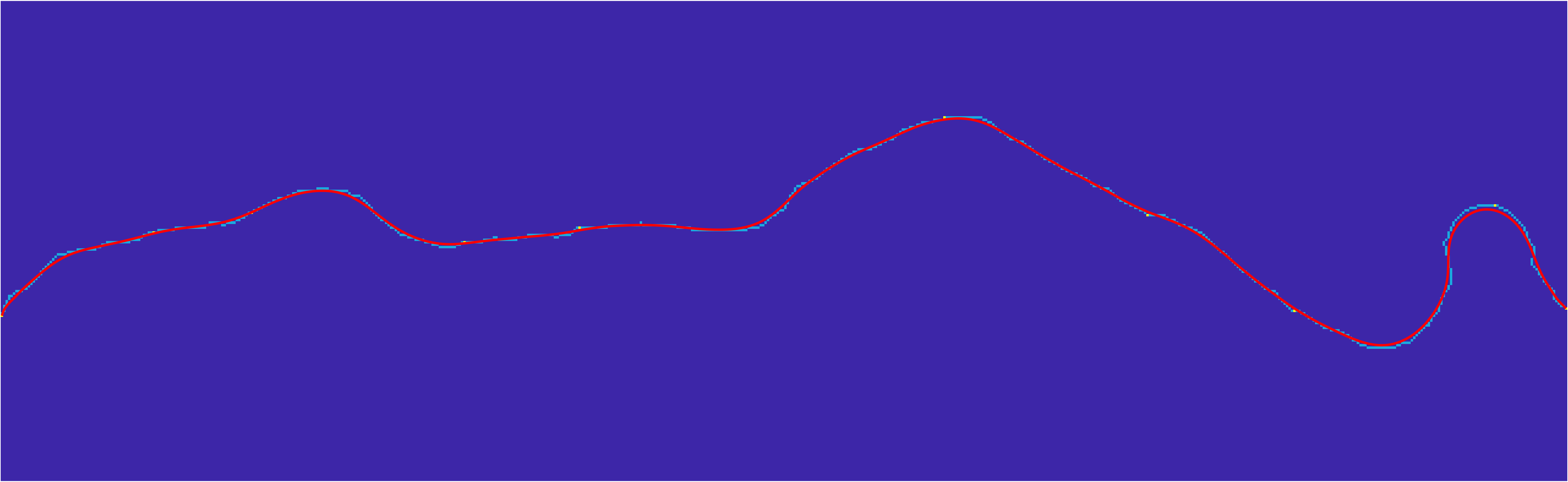

Supplement: S1 Appendix — Figures analogous to those shown in Figs. 3d, 3f, 3h, 3i, and 3j, are included. (ZIP) [file pone.0329379.s001.zip › S1 Appendix/179_Artery/i_smoothed segment_179.tif]

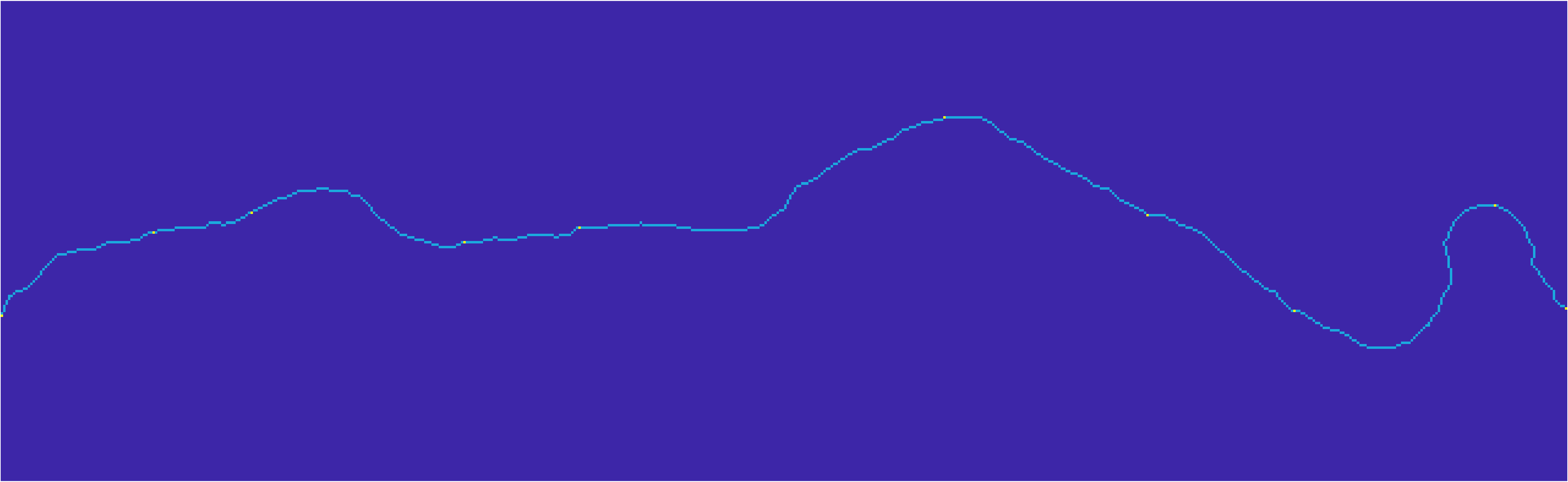

Supplement: S1 Appendix — Figures analogous to those shown in Figs. 3d, 3f, 3h, 3i, and 3j, are included. (ZIP) [file pone.0329379.s001.zip › S1 Appendix/179_Artery/h_centerline and division points_179.tif]

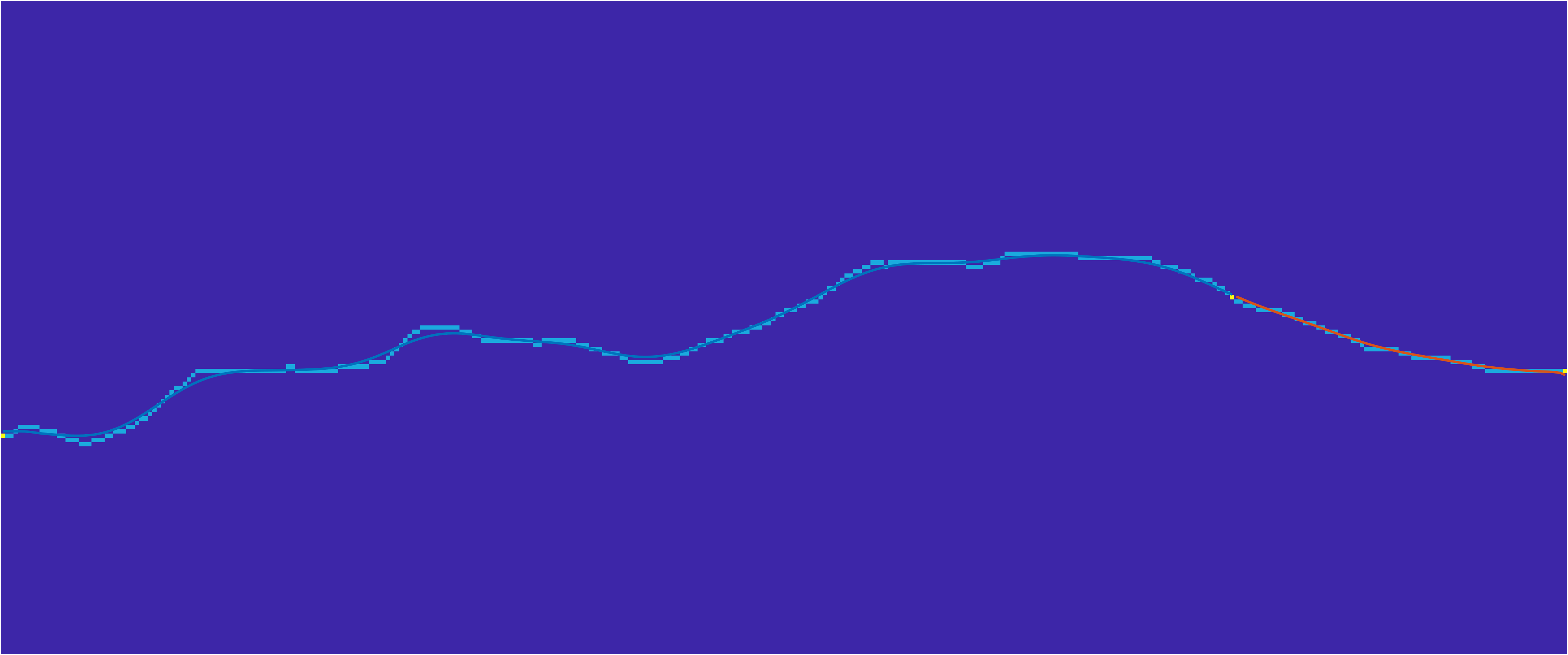

Supplement: S1 Appendix — Figures analogous to those shown in Figs. 3d, 3f, 3h, 3i, and 3j, are included. (ZIP) [file pone.0329379.s001.zip › S1 Appendix/032_Artery/j_partition_032.tif]

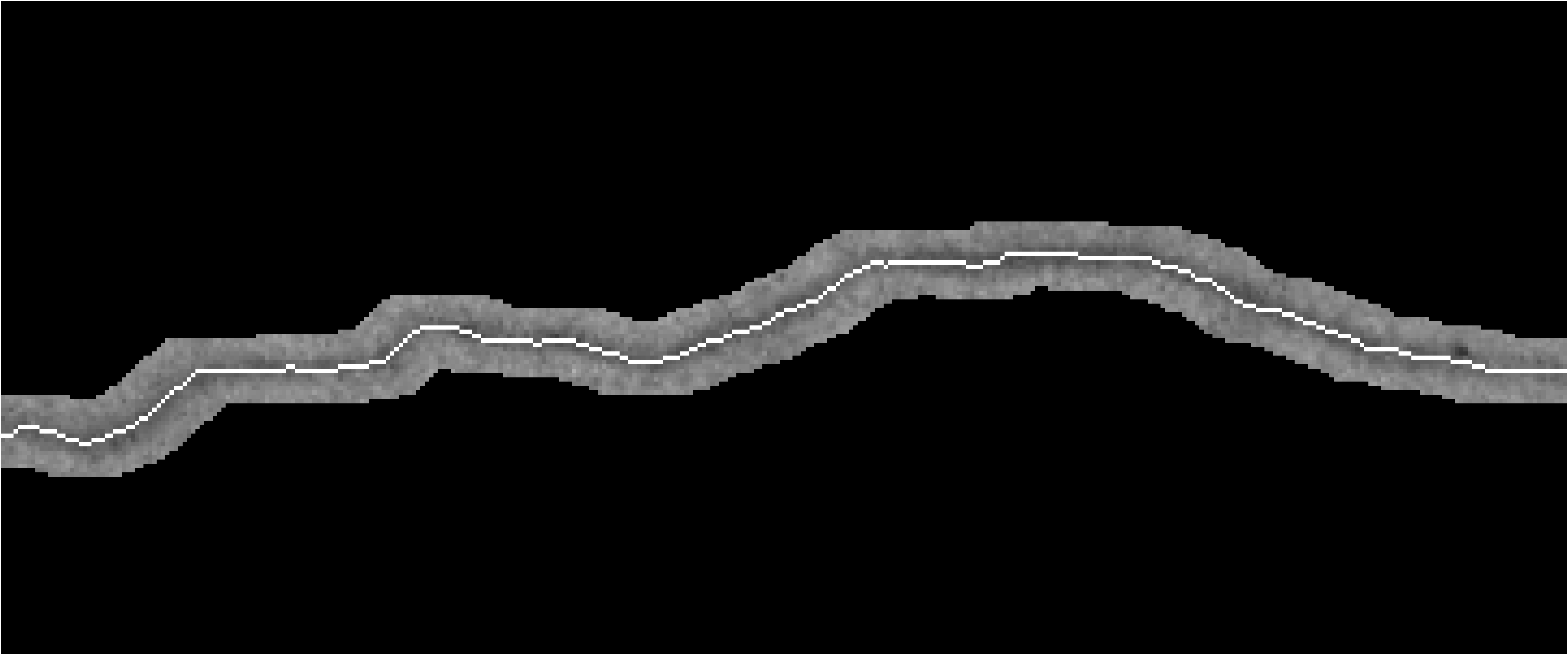

Supplement: S1 Appendix — Figures analogous to those shown in Figs. 3d, 3f, 3h, 3i, and 3j, are included. (ZIP) [file pone.0329379.s001.zip › S1 Appendix/032_Artery/d_ROI with manual trace_032.tif]

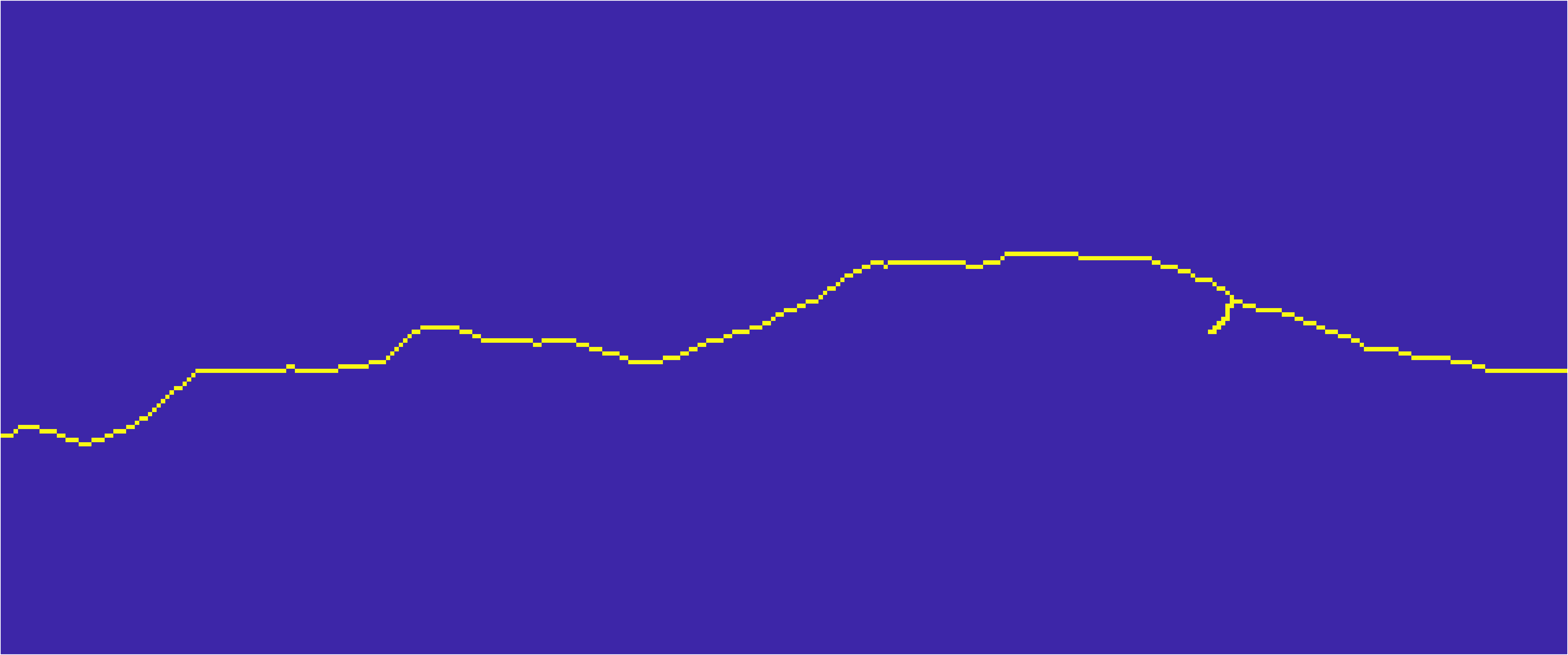

Supplement: S1 Appendix — Figures analogous to those shown in Figs. 3d, 3f, 3h, 3i, and 3j, are included. (ZIP) [file pone.0329379.s001.zip › S1 Appendix/032_Artery/f_Skeleton_032.tif]

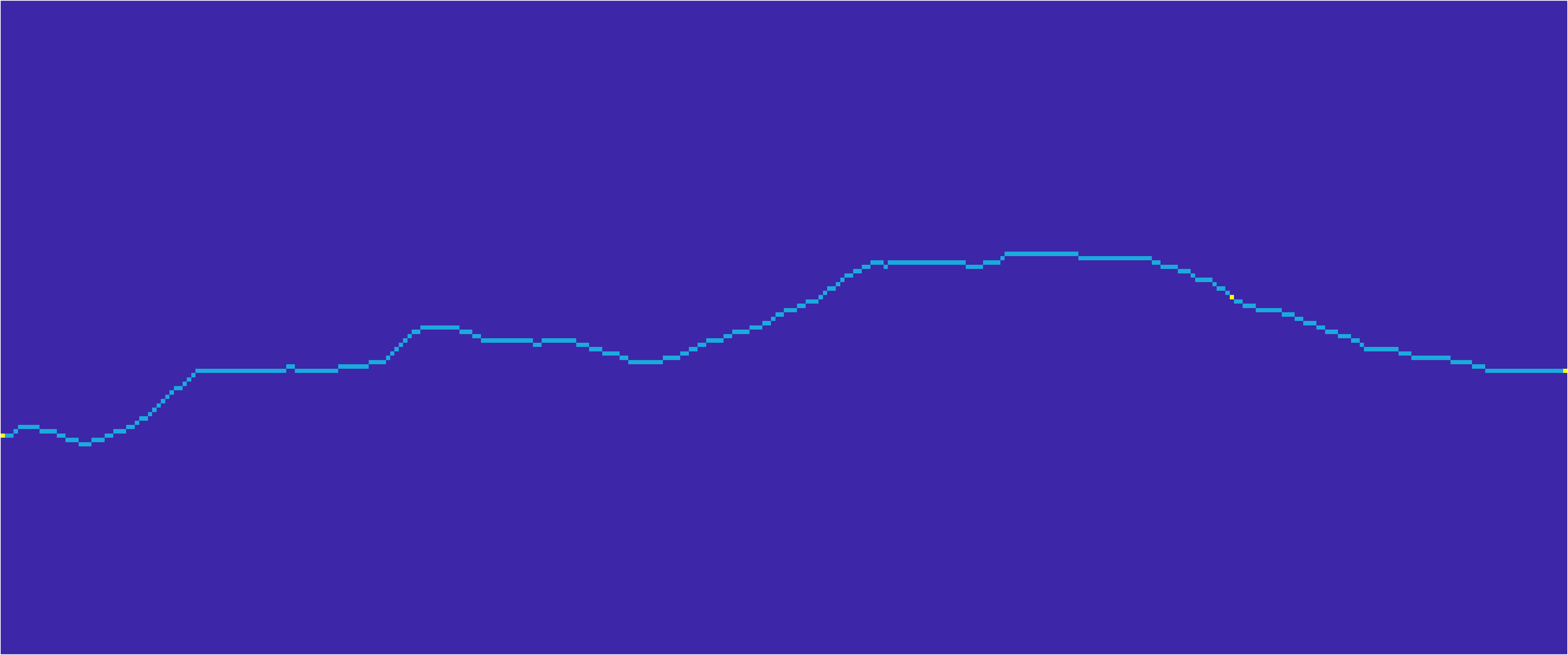

Supplement: S1 Appendix — Figures analogous to those shown in Figs. 3d, 3f, 3h, 3i, and 3j, are included. (ZIP) [file pone.0329379.s001.zip › S1 Appendix/032_Artery/h_centerline and division points_032.tif]

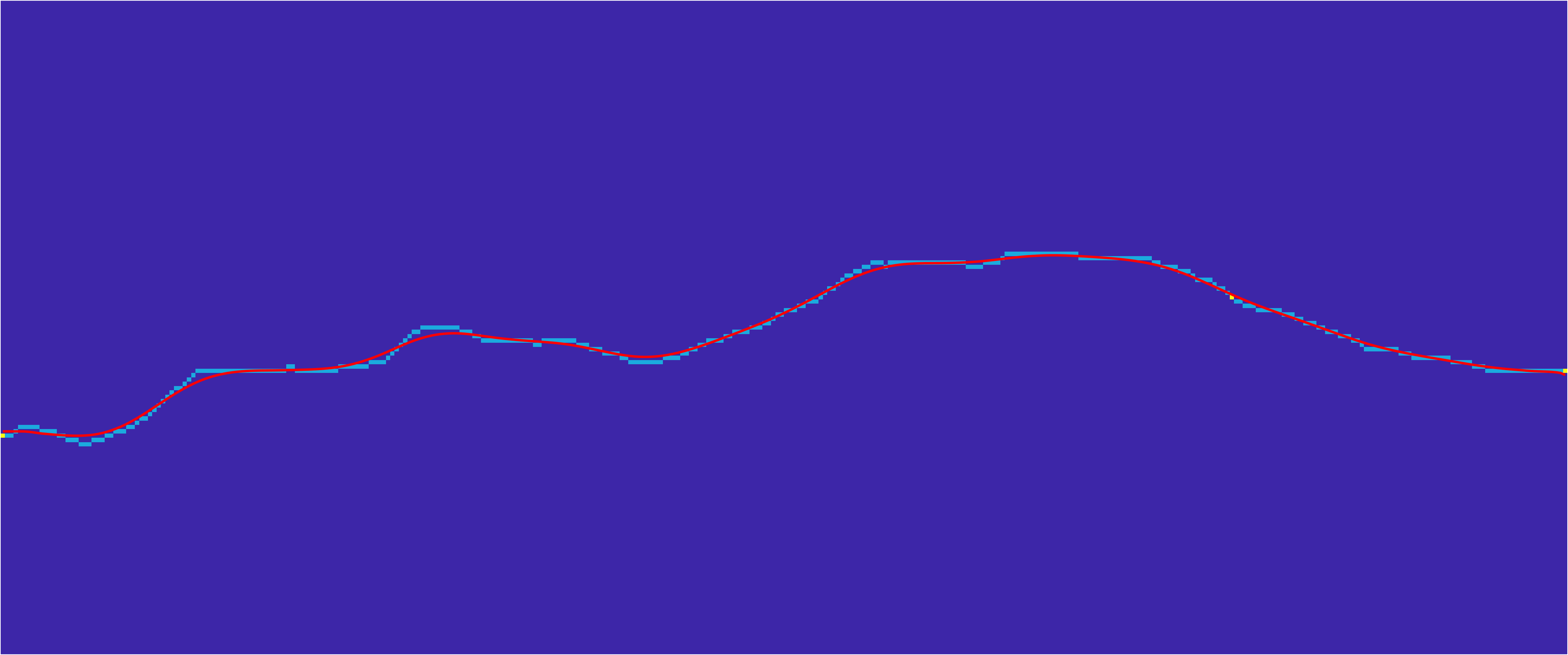

Supplement: S1 Appendix — Figures analogous to those shown in Figs. 3d, 3f, 3h, 3i, and 3j, are included. (ZIP) [file pone.0329379.s001.zip › S1 Appendix/032_Artery/i_smoothed segment_032.tif]

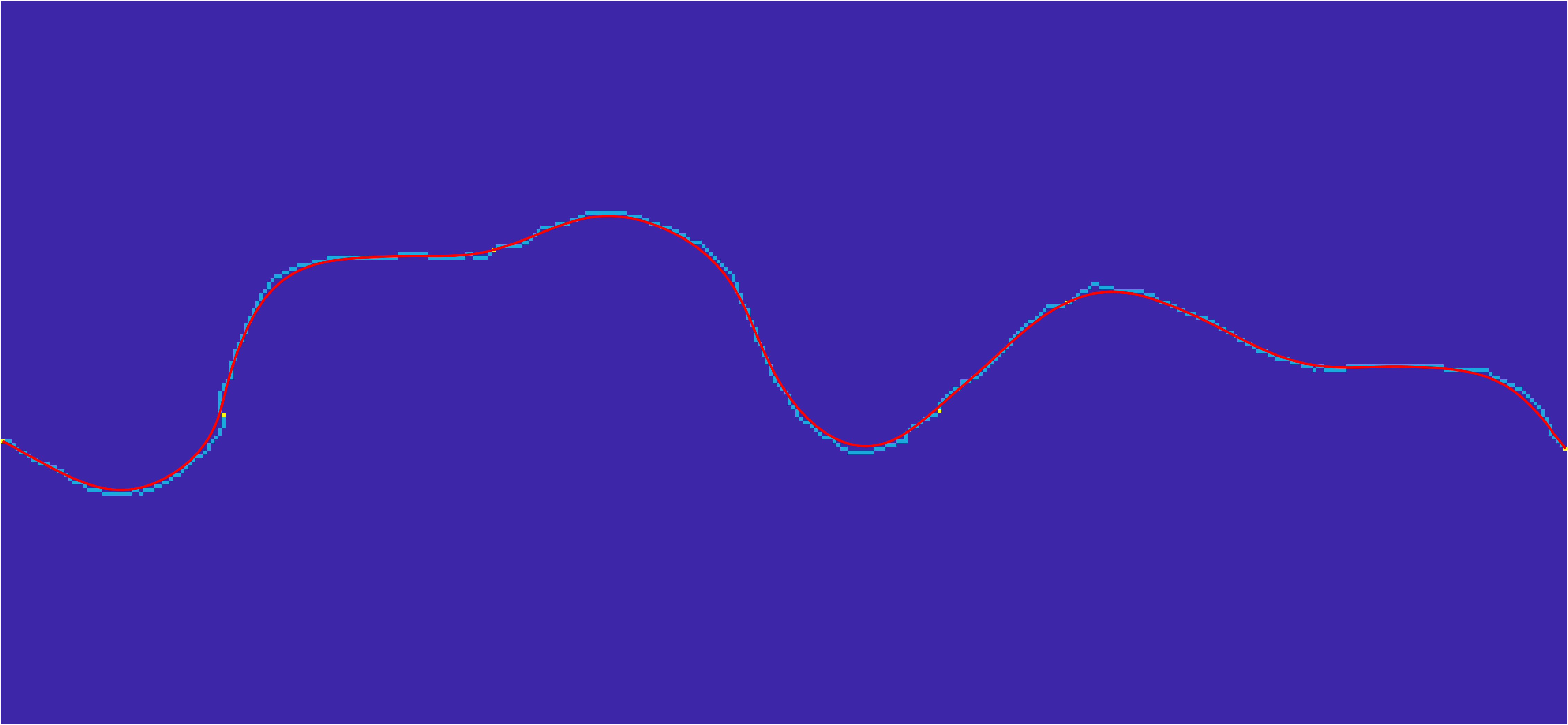

Supplement: S1 Appendix — Figures analogous to those shown in Figs. 3d, 3f, 3h, 3i, and 3j, are included. (ZIP) [file pone.0329379.s001.zip › S1 Appendix/014_Artery/i_smoothed segment_014.tif]

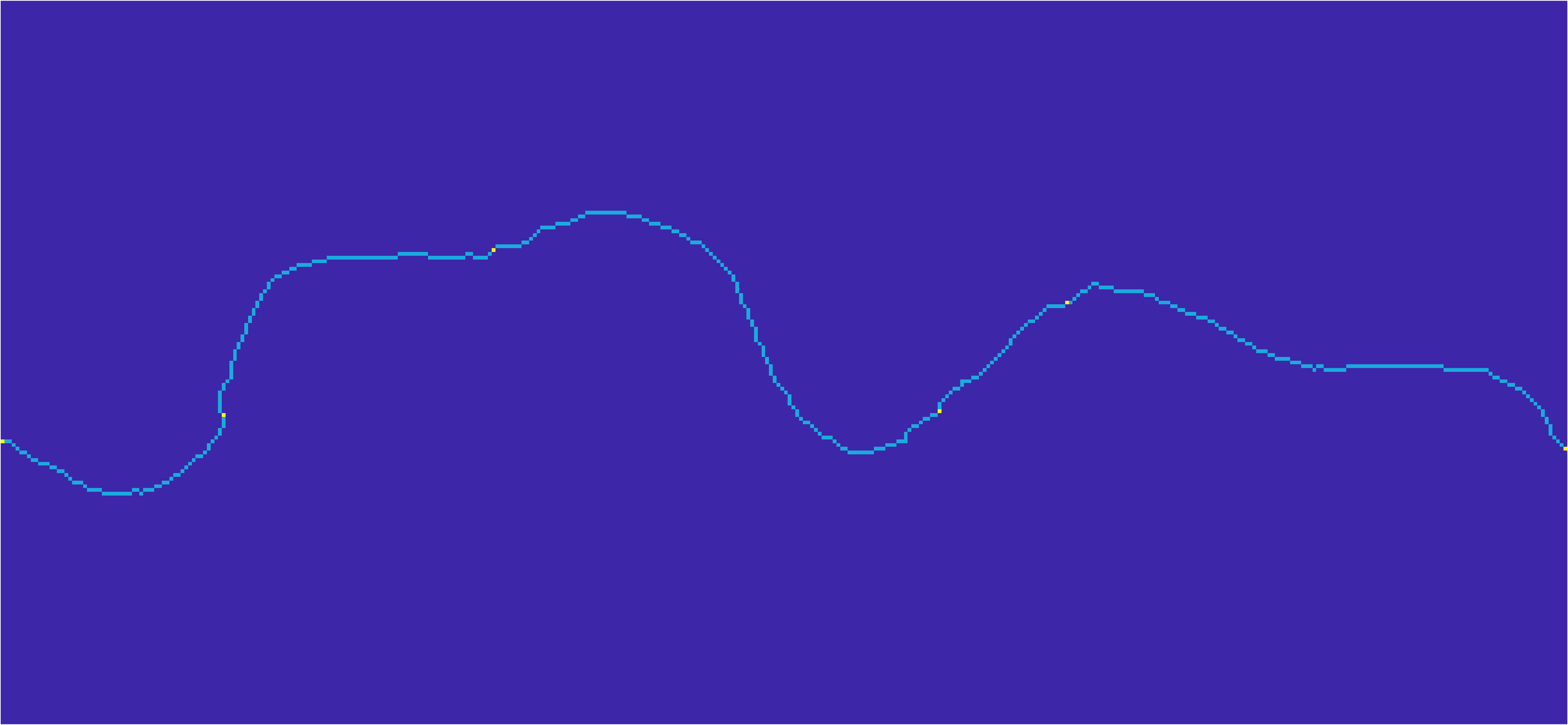

Supplement: S1 Appendix — Figures analogous to those shown in Figs. 3d, 3f, 3h, 3i, and 3j, are included. (ZIP) [file pone.0329379.s001.zip › S1 Appendix/014_Artery/h_centerline and division points_014.tif]

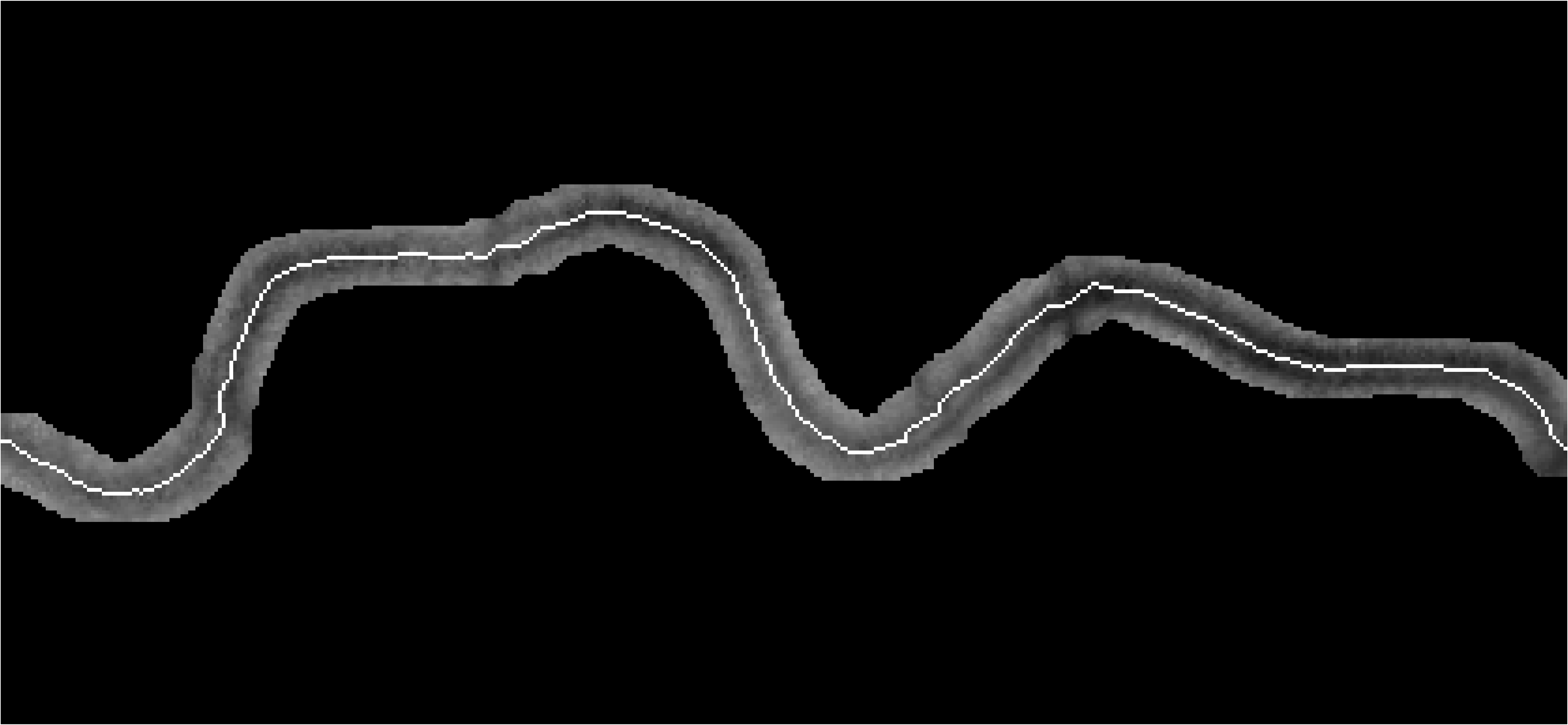

Supplement: S1 Appendix — Figures analogous to those shown in Figs. 3d, 3f, 3h, 3i, and 3j, are included. (ZIP) [file pone.0329379.s001.zip › S1 Appendix/014_Artery/d_ROI with manual trace_014.tif]

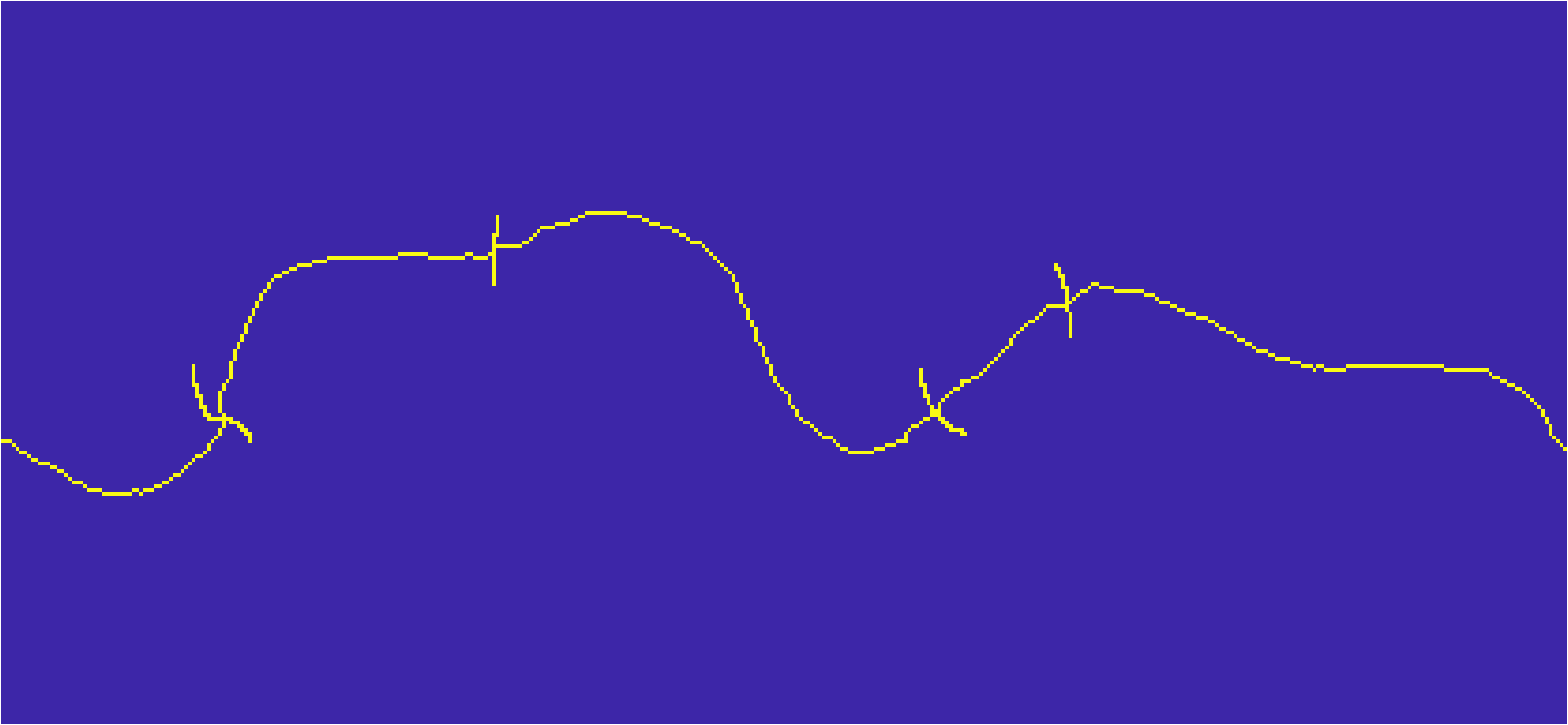

Supplement: S1 Appendix — Figures analogous to those shown in Figs. 3d, 3f, 3h, 3i, and 3j, are included. (ZIP) [file pone.0329379.s001.zip › S1 Appendix/014_Artery/f_Skeleton_014.tif]

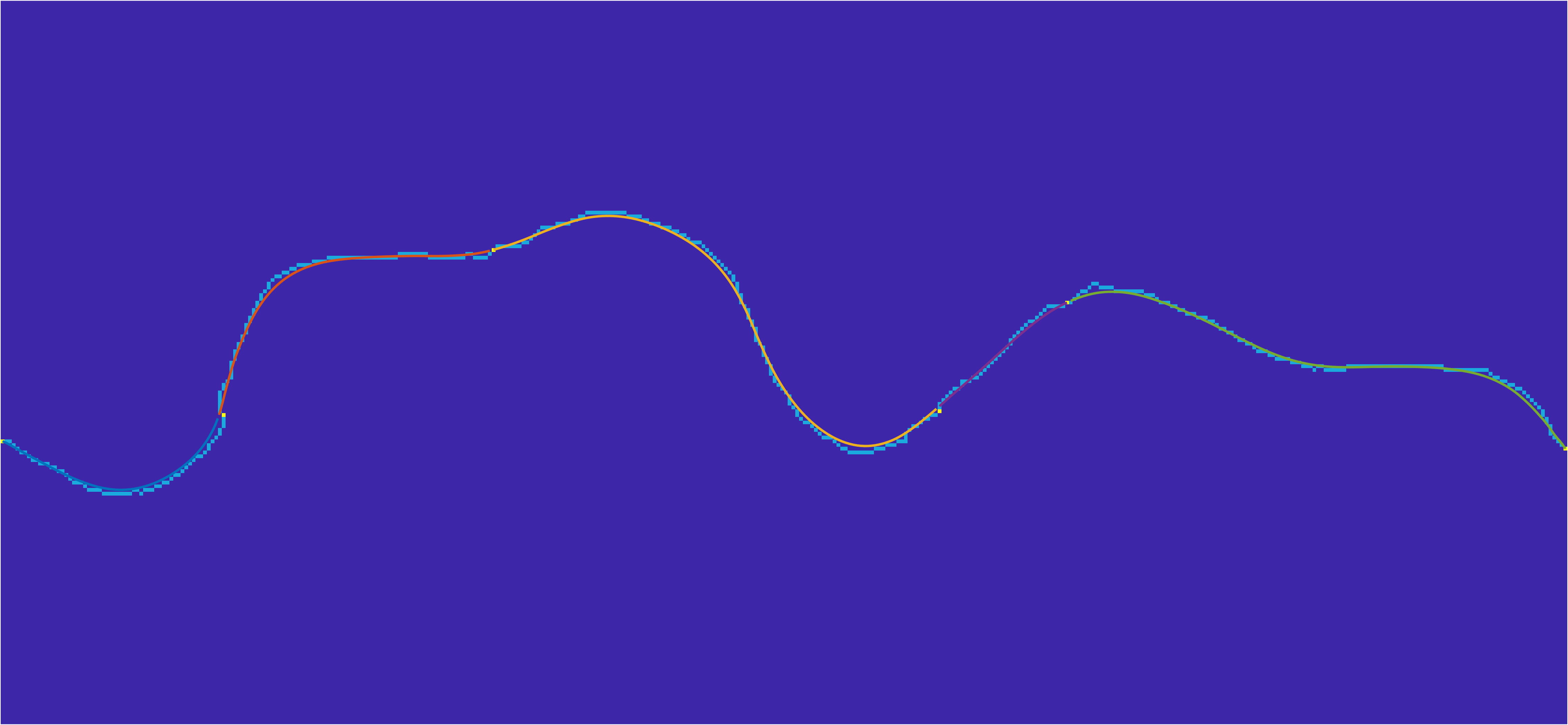

Supplement: S1 Appendix — Figures analogous to those shown in Figs. 3d, 3f, 3h, 3i, and 3j, are included. (ZIP) [file pone.0329379.s001.zip › S1 Appendix/014_Artery/j_partition_014.tif]

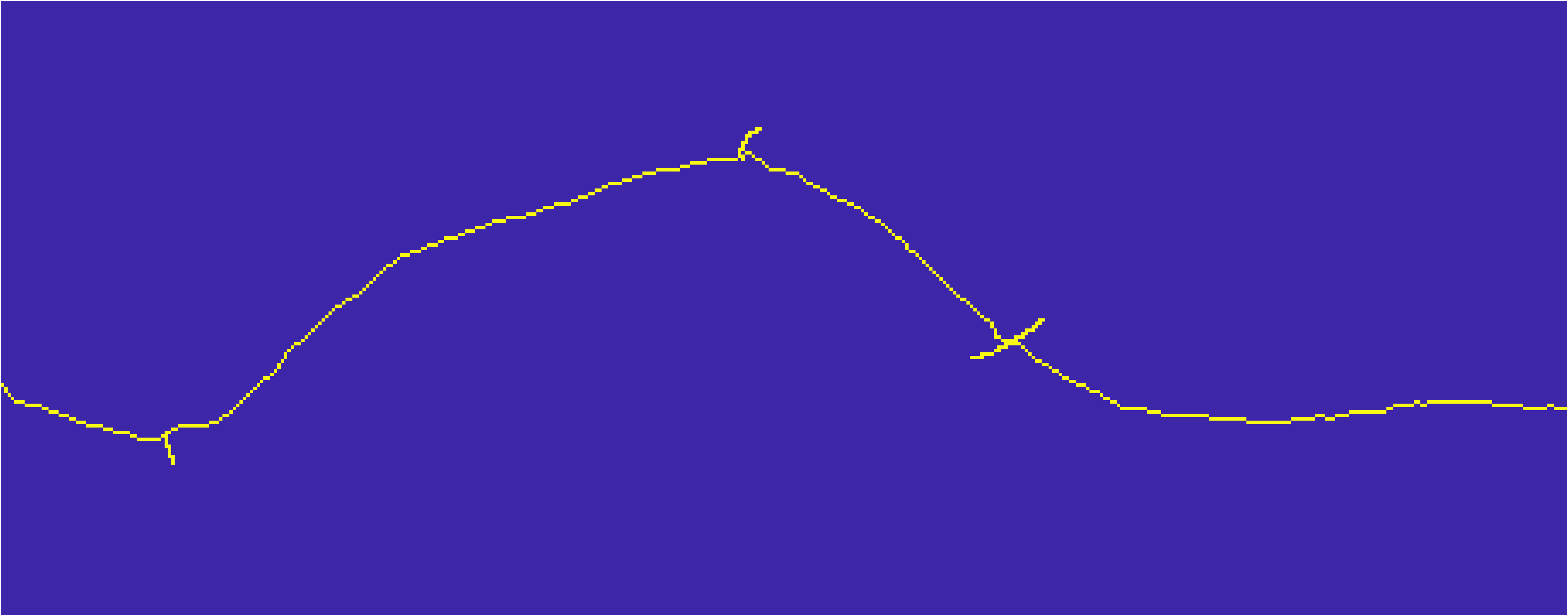

Supplement: S1 Appendix — Figures analogous to those shown in Figs. 3d, 3f, 3h, 3i, and 3j, are included. (ZIP) [file pone.0329379.s001.zip › S1 Appendix/169_Artery/f_Skeleton_169.tif]

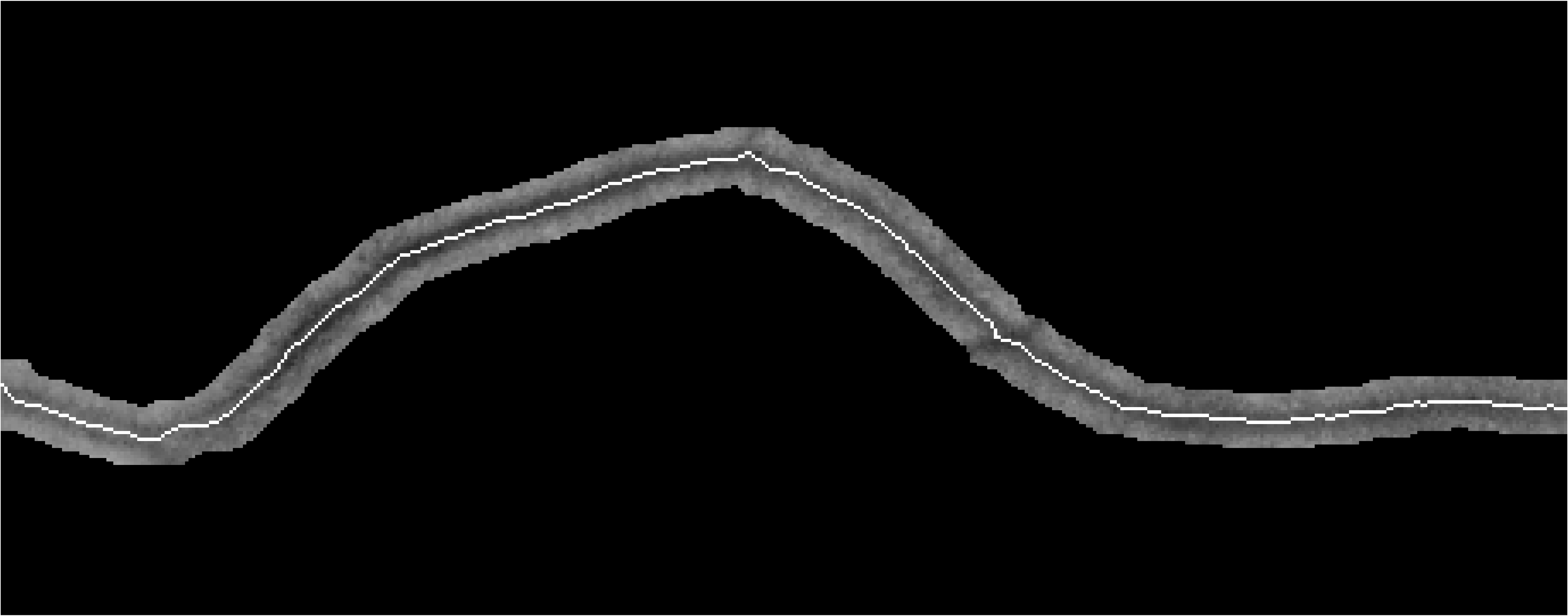

Supplement: S1 Appendix — Figures analogous to those shown in Figs. 3d, 3f, 3h, 3i, and 3j, are included. (ZIP) [file pone.0329379.s001.zip › S1 Appendix/169_Artery/d_ROI with manual trace_169.tif]

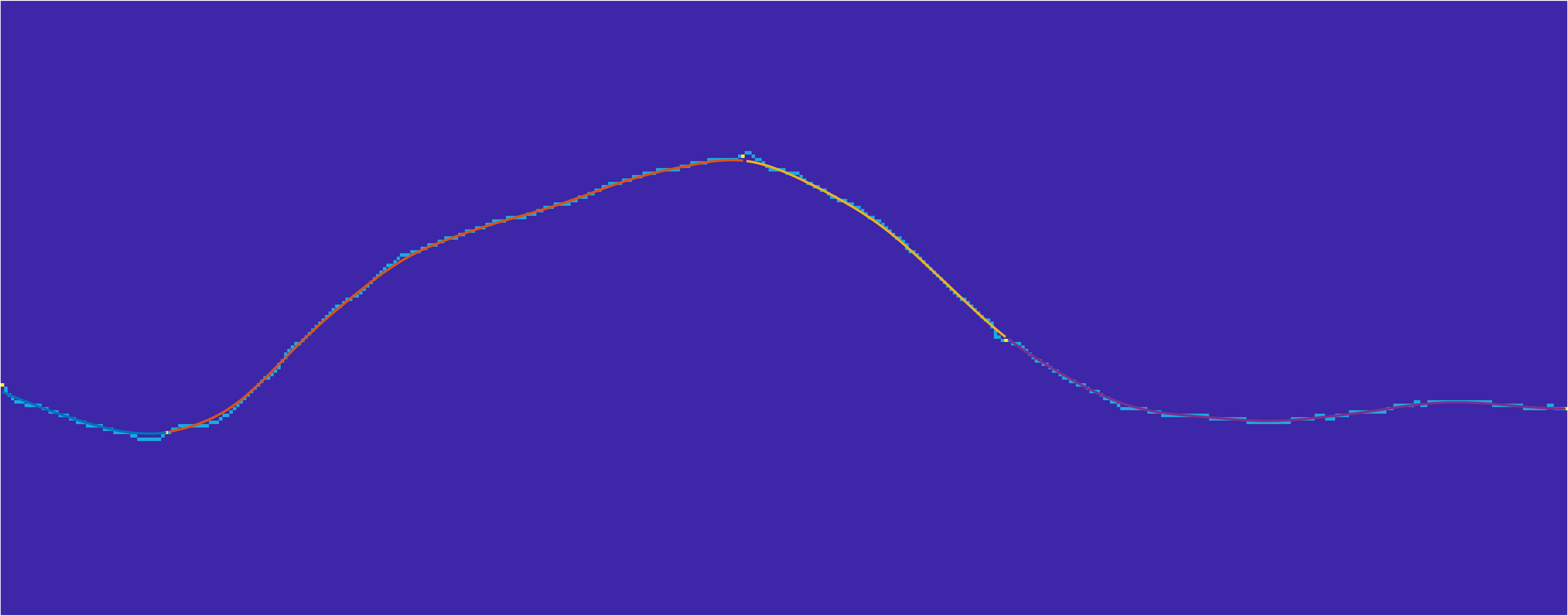

Supplement: S1 Appendix — Figures analogous to those shown in Figs. 3d, 3f, 3h, 3i, and 3j, are included. (ZIP) [file pone.0329379.s001.zip › S1 Appendix/169_Artery/j_partition_169.tif]

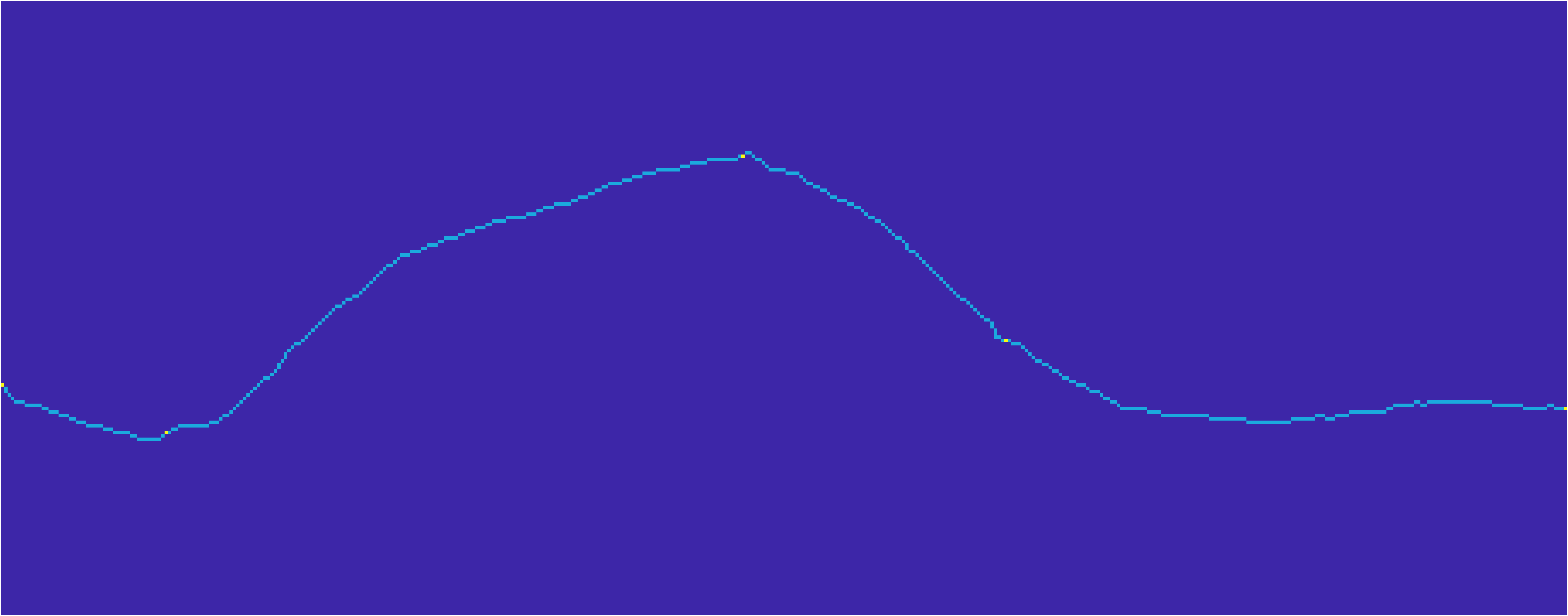

Supplement: S1 Appendix — Figures analogous to those shown in Figs. 3d, 3f, 3h, 3i, and 3j, are included. (ZIP) [file pone.0329379.s001.zip › S1 Appendix/169_Artery/h_centerline and division points_169.tif]

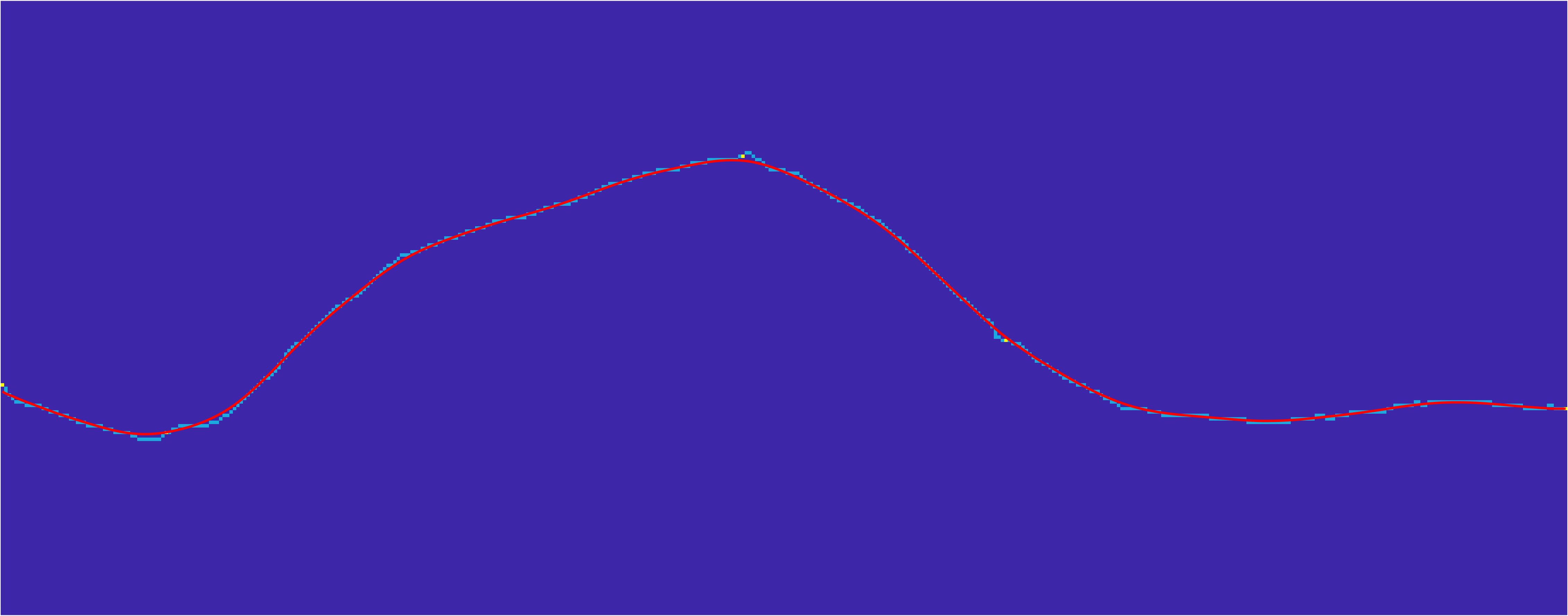

Supplement: S1 Appendix — Figures analogous to those shown in Figs. 3d, 3f, 3h, 3i, and 3j, are included. (ZIP) [file pone.0329379.s001.zip › S1 Appendix/169_Artery/i_smoothed segment_169.tif]

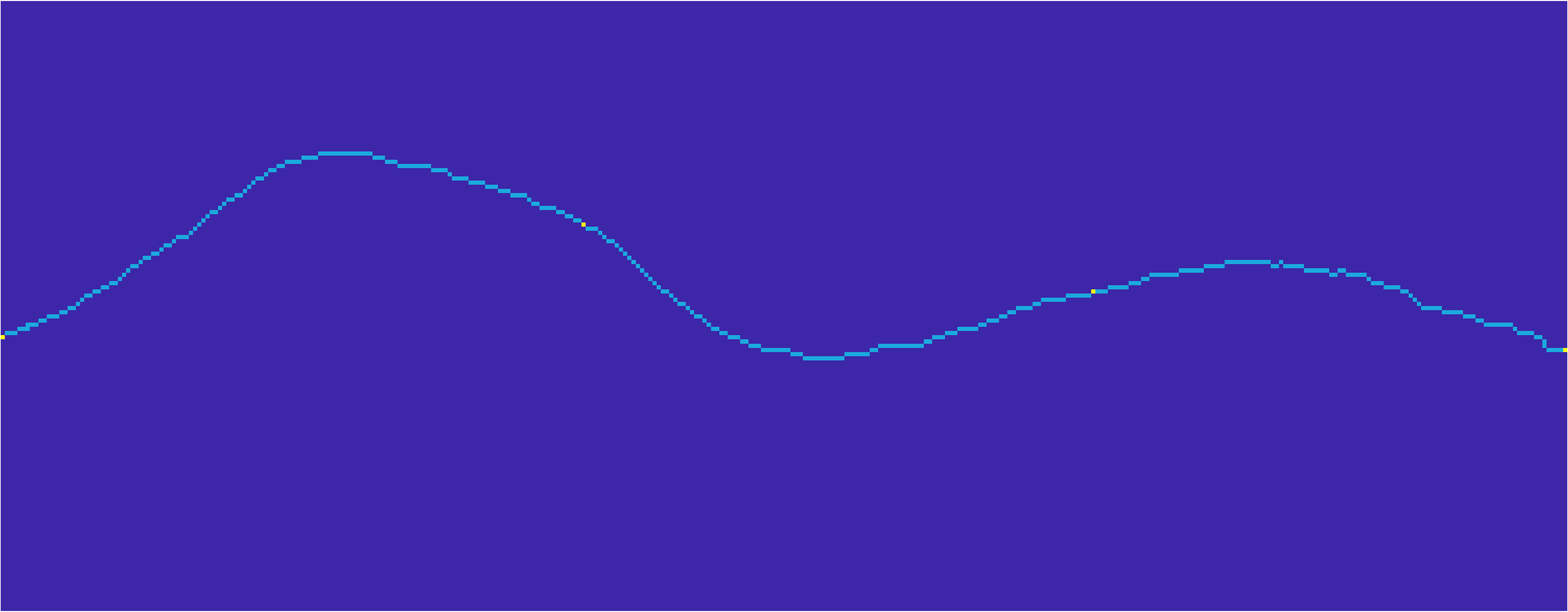

Supplement: S1 Appendix — Figures analogous to those shown in Figs. 3d, 3f, 3h, 3i, and 3j, are included. (ZIP) [file pone.0329379.s001.zip › S1 Appendix/242_Artery/h_centerline and division points_242.tif]

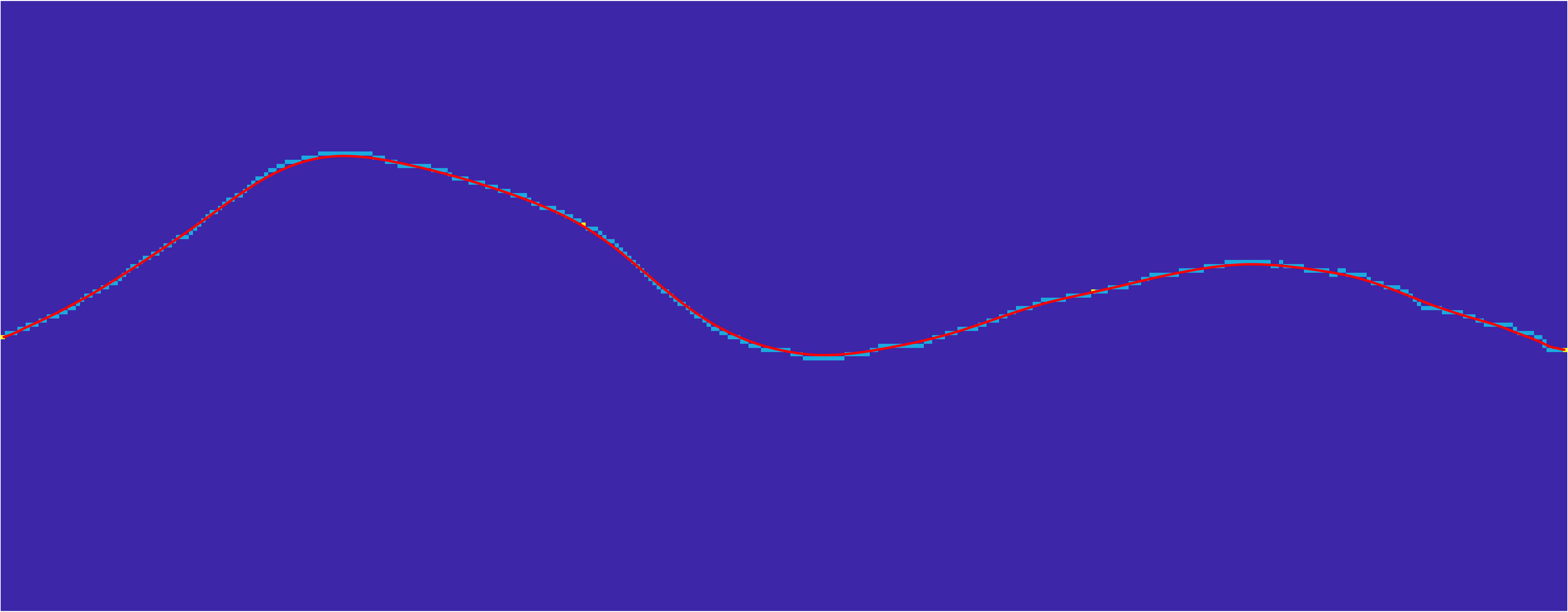

Supplement: S1 Appendix — Figures analogous to those shown in Figs. 3d, 3f, 3h, 3i, and 3j, are included. (ZIP) [file pone.0329379.s001.zip › S1 Appendix/242_Artery/i_smoothed segment_242.tif]

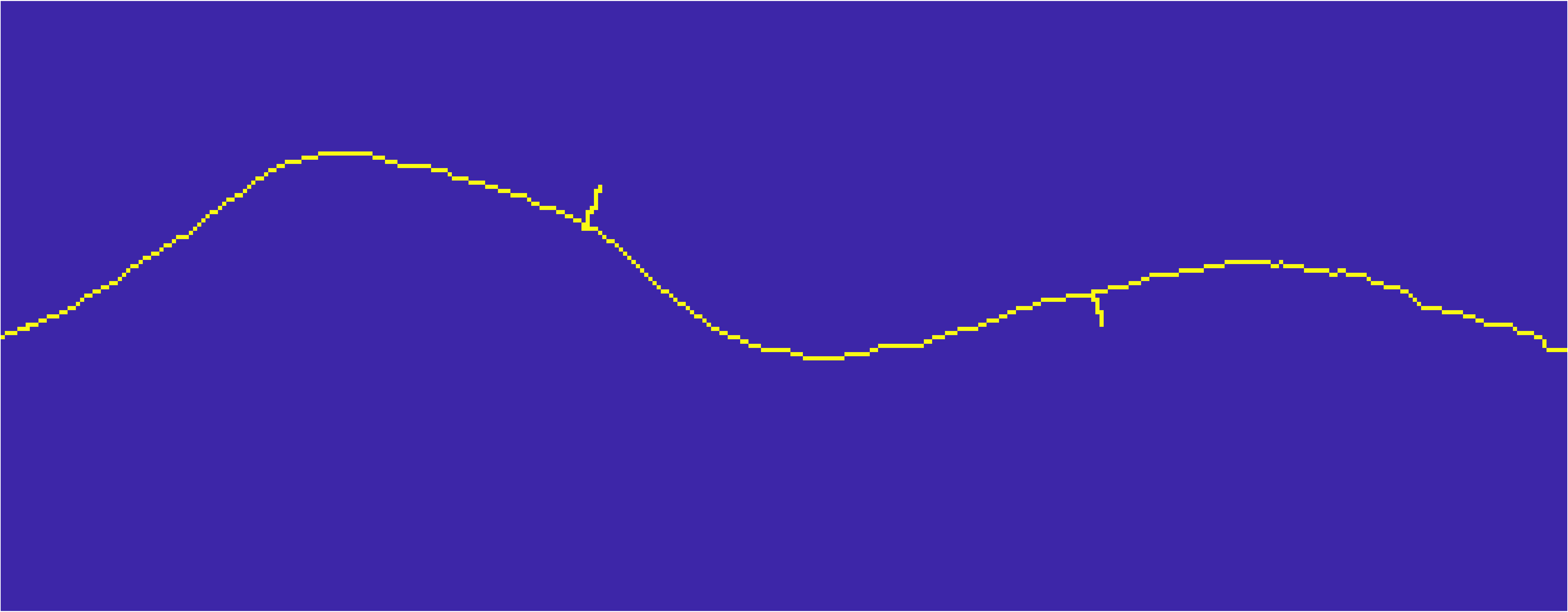

Supplement: S1 Appendix — Figures analogous to those shown in Figs. 3d, 3f, 3h, 3i, and 3j, are included. (ZIP) [file pone.0329379.s001.zip › S1 Appendix/242_Artery/f_Skeleton_242.tif]

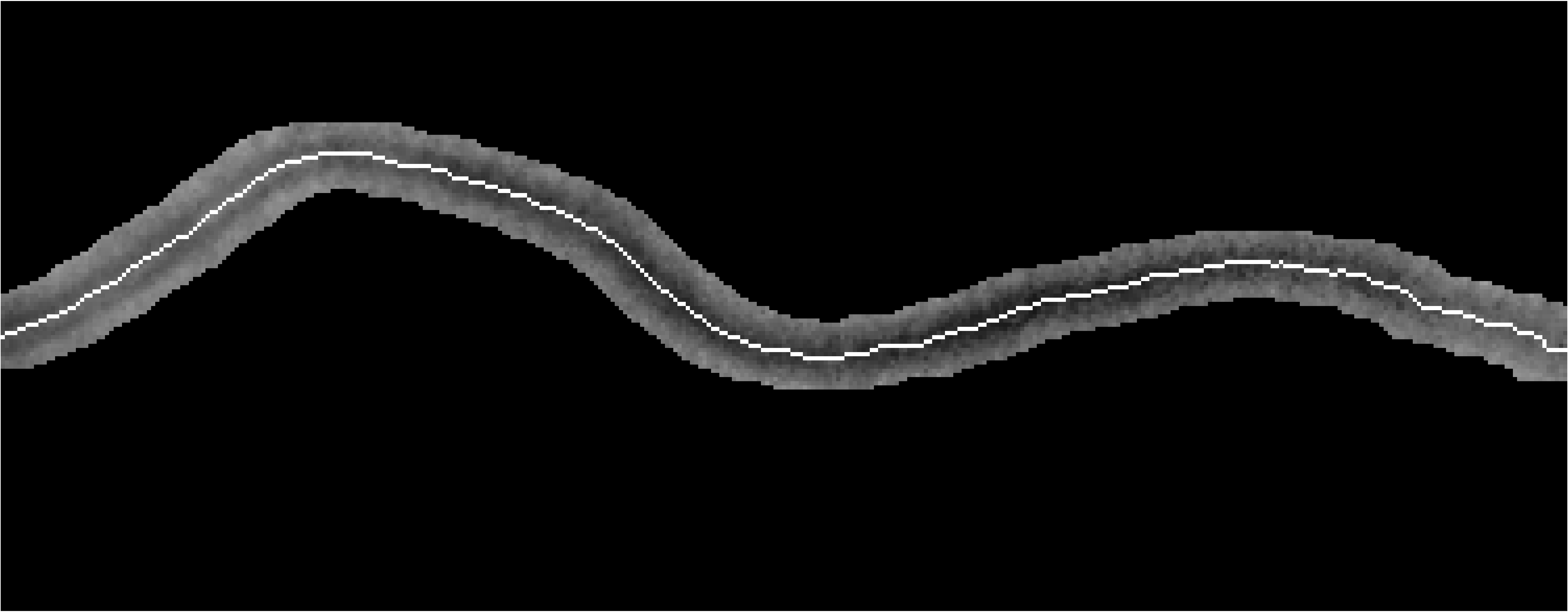

Supplement: S1 Appendix — Figures analogous to those shown in Figs. 3d, 3f, 3h, 3i, and 3j, are included. (ZIP) [file pone.0329379.s001.zip › S1 Appendix/242_Artery/d_ROI with manual trace_242.tif]

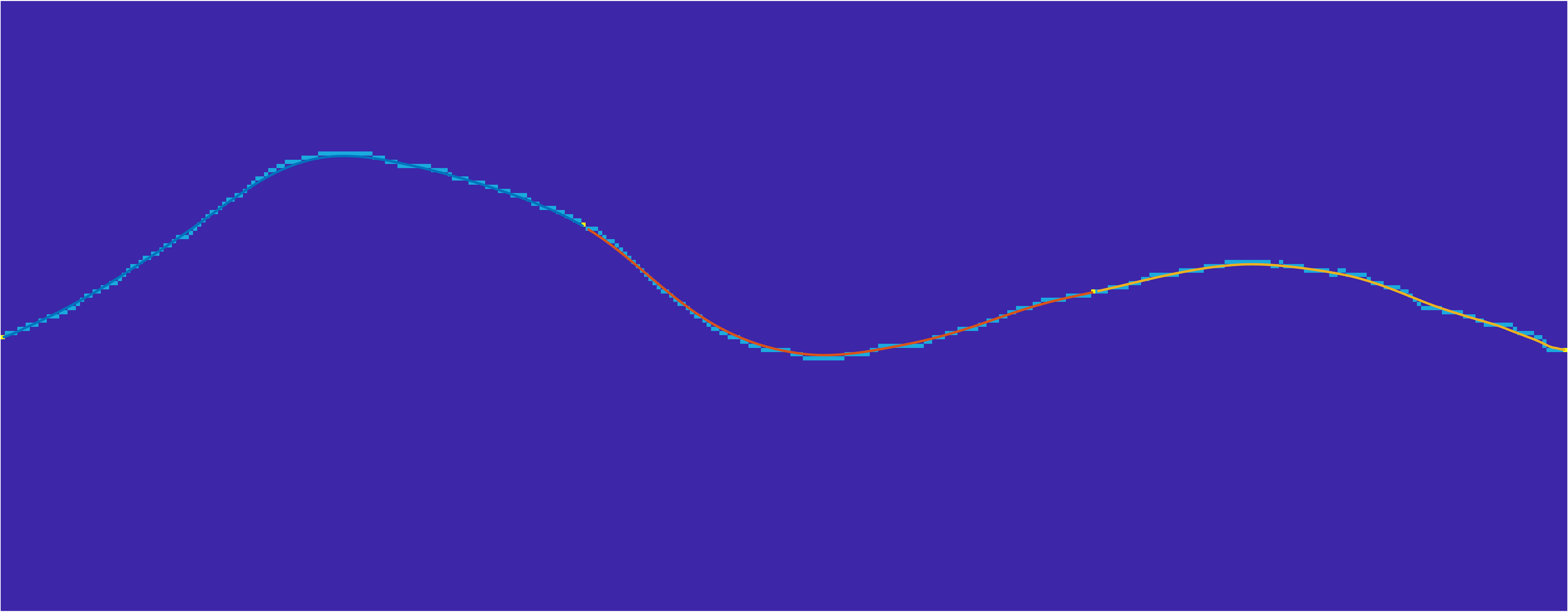

Supplement: S1 Appendix — Figures analogous to those shown in Figs. 3d, 3f, 3h, 3i, and 3j, are included. (ZIP) [file pone.0329379.s001.zip › S1 Appendix/242_Artery/j_partition_242.tif]

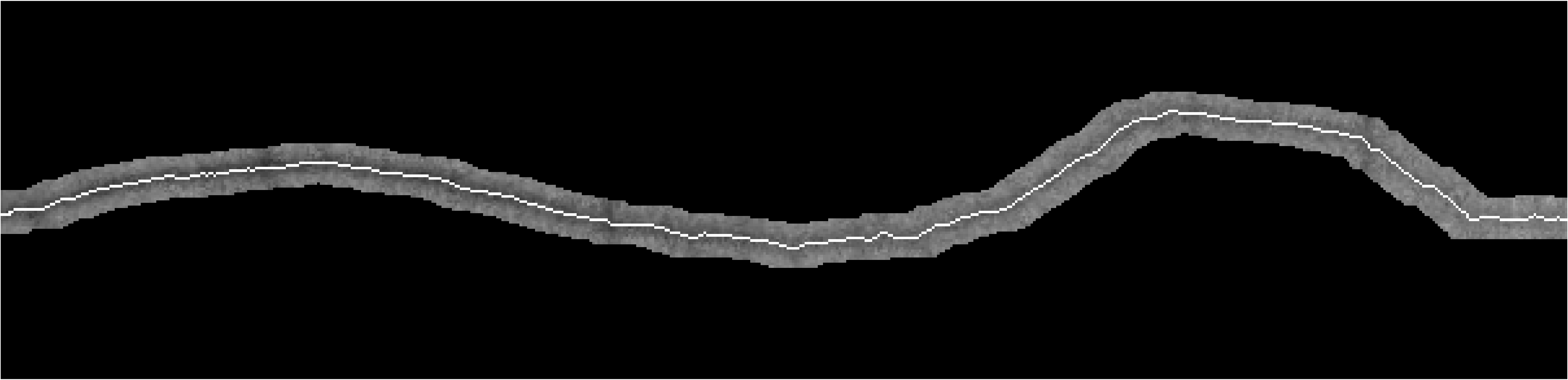

Supplement: S1 Appendix — Figures analogous to those shown in Figs. 3d, 3f, 3h, 3i, and 3j, are included. (ZIP) [file pone.0329379.s001.zip › S1 Appendix/209_Artery/d_ROI with manual trace_209.tif]

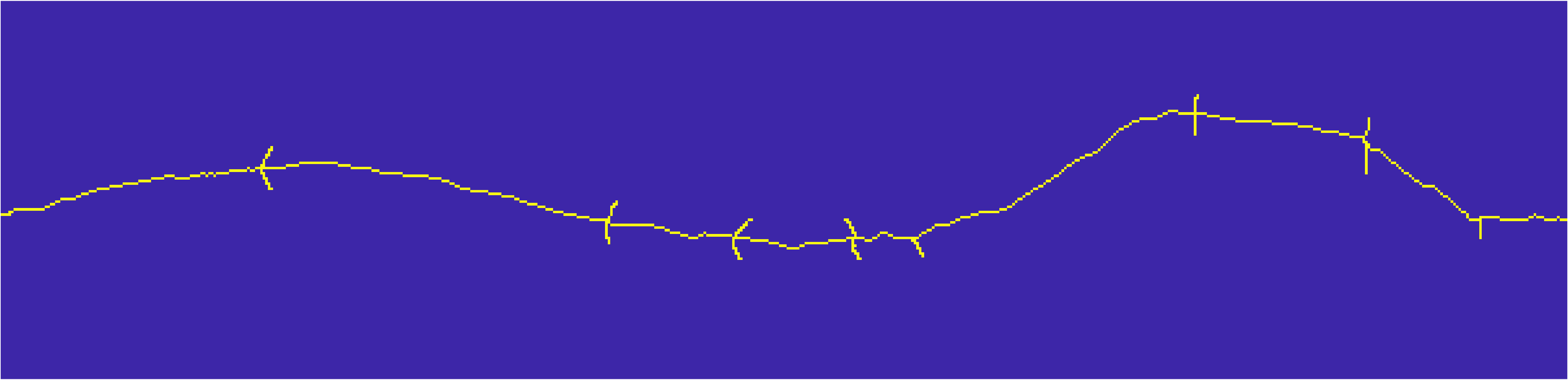

Supplement: S1 Appendix — Figures analogous to those shown in Figs. 3d, 3f, 3h, 3i, and 3j, are included. (ZIP) [file pone.0329379.s001.zip › S1 Appendix/209_Artery/f_Skeleton_209.tif]

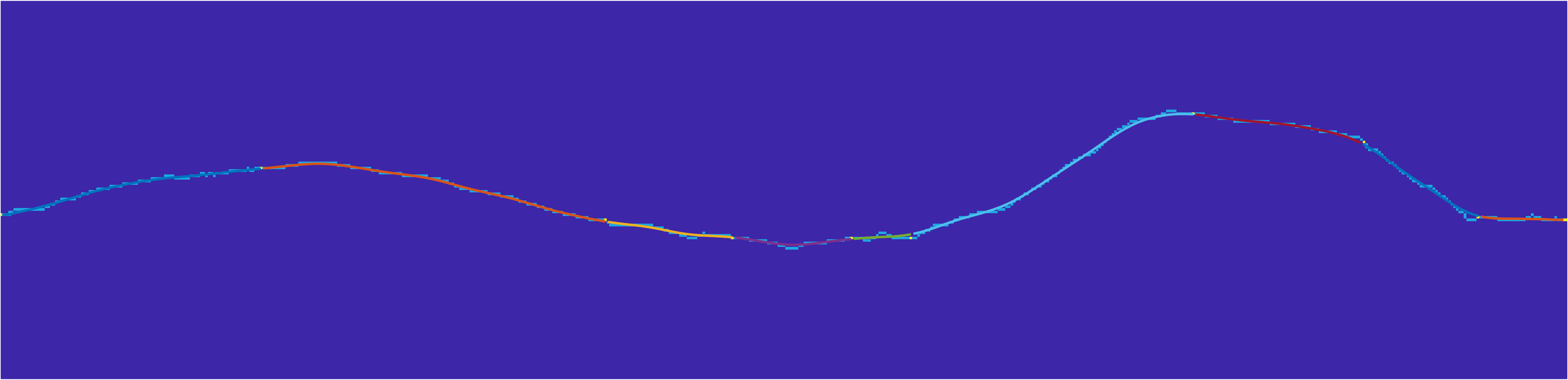

Supplement: S1 Appendix — Figures analogous to those shown in Figs. 3d, 3f, 3h, 3i, and 3j, are included. (ZIP) [file pone.0329379.s001.zip › S1 Appendix/209_Artery/j_partition_209.tif]

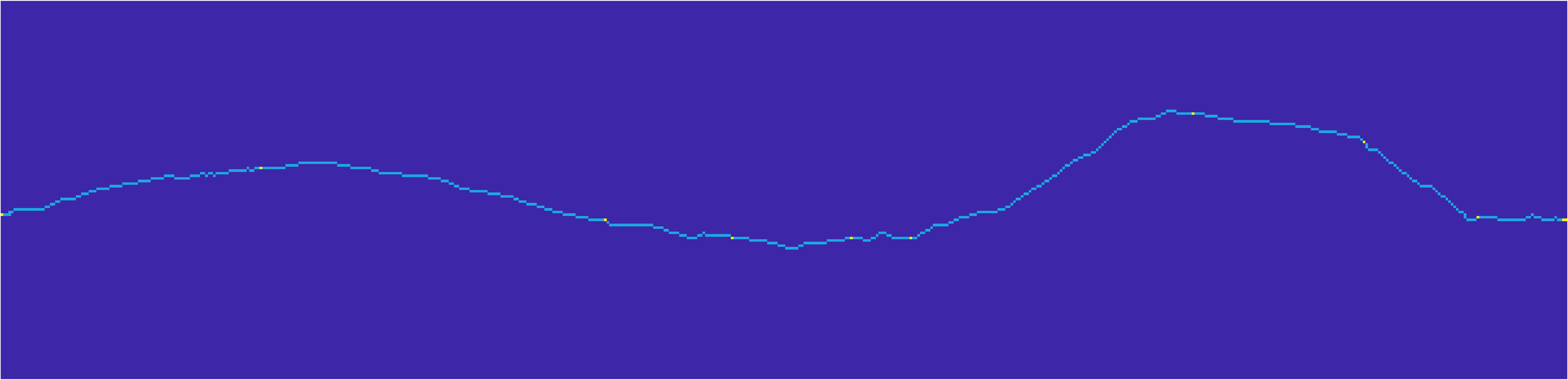

Supplement: S1 Appendix — Figures analogous to those shown in Figs. 3d, 3f, 3h, 3i, and 3j, are included. (ZIP) [file pone.0329379.s001.zip › S1 Appendix/209_Artery/h_centerline and division points_209.tif]

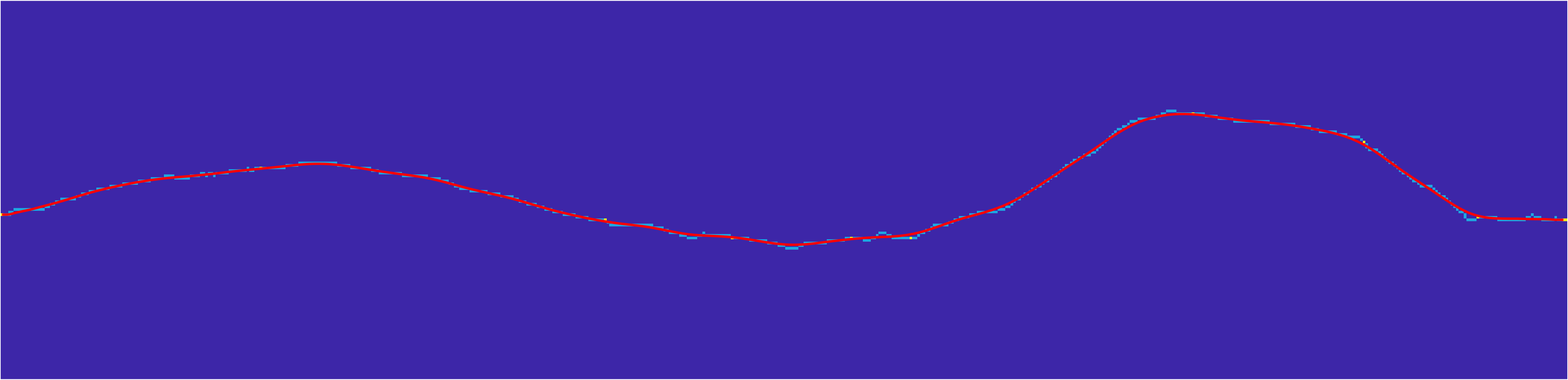

Supplement: S1 Appendix — Figures analogous to those shown in Figs. 3d, 3f, 3h, 3i, and 3j, are included. (ZIP) [file pone.0329379.s001.zip › S1 Appendix/209_Artery/i_smoothed segment_209.tif]

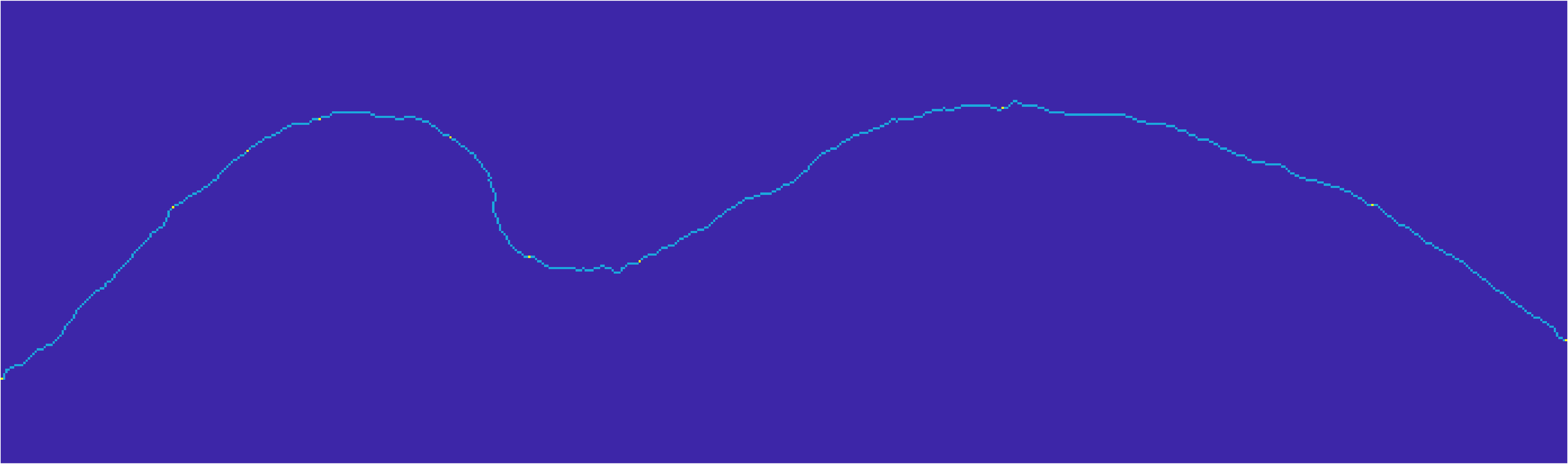

Supplement: S1 Appendix — Figures analogous to those shown in Figs. 3d, 3f, 3h, 3i, and 3j, are included. (ZIP) [file pone.0329379.s001.zip › S1 Appendix/205_Artery/h_centerline and division points_205.tif]

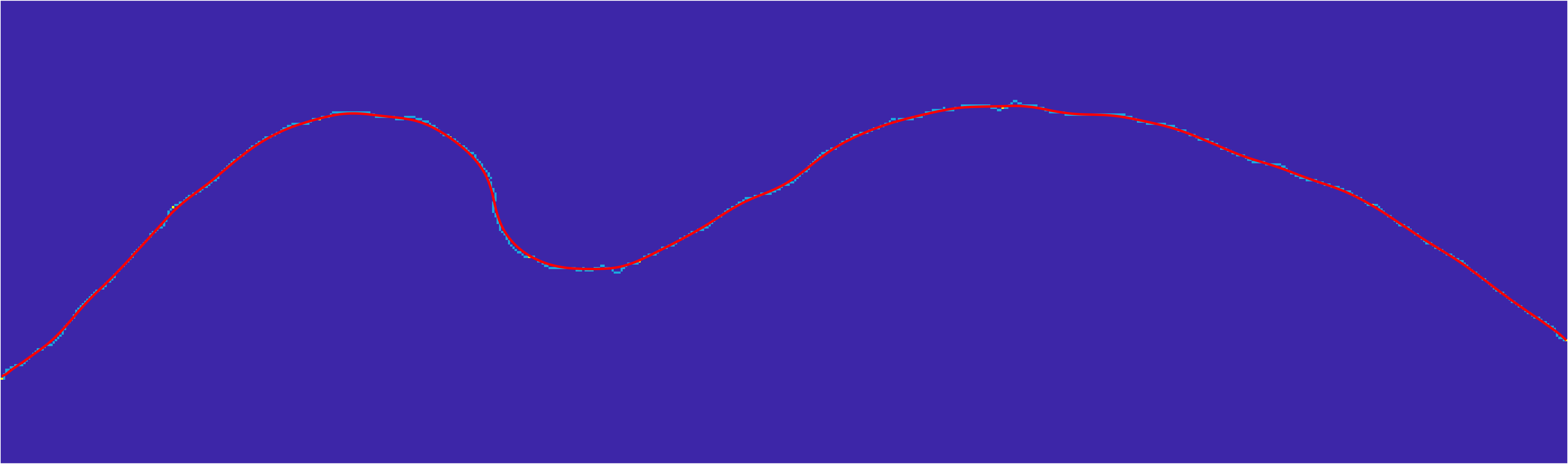

Supplement: S1 Appendix — Figures analogous to those shown in Figs. 3d, 3f, 3h, 3i, and 3j, are included. (ZIP) [file pone.0329379.s001.zip › S1 Appendix/205_Artery/i_smoothed segment_205.tif]

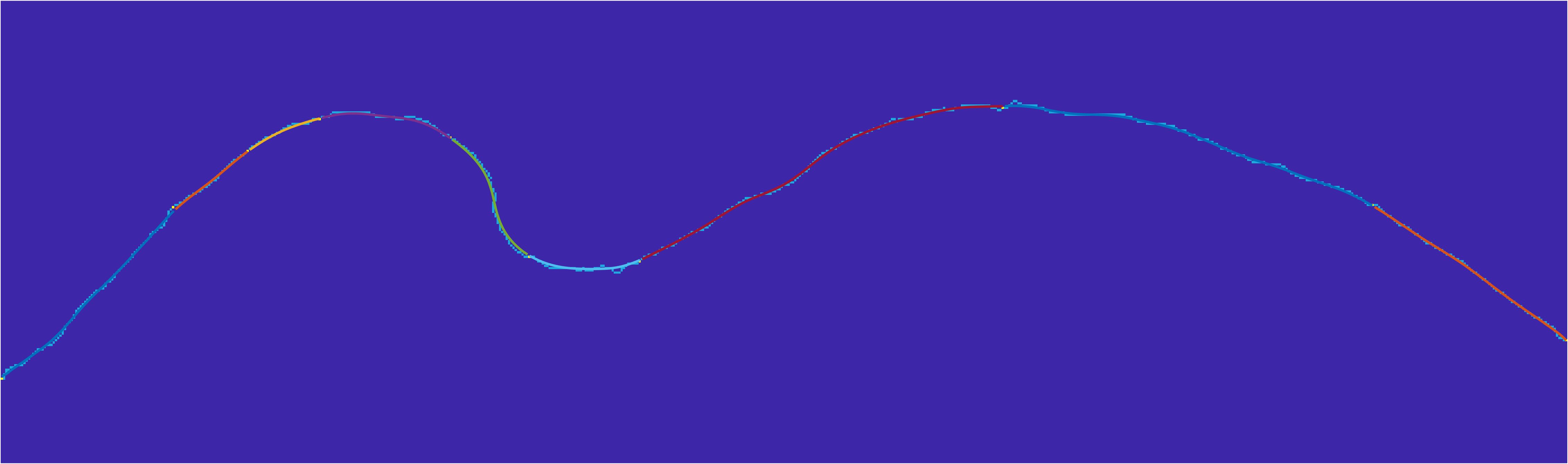

Supplement: S1 Appendix — Figures analogous to those shown in Figs. 3d, 3f, 3h, 3i, and 3j, are included. (ZIP) [file pone.0329379.s001.zip › S1 Appendix/205_Artery/j_partition_205.tif]

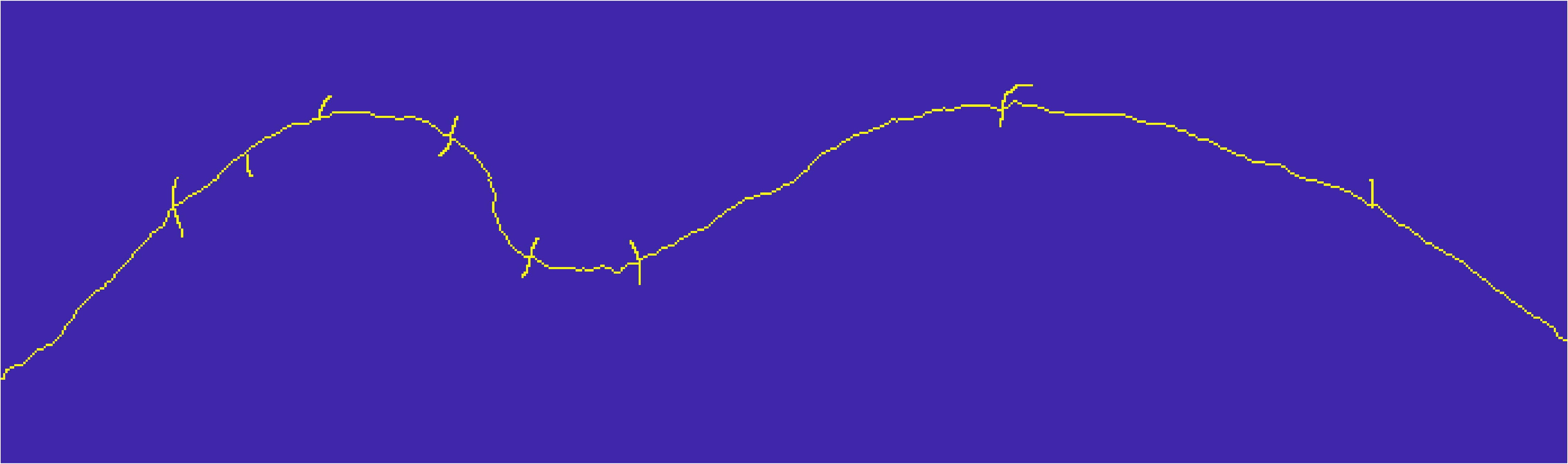

Supplement: S1 Appendix — Figures analogous to those shown in Figs. 3d, 3f, 3h, 3i, and 3j, are included. (ZIP) [file pone.0329379.s001.zip › S1 Appendix/205_Artery/f_Skeleton_205.tif]

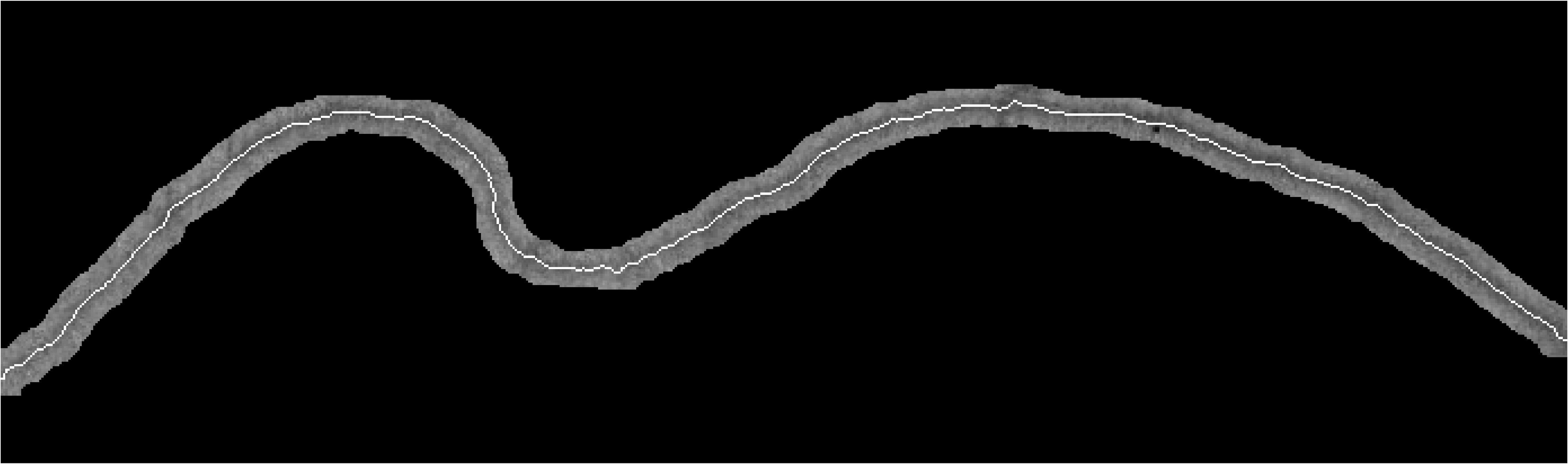

Supplement: S1 Appendix — Figures analogous to those shown in Figs. 3d, 3f, 3h, 3i, and 3j, are included. (ZIP) [file pone.0329379.s001.zip › S1 Appendix/205_Artery/d_ROI with manual trace_205.tif]

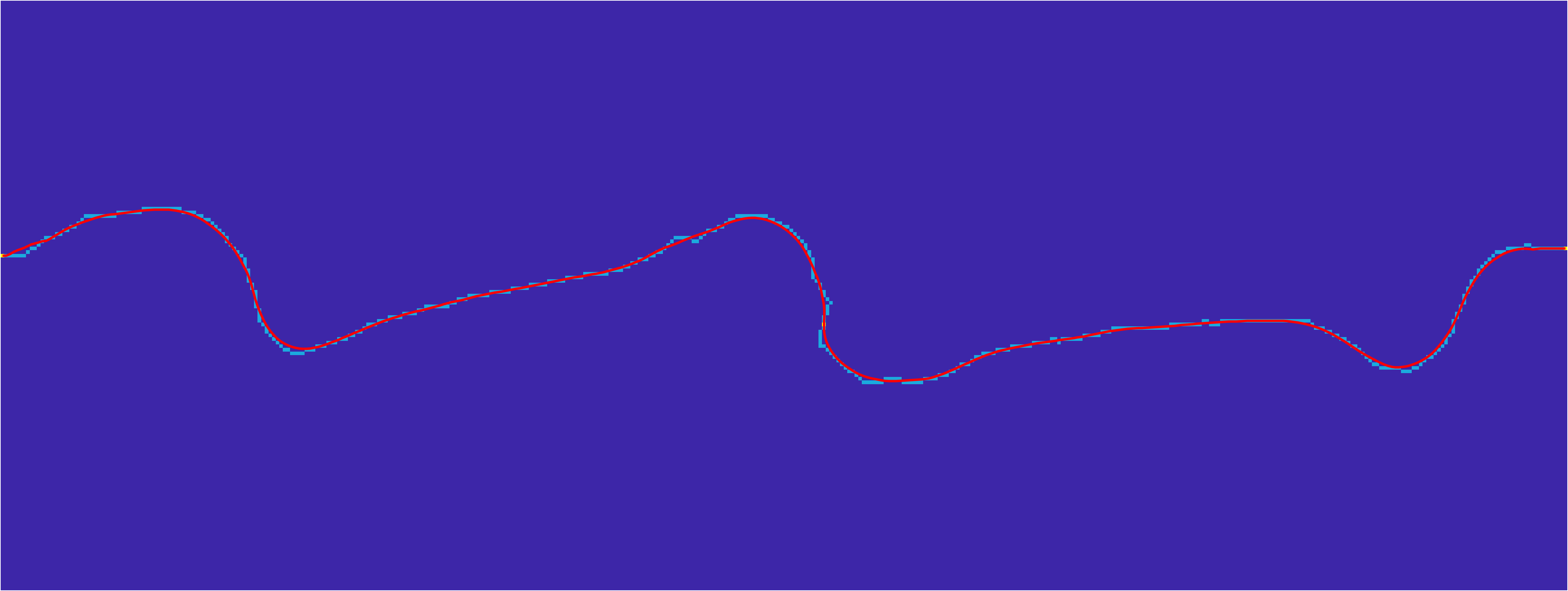

Supplement: S1 Appendix — Figures analogous to those shown in Figs. 3d, 3f, 3h, 3i, and 3j, are included. (ZIP) [file pone.0329379.s001.zip › S1 Appendix/011_Artery/i_smoothed segment_011.tif]

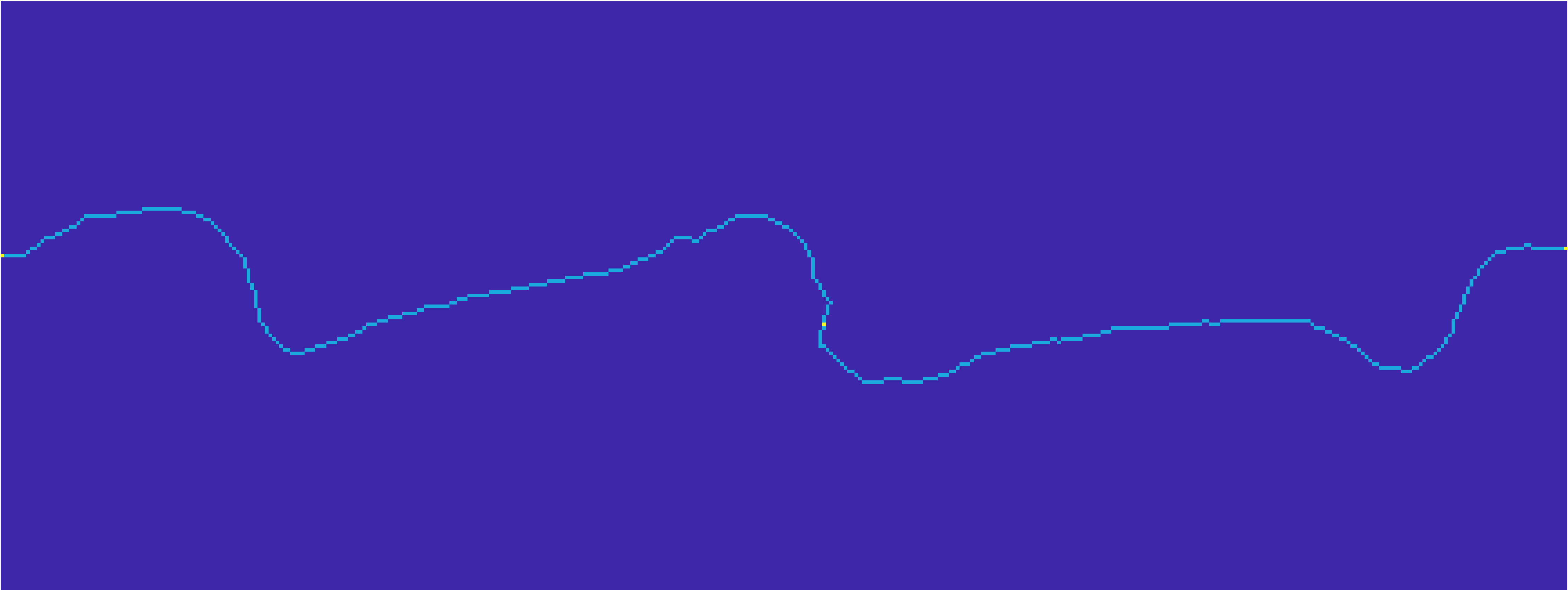

Supplement: S1 Appendix — Figures analogous to those shown in Figs. 3d, 3f, 3h, 3i, and 3j, are included. (ZIP) [file pone.0329379.s001.zip › S1 Appendix/011_Artery/h_centerline and division points_011.tif]

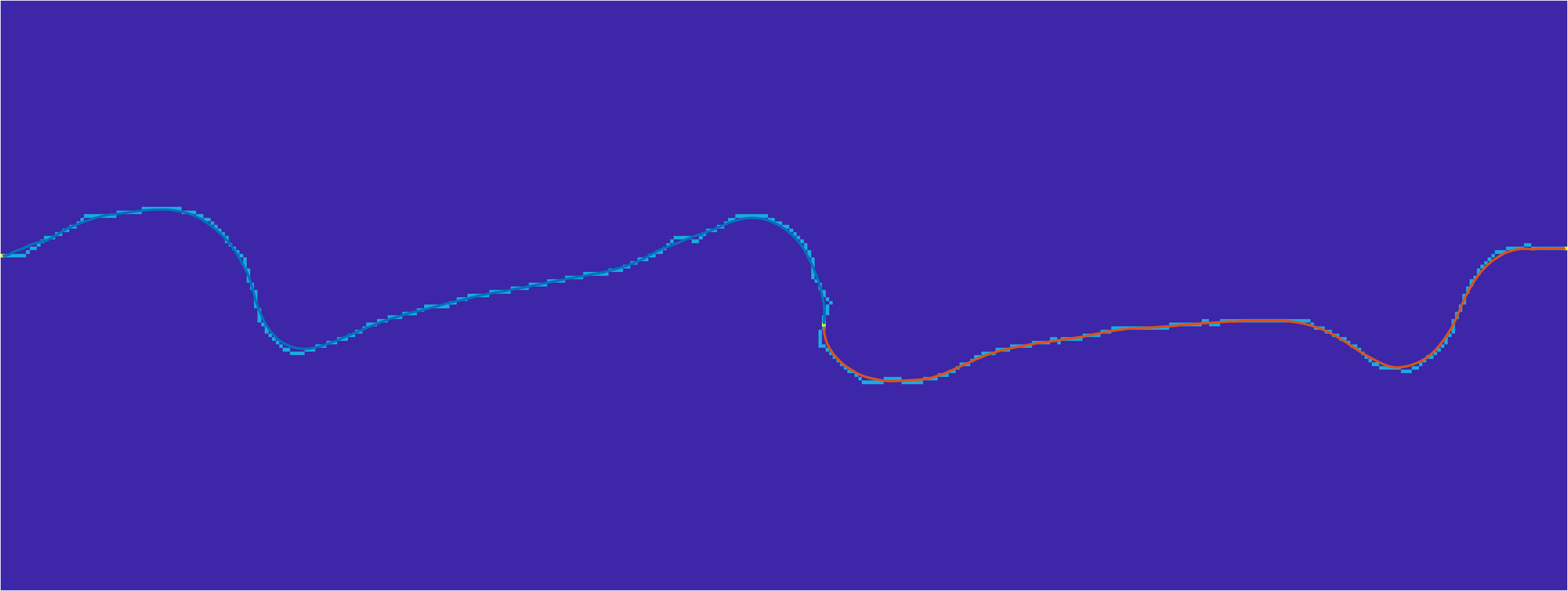

Supplement: S1 Appendix — Figures analogous to those shown in Figs. 3d, 3f, 3h, 3i, and 3j, are included. (ZIP) [file pone.0329379.s001.zip › S1 Appendix/011_Artery/j_partition_011.tif]

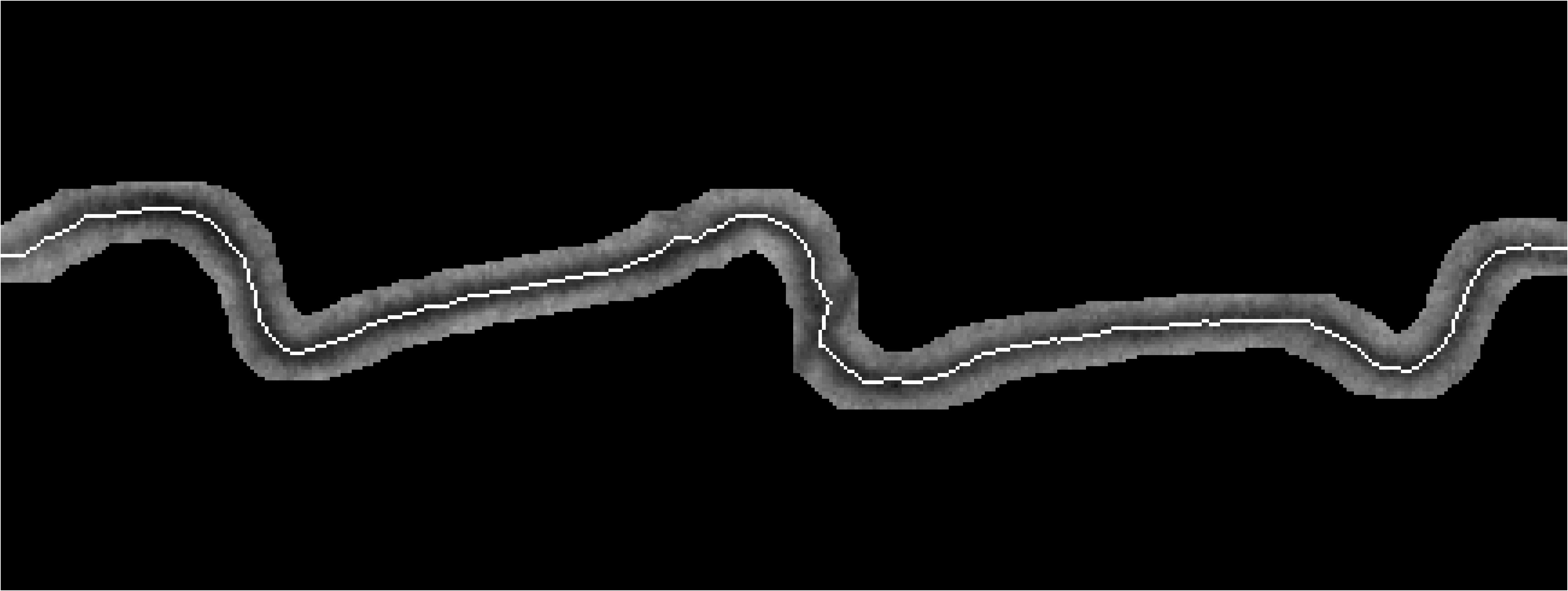

Supplement: S1 Appendix — Figures analogous to those shown in Figs. 3d, 3f, 3h, 3i, and 3j, are included. (ZIP) [file pone.0329379.s001.zip › S1 Appendix/011_Artery/d_ROI with manual trace_011.tif]

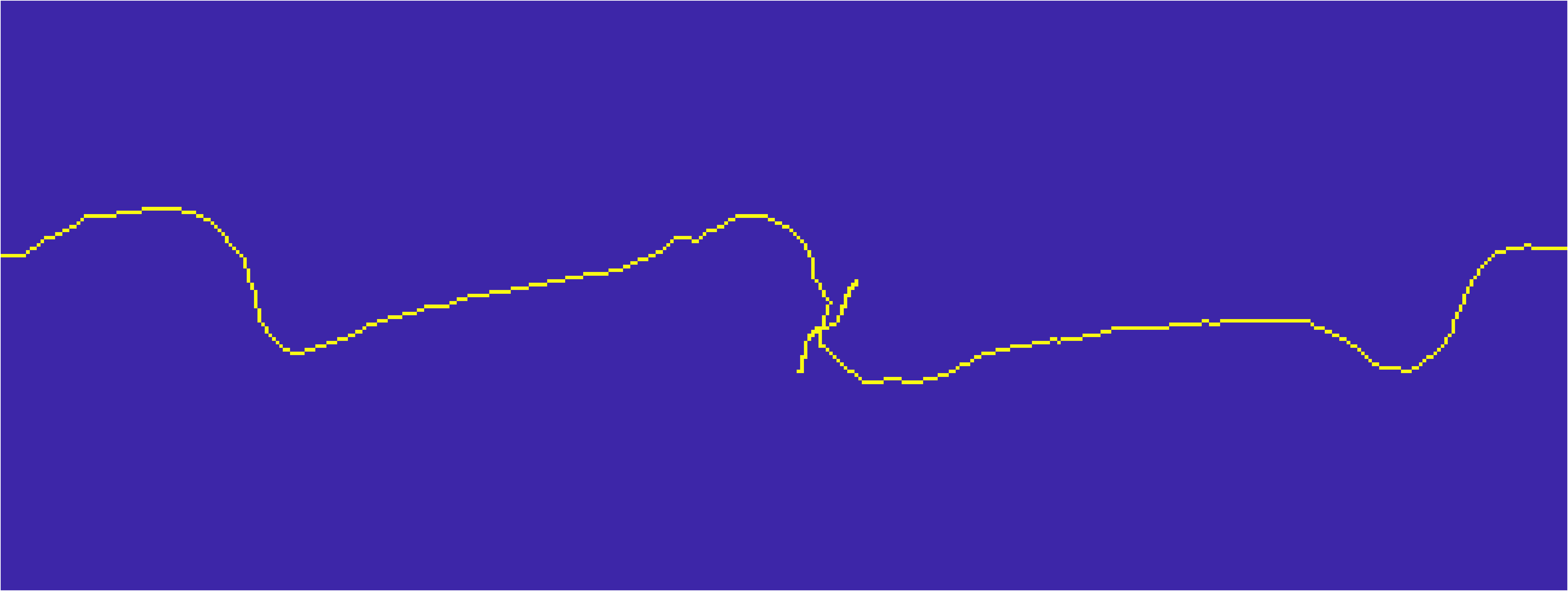

Supplement: S1 Appendix — Figures analogous to those shown in Figs. 3d, 3f, 3h, 3i, and 3j, are included. (ZIP) [file pone.0329379.s001.zip › S1 Appendix/011_Artery/f_Skeleton_011.tif]

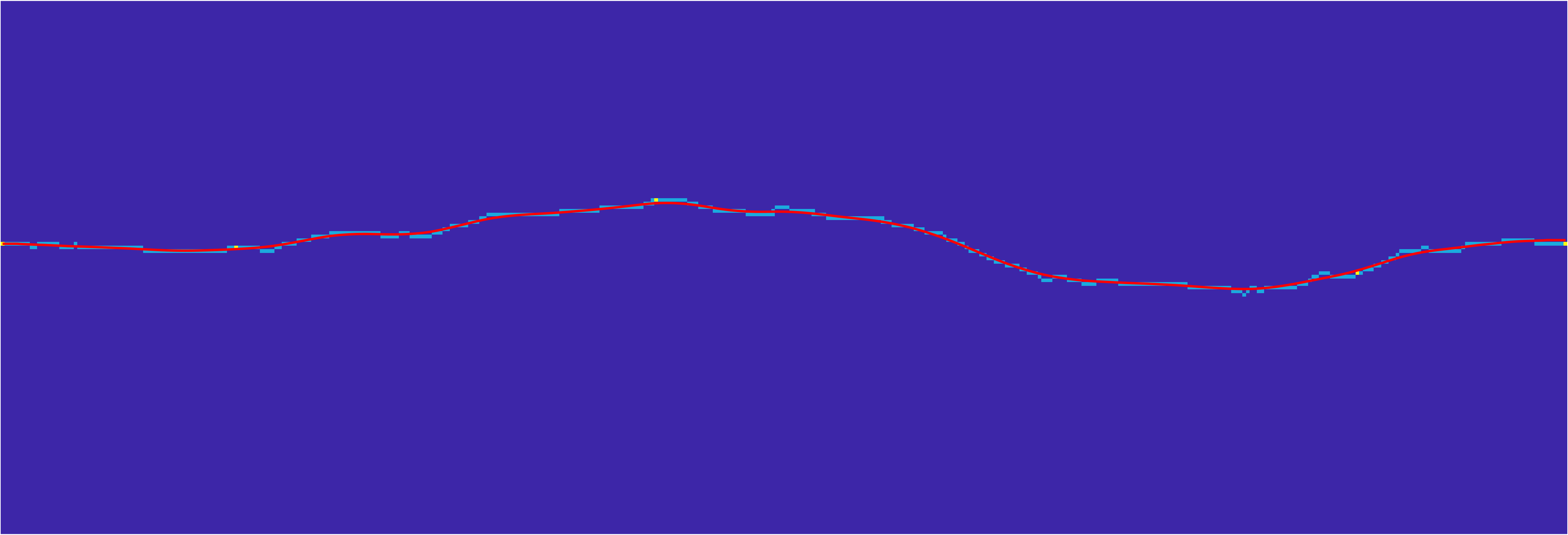

Supplement: S1 Appendix — Figures analogous to those shown in Figs. 3d, 3f, 3h, 3i, and 3j, are included. (ZIP) [file pone.0329379.s001.zip › S1 Appendix/056_Artery/i_smoothed segment_056.tif]

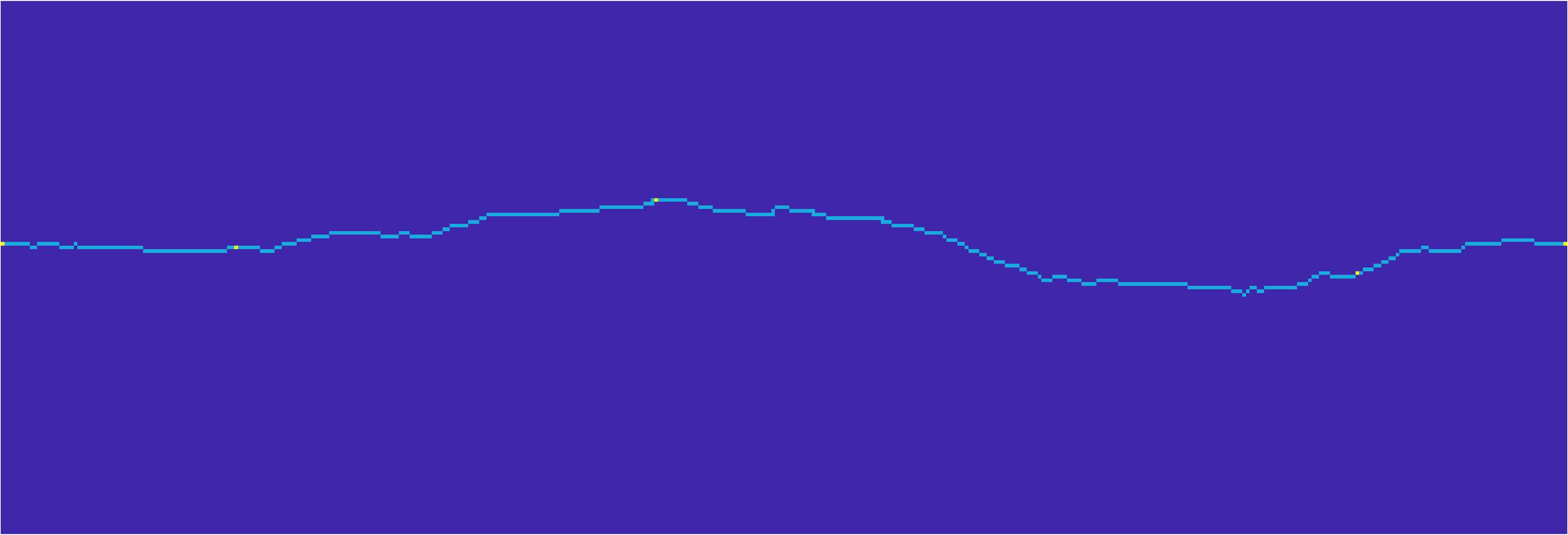

Supplement: S1 Appendix — Figures analogous to those shown in Figs. 3d, 3f, 3h, 3i, and 3j, are included. (ZIP) [file pone.0329379.s001.zip › S1 Appendix/056_Artery/h_centerline and division points_056.tif]

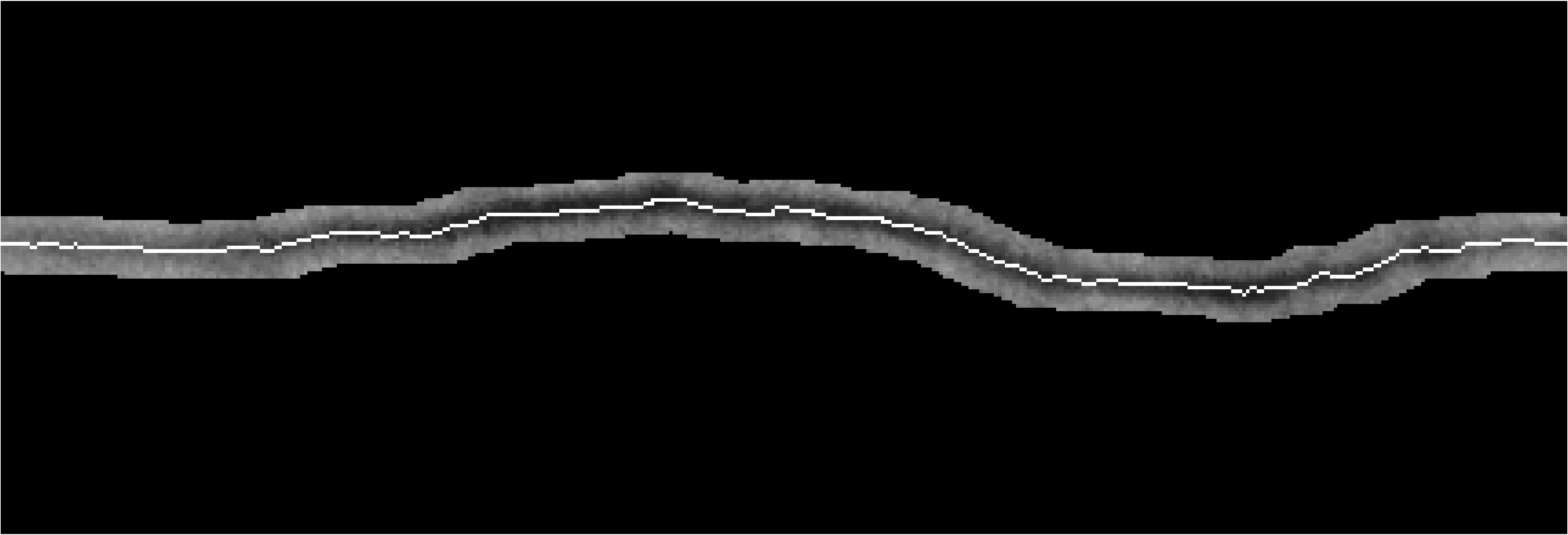

Supplement: S1 Appendix — Figures analogous to those shown in Figs. 3d, 3f, 3h, 3i, and 3j, are included. (ZIP) [file pone.0329379.s001.zip › S1 Appendix/056_Artery/d_ROI with manual trace_056.tif]

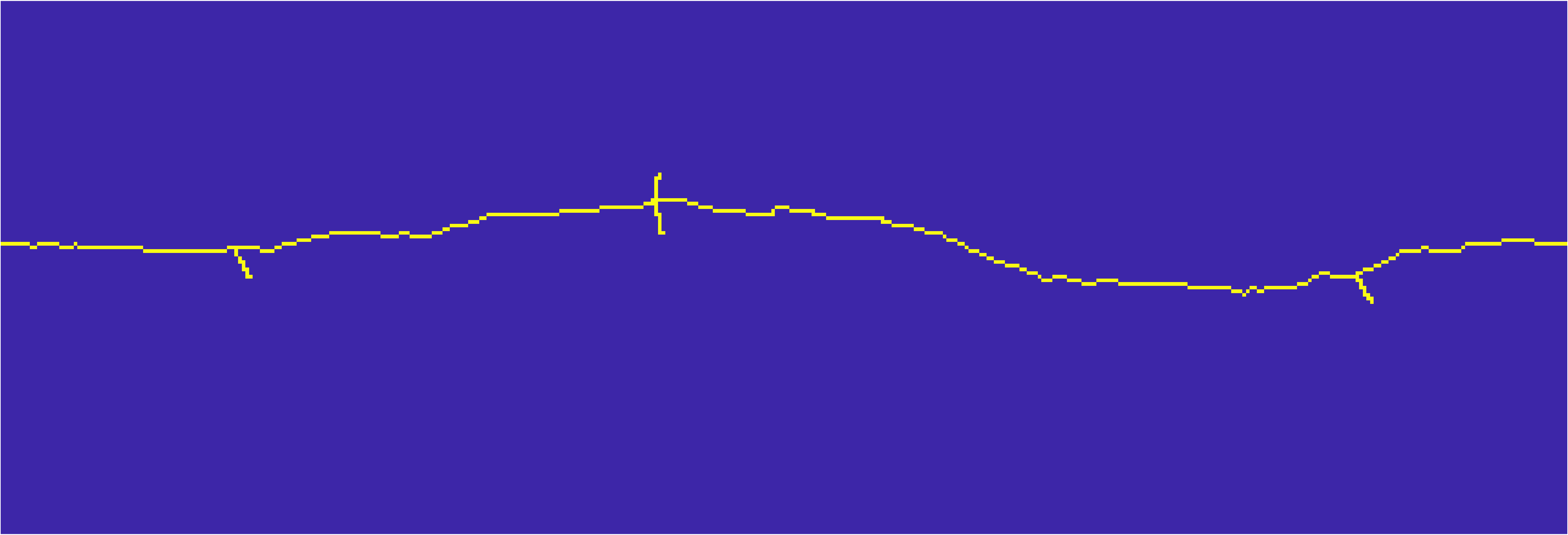

Supplement: S1 Appendix — Figures analogous to those shown in Figs. 3d, 3f, 3h, 3i, and 3j, are included. (ZIP) [file pone.0329379.s001.zip › S1 Appendix/056_Artery/f_Skeleton_056.tif]

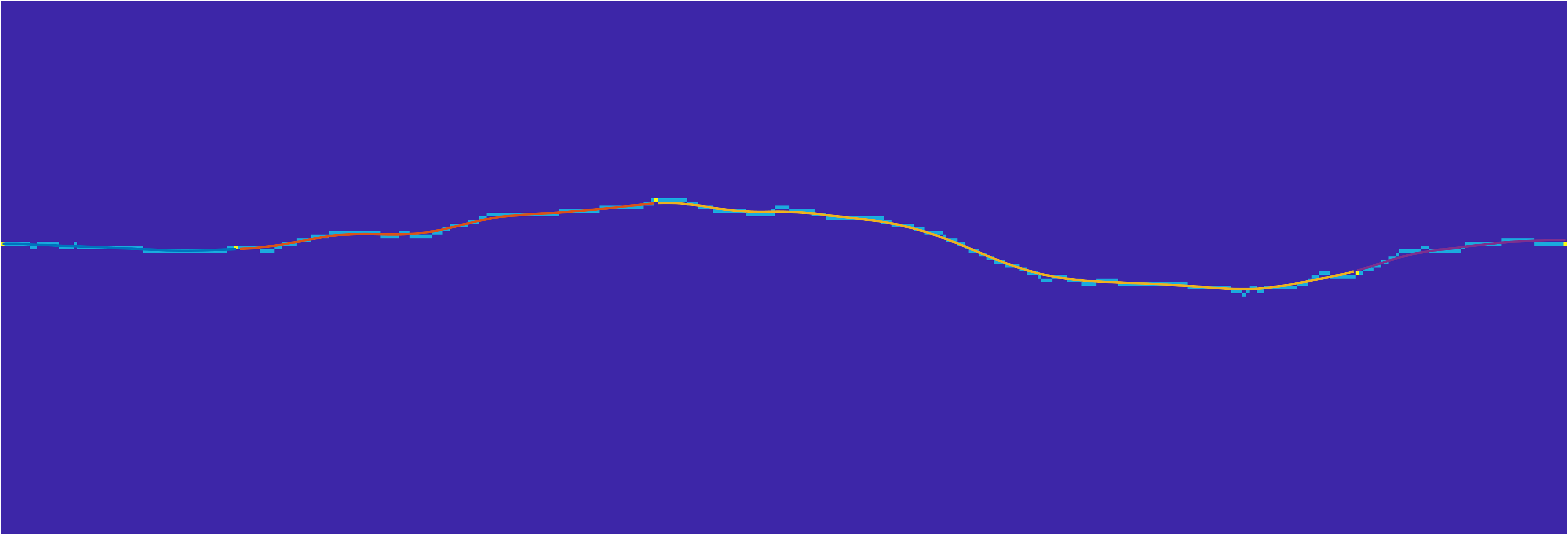

Supplement: S1 Appendix — Figures analogous to those shown in Figs. 3d, 3f, 3h, 3i, and 3j, are included. (ZIP) [file pone.0329379.s001.zip › S1 Appendix/056_Artery/j_partition_056.tif]

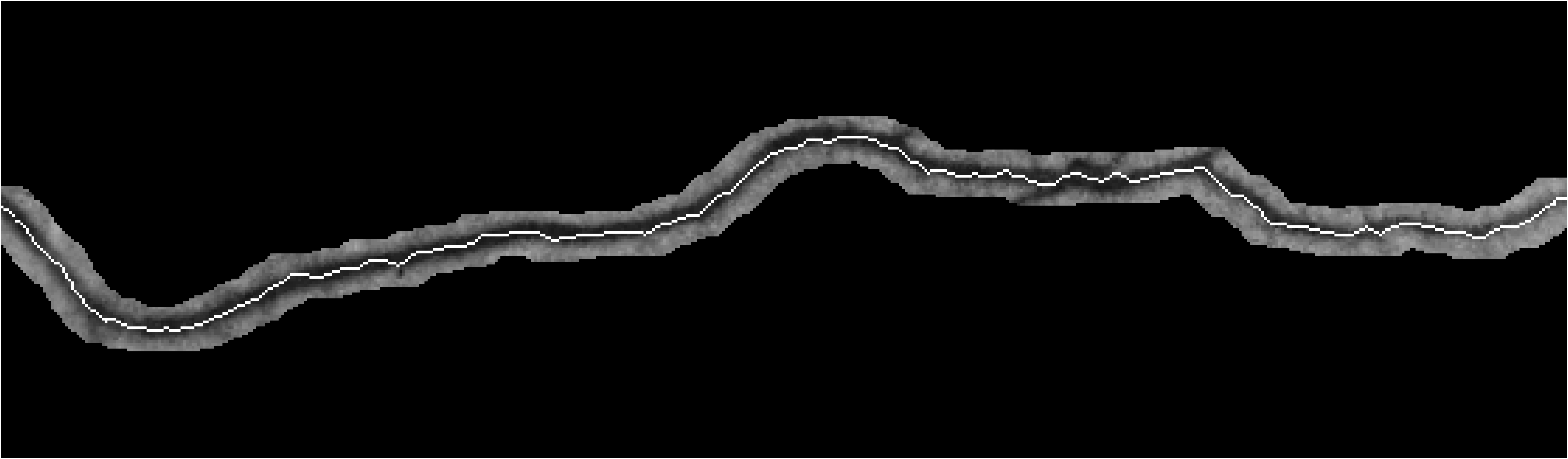

Supplement: S1 Appendix — Figures analogous to those shown in Figs. 3d, 3f, 3h, 3i, and 3j, are included. (ZIP) [file pone.0329379.s001.zip › S1 Appendix/035_Artery/d_ROI with manual trace_035.tif]

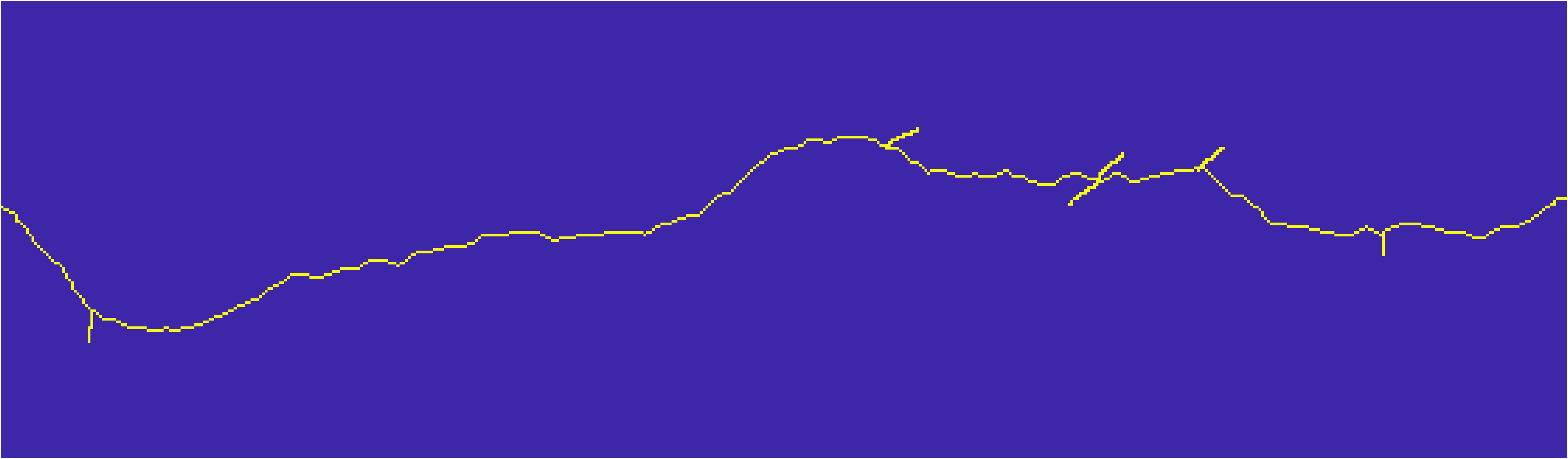

Supplement: S1 Appendix — Figures analogous to those shown in Figs. 3d, 3f, 3h, 3i, and 3j, are included. (ZIP) [file pone.0329379.s001.zip › S1 Appendix/035_Artery/f_Skeleton_035.tif]

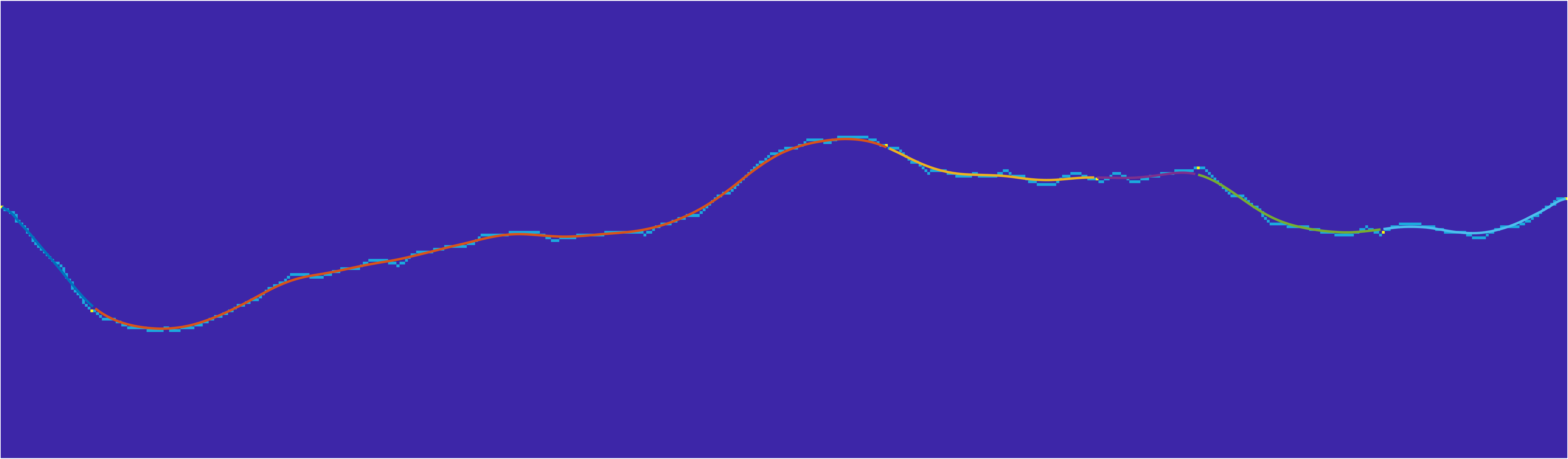

Supplement: S1 Appendix — Figures analogous to those shown in Figs. 3d, 3f, 3h, 3i, and 3j, are included. (ZIP) [file pone.0329379.s001.zip › S1 Appendix/035_Artery/j_partition_035.tif]

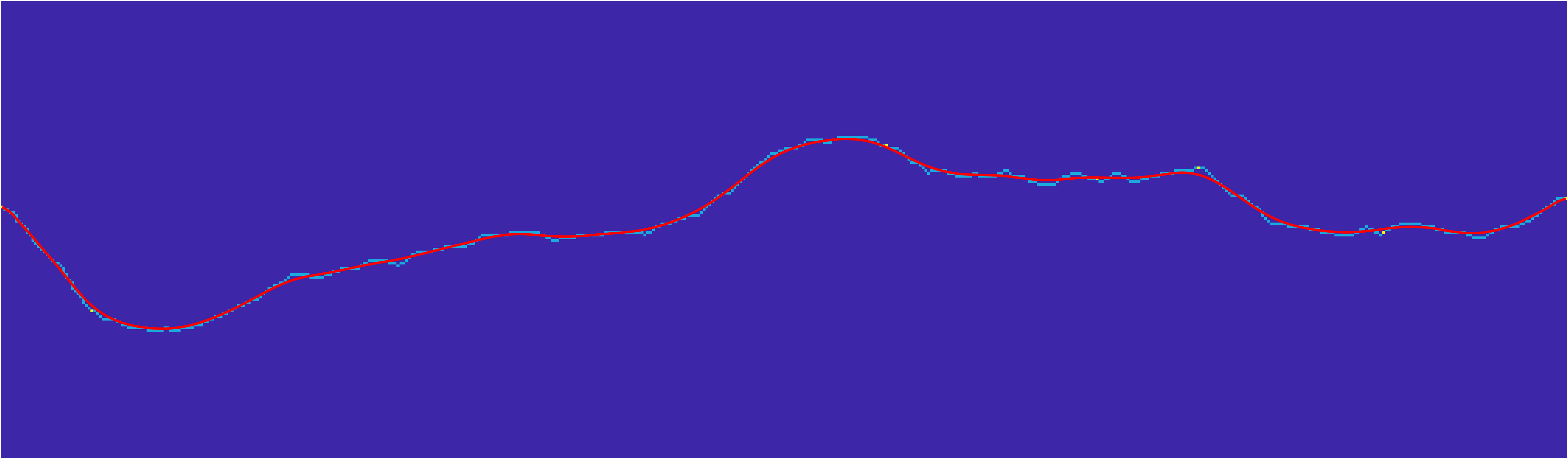

Supplement: S1 Appendix — Figures analogous to those shown in Figs. 3d, 3f, 3h, 3i, and 3j, are included. (ZIP) [file pone.0329379.s001.zip › S1 Appendix/035_Artery/i_smoothed segment_035.tif]

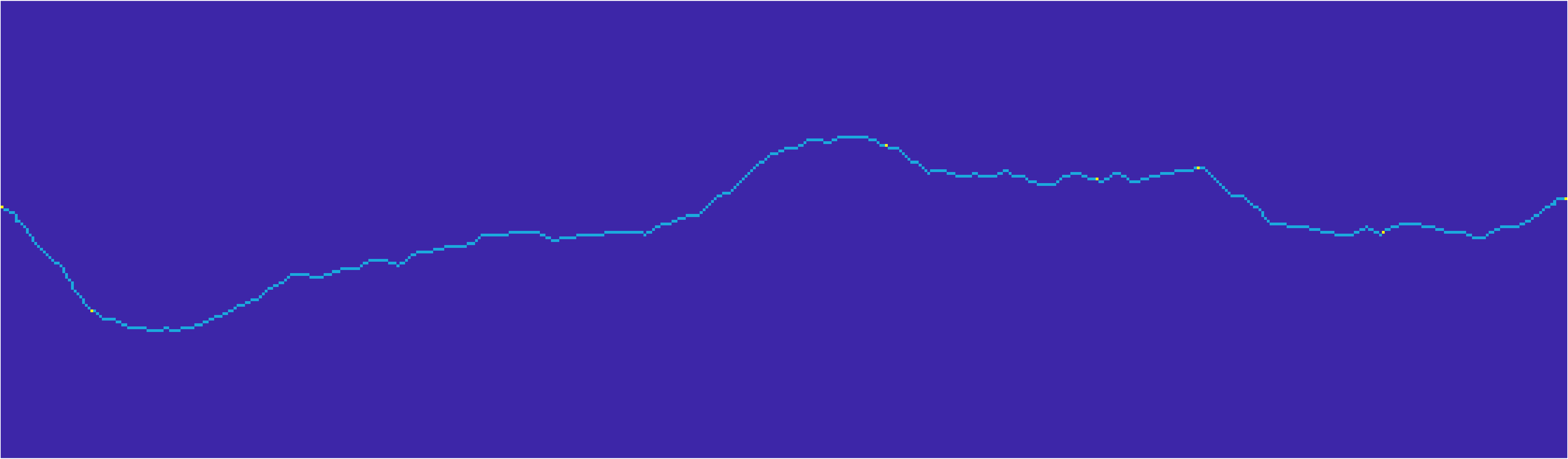

Supplement: S1 Appendix — Figures analogous to those shown in Figs. 3d, 3f, 3h, 3i, and 3j, are included. (ZIP) [file pone.0329379.s001.zip › S1 Appendix/035_Artery/h_centerline and division points_035.tif]
